# Structure-Guided Design of *N*-Methylpropargylamino-Quinazoline Derivatives as Multipotent Agents for the Treatment of Alzheimer’s Disease

**DOI:** 10.3390/ijms24119124

**Published:** 2023-05-23

**Authors:** Barbora Svobodova, Lenka Pulkrabkova, Dawid Panek, Anna Misiachna, Marharyta Kolcheva, Rudolf Andrys, Jiri Handl, Jan Capek, Pavlina Nyvltova, Tomas Rousar, Lukas Prchal, Vendula Hepnarova, Martina Hrabinova, Lubica Muckova, Daniela Tosnerova, Galina Karabanovich, Vladimir Finger, Ondrej Soukup, Martin Horak, Jan Korabecny

**Affiliations:** 1 Biomedical Research Center, University Hospital Hradec Kralove, Sokolska 581, 500 05 Hradec Kralove, Czech Republic; barbora.svobodova@fnhk.cz (B.S.); lenka.pulkrabkova@fnhk.cz (L.P.); dawid.panek@uj.edu.pl (D.P.); lukas.prchal@fnhk.cz (L.P.); vendula.hepnarova@unob.cz (V.H.); hrabinovamartina@gmail.com (M.H.); lubica.muckova@unob.cz (L.M.); vladimirfinger9@gmail.com (V.F.); ondrej.soukup@fnhk.cz (O.S.); 2 Department of Toxicology and Military Pharmacy, Faculty of Military Health Sciences, University of Defence, Trebesska 1575, 500 01 Hradec Kralove, Czech Republic; 3 Department of Physicochemical Drug Analysis, Chair of Pharmaceutical Chemistry, Faculty of Pharmacy, Jagiellonian University Medical College, Medyczna 9, 30-688 Kraków, Poland; 4 Department of Neurochemistry, Institute of Experimental Medicine of the Czech Academy of Sciences, Videnska 1083, 142 20 Prague, Czech Republic; anna.misiachna@iem.cas.cz (A.M.); marharyta.kolcheva@iem.cas.cz (M.K.); 5 Department of Physiology, Faculty of Science, Charles University in Prague, Albertov 6, 128 43 Prague, Czech Republic; 6 Department of Chemistry, Faculty of Science, University Hradec Kralove, Rokitanskeho 62, 500 03 Hradec Kralove, Czech Republic; rudolf.andrys@uhk.cz; 7 Department of Biological and Biochemical Sciences, Faculty of Chemical Technology, University of Pardubice, Studentska 573, 532 10 Pardubice, Czech Republic; jiri.handl@upce.cz (J.H.); jan.capek7@upce.cz (J.C.); pavlina.nyvltova@upce.cz (P.N.); tomas.tousar@upce.cz (T.R.); 8 Department of Organic and Bioorganic Chemistry, Faculty of Pharmacy in Hradec Kralove, Charles University, Akademika Heyrovskeho 1203, 500 05 Hradec Kralove, Czech Republic; tosnerod@faf.cuni.cz (D.T.); karabang@faf.cuni.cz (G.K.)

**Keywords:** Alzheimer’s disease, acetylcholinesterase, enzyme inhibition, *N*-methyl-d-aspartate receptor, monoamine oxidase A/B, multi-target directed ligands

## Abstract

Alzheimer’s disease (AD) is a complex disease with an unknown etiology. Available treatments, limited to cholinesterase inhibitors and *N*-methyl-d-aspartate receptor (NMDAR) antagonists, provide symptomatic relief only. As single-target therapies have not proven effective, rational specific-targeted combination into a single molecule represents a more promising approach for treating AD, and is expected to yield greater benefits in alleviating symptoms and slowing disease progression. In the present study, we designed, synthesized, and biologically evaluated 24 novel *N*-methylpropargylamino-quinazoline derivatives. Initially, compounds were thoroughly inspected by in silico techniques determining their oral and CNS availabilities. We tested, in vitro, the compounds’ effects on cholinesterases and monoamine oxidase A/B (MAO-A/B), as well as their impacts on NMDAR antagonism, dehydrogenase activity, and glutathione levels. In addition, we inspected selected compounds for their cytotoxicity on undifferentiated and differentiated neuroblastoma SH-SY5Y cells. We collectively highlighted **II-6h** as the best candidate endowed with a selective MAO-B inhibition profile, NMDAR antagonism, an acceptable cytotoxicity profile, and the potential to permeate through BBB. The structure-guided drug design strategy applied in this study imposed a novel concept for rational drug discovery and enhances our understanding on the development of novel therapeutic agents for treating AD.

## 1. Introduction

Alzheimer’s disease (AD) is a common cause of dementia that results in memory and cognitive impairments, as well as behavioral and psychiatric disturbances [1]. It affects 10.5 million people in Europe, and the number is expected to rise to 11.5 million by 2025 and 19 million by 2050. Globally, AD affects 56 million people, with projections to increase to 88 million by 2050 [2]. The two main histopathological hallmarks of AD consist of extracellular deposition of amyloid beta protein plaques in the brain tissue and intraneuronal neurofibrillary tangles of the phosphorylated tau protein [3,4]. Despite extensive research and the recent approval of the amyloid beta monoclonal antibodies lecanemab and aducanumab, the efficacy of disease-modifying agents remains under clinicians’ scrutiny [5]. Given the complexity of the disorder, multi-target directed ligands (MTDLs) seem to provide higher therapeutic efficacy by combining different targets to mitigate symptoms and better address disease progression, offering advantages over combination therapy. However, several critical factors have often been ignored while designing novel MTDLs that hamper their success in the early research stage. These include low drug-likeness from the perspective of the physicochemical properties of the drug candidates, the inappropriate selection of potential targets based on the disease stage, mixing receptor/enzyme-specific mechanisms of action with unspecific activity, and multiple unbalanced activities limiting the achievement of pharmacological effects with comparable affinity [6,7].

**Figure 1:**
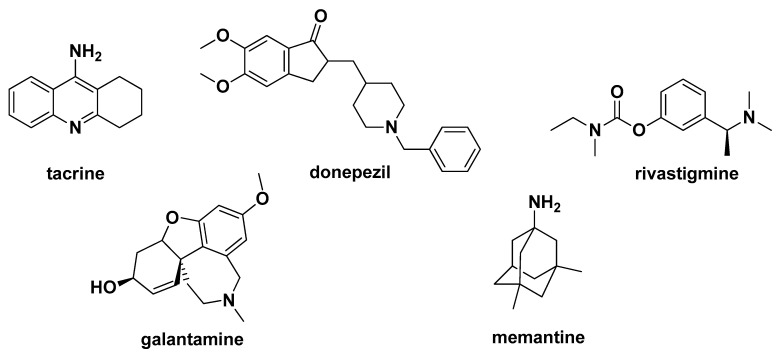
Currently approved drugs for the treatment of AD.

The changes in neurotransmitter levels and respective receptor expressions in patients with AD have been well documented. A consistent loss of cholinergic neurons and a considerable decline in choline acetyltransferase activity are the most prominent phenotypes of AD progression [8]. The loss of cholinergic neurons and acetylcholine receptors (AChRs) in AD makes acetylcholinesterase (AChE, EC 3.1.1.7) a valid therapeutic target. AChE inhibitors, such as tacrine (THA), donepezil, galantamine, and rivastigmine (Figure 1) have been approved for AD symptomatic treatment by reducing AChE activity [9]. In addition to their pro-cognitive properties, mounting evidence suggests other beneficial attributes of AChE inhibitors, including the ability to reduce cortical thinning, basal forebrain atrophy, oxidative stress, amyloid toxicity, and inflammation [10].

The glutamatergic pathway, particularly involving *N*-methyl-d-aspartate receptor (NMDAR) activation, is important for long-term synaptic plasticity and cognitive function [11]. However, overstimulation of extrasynaptic NMDAR by excessive glutamate levels is associated with excitotoxicity and neuronal loss. Antagonists such as memantine (Figure 1) mitigate the excitotoxic effect of glutamate by low-affinity binding to NMDAR to maintain physiological synaptic activity [12]. On the other hand, high-affinity NMDAR antagonists affect not only extrasynaptic but also synaptic NMDARs, which may trigger neuronal death and cause severe side effects in vivo [13]. Furthermore, the blockade of specific NMDAR subtypes, such as GluN1/GluN2B receptors, can provide an effect against Aβ-induced tau phosphorylation and cell toxicity [14].

The monoaminergic system is another disrupted neurotransmission in AD driven by two flavoenzymes, namely monoamine oxidase A (MAO-A) and monoamine oxidase B (MAO-B) [15]. MAOs catalyze the oxidative deamination of primary and some secondary amines and are responsible for neurotransmitter metabolism [16]. MAOs produce toxic metabolites, such as aldehydes and hydrogen peroxide, contributing to neuronal damage and death [17]. Various studies have reported overexpression of MAO-B in AD and its association with cognitive impairment [18]. The activity of both MAO isoforms is also related to serotoninergic and dopaminergic systems, highlighting the importance of MAO inhibitors in AD [17].

**Scheme 1:**
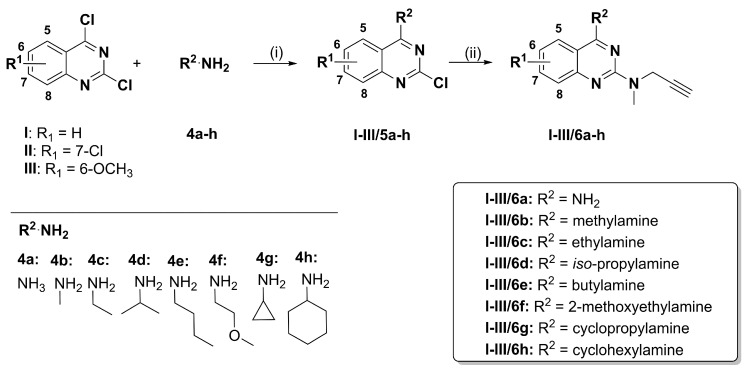
General procedure for quinazoline derivatives **I-III/6a-h**. Reagents and conditions: (i) acetonitrile (10 mL); DIPEA (2 eq.); primary amine (1.1 eq.); 30min-5d; RT; and (ii) methyl(prop-2-yn-1-yl)amine (6 eq.); acetonitrile (5 mL); 1h–4d; 130 °C.

Based on the aforementioned findings, we decided to pursue disrupted neurotransmitter pathways, namely cholinergic, glutamatergic and monoaminergic pathways. The rationale is corroborated by the fact that all these systems are disrupted concomitantly, i.e., at the advanced stage of AD, and are suitable for the treatment of AD [12]. To this end, we developed 24 new *N*-methylpropargylamino-quinazoline derivatives potentially affecting all these pathways (Scheme 1) and evaluated their biological properties towards both cholinesterases (ChEs), represented by AChE and butyrylcholinesterase (BChE; EC 3.1.1.8) and MAO-A/B. Additionally, we investigated the compounds’ effects on NMDAR antagonism, dehydrogenase activity, and glutathione levels. We also screened selected compounds for their ability to cross the blood–brain barrier (BBB), and assessed their cytotoxicity on both undifferentiated and differentiated neuroblastoma SH-SY5Y cells. These findings set forth a new concept of AD drugs and contribute towards understanding the potential utility of these compounds as therapeutic agents for treating AD.

## 2. Results and Discussion

### 2.1. Design Design of Novel Compounds Combining ChE, NMDAR, and MAO-A/B Affinities

**Figure 2:**
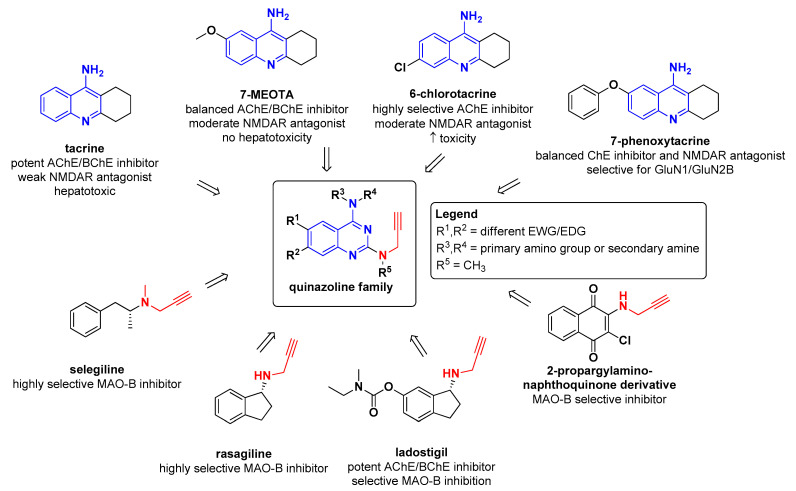
Rational design of novel MTDLs from the quinazoline family combining ChE, MAO-A/B inhibition properties, and NMDAR antagonism.

The design of new molecules pursued a structure-guided drug discovery scheme, following pharmacophore properties responsible for the specific activities, i.e., (i) 4-aminoquinazoline derivative with an affinity towards ChE and NMDAR [19]; and (ii) with attached *N*-methylpropargylamine moiety determining MAO-A/B inhibition ability (Figure 2) [20]. The activity enhancement towards ChE/NMDAR can be achieved by substitution in two different regions, namely: (i) in the aromatic part with emphasis on the positions 6- or 7- of quinazoline moiety by electron-donating (EDG) or electron-withdrawing groups (EWG); and (ii) by substitution of the halogen to primary amines in position 4- of the core scaffold. Regarding the aromatic substitution, these changes proved to have a critical role in driving: (i) the selectivity (AChE vs. BChE); (ii) ChE activity; and (iii) the toxicity profile [21,22,23]. To name a few examples, methoxy group introduction to position 6- of quinazoline heterocycle mimics a less-toxic tacrine derivative (7-MEOTA) [21]; whereas the introduction of the chlorine atom into position 7- of quinazoline heterocycle is analogous to 6-chlorotacrine, one of the most active AChE inhibitors developed to date [22]. These observations can be expanded to 4-chloro substitution of quinazoline moiety, where the attachment of small aliphatic chains or alicyclic substituents improved the ChE activity [24]. Our recent data also correlate well between affinity to NMDARs and substitution in the aromatic region, where, for instance, the attachment of bulky moieties into position 7- of tacrine moiety (analogous to position 6- of quinazoline moieties) improved both the affinity towards NMDARs and selectivity towards GluN1/GluN2A over GluN1/GluN2B receptors [25]. *N*-Methylpropargylamine or propargylamine moieties-containing drugs are highly potent and selective irreversible MAO-A/B inhibitors that can be found in various CNS drugs such as selegiline (used for the treatment of Parkinson’s disease and major depressive disorder), rasagiline (Parkinson’s disease therapy), or ladostigil. The latter is the only MTDL against AD that reached II phase clinical trials, discovered by merging an approach to exploiting rivastigmine and rasagiline scaffolds [26]. Accordingly, the introduction of *N*-methylpropargylamine appendages into novel molecules to position 2- of quinazoline within this study was driven by the synthetic feasibility [27], hand-in-hand with the tolerance of this position to aliphatic moieties, such as bicyclic cages typical for highly active and AChE-selective huprine derivatives [28], chiral terpenic compounds [29], or more simple aliphatic moieties [30].

### 2.2. In Silico Prediction of CNS and Oral Availability

**Figure 3:**
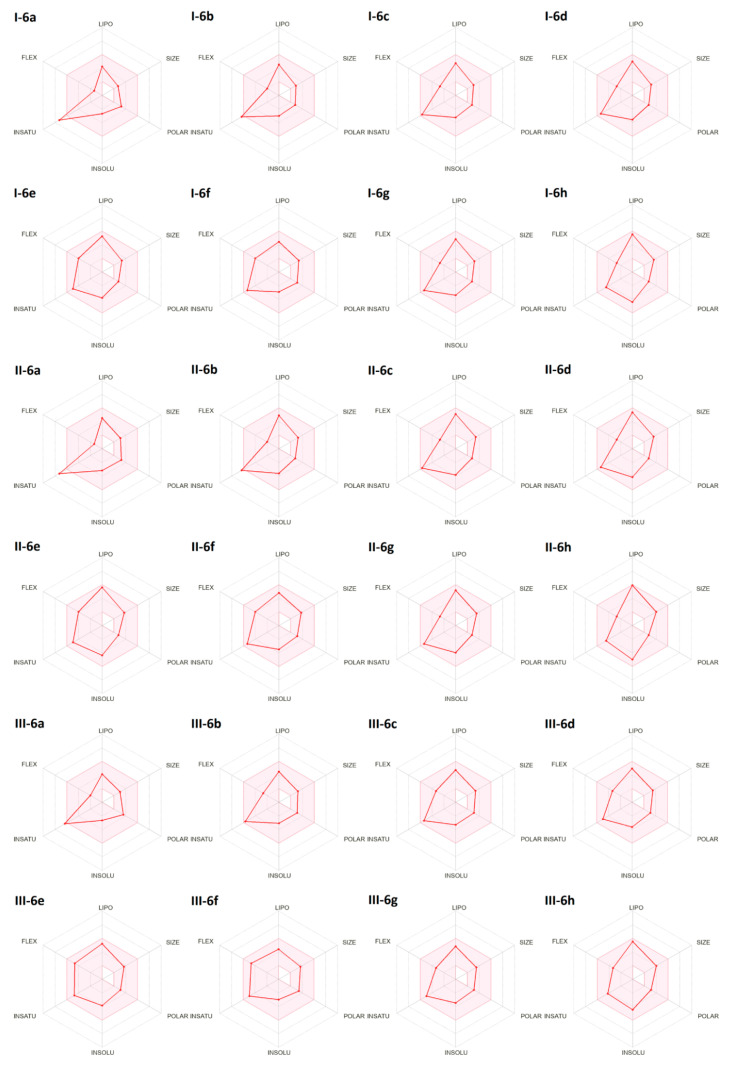
The predicted bioavailability rendered by radar chart for the designed *N*-methylpropargylamino-quinazoline derivatives **I-III/6a-6h**. Each compound is defined by area counting lipophilicity (LIPO), size, polarity (POLAR), solubility (INSOLU), saturation (INSATU), and flexibility (FLEX).

All the newly designed compounds were initially screened in silico to predict their peroral and/or CNS availabilities. These features are essentially viable to consider in the drug discovery process for potential anti-AD therapeutics and should be estimated before synthesis. For this purpose, the in silico pharmacokinetics, drug-likeness and ADME (absorption, distribution, metabolism, excretion) prediction was applied to compounds **I-III/6a-6h** using a web-based tool SwissADME (http://www.swissadme.ch/, accessed on 20 October 2022) [31,32]. We also applied pan-assay interference compounds (PAINS) analysis according to the Free ADME-Tox Filtering Tool (FAF-Drugs4) program (http://fafdrugs4.mti.univ-paris-diderot.fr/, accessed on 20 October 2022). THA and memantine were used as references with known CNS status and pharmacokinetic profiles [21,33,34]. Data are presented in Figure 3, Table 1 and Table S1 (Supplementary Information).

Figure 3 illustrates the ideal chemical space for oral bioavailability, which is determined by a combination of factors including lipophilicity (LIPO), size, polarity (POLAR), solubility (INSOLU), saturation (INSATU), and flexibility (FLEX). The pink area represents this optimum range, while the red lines indicate the predicted state for the designed compounds [31,32]. Calculated parameters for all the compounds, except for **I-6a**, **II-6a** and **III-6a**, fully fall within the pink area, suggesting that the compounds are endowed with high oral bioavailability [37]. Additionally, the BOILED-Egg method, which predicts gastrointestinal absorption and penetration through the BBB, also supports a positive correlation for absorption and brain distribution. Given the importance of physicochemical parameters, we also predicted and calculated the potential CNS availability by applying the so-called blood–brain barrier (BBB) score [21]. All the proposed structures are estimated to cross the BBB reaching BBB score values ≥ 4. Moreover, the predicted water solubility of all newly designed structures shows good results with clogS values ≥ −3, suggesting good water solubility. All the designed compounds were screened for known classes of assay interference (PAINS) compounds [38] with a negative result. Finally, all compounds also fulfill the drug-likeness criteria according to various pharmacological models defined by pharmaceutical companies such as Veber’s (GSK) [39], Lipinski’s (Pfizer) [40], Egan’s (Pharmacopeia) [41], Ghose’s (Amgen) [42], and Muegge’s (Bayer) [43] (Table S2; Supplementary Information). In summary, derivatives **I-III/6a-6h** unambiguously showed high predictive peroral bioavailability, BBB permeation, and acceptable pharmacokinetic profiles, making the compounds prospective candidates to be investigated under in vitro conditions with potential follow-up in in vivo studies.

### 2.3. Chemical Synthesis of Novel Quinazoline Derivatives

All the newly designed target compounds were synthesized as outlined in Scheme 1. Compounds **I-III/6a-h** were prepared in a two-step chemical synthesis according to the previously described method [27]. The initial step involved the nucleophilic substitution of starting aromatic-substituted dichloroquinazoline (**I-III**) in the presence of primary amine (**4a-h**) and DIPEA. The reaction proceeded under mild conditions selectively to position 4-. Finally, *N*-methylpropargylamine group was introduced using nucleophilic substitution conditions in acetonitrile, resulting in **I-III/6a-6h** as bases in good overall yields (18–74%). All the final compounds were characterized by ^1^H and ^13^C NMR, HRMS, and LC-UV analysis confirmed their purity to be higher than 95% (Supplementary Information).

### 2.4. Evaluation of Cholinesterase Inhibitory Activity

*N*-Methylpropargylamine-quinazolines were tested for their inhibitory potency against human AChE (*h*AChE) and human BChE (*h*BChE) enzymes using the modified spectrophotometric method of Ellman et al. [44,45,46]. It should be noted that IC_50_ values were not determined for the tested compounds, as their maximum inhibition was below the 50% limit, reaching only up to 15% when screened at 1 μM. The percentage inhibition values of all compounds for *h*AChE/*h*BChE are summarized in Table S3 (Supplementary information).

### 2.5. In Vitro Inhibition of MAO-A/B

All the final compounds (**I-III/6a-6h**) were also initially screened at two different concentrations (1 and 10 μM) against both isoforms of human MAOs (Figure S1; Supplementary information). Given the preliminary results from the screening, only the compounds exerting a decrease in the activity below 50% were selected for IC_50_ determination. The IC_50_ values were established for the five most active compounds **I-6e**, **I-6h**, **II-6e**, **II-6h** and **III-6a** and these values are given in Table 2. Clorogyline (an irreversible and selective inhibitor of MAO-A) and pargyline (an irreversible and selective inhibitor of MAO-B) were used as reference compounds. Out of the five derivatives, three (**I-6h, I-6e, III-6a**) inhibited MAO-A even at the submicromolar range with IC_50_ values ranging between 0.62–2.60 μM, although not reaching clorgyline inhibition potency (IC_50_ = 0.05 μM). Regarding MAO-B inhibition, compounds **I-6h**, **II-6e** and **II-6h** were the most active, resulting in a micromolar to sub-micromolar inhibition range. The results were also encouraging from the perspective of the selectivity profile, where compounds **I-6e** and **III-6a** can be classified as *h*MAO-A selective inhibitors. In contrast, derivatives **II-6e** and **II-6h** are *h*MAO-B selective inhibitors. Note that **I-6h** turned out to be an inhibitor of both *h*MAO isoforms, with a preference for *h*MAO-B inhibition. The most effective *h*MAO-B inhibition was manifested by **II-6h** (IC_50_ = 0.33 μM). It can be roughly estimated that MAO-B inhibition is coined to bulkier, non-polar moieties, such as *n-*butyl or cyclohexyl substituents attached to the amino group in position 4- of quinazoline core. 4-Aminoquinazoline **III-6a** was the only one from the amino-unsubstituted members tolerating MAO-A inhibition.

### 2.6. Study of MAO-A/B Inhibition Mechanism

**Figure 4:**
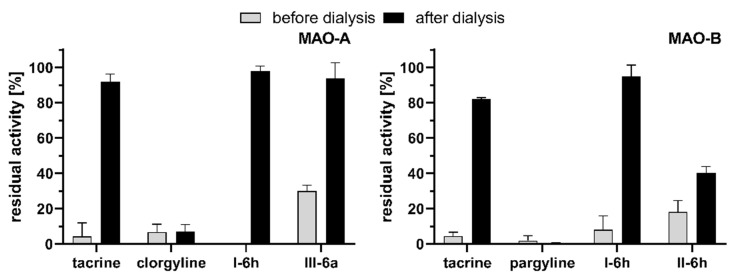
Reversible/irreversible inhibitory action of **I-6h**, **III-6a** and **II-6h** towards *h*MAO-A/B.

**Figure 5:**
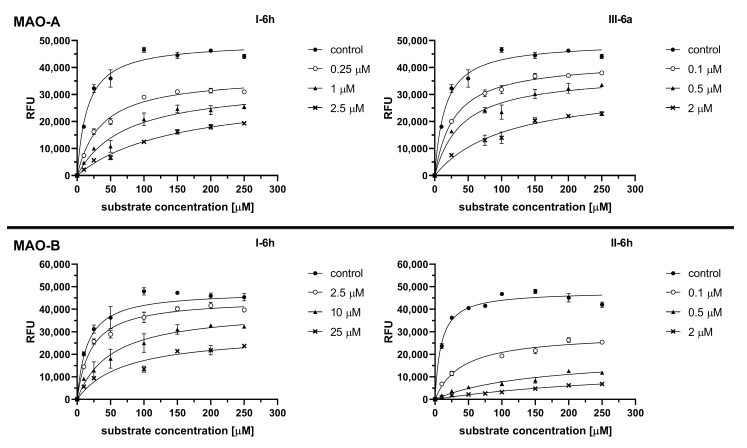
Determination of the enzyme inhibition kinetics for compounds **I-6h**, **III-6a** on MAO-A and for **I-6h**, **II-6h** on MAO-B.

The mechanism of *h*MAO-A and *h*MAO-B inhibition was deeply investigated for compounds **I-6h**, **III-6a** and **I-6h**, **II-6h**, respectively. First, the reversible/irreversible inhibitory action towards MAO enzymes was verified (Figure 4). The results showed that compounds **I-6h**, **III-6a** and tacrine acted as fully reversible inhibitors of both *h*MAO-A and *h*MAO-B, as there was an almost complete recovery of enzyme activity after sample dialysis. Therefore, inhibition caused by these compounds is likely to be mediated by weak non-covalent interactions (e.g., hydrophobic interactions). Clorgyline and pargyline acted as fully irreversible inhibitors as there was no recovery of enzyme activity [47]. Compounds **II-6h** were characterized by a somewhat different pattern of behavior, as there was only a partial recovery of the original MAO-B enzyme activity (increase by ~20%). These results indicate the formation of a stronger/tight bond between the inhibitor and the enzyme compared to compounds **I-6h** and **III-6a**. The MAOs were subsequently incubated with selected compounds at different concentrations and evaluated by non-linear regression according to Michaelis–Menten (Figure 5). In all cases, the compounds showed characteristics of a mixed mode inhibition, when K_m_ increases and V_max_ decreases with increasing inhibitor concentration. Thus, the tested compounds displayed affinity to the free enzyme as well as to the enzyme–substrate complex.

### 2.7. Evaluation of Antagonist Activity towards NMDAR

We further investigated the inhibitory effect of novel derivatives at a selected subtype of NMDAR associated with neurodegeneration, namely the GluN1/GluN2B receptor [48]. We tested the derivatives by the whole-cell patch-clamp method at a concentration of 10 μM under steady-state conditions in the presence of the saturating concentrations of NMDAR (co-)agonists (L-glutamate and glycine). Our measurements showed that all tested derivatives exhibited inhibitory activity toward the GluN1/GluN2B receptor, ranging from ~18 to ~63% (Table 3). As memantine showed ~95% and 7-MEOTA ~60% inhibitory activity under these conditions, it is evident that the new derivatives exhibit promising inhibitory activity at NMDAR, although to a lesser extent than memantine.

### 2.8. Prediction of BBB Permeability

To confirm the in silico predicted ability of compounds **I-6e**, **I-6h**, **II-6e**, **II-6h**, **III-6a** to penetrate through the BBB, the parallel artificial membrane-permeability assay (PAMPA) was used [49,50]. The assay validation was performed using standard compounds whose CNS status is experimentally known from in vivo conditions [51,52,53]. The results of commercial drugs and new investigational compounds are summarised in Table 4. Based on obtained results, selected compounds **I-6e**, **I-6h** and **II-6h** demonstrated a high probability of crossing BBB via passive diffusion, whereas the derivatives **III-6a** and **II-6e** did not penetrate the artificial barrier simulating the BBB.

### 2.9. In Vitro Neurotoxicity

**Figure 6:**
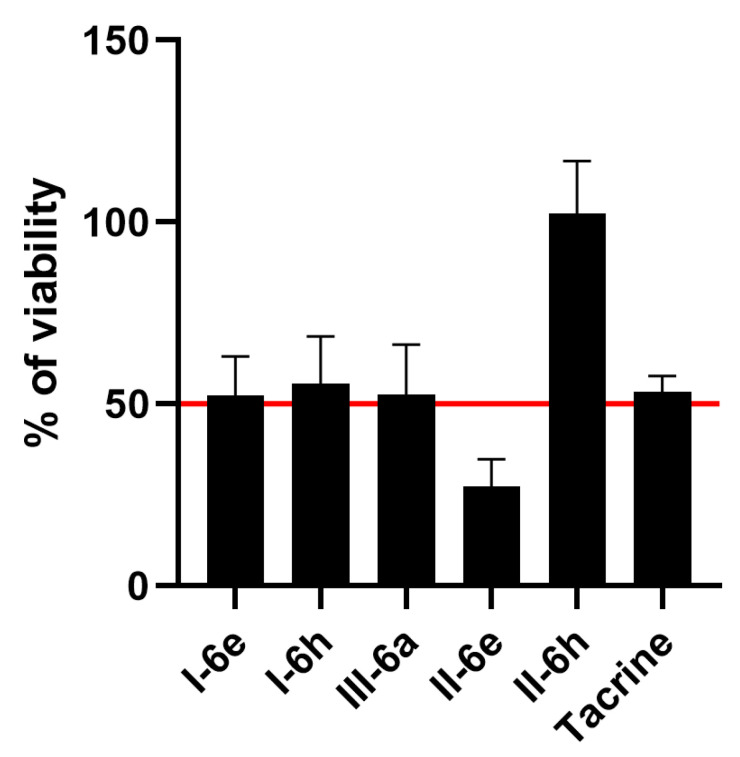
The viability of differentiated SH-SY5Y cells treated with tested compounds in the concentration corresponding to the IC_50_ values (red line) obtained from undifferentiated SH-SY5Y cells.

To evaluate the neurotoxicity of selected derivatives **I-6e**, **I-6h**, **II-6e**, **II-6h** and **III-6a**, we tested the cytotoxic effects of these compounds on the SH-SY5Y cells in both undifferentiated and differentiated forms (ECACC, 94030304) using the MTT assay (MTT: (3-(4,5-dimethylthiazol-2-yl)-2,5-diphenyl-tetraziolium bromide, Sigma-Aldrich, St. Louis, MO) [54]. The results are presented in Table 5. All the tested compounds revealed high micromolar-range cytotoxicity towards undifferentiated SH-SY5Y cell lines, highlighting **I-6e** and **II-6e** as the least toxic ones. Furthermore, we tested their effect on the cell viability in the model of mature human neurons represented by the differentiated SH-SY5Y (Figure 6). The compounds were applied in the concentration equal to IC_50_ from the undifferentiated model. Interestingly, while **I-6e**, **I-6h** and **III-6a** showed no difference from the undifferentiated model, **II-6e** displayed increased toxicity. On the contrary, **II-6h** lost the cytotoxic effect in the differentiated model which further raised interest in this compound.

### 2.10. Assessment of Dehydrogenase Activity and Glutathione Levels in Neuronal Cells

Next, we tested the effects of the compounds on intracellular dehydrogenase activity and glutathione levels. The SH-SY5Y cells were treated with **I-6a**, **I-6h**, **II-6e**, **II-6h**, and **III-6a** (100 μM) for 6 h and 24 h. To evaluate the effects of the compounds on cells, we measured total intracellular dehydrogenase activity as a marker of cellular viability and glutathione levels for evaluation of oxidative stress [55] (Table 6).

After incubation, the dehydrogenase activity and glutathione level were decreased in cells treated with most of the tested compounds. In contrast, compound **III-6a** increased dehydrogenase activity after 24 h of treatment. Compound **II-6h** induced the most harmful effect, as can be seen in both the reduction in dehydrogenase activity and glutathione depletion. Based on these results, compounds **III-6a** and **II-6h** were chosen for further analysis to compare compounds exhibiting low and high levels of toxicity.

**Figure 7:**
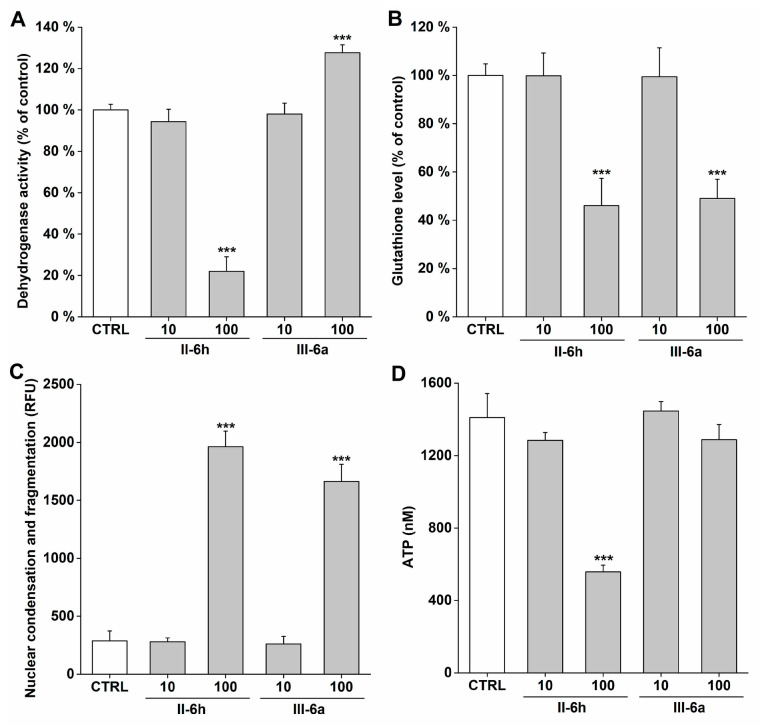
Characterizing **II-6h** and **III-6a**-induced toxicity in SH-SY5Y cells. SH-SY5Y cells were incubated with **II-6h** (10 and 100 µM) and **III-6a** (10 and 100 µM) for 24 h. After treatment dehydrogenase activity using the WST-1 test (**A**); glutathione levels using monochlorobimane assay (**B**); nuclear condensation and fragmentation using the H33258 spectrofluorimetric assay (**C**); and ATP level using luciferase assay (**D**) were measured. The data are presented as mean ± SD (**A**,**B**) and mean ± SEM (**C**,**D**). (*** *p* < 0.001, vs. untreated cells).

Thus, SH-SY5Y cells were incubated with **II-6h** and **III-6a** (10 and 100 µM) for 24 h. After the treatment, dehydrogenase activity, glutathione levels, nuclear condensation and fragmentation, and ATP levels were measured (Figure 7). According to the findings from the dehydrogenase activity measurement (Figure 7A), we found that both compounds at 100 µM caused significant changes in dehydrogenase activity compared to untreated cells. The decrease in dehydrogenase activity was found in cells treated with 100 µM **II-6h** (*p* < 0.001). In addition, we found a significant decrease in GSH levels in cells treated with 100 µM **II-6h** and **III-6a** to be 46 ± 11% (*p* < 0.001) and 49 ± 8% (*p* < 0.001), respectively, compared to untreated cells. After incubation of the cells with 10 µM **II-6h** and 10 µM **III-6a**, no significant changes in dehydrogenase activity or glutathione levels were detected. The detection of nuclear condensation and fragmentation in SH-SY5Y cells exposed to **II-6h** and **III-6a** (10 and 100 µM) (Figure 7C) showed a significant increase only in 100 µM **II-6h** and 100 µM **III-6a**, corresponding with the findings of GSH depletion. Finally, we measured the intracellular ATP levels, and observed partial exhaustion of ATP only after treatment with 100 µM **II-6h**, corresponding with the finding of decreased dehydrogenase activity in these cells.

## 3. Materials and Methods

### 3.1. General Methods and Chemistry

All chemical solvents and reagents were used in the highest available purity without further purification, and were purchased from Sigma-Aldrich (Prague, Czech Republic) or FluoroChem (Hadfield, UK). The reactions were monitored by thin layer chromatography (TLC) on silica gel plates (60 F254, Merck, Prague, Czech Republic) and the spots were visualized by ultraviolet light (254 nm). Purification of the crude products was carried out using columns of silica gel (silica gel 100, 0.063–0.200 mm, 70–230 mesh ASTM, Fluka, Prague, Czech Republic) and PuriFlash GEN5 column, 5.250 (Interchim, Montluçon, France) (silica gel 100, 60 Å, 230–400 mesh ASTM, Sigma-Aldrich, Prague, Czech Republic). NMR spectra were recorded in deuterated chloroform (CDCl_3_), deuterated methanol (CD_3_OD) and deuterated dimethyl sulfoxide (DMSO-d_6_) on Bruker Avance NEO 500 MHz spectrometer (499.87 MHz for ^1^H NMR, and 125.71 MHz for ^13^C NMR). Chemical shifts (*δ*) are reported in parts per millions (ppm) and spin multiplicities are given as broad singlet (bs), doublet (d), doublet of doublet (dd), triplet (t), quartet (q), pentet (p), or multiplet (m). Coupling constants (*J*) are reported in Hz. All NMR spectra are provided in Supplementary Information (Figures S2–S89). The synthesized compounds were analyzed by LC-MS system consisting of UHPLC Dionex Ultimate 3000 RS coupled with Q Exactive Plus mass spectrometer to obtain high resolution mass spectra (Thermo Fisher Scientific, Bremen, Germany). Melting points were measured using automated melting point recorder M-565 (Büchi, Flawil, Switzerland). The final compounds were analyzed by UHPLC mass spectrometry as above. Gradient LC analysis confirmed >95% purity. All HRMS spectra and LC-UV chromatograms are provided in Supplementary Information (Figures S90–S137).

#### 3.1.1. General Procedure for the Preparation of Compounds **I-III/5a-5h**

Compounds **I-III/5a-5g** were synthesized according to the method shown in Scheme 1. Commercially available starting material **I-III** (2.5 mmol; 1.0 eq.) were charged into a 50 mL flask. Acetonitrile (10 mL) and DIPEA (2.0 eq.) were then added to the flask. Primary amine **4a-h** (2.7 mmol; 1.1 eq.) was added to the reaction mixture. The reaction mixture was stirred at room temperature for 1 h–5 days. After completion of the reaction (observed by TLC), the solvent was evaporated in vacuo and the crude intermediate **I-III/a5a-h** was purified by flash chromatography using PE/EA (7:3) as mobile phase.

2-chloroquinazolin-4-amine (**I-5a**)

Yield: 95%. Brown viscous oil. ^1^H NMR (500 MHz, DMSO-*d*_6_): δ 8.32 (bs, 2H), 8.23 (dd, *J* = 8.2, 1.4 Hz, 1H), 7.83–7.76 (m, 1H), 7.61 (dd, *J* = 8.4, 1.2 Hz, 1H), 7.55–7.48 (m, 1H) ppm; ^13^C NMR (126 MHz, DMSO-*d*_6_): δ 164.12, 157.48, 151.30, 134.38, 126.99, 126.39, 124.36, 113.48 ppm.

2-chloro-*N*-methylquinazolin-4-amine (**I-5b**)

Yield: 89%. Brown viscous oil. ^1^H NMR (500 MHz, DMSO-*d*_6_): δ 8.86–8.75 (m, 1H), 8.22 (dd, *J* = 8.3, 1.3 Hz, 1H), 7.85–7.77 (m, 1H), 7.64 (dd, *J* = 8.4, 1.2 Hz, 1H), 7.58–7.51 (m, 1H), 3.03 (d, *J* = 4.0 Hz, 3H) ppm; ^13^C NMR (126 MHz, DMSO-*d*_6_): δ 161.99, 157.56, 150.50, 133.95, 127.09, 126.53, 123.39, 114.13, 28.43 ppm.

2-chloro-*N*-ethylquinazolin-4-amine (**I-5c**)

Yield: 93%. Brown viscous oil. ^1^H NMR (500 MHz, DMSO-*d*_6_): δ 8.67 (t, *J* = 5.5 Hz, 1H), 8.18 (dd, *J* = 8.3, 1.3 Hz, 1H), 7.72 (ddd, *J* = 8.4, 7.0, 1.3 Hz, 1H), 7.54 (dd, *J* = 8.4, 1.2 Hz, 1H), 7.46 (ddd, *J* = 8.3, 6.9, 1.2 Hz, 1H), 3.51–3.43 (m, 2H), 1.17 (t, *J* = 7.2 Hz, 3H) ppm; ^13^C NMR (126 MHz, DMSO-*d*_6_): δ 161.34, 157.56, 150.68, 133.97, 127.08, 126.43, 123.53, 114.04, 36.21, 14.44 ppm.

2-chloro-*N*-(propan-2-yl)quinazolin-4-amine (**I-5d**)

Yield: 75%. Brown viscous oil. ^1^H NMR (500 MHz, DMSO-*d*_6_): δ 8.39 (d, *J* = 7.6 Hz, 1H), 8.34 (dd, *J* = 8.3, 1.4 Hz, 1H), 7.81–7.72 (m, 1H), 7.60 (dd, *J* = 8.3, 1.2 Hz, 1H), 7.55–7.48 (m, 1H), 4.52–4.36 (m, 1H), 1.27 (d, *J* = 6.6 Hz, 6H) ppm; ^13^C NMR (126 MHz, DMSO-*d*_6_): δ 160.66, 157.55, 150.81, 133.96, 127.05, 126.28, 123.77, 113.97, 43.06, 22.23 ppm.

*N*-butyl-2-chloroquinazolin-4-amine (**I-5e**)

Yield: 96%. Brown viscous oil. ^1^H NMR (500 MHz, DMSO-*d*_6_): δ 8.69 (t, *J* = 5.5 Hz, 1H), 8.27 (dd, *J* = 8.3, 1.4 Hz, 1H), 7.78 (ddd, *J* = 8.3, 7.0, 1.3 Hz, 1H), 7.61 (dd, *J* = 8.4, 1.2 Hz, 1H), 7.53 (ddd, *J* = 8.3, 7.0, 1.3 Hz, 1H), 3.54–3.47 (m, 2H), 1.69–1.60 (m, 2H), 1.46–1.30 (m, 2H), 0.93 (t, *J* = 7.4 Hz, 3H) ppm; ^13^C NMR (126 MHz, DMSO-*d*_6_): δ 161.53, 157.56, 150.72, 133.96, 127.09, 126.42, 123.56, 114.03, 40.98, 30.82, 20.13, 14.19 ppm.

2-chloro-*N*-(2-methoxyethyl)quinazolin-4-amine (**I-5f**)

Yield: 98%. Brown viscous oil. ^1^H NMR (500 MHz, DMSO-*d*_6_): δ 8.81 (t, *J* = 5.6 Hz, 1H), 8.29 (dd, *J* = 8.2, 1.3 Hz, 1H), 7.80 (ddd, *J* = 8.3, 7.0, 1.3 Hz, 1H), 7.62 (dd, *J* = 8.3, 1.2 Hz, 1H), 7.54 (ddd, *J* = 8.2, 7.0, 1.3 Hz, 1H), 3.69 (q, *J* = 5.6 Hz, 2H), 3.59 (t, *J* = 5.6 Hz, 2H), 3.29 (s, 3H) ppm; ^13^C NMR (126 MHz, DMSO-*d*_6_): δ 161.68, 157.37, 150.75, 134.10, 127.12, 126.55, 123.61, 114.02, 70.13, 58.46, 41.01 ppm.

2-chloro-*N*-cyclopropylquinazolin-4-amine (**I-5g**)

Yield: 94%. Brown viscous oil. Product **(I-5g**) was used directly for the next step of synthesis.

2-chloro-*N*-cyclohexylquinazolin-4-amine (**I-5h**)

Yield: 91%. Brown viscous oil. Product (**I-5h**) was used directly for the next step of synthesis.

2,7-dichloroquinazolin-4-amine (**II-5a**)

Yield: 89%. Brown viscous oil. ^1^H NMR (500 MHz, DMSO-*d*_6_): δ 8.47 (bs, 2H), 8.26 (d, *J* = 8.8 Hz, 1H), 7.68 (d, *J* = 2.1 Hz, 1H), 7.58 (dd, *J* = 8.9, 2.1 Hz, 1H) ppm; ^13^C NMR (126 MHz, DMSO-*d*_6_): δ 163.83, 158.65, 152.25, 139.04, 126.81, 126.50, 125.91, 112 ppm.22.

2,7-dichloro-*N*-methylquinazolin-4-amine (**II-5b**)

Yield: 83%. Brown viscous oil. ^1^H NMR (500 MHz, DMSO-*d*_6_): δ 8.91 (q, *J* = 4.6 Hz, 1H), 8.21 (d, *J* = 8.8 Hz, 1H), 7.67 (d, *J* = 2.2 Hz, 1H), 7.58 (dd, *J* = 8.7, 2.2 Hz, 1H), 2.99 (d, *J* = 4.6 Hz, 3H) ppm; ^13^C NMR (126 MHz, DMSO-*d*_6_): δ 161.65, 158.70, 151.48, 138.58, 126.89, 126.00, 125.55, 112.89, 28.47 ppm.

2,7-dichloro-*N*-ethylquinazolin-4-amine (**II-5c**)

Yield: 87%. Brown viscous oil. ^1^H NMR (500 MHz, DMSO-*d*_6_): δ 8.85 (t, *J* = 5.4 Hz, 1H), 8.27 (d, *J* = 8.8 Hz, 1H), 7.67 (d, *J* = 2.2 Hz, 1H), 7.58 (dd, *J* = 8.8, 2.1 Hz, 1H), 3.53 (m, *J* = 7.2, 2.7 Hz, 2H), 1.23 (t, *J* = 7.2 Hz, 3H) ppm; ^13^C NMR (126 MHz, DMSO-*d*_6_): δ 161.02, 160.94, 158.70, 151.67, 138.62, 126.79, 125.98, 125.71, 112.79, 112.73, 36.34, 36.22, 14.32, 14.30 ppm.

2,7-dichloro-*N*-(propan-2-yl)quinazolin-4-amine (**II-5d**)

Yield: 92%. Brown viscous oil. ^1^H NMR (500 MHz, Methanol-*d*_4_): δ 8.07–8.02 (m, 1H), 7.46–7.42 (m, 1H), 7.35 (dd, *J* = 8.8, 2.1 Hz, 1H), 4.46 (hept, *J* = 6.6 Hz, 1H), 1.23 (d, *J* = 6.6 Hz, 6H) ppm; ^13^C NMR (126 MHz, Methanol-*d*_4_): δ 160.29, 158.87, 151.02, 139.20, 126.27, 124.73, 124.33, 112.01, 43.15, 20.67 ppm.

*N*-butyl-2,7-dichloroquinazolin-4-amine (**II-5e**)

Yield: 96%. Brown viscous oil. ^1^H NMR (500 MHz, Methanol-*d*_4_): δ 7.95 (d, *J* = 8.8 Hz, 1H), 7.45 (d, *J* = 2.0 Hz, 1H), 7.34 (dd, *J* = 8.8, 2.1 Hz, 1H), 3.50 (t, *J* = 7.3 Hz, 2H), 1.63–1.56 (m, 2H), 1.34 (m, *J* = 7.4 Hz, 2H), 0.89 (t, *J* = 7.4 Hz, 3H) ppm; ^13^C NMR (126 MHz, Methanol-*d*_4_): δ 161.11, 158.86, 150.91, 139.21, 126.34, 124.78, 124.13, 112.02, 40.87, 30.56, 19.78, 12.76 ppm.

2,7-dichloro-*N*-(2-methoxyethyl)quinazolin-4-amine (**II-5f**)

Yield: 94%. Brown viscous oil. ^1^H NMR (500 MHz, Methanol-*d*_4_): δ 7.98 (d, *J* = 8.8 Hz, 1H), 7.48–7.45 (m, 1H), 7.36 (dd, *J* = 8.8, 2.1 Hz, 1H), 3.70 (t, *J* = 5.5 Hz, 2H), 3.56 (t, *J* = 5.5 Hz, 2H), 3.29 (s, 3H) ppm; ^13^C NMR (126 MHz, Methanol-*d*_4_): δ 161.30, 158.67, 150.97, 139.35, 126.45, 124.84, 124.22, 112.02, 69.95, 57.58, 40.73, 29.27 ppm.

2,7-dichloro-*N*-cyclopropylquinazolin-4-amine (**II-5g**)

Yield: 98%. Brown viscous oil. Product (**II-5g**) was used directly for the next step of synthesis.

2,7-dichloro-*N*-cyclohexylquinazolin-4-amine (**II-5h**)

Yield: 91%. Brown viscous oil. ^1^H NMR (500 MHz, DMSO-*d*_6_): δ 8.49 (d, *J* = 7.8 Hz, 1H), 8.38 (d, *J* = 8.9 Hz, 1H), 7.65 (d, *J* = 2.2 Hz, 1H), 7.57 (dd, *J* = 8.8, 2.2 Hz, 1H), 4.15–4.02 (m, 1H), 1.93 (m, *J* = 9.3, 3.3 Hz, 2H), 1.82–1.73 (m, 2H), 1.69–1.60 (m, 1H), 1.45–1.27 (m, 4H), 1.23–1.10 (m, 1H) ppm; ^13^C NMR (126 MHz, DMSO-*d*_6_): δ 160.32, 158.70, 151.84, 138.62, 126.62, 126.00, 125.91, 112.72, 50.44, 32.16, 25.66, 25.33 ppm.

2-chloro-6-methoxyquinazolin-4-amine (**III-5a**)

Yield: 85%. Brown viscous oil. ^1^H NMR (500 MHz, DMSO-*d*_6_): δ 8.17 (s, 2H), 7.66 (d, *J* = 2.8 Hz, 1H), 7.56 (d, *J* = 9.0 Hz, 1H), 7.44 (dd, *J* = 9.1, 2.7 Hz, 1H), 3.87 (s, 3H) ppm; ^13^C NMR (126 MHz, DMSO-*d*_6_): δ 163.47, 157.52, 155.29, 146.42, 128.50, 125.17, 114.03, 103.88, 56.34 ppm.

2-chloro-6-methoxy-*N*-methylquinazolin-4-amine (**III-5b**)

Yield: 88%. Brown viscous oil. ^1^H NMR (500 MHz, DMSO-*d*_6_): δ 8.59 (q, *J* = 4.6 Hz, 1H), 7.63 (d, *J* = 2.8 Hz, 1H), 7.55 (d, *J* = 9.0 Hz, 1H), 7.41 (dd, *J* = 9.0, 2.7 Hz, 1H), 3.87 (s, 3H), 3.01 (d, *J* = 4.5 Hz, 3H) ppm; ^13^C NMR (126 MHz, DMSO-*d*_6_): δ 161.43, 157.63, 155.39, 145.52, 128.58, 124.56, 114.66, 103.14, 56.30, 28.36 ppm.

2-chloro-*N*-ethyl-6-methoxyquinazolin-4-amine (**III-5c**)

Yield: 84%. Brown viscous oil. ^1^H NMR (500 MHz, DMSO-*d*_6_): δ 8.55 (t, *J* = 5.5 Hz, 1H), 7.67 (d, *J* = 2.7 Hz, 1H), 7.55 (d, *J* = 9.0 Hz, 1H), 7.41 (dd, *J* = 9.1, 2.7 Hz, 1H), 3.88 (s, 3H), 3.59–3.50 (m, 2H), 1.25 (t, *J* = 7.2 Hz, 3H) ppm; ^13^C NMR (126 MHz, DMSO-*d*_6_): δ 160.76, 157.62, 155.38, 145.74, 128.60, 124.63, 114.57, 103.23, 56.38, 36.19, 14.54 ppm.

2-chloro-6-methoxy-*N*-(propan-2-yl)quinazolin-4-amine (**III-5d**)

Yield: 90%. Brown viscous oil. ^1^H NMR (500 MHz, DMSO-*d*_6_): δ 8.22 (bs, *J* = 6.0 Hz, 1H), 7.73 (t, *J* = 3.3 Hz, 1H), 7.54 (dd, *J* = 9.1, 3.5 Hz, 1H), 7.46–7.35 (m, 1H), 4.45 (m, *J* = 6.9 Hz, 1H), 3.89 (d, *J* = 3.3 Hz, 3H), 1.28 (dd, *J* = 6.4, 3.1 Hz, 6H) ppm; ^13^C NMR (126 MHz, DMSO-*d*_6_): δ 160.06, 157.62, 155.35, 145.90, 128.59, 124.64, 114.50, 103.41, 56.51, 43.03, 22.37 ppm.

*N*-butyl-2-chloro-6-methoxyquinazolin-4-amine (**III-5e**)

Yield: 84%. Brown viscous oil. ^1^H NMR (500 MHz, DMSO-*d*_6_): δ 8.51 (t, *J* = 5.5 Hz, 1H), 7.68 (d, *J* = 2.8 Hz, 1H), 7.55 (d, *J* = 9.0 Hz, 1H), 7.41 (dd, *J* = 9.1, 2.7 Hz, 1H), 3.88 (s, 3H), 3.54–3.46 (m, 2H), 1.68–1.58 (m, 2H), 1.42–1.27 (m, 2H), 0.94 (t, *J* = 7.4 Hz, 3H) ppm; ^13^C NMR (126 MHz, DMSO-*d*_6_): δ 160.93, 157.63, 155.37, 145.76, 128.59, 124.59, 114.56, 103.26, 56.40, 40.99, 30.96, 20.18, 14.21 ppm.

2-chloro-6-methoxy-*N*-(2-methoxyethyl)quinazolin-4-amine (**III-5f**)

Yield: 93%. Brown viscous oil. Product (**III-5f**) was used directly for the next step of synthesis.

2-chloro-*N*-cyclopropyl-6-methoxyquinazolin-4-amine (**III-5g**)

Yield: 87%. Brown viscous oil. ^1^H NMR (500 MHz, DMSO-*d*_6_): δ 8.51 (d, *J* = 3.7 Hz, 1H), 7.65 (d, *J* = 2.7 Hz, 1H), 7.56 (d, *J* = 9.1 Hz, 1H), 7.41 (dd, *J* = 9.1, 2.7 Hz, 1H), 3.87 (s, 3H), 3.05–2.97 (m, 1H), 0.90–0.80 (m, 2H), 0.73–0.62 (m, 2H) ppm; ^13^C NMR (126 MHz, DMSO-*d*_6_): δ 162.24, 157.64, 155.35, 145.68, 128.60, 124.78, 114.49, 103.21, 56.42, 24.86, 6.65 ppm.

2-chloro-*N*-cyclohexyl-6-methoxyquinazolin-4-amine (**III-5h**)

Yield: 91%. Brown viscous oil. ^1^H NMR (500 MHz, DMSO-*d*_6_): δ 8.17 (d, *J* = 7.8 Hz, 1H), 7.73 (d, *J* = 2.7 Hz, 1H), 7.54 (d, *J* = 9.0 Hz, 1H), 7.41 (dd, *J* = 9.1, 2.7 Hz, 1H), 3.89 (s, 3H), 1.99–1.92 (m, 2H), 1.83–1.75 (m, 2H), 1.71–1.64 (m, 1H), 1.46–1.30 (m, 4H), 1.23–1.13 (m, 1H) ppm; ^13^C NMR (126 MHz, DMSO-*d*_6_): δ 160.03, 157.62, 155.36, 145.95, 128.59, 124.66, 114.50, 103.44, 56.52, 50.26, 49.07, 32.47, 25.78, 25.48 ppm.

#### 3.1.2. General Procedure for the Preparation of Compounds **I-III/6a-6h**

The intermediate compound **I-III**/**5a-5h** (1.0 eq.) was charged into a 50 mL flask and dissolved in acetonitrile (10 mL). *N*-methylpropargylamine (6 eq.) was added to the reaction mixture. The reaction mixture was heated at 130 °C for 1 h–4 days. After completion of the reaction, the mixture was cooled to room temperature. The solvent was evaporated in vacuo and the crude intermediate **I-III/6a-h** was purified by flash chromatography using DCM/MeOH/25% aq.NH_3_ (20:1:0.1) as mobile phase. Substituted derivatives **I-III**/**6a-h** were obtained as free bases in yields (21–87%). Compounds **I-III**/**6a-6h** were obtained by this procedure, the characterization of which is reported below.

*N2*-methyl-*N2*-(prop-2-yn-1-yl)quinazoline-2,4-diamine (**I-6a**)

Yield: 58%. Brown crystalline powder; melting point: 167 °C (decomposed). ^1^H NMR (500 MHz, Methanol-*d*_4_): *δ* 8.13 (dd, *J* = 8.2, 1.4 Hz, 1H), 7.99 (dd, *J* = 8.6, 1.0 Hz, 1H), 7.83 (ddd, *J* = 8.7, 7.3, 1.5 Hz, 1H), 7.42 (ddd, *J* = 8.2, 7.2, 1.0 Hz, 1H), 5.58 (dt, *J* = 4.1, 2.9 Hz, 1H), 5.04 (dt, *J* = 4.2, 2.5 Hz, 1H), 4.49 (t, *J* = 2.7 Hz, 2H), 3.12 (s, 3H) ppm; ^13^C NMR (126 MHz, Methanol-*d*_4_): *δ* 164.16, 155.96, 137.65, 137.30, 136.12, 125.85, 124.97, 115.07, 110.54, 94.59, 52.77, 29.87 ppm; HRMS (ESI^+^): [M+H]^+^: calculated for C_12_H_13_N_4_^+^ (*m*/*z*): 213.1135; found 213.1133. LC-UV purity 98%.

*N2*,*N4*-dimethyl-*N2*-(prop-2-yn-1-yl)quinazoline-2,4-diamine (**I-6b**)

Yield: 33%. White crystalline powder; melting point: 163 °C (decomposed). ^1^H NMR (500 MHz, DMSO-*d*_6_): *δ* 8.54 (dd, *J* = 8.1, 1.5 Hz, 1H), 8.07 (dd, *J* = 8.6, 1.0 Hz, 1H), 7.88 (ddd, *J* = 8.6, 7.3, 1.4 Hz, 1H), 7.56–7.47 (m, 1H), 5.68–5.64 (m, 1H), 5.13–5.08 (m, 1H), 4.59 (t, *J* = 2.7 Hz, 2H), 3.17 (s, 3H), 3.14 (s, 3H) ppm; ^13^C NMR (126 MHz, DMSO-*d*_6_): *δ* 160.65, 155.58, 137.69, 136.68, 136.19, 126.14, 125.35, 115.61, 111.49, 95.52, 53.33, 31.27, 29.00 ppm; HRMS (ESI^+^): [M+H]^+^: calculated for C_13_H_15_N_4_^+^ (*m*/*z*): 227.1292; found 227.1288. LC-UV purity ˃ 99%.

*N4*-ethyl-*N2*-methyl-*N2*-(prop-2-yn-1-yl)quinazoline-2,4-diamine (**I-6c**)

Yield: 55%. Brown crystalline powder; melting point: 157 °C (decomposed). ^1^H NMR (500 MHz, DMSO-*d*_6_): *δ* 8.64 (dd, *J* = 8.2, 1.4 Hz, 1H), 8.08 (dd, *J* = 8.6, 1.0 Hz, 1H), 7.92–7.85 (m, 1H), 7.56–7.49 (m, 1H), 5.67 (q, *J* = 3.0 Hz, 1H), 5.13–5.10 (m, 1H), 4.59 (t, *J* = 2.7 Hz, 2H), 3.70 (q, *J* = 7.2 Hz, 2H), 3.16 (s, 3H), 1.29 (t, *J* = 7.2 Hz, 3H) ppm; ^13^C NMR (126 MHz, DMSO-*d*_6_): *δ* 160.08, 155.63, 137.72, 136.84, 136.22, 126.38, 125.27, 115.60, 111.50, 95.48, 53.33, 37.12, 31.25, 14.10 ppm; HRMS (ESI^+^): [M+H]^+^: calculated for C_14_H_17_N_4_^+^ (*m*/*z*): 241.1448; found 241.1444. LC-UV purity 99%.

*N2*-methyl-*N2*-(prop-2-yn-1-yl)-*N4*-(propan-2-yl)quinazoline-2,4-diamine (**I-6d**)

Yield: 85%. Brown crystalline powder; melting point: 183 °C (decomposed). ^1^H NMR (500 MHz, DMSO-*d*_6_): *δ* 8.77 (dd, *J* = 8.2, 1.4 Hz, 1H), 8.08 (dd, *J* = 8.6, 1.0 Hz, 1H), 7.89 (ddd, *J* = 8.5, 7.2, 1.4 Hz, 1H), 7.52 (ddd, *J* = 8.2, 7.2, 1.0 Hz, 1H), 5.67 (dt, *J* = 4.1, 2.9 Hz, 1H), 5.12 (dt, *J* = 4.0, 2.5 Hz, 1H), 4.68 (h, *J* = 6.7 Hz, 1H), 4.58 (t, *J* = 2.7 Hz, 2H), 3.15 (s, 3H), 1.36 (d, *J* = 6.6 Hz, 6H) ppm; ^13^C NMR (126 MHz, DMSO-*d*_6_): *δ* 159.41, 155.62, 137.72, 136.92, 136.23, 126.69, 125.14, 115.54, 111.43, 95.50, 53.34, 44.72, 31.26, 21.78 ppm; HRMS (ESI^+^): [M+H]^+^: calculated for C_15_H_19_N_4_^+^ (*m*/*z*): 255.1605; found 255.1600. LC-UV purity 94%.

*N4*-butyl-*N2*-methyl-*N2*-(prop-2-yn-1-yl)quinazoline-2,4-diamine (**I-6e**)

Yield: 71%. Brown crystalline powder; melting point: 135 °C (decomposed). ^1^H NMR (500 MHz, Methanol-*d*_4_): *δ* 8.15 (dd, *J* = 8.2, 1.4 Hz, 1H), 7.99 (dd, *J* = 8.6, 1.0 Hz, 1H), 7.81 (ddd, *J* = 8.6, 7.3, 1.4 Hz, 1H), 7.42 (ddd, *J* = 8.2, 7.2, 1.0 Hz, 1H), 5.57 (dt, *J* = 4.1, 2.9 Hz, 1H), 5.04 (dt, *J* = 4.1, 2.5 Hz, 1H), 4.51 (t, *J* = 2.7 Hz, 2H), 3.67 (t, *J* = 7.3 Hz, 2H), 3.25 (s, 2H), 3.15 (s, 3H), 1.72–1.63 (m, 2H), 1.37 (h, *J* = 7.4 Hz, 2H), 0.91 (t, *J* = 7.4 Hz, 3H) ppm; ^13^C NMR (126 MHz, Methanol-*d*_4_): *δ* 160.75, 155.74, 137.61, 136.86, 135.50, 124.99, 124.81, 115.15, 111.22, 94.53, 52.82, 41.68, 30.29, 29.90, 19.80, 12.71 ppm; HRMS (ESI^+^): [M+H]^+^: calculated for C_16_H_21_N_4_^+^ (*m*/*z*): 269.1761; found 269.1755. LC-UV purity 99%.

*N4*-(2-methoxyethyl)-*N2*-methyl-*N2*-(prop-2-yn-1-yl)quinazoline-2,4-diamine (**I-6f**)

Yield: 57%. Brown crystalline powder; melting point: 154 °C (decomposed). ^1^H NMR (500 MHz, DMSO-*d*_6_): *δ* 8.65 (dd, *J* = 8.2, 1.4 Hz, 1H), 8.09 (dd, *J* = 8.6, 1.0 Hz, 1H), 7.90 (ddd, *J* = 8.6, 7.2, 1.4 Hz, 1H), 7.53 (ddd, *J* = 8.2, 7.2, 1.0 Hz, 1H), 5.68 (dt, *J* = 4.0, 2.9 Hz, 1H), 5.12 (dt, *J* = 4.1, 2.5 Hz, 1H), 4.59 (t, *J* = 2.7 Hz, 2H), 3.85 (q, *J* = 4.1, 2.4 Hz, 2H), 3.68 (t, *J* = 5.6 Hz, 2H), 3.31 (s, 3H), 3.16 (s, 3H) ppm; ^13^C NMR (126 MHz, DMSO-*d*_6_): *δ* 160.69, 155.58, 137.71, 136.89, 136.37, 126.40, 125.34, 115.64, 111.41, 95.63, 69.61, 58.44, 53.37, 41.69, 31.27 ppm; HRMS (ESI^+^): [M+H]^+^: calculated for C_15_H_19_N_4_O^+^ (*m*/*z*): 271.1481; found 271.1551. LC-UV purity 95%.

*N4*-cyclopropyl-*N2*-methyl-*N2*-(prop-2-yn-1-yl)quinazoline-2,4-diamine (**I-6g**)

Yield: 69%. Yellow crystalline powder; melting point: 189 °C (decomposed). ^1^H NMR (500 MHz, Chloroform-*d*): *δ* 7.44–7.42 (m, 2H), 7.37–7.34 (m, 1H), 6.99–6.95 (m, 1H), 4.58 (d, *J* = 2.4 Hz, 2H), 3.24 (s, 3H), 2.92–2.87 (m, 1H), 2.09 (t, *J* = 2.4 Hz, 1H), 0.85–0.79 (m, 2H), 0.59–0.54 (m, 2H) ppm; ^13^C NMR (126 MHz, Chloroform-*d*): *δ* 160.92, 159.01, 152.20, 132.41, 126.21, 121.00, 120.53, 113.36, 110.33, 80.88, 70.56, 52.78, 38.30, 34.13, 24.13, 7.19 ppm; HRMS (ESI^+^): [M+H]^+^: calculated for C_15_H_17_N_4_^+^ (*m*/*z*): 253.1448; found 253.1444. LC-UV purity 99%.

*N4*-cyclohexyl-*N2*-methyl-*N2*-(prop-2-yn-1-yl)quinazoline-2,4-diamine (**I-6h**)

Yield: 72%. White crystalline powder; melting point: 174 °C (decomposed). ^1^H NMR (500 MHz, DMSO-*d*_6_): *δ* 8.09 (dd, *J* = 8.2, 1.4 Hz, 1H), 7.62 (d, *J* = 7.4 Hz, 1H), 7.50 (ddd, *J* = 8.4, 6.9, 1.4 Hz, 1H), 7.29 (dd, *J* = 8.4, 1.2 Hz, 1H), 7.07 (ddd, *J* = 8.2, 6.9, 1.3 Hz, 1H), 4.48 (d, *J* = 2.4 Hz, 2H), 4.10–4.02 (m, 1H), 3.13 (s, 3H), 3.05 (t, *J* = 2.4 Hz, 1H), 2.06–1.99 (m, 2H), 1.84–1.75 (m, 2H), 1.69–1.62 (m, 1H), 1.40–1.33 (m, 4H), 1.25–1.14 (m, 1H) ppm; ^13^C NMR (126 MHz, DMSO-*d*_6_): *δ* 159.44, 159.07, 152.20, 132.70, 125.55, 123.45, 120.84, 111.00, 81.54, 73.67, 50.30, 38.13, 34.34, 32.45, 25.95, 25.61 ppm; HRMS (ESI^+^): [M+H]^+^: calculated for C_18_H_23_N_4_^+^ (*m*/*z*): 295.1918; found 295.1913. LC-UV purity 98%.

7-chloro-*N2*-methyl-*N2*-(prop-2-yn-1-yl)quinazoline-2,4-diamine (**II-6a**)

Yield: 29%. yellow crystalline powder; melting point: 169 °C (decomposed). ^1^H NMR (500 MHz, Methanol-*d*_4_): *δ* 8.04 (d, *J* = 8.6 Hz, 1H), 7.76 (d, *J* = 1.9 Hz, 1H), 7.29 (dd, *J* = 8.6, 1.9 Hz, 1H), 5.36–5.32 (m, 1H), 4.93–4.90 (m, 1H), 4.36 (t, *J* = 2.6 Hz, 2H), 3.03 (s, 3H) ppm; ^13^C NMR (126 MHz, Methanol-*d*_4_): *δ* 162.62, 154.68, 140.28, 137.70, 137.48, 127.90, 124.61, 114.25, 112.34, 92.82, 52.49, 29.77 ppm; HRMS (ESI^+^): [M+H]^+^: calculated for C_12_H_12_ClN_4_^+^ (*m*/*z*): 247.0745; found 247.0742. LC-UV purity 96%.

7-chloro-*N2*,*N4*-dimethyl-*N2*-(prop-2-yn-1-yl)quinazoline-2,4-diamine (**II-6b**)

Yield: 55%. Brown crystalline powder; melting point: 156 °C (decomposed). ^1^H NMR (500 MHz, Methanol-*d*_4_): *δ* 8.18 (d, *J* = 8.7 Hz, 1H), 8.05 (d, *J* = 1.9 Hz, 1H), 7.55 (dd, *J* = 8.7, 1.9 Hz, 1H), 5.66–5.62 (m, 1H), 5.19 (dt, *J* = 4.4, 2.5 Hz, 1H), 4.63 (t, *J* = 2.7 Hz, 2H), 3.28 (s, 3H), 3.27 (s, 3H), 3.26 (s, 1H) ppm; ^13^C NMR (126 MHz, Methanol-*d*_4_): *δ* 141.51, 137.26, 126.45, 125.23, 118.47, 116.64, 114.85, 110.17, 94.91, 52.84, 29.99, 27.97, 12.44 ppm; HRMS (ESI^+^): [M+H]^+^: calculated for C_13_H_14_ClN_4_^+^ (*m*/*z*): 261.0902; found 261.0898. LC-UV purity 98%.

7-chloro-*N4*-ethyl-*N2*-methyl-*N2*-(prop-2-yn-1-yl)quinazoline-2,4-diamine (**II-6c**)

Yield: 83%. Brown crystalline powder; melting point: 189 °C (decomposed). ^1^H NMR (500 MHz, Methanol-*d*_4_): *δ* 8.13 (d, *J* = 8.7 Hz, 1H), 7.93 (d, *J* = 1.8 Hz, 1H), 7.43 (dd, *J* = 8.7, 1.9 Hz, 1H), 5.52 (dt, *J* = 4.3, 2.9 Hz, 1H), 5.07 (dt, *J* = 4.4, 2.5 Hz, 1H), 4.51 (t, *J* = 2.7 Hz, 2H), 3.70 (q, *J* = 7.2 Hz, 2H), 3.15 (s, 3H), 1.27 (t, *J* = 7.3 Hz, 3H) ppm; ^13^C NMR (126 MHz, Methanol-*d*_4_): *δ* 160.06, 155.72, 141.61, 137.70, 137.25, 126.56, 125.22, 114.85, 110.05, 94.96, 52.84, 37.07, 29.97, 12.49 ppm; HRMS (ESI^+^): [M+H]^+^: calculated for C_14_H_16_ClN_4_O^+^ (*m*/*z*): 275.1058; found 275.1054. LC-UV purity 95%.

7-chloro-*N2*-methyl-*N2*-(prop-2-yn-1-yl)-*N4*-(propan-2-yl)quinazoline-2,4-diamine (**II-6d**)

Yield: 44%. Yellow crystalline powder; melting point: ˃205 °C (decomposed). ^1^H NMR (500 MHz, Methanol-*d*_4_): *δ* 8.23 (d, *J* = 8.7 Hz, 1H), 7.92 (d, *J* = 2.0 Hz, 1H), 7.43 (dd, *J* = 8.7, 1.9 Hz, 1H), 5.52 (dt, *J* = 4.3, 2.9 Hz, 1H), 5.07 (dt, *J* = 4.3, 2.5 Hz, 1H), 4.65 (hept, *J* = 6.6 Hz, 1H), 4.51 (t, *J* = 2.7 Hz, 2H), 3.15 (s, 4H), 1.30 (d, *J* = 6.6 Hz, 6H) ppm; ^13^C NMR (126 MHz, Methanol-*d*_4_): *δ* 159.31, 155.67, 141.58, 137.78, 137.25, 126.74, 125.14, 114.79, 110.07, 94.93, 52.83, 44.78, 29.96, 20.28 ppm; HRMS (ESI^+^): [M+H]^+^: calculated for C_15_H_18_ClN_4_^+^ (*m*/*z*): 289.1215; found 289.1211. LC-UV purity 96%.

*N4*-butyl-7-chloro-*N2*-methyl-*N2*-(prop-2-yn-1-yl)quinazoline-2,4-diamine (**II-6e**)

Yield: 71%. White crystalline powder; melting point: 213 °C (decomposed). ^1^H NMR (500 MHz, Methanol-*d*_4_): *δ* 8.32 (d, *J* = 8.8 Hz, 1H), 8.24 (d, *J* = 2.0 Hz, 1H), 7.67 (dd, *J* = 8.8, 1.9 Hz, 1H), 7.25 (q, *J* = 1.3 Hz, 1H), 3.70 (s, 3H), 3.65 (d, *J* = 7.2 Hz, 2H), 2.74 (d, *J* = 1.3 Hz, 3H), 1.73–1.65 (m, 2H), 1.39 (h, *J* = 7.4 Hz, 2H), 0.92 (t, *J* = 7.4 Hz, 3H) ppm; ^13^C NMR (126 MHz, Methanol-*d*_4_): *δ* 156.57, 144.57, 140.26, 136.66, 127.32, 126.45, 122.38, 118.46, 116.63, 111.54, 41.39, 30.84, 30.27, 19.87, 12.76, 12.45 ppm; HRMS (ESI^+^): [M+H]^+^: calculated for C_16_H_20_ClN_4_^+^ (*m*/*z*): 303.1371; found 303.1367. LC-UV purity 98%.

7-chloro-*N4*-(2-methoxyethyl)-*N2*-methyl-*N2*-(prop-2-yn-1-yl)quinazoline-2,4-diamine (**II-6f**)

Yield: 65%. Brown crystalline powder; melting point: 187 °C (decomposed). ^1^H NMR (500 MHz, Methanol-*d*_4_): *δ* 8.11 (d, *J* = 8.7 Hz, 1H), 7.85 (d, *J* = 1.9 Hz, 1H), 7.37 (dd, *J* = 8.7, 1.9 Hz, 1H), 5.45 (dt, *J* = 4.2, 2.8 Hz, 1H), 5.01 (dt, *J* = 4.2, 2.4 Hz, 1H), 4.46 (t, *J* = 2.7 Hz, 2H), 3.82 (t, *J* = 5.6 Hz, 2H), 3.61 (t, *J* = 5.6 Hz, 2H), 3.30 (s, 3H), 3.11 (s, 3H) ppm; ^13^C NMR (126 MHz, Methanol-*d*_4_): *δ* 159.61, 155.17, 140.85, 137.32, 126.86, 124.95, 114.57, 111.34, 94.12, 69.99, 57.63, 52.71, 42.68, 29.92, 28.42 ppm; HRMS (ESI^+^): [M+H]^+^: calculated for C_15_H_18_ClN_4_O^+^ (*m*/*z*): 305.1164; found 305.1160. LC-UV purity 95%.

7-chloro-*N4*-cyclopropyl-*N2*-methyl-*N2*-(prop-2-yn-1-yl)quinazoline-2,4-diamine (**II-6g**)

Yield: 69%. Yellow crystalline powder; melting point: 195 °C (decomposed). 1H NMR (500 MHz, Methanol-*d*_4_) δ 7.97 (d, *J* = 8.6 Hz, 1H), 7.73 (d, *J* = 2.0 Hz, 1H), 7.22 (dd, *J* = 8.7, 2.0 Hz, 1H), 5.31–5.27 (m, 1H), 4.91–4.85 (m, 1H), 4.37–4.33 (m, 2H), 3.58–3.50 (m, 1H), 3.07 (s, 2H), 0.81–0.75 (m, 2H), 0.70–0.64 (m, 2H) ppm; ^13^C NMR (126 MHz, Methanol-*d*_4_): δ 154.19, 137.61, 137.13, 127.07, 124.26, 113.96, 91.90, 82.93, 70.13, 52.42, 29.75, 27.68, 6.02, 5.47 ppm; HRMS (ESI^+^): [M+H]^+^: calculated for C_15_H_16_ClN_4_^+^ (*m*/*z*): 287.1058; found 287.1054. LC-UV purity ˃ 99%.

7-chloro-*N4*-cyclohexyl-*N2*-methyl-*N2*-(prop-2-yn-1-yl)quinazoline-2,4-diamine (**II-6h**)

Yield: 68%. Yellow crystalline powder; melting point: 191 °C (decomposed). ^1^H NMR (500 MHz, Methanol-*d*_4_): *δ* 8.23 (d, *J* = 8.8 Hz, 1H), 7.92 (d, *J* = 1.9 Hz, 1H), 7.43 (dd, *J* = 8.7, 1.9 Hz, 1H), 5.51 (dt, *J* = 4.3, 2.9 Hz, 1H), 5.07 (dt, *J* = 4.3, 2.5 Hz, 1H), 4.51 (t, *J* = 2.7 Hz, 2H), 4.33–4.24 (m, 1H), 3.14 (s, 3H), 2.00–1.93 (m, 2H), 1.82–1.76 (m, 2H), 1.68–1.62 (m, 1H), 1.49–1.32 (m, 4H), 1.25–1.14 (m, 1H) ppm; ^13^C NMR (126 MHz, Methanol-*d*_4_): *δ* 159.33, 155.68, 141.60, 137.79, 137.23, 126.75, 125.14, 114.78, 110.05, 94.94, 52.83, 52.14, 31.33, 29.96, 25.05, 24.92 ppm; HRMS (ESI^+^): [M+H]^+^: calculated for C_18_H_22_ClN_4_^+^ (*m*/*z*): 329.1528; found 329.1523. LC-UV purity 99%.

6-methoxy-*N2*-methyl-*N2*-(prop-2-yn-1-yl)quinazoline-2,4-diamine (**III-6a**)

Yield: 87%. Dark brown crystalline powder; melting point: ˃184 °C (decomposed). ^1^H NMR (500 MHz, Methanol-*d*_4_): *δ* 8.26 (d, *J* = 9.4 Hz, 1H), 7.76 (d, *J* = 2.8 Hz, 1H), 7.52 (dd, *J* = 9.4, 2.8 Hz, 1H), 7.20 (d, *J* = 1.3 Hz, 1H), 4.47 (t, *J* = 2.7 Hz, 1H), 3.89 (s, 3H), 3.84 (s, 1H), 3.68 (s, 3H) ppm; ^13^C NMR (126 MHz, Methanol-*d*_4_): *δ* 158.35, 156.91, 123.53, 121.99, 118.17, 117.92, 106.98, 55.37, 55.27, 52.73, 30.73, 29.84, 12.58 ppm; HRMS (ESI^+^): [M+H]^+^: calculated for C_13_H_15_N_4_O^+^ (*m*/*z*): 243.1241; found 243.1238. LC-UV purity 96%.

6-methoxy-*N2*,*N4*-dimethyl-*N2*-(prop-2-yn-1-yl)quinazoline-2,4-diamine (**III-6b**)

Yield: 61%. Brown crystalline powder; melting point: 178 °C (decomposed). ^1^H NMR (500 MHz, Methanol-*d*_4_): *δ* 8.23 (d, *J* = 9.4 Hz, 1H), 7.70 (d, *J* = 2.8 Hz, 1H), 7.48 (dd, *J* = 9.4, 2.8 Hz, 1H), 7.21 (q, *J* = 1.3 Hz, 1H), 3.89 (s, 3H), 3.71 (s, 3H), 3.15 (s, 3H), 2.72 (d, *J* = 1.3 Hz, 3H) ppm; ^13^C NMR (126 MHz, Methanol-*d*_4_): *δ* 158.39, 157.13, 143.53, 129.77, 122.76, 121.92, 118.24, 114.00, 106.28, 93.78, 55.38, 30.76, 27.52, 12.59 ppm; HRMS (ESI^+^): [M+H]^+^: calculated for C_14_H_17_N_4_O^+^ (*m*/*z*): 257.1397; found 257.1393. LC-UV purity 96%.

*N4*-ethyl-6-methoxy-*N2*-methyl-*N2*-(prop-2-yn-1-yl)quinazoline-2,4-diamine (**III-6c**)

Yield: 48%. Yellow crystalline powder; melting point: 198 °C (decomposed). ^1^H NMR (500 MHz, Methanol-*d*_4_): *δ* 8.34 (d, *J* = 9.4 Hz, 1H), 7.88 (d, *J* = 2.8 Hz, 1H), 7.60 (dd, *J* = 9.4, 2.8 Hz, 1H), 7.32 (q, *J* = 1.3 Hz, 1H), 4.02 (s, 3H), 3.84–3.79 (m, 5H), 2.83 (d, *J* = 1.3 Hz, 3H), 1.43 (t, *J* = 7.2 Hz, 3H) ppm; ^13^C NMR (126 MHz, Methanol-*d*_4_): *δ* 158.39, 156.43, 143.48, 129.90, 122.78, 121.91, 118.21, 117.89, 114.00, 106.38, 55.42, 36.56, 30.72, 12.68, 12.61 ppm; HRMS (ESI^+^): [M+H]^+^: calculated for C_15_H_19_N_4_O^+^ (*m*/*z*): 271.1554; found 271.1548. LC-UV purity 96%.

6-methoxy-*N2*-methyl-*N2*-(prop-2-yn-1-yl)-*N4*-(propan-2-yl)quinazoline-2,4-diamine (**III-6d**)

Yield: 31%. Brown crystalline powder; melting point: 135 °C (decomposed). ^1^H NMR (500 MHz, Methanol-*d*_4_): *δ* 7.94 (d, *J* = 9.3 Hz, 1H), 7.77 (d, *J* = 2.8 Hz, 1H), 7.41 (dd, *J* = 9.3, 2.8 Hz, 1H), 5.52 (dt, *J* = 4.1, 2.9 Hz, 1H), 4.99 (dt, *J* = 4.1, 2.6 Hz, 1H), 3.85 (s, 3H), 3.54–3.48 (m, 1H), 3.13 (s, 3H), 2.06 (s, 6H) ppm; ^13^C NMR (126 MHz, Methanol-*d*_4_): *δ* 174.98, 156.98, 137.87, 131.00, 123.32, 116.65, 112.24, 107.42, 93.75, 55.28, 52.77, 44.65, 29.85, 29.28, 20.66, 20.32 ppm; HRMS (ESI^+^): [M+H]^+^: calculated for C_16_H_21_N_4_O^+^ (*m*/*z*): 285.1710; found 285.1706. LC-UV purity 95%.

*N4*-butyl-6-methoxy-*N2*-methyl-*N2*-(prop-2-yn-1-yl)quinazoline-2,4-diamine (**III-6e**)

Yield: 83%. Brown crystalline powder; melting point: 187 °C (decomposed). ^1^H NMR (500 MHz, Methanol-*d*_4_): *δ* 8.25 (d, *J* = 9.4 Hz, 1H), 7.78 (d, *J* = 2.8 Hz, 1H), 7.50 (dd, *J* = 9.4, 2.8 Hz, 1H), 7.20 (d, *J* = 1.3 Hz, 1H), 3.90 (s, 3H), 3.70 (s, 3H), 3.67 (t, *J* = 7.3 Hz, 2H), 2.72 (d, *J* = 1.3 Hz, 3H), 1.75–1.68 (m, 2H), 1.43–1.38 (m, 2H), 0.93 (t, *J* = 7.4 Hz, 3H) ppm; ^13^C NMR (126 MHz, Methanol-*d*_4_): *δ* 158.43, 156.64, 143.52, 129.98, 122.75, 121.94, 118.28, 117.86, 106.40, 93.74, 55.37, 41.35, 30.70, 30.36, 19.91, 12.78, 12.58 ppm; HRMS (ESI^+^): [M+H]^+^: calculated for C_17_H_23_N_4_O^+^ (*m*/*z*): 299.1867; found 299.1863. LC-UV purity 99%.

6-methoxy-*N4*-(2-methoxyethyl)-*N2*-methyl-*N2*-(prop-2-yn-1-yl)quinazoline-2,4-diamine (**III-6f**)

Yield: 28%. Dark brown crystalline powder; melting point: 195 °C (decomposed). ^1^H NMR (500 MHz, Methanol-*d*_4_): *δ* 8.26 (d, *J* = 9.4 Hz, 1H), 7.80 (d, *J* = 2.8 Hz, 1H), 7.51 (dd, *J* = 9.4, 2.8 Hz, 1H), 7.23–7.18 (m, 1H), 3.90 (s, 3H), 3.86 (t, *J* = 5.5 Hz, 2H), 3.70 (s, 3H), 3.67 (t, *J* = 5.5 Hz, 2H), 3.32 (s, 3H), 2.73 (d, *J* = 1.3 Hz, 3H) ppm; ^13^C NMR (126 MHz, Methanol-*d*_4_): *δ* 174.98, 158.44, 156.87, 143.35, 130.03, 122.95, 122.04, 118.28, 117.98, 114.01, 106.43, 69.70, 57.65, 55.36, 41.28, 30.76, 20.65, 12.58 ppm; HRMS (ESI^+^): [M+H]^+^: calculated for C_16_H_21_N_4_O_2_^+^ (*m*/*z*): 301.1660; found 301.1655. LC-UV purity 96%.

*N4*-cyclopropyl-6-methoxy-*N2*-methyl-*N2*-(prop-2-yn-1-yl)quinazoline-2,4-diamine (**III-6g**)

Yield: 21%. Brown crystalline powder; melting point: 187 °C (decomposed). ^1^H NMR (500 MHz, Methanol-*d*_4_): *δ* 7.58 (d, *J* = 9.2 Hz, 1H), 7.52 (d, *J* = 3.0 Hz, 1H), 7.03 (dd, *J* = 9.1, 3.0 Hz, 1H), 5.15 (q, *J* = 2.8 Hz, 1H), 4.68–4.63 (m, 1H), 4.23 (t, *J* = 2.5 Hz, 2H), 3.74 (s, 3H), 3.69–3.63 (m, 1H), 2.99 (s, 3H), 0.75–0.71 (m, 2H), 0.66–0.62 (m, 2H) ppm; ^13^C NMR (126 MHz, Methanol-*d*_4_): *δ* 157.07, 156.20, 153.69, 138.23, 129.95, 119.04, 118.26, 115.27, 108.49, 89.13, 54.75, 52.29, 29.67, 28.71, 6.22, 5.47 ppm; HRMS (ESI^+^): [M+H]^+^: calculated for C_16_H_19_N_4_O^+^ (*m*/*z*): 283.1554; found 283.1550. LC-UV purity 99%.

*N4*-cyclohexyl-6-methoxy-*N2*-methyl-*N2*-(prop-2-yn-1-yl)quinazoline-2,4-diamine (**III-6h**)

Yield: 66%. Yellow crystalline powder; melting point: 177 °C (decomposed). ^1^H NMR (500 MHz, Methanol-*d*_4_): *δ* 7.92 (d, *J* = 9.3 Hz, 1H), 7.78 (d, *J* = 2.8 Hz, 1H), 7.39 (dd, *J* = 9.3, 2.8 Hz, 1H), 5.51 (dt, *J* = 4.1, 2.9 Hz, 1H), 4.99 (dt, *J* = 4.2, 2.5 Hz, 1H), 4.48 (t, *J* = 2.7 Hz, 2H), 4.34–4.25 (m, 1H), 3.85 (s, 3H), 3.13 (s, 3H), 2.02–1.95 (m, 2H), 1.83–1.77 (m, 2H), 1.69–1.62 (m, 1H), 1.52–1.43 (m, 2H), 1.42–1.32 (m, 2H), 1.24–1.15 (m, 1H) ppm; ^13^C NMR (126 MHz, Methanol-*d*_4_): *δ* 159.55, 156.96, 155.44, 137.84, 130.99, 123.34, 116.62, 112.23, 107.48, 93.75, 56.92, 55.38, 52.77, 52.12, 31.40, 29.87, 25.11, 25.02, 16.98 ppm; HRMS (ESI^+^): [M+H]^+^: calculated for C_19_H_25_N_4_O^+^ (*m*/*z*): 325.2023; found 325.2019. LC-UV purity 97%.

### 3.2. Biological Evaluation

#### 3.2.1. In Vitro Inhibition Studies on AChE and BChE

The *h*AChE/*h*BChE inhibitory activity of the tested derivatives was determined using Ellman’s method [44,45,46] and is expressed as IC_50_, i.e., a concentration that reduces the cholinesterase activity by 50%. The human recombinant BChE and AChE were prepared at the Department of Toxicology and Military Pharmacy (Faculty of Military Health Sciences, Hradec Kralove, Czech Republic). 5,5′-dithiobis(2-nitrobenzoic acid) (Ellman’s reagent, DTNB), phosphate buffer (PB, pH 7.4), acetylthiocholine (ATC) and butyrylthiocholine (BTC), were purchased from Sigma-Aldrich, Prague, Czech Republic. For measuring purposes, polystyrene Nunc 96-well microplates with a flat-bottom shape (ThermoFisher Scientific, Waltham, MA, USA) were utilized. All the assays were carried out in 0.1 M KH_2_PO_4_/K_2_HPO_4_ buffer, pH 7.4. Enzyme solutions were prepared at 2.0 units/mL in 2 mL aliquots. The assay medium consisted of a 0.1 M phosphate buffer (pH 7.4), 0.01 M DTNB, the enzyme, and 0.01 M substrate (ATC/BTC iodide solution). Concentration 1 µM was tested and the % of inhibition was calculated. Tested compounds were pre-incubated for 5 min. The reaction was started by immediate addition of 20 μL of the substrate. The activity was determined by measuring the increase in absorbance at 412 for *h*AChE/*h*BChE at 37 °C at 2 min intervals-using a multi-mode microplate reader Synergy 2 (Winooski, VT, USA). Each concentration was assayed in triplicate. Software GraphPad Prism 5 (San Diego, CA, USA) was used for the statistical data evaluation.

#### 3.2.2. In Vitro Inhibition of MAO-A/B

The *h*MAO-A and *h*MAO-B enzymes were purchased from Merck (Prague, Czech Republic). The reaction mixture contained *h*MAO-A (2.5 µg/mL protein final concentration) or *h*MAO-B (6.25 µg/mL protein final concentration) enzyme and tested compound in a final concentration of 1 and 10 µM in PBS with 20% (*v*/*v*) glycerol (pH 7.4). The mixture was pre-incubated at 37 °C for 15 min and subsequently substrate kynuramine was added to the final concentration of 100 µM. The final volume of reaction mixture was 0.1 mL. The whole reaction mixture was incubated at 37 °C for 30 min. The reaction was stopped by the addition of 20 µL 4M NaOH. The deamination product of kynuramine formed during the enzymatic reaction 4-hydroxyquinoline (4-HQ) was determined by fluorescent measurement (Ex/Em = 365 nm/480 nm).

#### 3.2.3. Kinetic Study of MAO-A/B Inhibition

Compounds with the highest inhibition potential against *h*MAOs were further analyzed regarding their inhibition kinetics parameters (inhibition mechanism). Thus, the amine oxidase activity assay was carried out at various concentrations of substrate kynuramine (ranging from 10 µM to 250 µM) and various concentrations of the tested compound (ranging from 0.1 µM to 25 µM). Inhibition mechanism was determined by non-linear regression and the double reciprocal method by Lineweaver–Burk [27] using GraphPad Prism 8.2 software. The reversible/irreversible inhibitory activity of selected compounds was determined by incubation of the enzyme with a high concentration of tested compounds (at least 4 times higher than IC_50_ if possible) followed by overnight dialysis (three buffer exchanges) of the enzyme-inhibitor complex against PBS buffer at 6 °C. The recovery of enzyme activity was determined using the above-mentioned protocol.

#### 3.2.4. Evaluation of Antagonist Activity at NMDAR

Human embryonic kidney 293 (HEK293) cells were grown in Opti-MEM I medium containing 5% fetal bovine serum (FBS; both from Thermo Fisher Scientific). Whole-cell patch-clamp recordings were performed using an Axopatch 200B amplifier (Molecular Devices, LLC., San Jose, CA, USA) at a holding membrane potential of −60 mV. HEK293 cells were co-transfected with DNA vectors encoding human versions of the GluN1-4a (GluN1) and GluN2B subunits and green fluorescent protein (GFP) in a 1:1:1 ratio using 0.9 μL PolyMag reagent (OZ Biosciences SAS, Marseille, France) in 50 μL Opti-MEM I [56,57]. After 15 min on a magnetic plate, HEK293 cells were trypsinized and resuspended in Opti-MEM I medium containing 1% FBS, 2 mM MgCl_2_ and 3 mM kynurenic acid (to reduce cell death caused by activation of NMDARs). Electrophysiological measurements were performed 24–48 h after completion of transfection at room temperature. The extracellular recording solution (ECS) contained (in mM): 160 NaCl, 2.5 KCl, 10 HEPES, 10 D-glucose, 0.2 EDTA, and 0.7 CaCl_2_ (pH 7.3 with NaOH). Borosilicate glass micropipettes (tip resistance 4–6 MΩ) were prepared using a P-1000 puller (Sutter Instruments, Novato, CA, USA) and filled with an intracellular recording solution containing (in mM): 125 gluconic acid, 15 CsCl, 10 BAPTA, 10 HEPES, 3 MgCl_2_, 0.5 CaCl_2_, and 2 ATP-Mg salts (pH 7.2 with CsOH). A microprocessor-controlled rapid perfusion system with a time constant for solution exchange around the recorded cell of ~20 ms was used. Currents were low-pass filtered at 2 kHz using an eight-pole Bessel filter, digitized at 5 kHz, and recorded using the pCLAMP 10 program (Molecular Devices, LLC., San Jose, CA, USA). The co-agonist glycine (100 µM) was applied throughout the recording, and the agonist L-glutamate (1 mM) was used to elicit responses of GluN1/GluN2B receptors. The derivatives were dissolved in dimethyl sulfoxide (DMSO) at a concentration of 10 mM; their inhibitory activity was tested at 10 µM and analyzed under steady-state conditions using Clampfit 10.7 (Molecular Devices, LLC., San Jose, CA, USA).

#### 3.2.5. Prediction of BBB Permeability

PAMPA (the parallel artificial membrane-permeability assay) is a high-throughput screening tool applicable for prediction of the passive transport of potential drugs across the blood–brain barrier (BBB) [49,50]. In this study, it was used as a non-cell-based in vitro assay carried out in a coated 96-well membrane filter. The filter membrane of the donor plate was coated with PBL (Polar Brain Lipid, Avanti, USA) in dodecane (4 µL of 20 mg/mL PBL in dodecane) and the acceptor well was filled with 300 µL of PBS (pH 7.4; *V_A_*). The tested compounds were dissolved first in DMSO and subsequently diluted with PBS (pH 7.4) to final concentrations of 50–100 μM in the donor wells. The concentration of DMSO did not exceed 0.5% (*v*/*v*) in the donor solution. The final concentration of DMSO did not exceed 0.5% (*v*/*v*) in the donor solution. About 300 µL of the donor solution was added to the donor wells (*V_D_*) and the donor filter plate was carefully put on the acceptor plate so that the coated membrane was “in touch” with both the donor solution and acceptor buffer. In principle, the test compound diffused from the donor well through the lipid membrane (*Area* = 0.28 cm^2^) to the acceptor well. The concentration of the drug in both the donor and acceptor wells were assessed after 3, 4, 5, and 6 h of incubation in quadruplicate using the UV plate reader Spark (Tecan Group Ltd., Männedorf, Switzerland) at the maximum absorption wavelength of each compound. In addition, the solution of the theoretical compound concentration, simulating the equilibrium state established if the membrane were ideally permeable, was prepared and assessed as well. The concentrations of the compounds in the donor and acceptor well and equilibrium concentration were calculated from the standard curve and expressed as the permeability (*Pe*) according to the equation:

$$Pe=C\times -ln\left(1-\frac{{\left[drug\right]}_{acceptor}}{{\left[drug\right]}_{equilibrium}}\right),$$

where

$$C=\left(\frac{V_{D}\times V_{A}}{\left(V_{D}+V_{A}\right)\times Area\times Time}\right).$$

#### 3.2.6. In Vitro Neurotoxicity

The cytotoxicity of the tested compounds was assessed on the SH-SY5Y cells in both undifferentiated and differentiated form (ECACC, 94030304) using the MTT ((3-(4,5-dimethylthiazol-2-yl)-2,5-diphenyl-tetraziolium bromide, Sigma-Aldrich, St. Louis, MO, USA) [54,58]. The cells were cultured according to ECACC-recommended conditions and seeded into 96-well plates in 100 µL and density of 8000 per well. For evaluation of the cytotoxicity of the differentiated form, the SH-SY5Y cells also underwent a 9-day differentiation protocol into mature neurons [59]. The tested compounds were dissolved in DMSO (Sigma Aldrich) or in phosphate buffer saline (PBS, Sigma Aldrich) and subsequently in the growth medium-high glucose Dulbecco’s Modified Eagle’s medium (DMEM; Sigma-Aldrich, D6429). The final concentration of DMSO did not exceed 1% (*v*/*v*). Then, the differentiated SH-SY5Y cells were treated with concentrations corresponding to the IC_50_ values obtained from the cytotoxicity measurement using undifferentiated SH-SY5Y cells. Cells were exposed to a tested compound for 24 h. Then, the medium was replaced by a medium containing 10 μM of MTT and cells were allowed to produce formazan for another, approximately 3 h under surveillance. Thereafter, the medium was aspirated and purple crystals of MTT formazan were dissolved in 100 μL DMSO under shaking. Cell viability was assessed spectrophotometrically by the amount of formazan produced. Absorbance was measured at 570 nm with 650 nm reference wavelength on Spark (Tecan Group Ltd., Männedorf, Switzerland). The IC_50_ value was then calculated from the control-subtracted triplicates using non-linear regression (four parameters) of GraphPad Prism 9 software. Final IC_50_ and SEM values were obtained as a mean of three.

#### 3.2.7. Assessment of Dehydrogenase Activity and Glutathione Levels

Dehydrogenase activity was evaluated by WST-1 test (Roche, Munich, Germany). The WST-1 test measures the activity of intra- and extra-mitochondrial dehydrogenases. After the treatment with **I-6e, I-6h, II-6e, II-6h** and **III-6a** (10–100 µM), the cells were incubated with the WST-1 reagent according to the manufacturer’s instructions for 1 h. The change of absorbance was measured by spectrophotometer at the wavelength of 440 nm using SPARK microplate reader (Tecan, Austria) while incubated at 37 °C. The dehydrogenase activity was expressed as the percentage of total cellular dehydrogenase activity relative to that in the control cells (control = 100%).

The glutathione (GSH) levels were measured using an optimized monochlorobimane assay [60]. The working solutions of monochlorobimane (MCB, Merck, Rahway, NJ, USA) were prepared fresh at the time of analysis by dilution in Dulbecco’s phosphate buffer (PBS; pH 7; 1 µM, Merck, USA) and tempered at 37 °C for 30 min. After the treatment of cells with **I-6e** (100 µM), **I-6h** (100 µM), **II-6e** (100 µM), **II-6h** (10–100 µM) and **III-6a** (10–100 µM), 20 μL of the MCB solution was added to the cells in 96-well microplates and the measurement started immediately (final concentration of MCB in a well was 40 μmol/L) The fluorescence intensity (Ex/Em = 394/490 nm) was measured kinetically for 20 min using SPARK microplate reader (Tecan, Austria). The fluorescence was expressed as the slope of a fluorescence change over time. The GSH levels were expressed as the percentage relative to the GSH levels in control cells (control = 100%).

#### 3.2.8. Detection of Nuclear Condensation and Fragmentation

To measure nuclear condensation and fragmentation in intact cells, we used fluorescence dye Hoechst 33258 (H33258, 1 mg/mL; Merck, USA) [61]. After cell treatment with **II-6h** (10–100 µM) and **III-6a** (10–100 µM), the cells grown in a 96-well plate were centrifuged (5 min; 8000g; RT). Then, 70 µL of a supernatant was replaced with 70 µL of PBS 1× and 10 µL of H33258 solution was added to a well. The final concentrations of H33258 in a well was 2 µg/mL. Then, the cells were incubated with H33258 for 5 min and spectrofluorometric measurement was performed at Ex/Em = 352/461 nm using SPARK microplate reader (Tecan, Austria) while incubated at 37 °C. The samples were measured at least in triplicates. After background subtraction, the fluorescence signal was presented in RFU as mean ± SEM.

#### 3.2.9. Detection of ATP

The quantification of intracellular ATP was performed using luciferase luminescence assay. After cell treatment with **II-6h** and **III-6a** (10–100 µM), the culture medium was replaced with 100 µL of cold water for cell lysis, incubated for 5 min and centrifuged (4800× *g*; 5 min; 4 °C). To detect intracellular ATP concentration, 90 µL of the reaction solution was added to 10 µL of a lysate. The reaction solution included 20× reaction buffer (500 mM Tricine buffer, pH 7.8; 10 mM MgSO_4_, 2 mM Na_2_EDTA), 5 mg/mL luciferase; 10 mM D-luciferin; 0.1 M DTT and dH_2_O. Luminometric measurement was performed at Em = 550 nm using SPARK microplate reader (Tecan, Grödig, Austria). The intracellular ATP levels were expressed as the percentage of total ATP level relative to that in the control cells (control = 100%) as mean ± SEM.

## 4. Conclusions

The cholinergic hypothesis, postulated 50 years ago [62], has resulted in the development of a few drugs that offer symptomatic relief for AD. Despite significant efforts to understand the disease biology, it remains a challenging task to discover disease-modifying agents to slow down or eventually halt disease onset or progression [63]. A glimmer of hope was ignited in 2019 with the approval of sodium oligomannate (GV-971) in China. GV-971 acts differently from other marketed drugs by suppressing gut dysbiosis, mitigating neuroinflammation, and reversing cognitive impairment [64]. While the recent endorsement of amyloid beta monoclonal antibodies lecanemab and aducanumab also offers potential benefits in the treatment of AD, the effectiveness of these disease-modifying agents is still under clinicians’ debate [5]. Drugs designed by the MTDLs approach offer an intriguing tool to produce various preclinical candidates for treating AD. Nevertheless, the majority of these emerging candidates are endowed with several drawbacks stemming from ab initio lack of knowledge in drug design, as recently outlined [63]. To find treatments for the symptomatic stage of AD, we developed 24 novel *N*-methylpropargylamino-quinazoline derivatives that modulate the cholinergic, glutamatergic, and monoaminergic systems. These compounds were either unsubstituted or substituted at core quinazoline with chlorine at C7 or methoxy group at C6 to mimic template tacrine scaffolds. Before synthesis, we evaluated the physicochemical parameters of the compounds and calculated their oral and CNS availability. All the compounds fitted well to these criteria. Under in vitro conditions, we demonstrated that the compounds interact with MAO-A/B and NMDAR while having a marginal effect on ChEs. We also assessed the neurotoxicity of selected derivatives (**I-6e**, **I-6h**, **II-6e**, **II-6h**, and **III-6a**). Our findings revealed that all these compounds displayed a moderate cytotoxicity profile towards undifferentiated SH-SY5Y cell lines in the micromolar range. Interestingly, in the differentiated model of SH-SY5Y, **II-6h** did not exhibit any cytotoxic effects, thus emphasizing the potential advantage of this compound. Lead candidate **II-6h** revealed significant changes of dehydrogenase activity or glutathione levels when tested at a high concentration (100 μM), while being relatively safe at a lower dose (10 μM). This finding is somewhat surprising, as MAO-B inhibitors reduce the formation of neurotoxic products, such as aldehydes and hydrogen peroxide, which are known to promote the formation of reactive oxygen species contributing ultimately to increased neuronal damage [65]. Our study opens a new avenue for the design of new MTDLs for the therapy of AD.

## Figures and Tables

**Table 1:** In silico prediction of the ADME properties and drug-likeness of derivatives **I-III/6a-6h** with THA and memantine as reference compounds by applying various predictive models.

Compound	Structure	GIA ^a^	BBB ^b^	BBB Score ^c^	Lipinski ^d^	Bio. Score ^e^	cLogS ^f^	PAINS Alert ^g^
**I-6a**	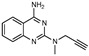	High	Yes	4.58	Yes	0.55	−3.19	0
**I-6b**	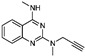	High	Yes	4.85	Yes	0.55	−3.99	0
**I-6c**	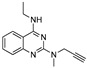	High	Yes	4.32	Yes	0.55	−4.39	0
**I-6d**	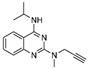	High	Yes	4.78	Yes	0.55	−4.42	0
**I-6e**	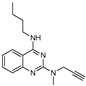	High	Yes	5.01	Yes	0.55	−5.19	0
**I-6f**	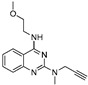	High	Yes	4.09	Yes	0.55	−4.53	0
**I-6g**	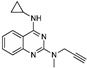	High	Yes	4.96	Yes	0.55	−4.20	0
**I-6h**	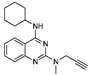	High	Yes	5.05	Yes	0.55	−5.02	0
**II-6a**	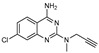	High	Yes	4.64	Yes	0.55	−3.80	0
**II-6b**	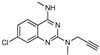	High	Yes	4.88	Yes	0.55	−4.60	0
**II-6c**	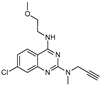	High	Yes	5.03	Yes	0.55	−5.13	0
**II-6d**	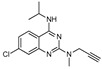	High	Yes	4.67	Yes	0.55	−5.02	0
**II-6e**	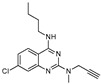	High	Yes	4.98	Yes	0.55	−5.80	0
**II-6f**	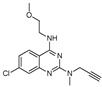	High	Yes	5.03	Yes	0.55	−5.13	0
**II-6g**	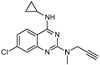	High	Yes	4.95	Yes	0.55	−4.80	0
**II-6h**	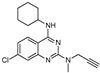	High	Yes	4.99	Yes	0.55	−5.62	0
**III-6a**	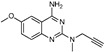	High	Yes	4.26	Yes	0.55	−3.31	0
**III-6b**	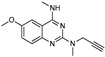	High	Yes	4.51	Yes	0.55	−4.11	0
**III-6c**	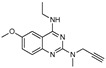	High	Yes	5.11	Yes	0.55	−4.51	0
**III-6d**	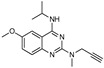	High	Yes	5.11	Yes	0.55	−4.54	0
**III-6e**	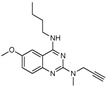	High	Yes	4.65	Yes	0.55	−5.31	0
**III-6f**	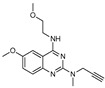	High	Yes	4.82	Yes	0.55	−4.64	0
**III-6g**	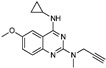	High	Yes	4.60	Yes	0.55	−4.32	0
**III-6**h	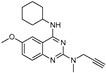	High	Yes	4.69	Yes	0.55	−5.13	0
THA	-	High	Yes	5.38	Yes	0.55	−4.46	0
Memantine	-	High	Yes	4.61	Yes	0.55	−2.80	0

^a^ GIA = Gastrointestinal absorption; ^b^ BBB = Blood-brain barrier permeation; ^c^ ref. [35]; ^d^ ref. [36]; ^e^ Bio. Score = Bioavailability score [37]; ^f^ cLogS (SILICOS-IT) = Water solubility; ^g^ PAINS = Pan-assay interference compounds.

**Table 2:** Inhibition of tested derivatives and reference drugs against both isoforms of human MAO (*h*MAO) enzymes.

Compound	IC_50_ (μM) ± SD		SI for MAO-B ^b^
	*h*MAO-A ^a^	*h*MAO-B ^a^	
**I-6e**	2.60 ± 0.23	– ^c^	– ^c^
**I-6h**	0.90 ± 0.17	8.69 ± 0.12	0.10
**II-6e**	– ^c^	3.65 ± 0.14	– ^c^
**II-6h**	– ^c^	0.33 ± 0.02	– ^c^
**III-6a**	0.62 ± 0.04	-	– ^c^
Clorgyline	0.05 ± 0.00	– ^c^	–
Pargyline	– ^c^	0.08 ± 0.01	–

^a^ Each IC_50_ value is the mean ± SD of at least three independent experiments, ^b^ SI = selectivity index, determined as IC_50_(MAO-A)/IC_50_(MAO-B), ^c^ not determined.

**Table 3:** Relative inhibition (RI, %) of selected and reference compounds against GluN1/GluN2B NMDAR.

Compound (10 µM)	RI, % ± SEM (*n* = 5–6)
**I-6e**	30.10 ± 3.13
**I-6h**	49.34 ± 1.16
**II-6e**	33.80 ± 2.56
**II-6h**	63.48 ± 3.46
**III-6a**	18.07 ± 5.75
Memantine	94.63 ± 1.07
7-MEOTA	59.98 ± 2.04

Human GluN1/GluN2B, −60 mV, 100% = full inhibition.

**Table 4:** Prediction of BBB penetration of studied compounds expressed as *Pe* ± SEM.

Compound	*Pe* ± SEM (×10^−6^ cm s^−1^)	*n*	CNS (+/−)
**I-6e**	7.37 ± 1.22	2	CNS (+)
**I-6h**	5.61 ± 1.01	3	CNS (+)
**II-6e**	0.90 ± 0.43	2	CNS (−)
**II-6h**	9.39 ± 1.17	2	CNS (+)
**III-6a**	0	2	CNS (−)
Tacrine	5.90 ± 0.67	8	CNS (+)
Donepezil	21.9 ± 2.06	6	CNS (+)
Rivastigmine	18.7 ± 2.03	4	CNS (+)
Ibuprofen	18.0 ± 4.32	3	CNS (+)
Chlorothiazide	1.14 ± 0.54	3	CNS (−)
Furosemide	0.19 ± 0.07	3	CNS (−)
Ranitidine	0.04 ± 0.02	2	CNS (−)
Sulfasalazine	0.09 ± 0.05	2	CNS (−)

*n* = number of measurements; CNS (+) (high BBB permeation predicted): *Pe* (×10^−6^ cm s^−1^) > 4.0; CNS (−) (low BBB permeation predicted): *Pe* (×10^−6^ cm s^−1^) < 2.0; CNS (+/−) (BBB permeation uncertain): *Pe* (×10^−6^ cm s^−1^) from 4.0 to 2.0.

**Table 5:** Cytotoxicity of tested compounds on the normal (undifferentiated) SH-SY5Y expressed as IC_50_ ± SEM.

Compound	IC_50_ ± SEM (µM) ^a^
**I-6e**	67.95 ± 0.22
**I-6h**	53.23 ± 3.03
**II-6e**	67.13 ± 3.81
**II-6h**	34.96 ± 1.53
**III-6a**	38.58 ± 0.82
Tacrine	122.11 ± 0.02

^a^ Results are expressed as the mean of three independent measurements.

**Table 6:** SH-SY5Y neurotoxicity testing in vitro ^a^. SH-SY5Y cells were treated with various compounds (100 µM) for 6 h and 24 h. After treatment, the dehydrogenase activity and glutathione levels were detected using WST-1 test and monochlorobimane assay, respectively. The data are presented as mean ± SD (*n* = 3–6; three independent experiments). Statistical significance was analyzed using ANOVA followed by Tukey’s test to compare results with negative controls at significance level *p* = 0.05 (***, *p* < 0.001, vs. untreated cells at appropriate time interval).

Compound (100 µM)	Dehydrogenase Activity (% of Control)		Glutathione Level (% of Control)	
	6 h	24 h	6 h	24 h
**I-6e**	44 ± 13 ***	49 ± 4 ***	70 ± 17 ***	59 ± 12 ***
**I-6h**	62 ± 10 ***	49 ± 2 ***	86 ± 8	71 ± 8 ***
**II-6e**	63 ± 9 ***	48 ± 9 ***	89 ± 19	57 ± 8 ***
**II-6h**	40 ± 3 ***	22 ± 7 ***	58 ± 12 ***	46 ± 11 ***
**III-6a**	107 ± 8	128 ± 4 ***	74 ± 17 ***	49 ± 8 ***

^a^ 100% = untreated cells = negative controls.

## Data Availability

Not applicable.

## Supplementary Materials

The supporting information can be downloaded at: https://www.mdpi.com/article/10.3390/ijms24119124/s1.
