# Supplementary material for: Structure-Guided Design of N-Methylpropargylamino-Quinazoline Derivatives as Multipotent Agents for the Treatment of Alzheimer’s Disease
Source: Int J Mol Sci. 2023 May 23;24(11):9124. doi: 10.3390/ijms24119124 (PMC10252614; doi:10.3390/ijms24119124)
Supplement: Supplementary file 1 [file ijms-24-09124-s001.zip › ijms-2414042-supplementary.pdf]

Supplementary Information

Structure-Guided Design of *N*-Methylpropargylamino-Quinazoline Derivatives as Multipotent Agents for the Treatment of Alzheimer's Disease

Barbora Svobodova [1,2], Lenka Pulkrabkova [1,2], Dawid Panek [1,3], Anna Misiachna [4,5], Marharyta Kolcheva [4], Rudolf Andrys [6], Jiri Handl [7], Jan Capek [7], Pavlina Nyvltova [7], Tomas Rousar [7], Lukas Prchal [1], Vendula Hepnarova [1,2], Martina Hrabinova [1,2], Lubica Muckova [1,2], Daniela Tosnerova [8], Galina Karabanovich [8], Vladimir Finger [1,8], Ondrej Soukup [1,2], Martin Horak [4], and Jan Korabecny [1]

[1] *Biomedical Research Center, University Hospital Hradec Kralove, Sokolska 581, 500 05 Hradec Kralove, Czech Republic*

[2] *Department of Toxicology and Military Pharmacy, Faculty of Military Health Sciences, University of Defence, Trebesska 1575, 500 01 Hradec Kralove, Czech Republic*

[3] *Department of Physicochemical Drug Analysis, Chair of Pharmaceutical Chemistry, Faculty of Pharmacy, Jagiellonian University Medical College, 30-688 Kraków, Medyczna 9, Poland*

[4] *Department of Neurochemistry, Institute of Experimental Medicine of the Czech Academy of Sciences, Videnska 1083, 142 20 Prague, Czech Republic*

[5] *Department of Physiology, Faculty of Science, Charles University in Prague, Albertov 6, 12843, Prague 2, Czech Republic*

[6] *Department of Chemistry, Faculty of Science, University Hradec Kralove, Rokitanskeho 62, 500 03 Hradec Kralove, Czech Republic*

[7] *Department of Biological and Biochemical Sciences, Faculty of Chemical Technology, University of Pardubice, Studentska 573, 532 10 Pardubice, Czech Republic*

[8] *Department of Organic and Bioorganic Chemistry, Faculty of Pharmacy in Hradec Kralove, Charles University, Akademika Heyrovskeho 1203, 50005 Hradec Kralove, Czech Republic*

Table of contents

1. **Physiochemical Properties and Drug-likeness of Final Compounds** 3
2. **Cholinesterase Inhibitory Activity** 5
3. **Anti-MAOs Activity Screening** 6
4. **^{1}H and ^{13}C NMR Spectra** 7
5. **HPLC Analysis of Compounds** 96
6. **References** 145

1. Physiochemical Properties and Drug-likeness of Final Compounds

For the prediction of physiochemical properties (**Table S1**) was used MarvinSketch 20.4.0, ChemAxon Ltd. Drug-likeness of designed derivatives was evaluated by SwissADME online tool [1].

Table S1. Physiochemical parameters of compounds **I-III/6a-6h** THA and Memantine.

Compound	MW	pKa	logP	HBA	HBD	TPSA
**I-6a**	212.25	6.55	2.21	2	1	55.04
**I-6b**	226.28	6.45	2.51	2	1	41.05
**I-6c**	240.30	6.42	2.86	2	1	41.05
**I-6d**	254.33	6.40	3.28	2	1	41.05
**I-6e**	268.36	6.42	3.83	2	1	41.05
**I-6f**	270.33	6.40	2.46	3	1	50.28
**I-6g**	252.31	6.39	2.97	2	1	41.05
**I-6h**	294.39	6.39	4.31	2	1	41.05
**II-6a**	246.70	5.82	2.81	2	1	55.04
**II-6b**	260.72	5.72	3.11	2	1	41.05
**II-6c**	304.77	5.67	3.06	3	1	50.28
**II-6d**	288.78	5.67	3.88	2	1	41.05
**II-6e**	302.80	5.69	4.44	2	1	41.05
**II-6f**	304.77	5.67	3.06	3	1	50.28
**II-6g**	286.76	5.66	3.58	2	1	41.05
**II-6h**	328.84	5.66	4.91	2	1	41.05
**III-6a**	242.28	6.60	2.05	3	1	64.27
**III-6b**	256.30	6.51	2.35	3	1	50.28
**III-6c**	270.33	6.48	2.71	3	1	50.28
**III-6d**	284.36	6.48	3.12	3	1	50.28
**III-6e**	298.38	6.48	3.67	3	1	50.28
**III-6f**	300.36	6.46	2.30	4	1	59.51
**III-6g**	282.34	6.45	2.82	3	1	50.28
**III-6h**	324.42	6.45	4.15	3	1	50.28
**THA**	198.26	8.95	2.63	2	1	38.91
**Memantine**	179.30	10.70	2.07	1	1	26.02

MW = molecular weight, pKa = acid dissociation constant, logP = partition coefficient, HBA = hydrogen bond acceptor, HBB = hydrogen bond donor, TPSA = topological polar surface area

Table S2. Drug-likeness of derivatives **I-III/6a-6h**; THA and memantine.

Compound	Drug-likeness				
	**Lipinski [a]**	**Ghose [b]**	**Veber [c]**	**Egan [d]**	**Muegge [e]**
**I-6a**	Yes	Yes	Yes	Yes	Yes
**I-6b**	Yes	Yes	Yes	Yes	Yes
**I-6c**	Yes	Yes	Yes	Yes	Yes
**I-6d**	Yes	Yes	Yes	Yes	Yes
**I-6e**	Yes	Yes	Yes	Yes	Yes
**I-6f**	Yes	Yes	Yes	Yes	Yes
**I-6g**	Yes	Yes	Yes	Yes	Yes
**I-6h**	Yes	Yes	Yes	Yes	Yes
**II-6a**	Yes	Yes	Yes	Yes	Yes
**II-6b**	Yes	Yes	Yes	Yes	Yes
**II-6c**	Yes	Yes	Yes	Yes	Yes
**II-6d**	Yes	Yes	Yes	Yes	Yes
**II-6e**	Yes	Yes	Yes	Yes	Yes
**II-6f**	Yes	Yes	Yes	Yes	Yes
**II-6g**	Yes	Yes	Yes	Yes	Yes
**II-6h**	Yes	Yes	Yes	Yes	Yes
**III-6a**	Yes	Yes	Yes	Yes	Yes
**III-6b**	Yes	Yes	Yes	Yes	Yes
**III-6c**	Yes	Yes	Yes	Yes	Yes
**III-6d**	Yes	Yes	Yes	Yes	Yes
**III-6e**	Yes	Yes	Yes	Yes	Yes
**III-6f**	Yes	Yes	Yes	Yes	Yes
**III-6g**	Yes	Yes	Yes	Yes	Yes
**III-6h**	Yes	Yes	Yes	Yes	Yes
**THA**	Yes	Yes	Yes	Yes	No [f]
**Memantine**	Yes	Yes	Yes	Yes	No [g]

[a] Lipinski represent [2], MW < 500 Da, logP <5, HBD <5, HBA <10; [b] Ghose [3], MW = 180 – 480, MR (molar refractivity) = 40 – 130, logP = -0.4 – +5.6, number of atoms = 20 – 70; [c] Veber [4], rotatable bonds <10, TPSA <140; [d] Egan [5], TPSA <132, logP <5.88; [e] Muegge [6], MW = 200 – 500, log P = -2 -+5, HBD <5, HBA <10, MR = 40 – 130, rotatable bonds <8, heavy atoms = 20 – 70, TPSA <120, net charge -2 -+2; [f] 1 violation, [g] 2 violations.

2. Cholinesterase Inhibitory Activity

Table S3. The inhibitory activities of compounds **I-III/6a-6h** for human acetylcholinesterase (*h*AChE) and human butyrylcholinesterase (*h*BChE).

Compound	% of AChE inhibition 10^{-6} M	% of BChE inhibition 10^{-6} M
**I-6a**	5	0
**I-6b**	5	3
**I-6c**	4	0
**I-6d**	4	1
**I-6e**	3	4
**I-6f**	5	1
**I-6g**	4	2
**I-6h**	4	0
**II-6a**	7	2
**II-6b**	15	2
**II-6c**	4	0
**II-6d**	1	3
**II-6e**	9	0
**II-6f**	9	2
**II-6g**	4	0
**II-6h**	3	3
**III-6a**	9	1
**III-6b**	4	1
**III-6c**	7	2
**III-6d**	2	2
**III-6e**	8	1
**III-6f**	6	1
**III-6g**	5	0
**III-6h**	3	2
**THA**	59	14
**7-MEOTA**	91	18

3. Anti-MAOs Activity Screening

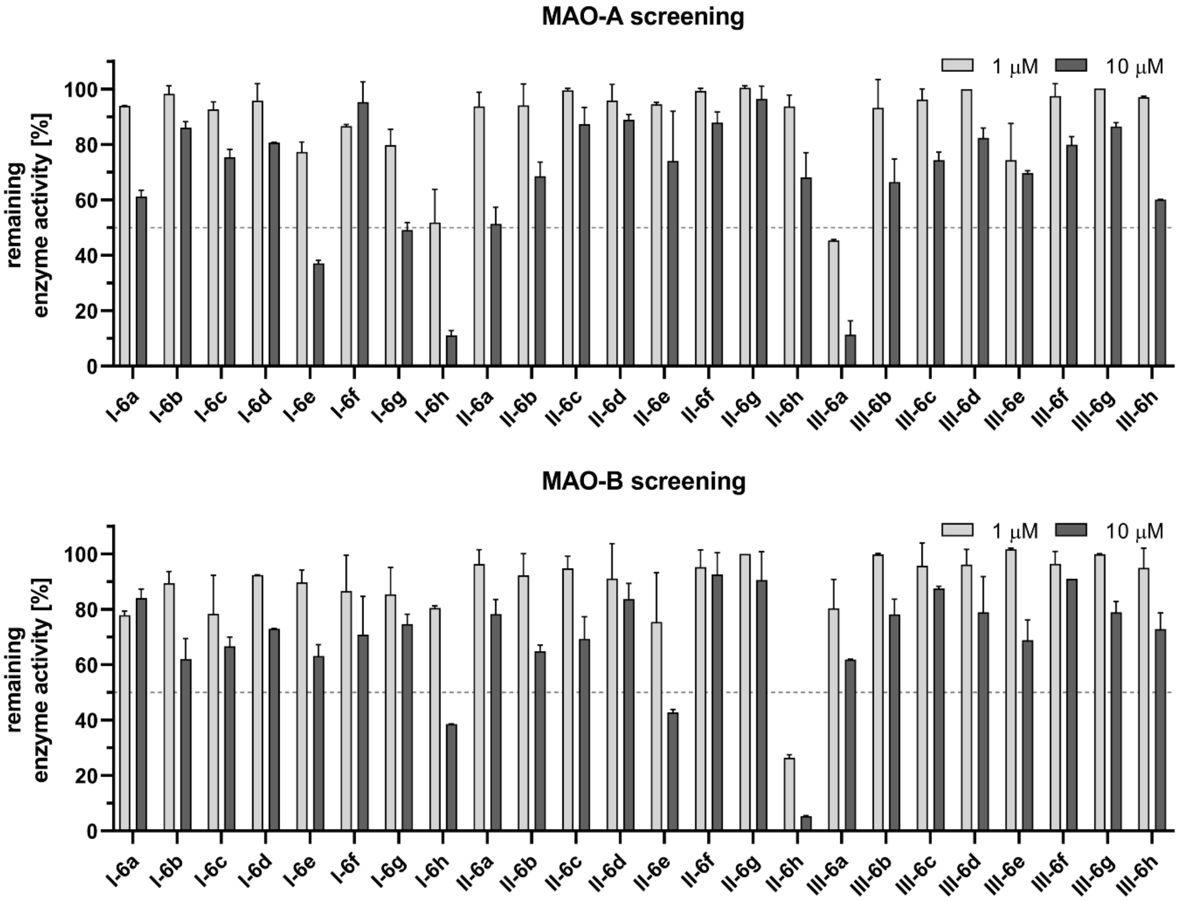

Figure S1. Screening MAO-A/B.

4. ^{1}H and ^{13}C NMR Spectra

Fig. S2. ^{1}H NMR of 2-chloroquinazolin-4-amine (I-5a)

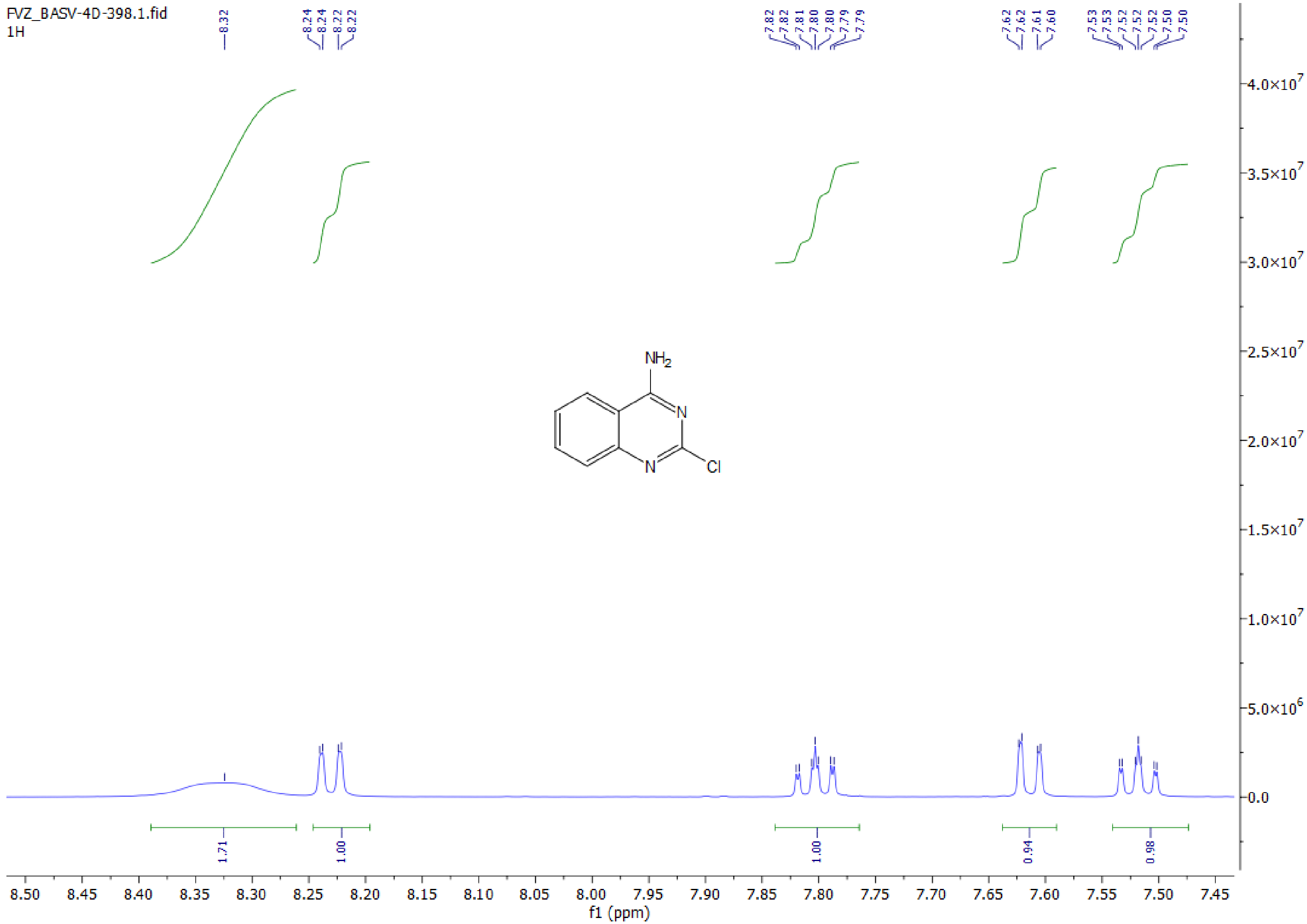

Fig. S3. ^{13}C NMR of 2-chloroquinazolin-4-amine (I-5a)

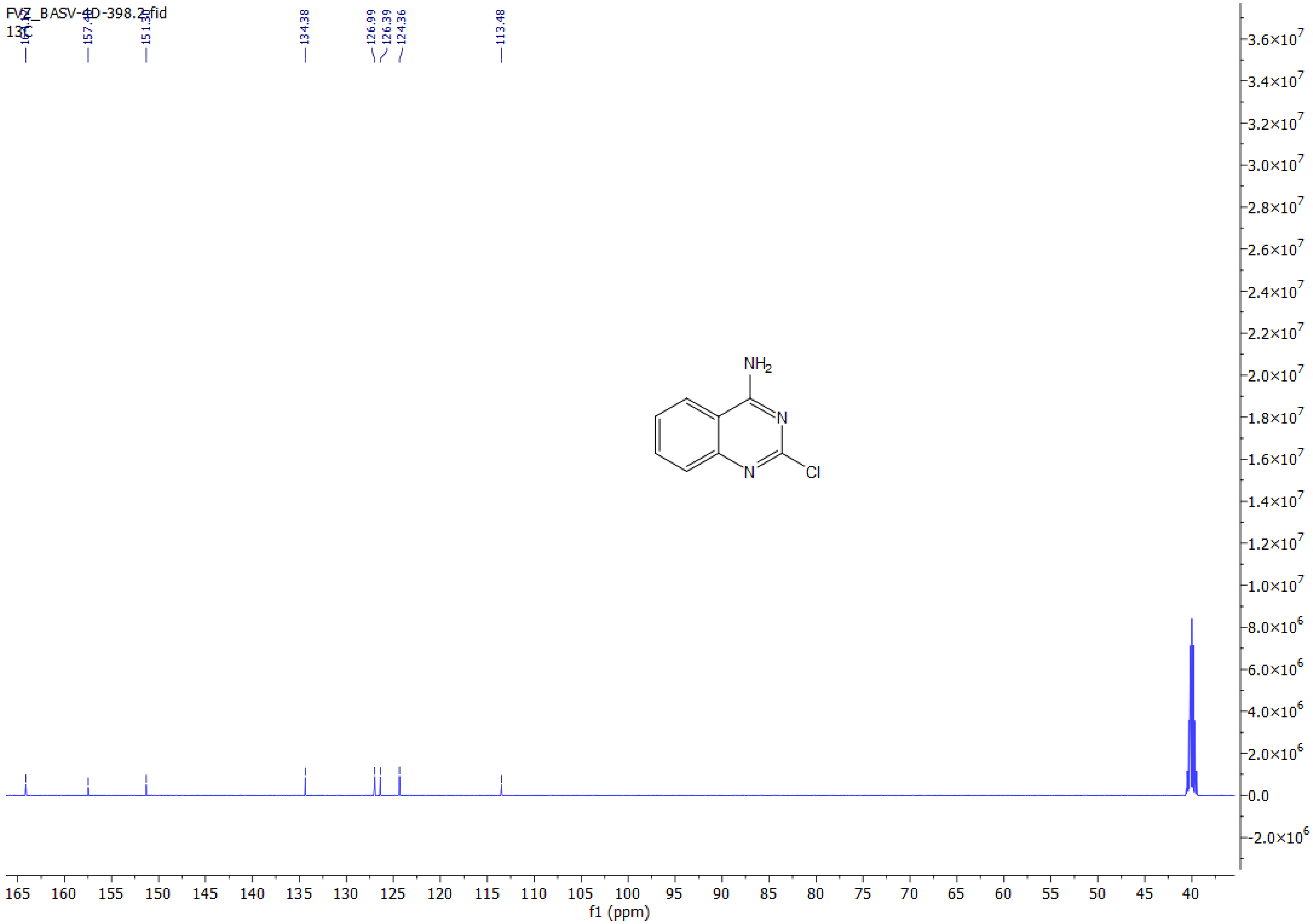

Fig. S4. ^{1}H NMR of 2-chloro-*N*-methylquinazolin-4-amine (I-5b)

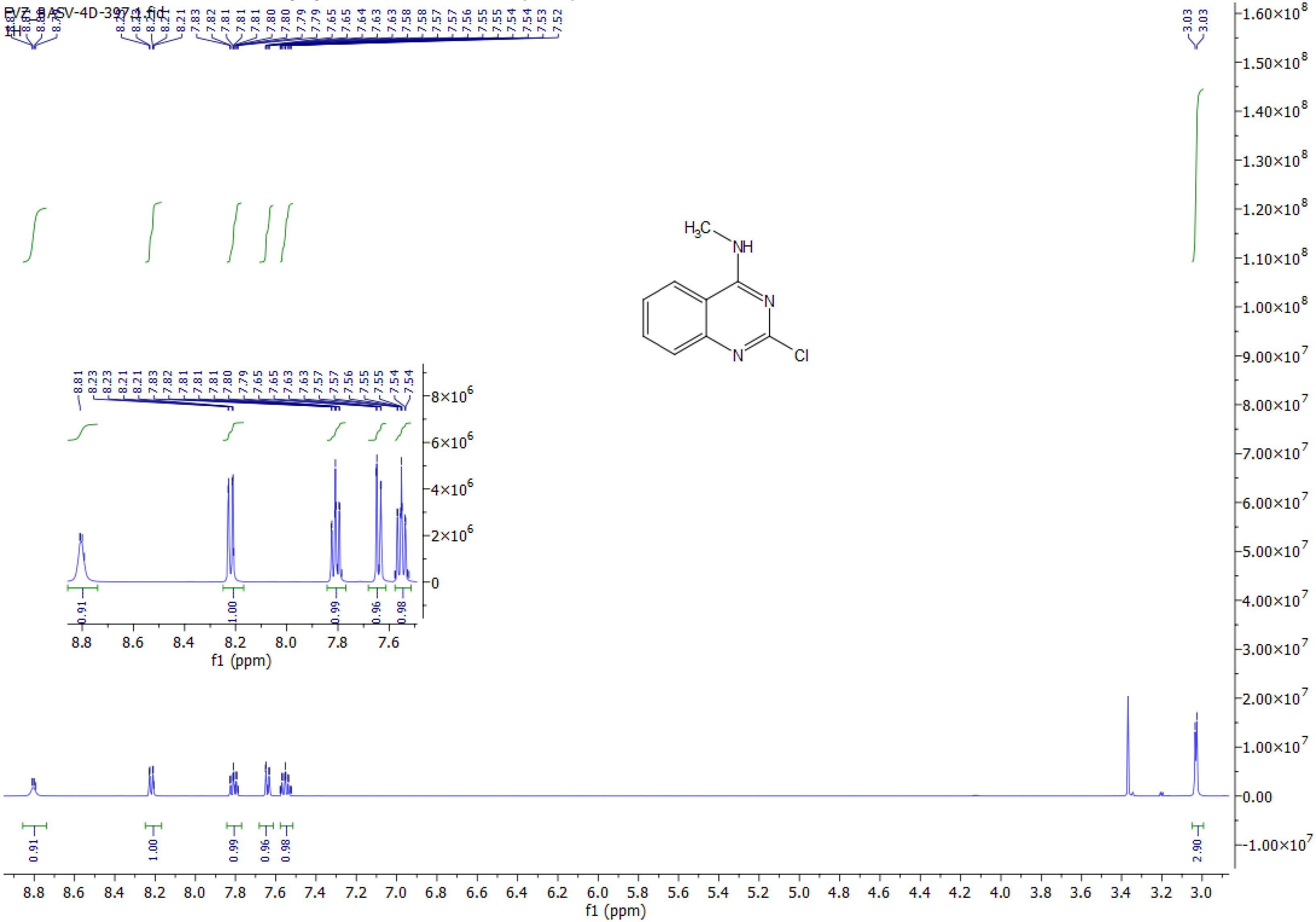

Fig. S5. ^{13}C NMR of 2-chloro-*N*-methylquinazolin-4-amine (I-5b)

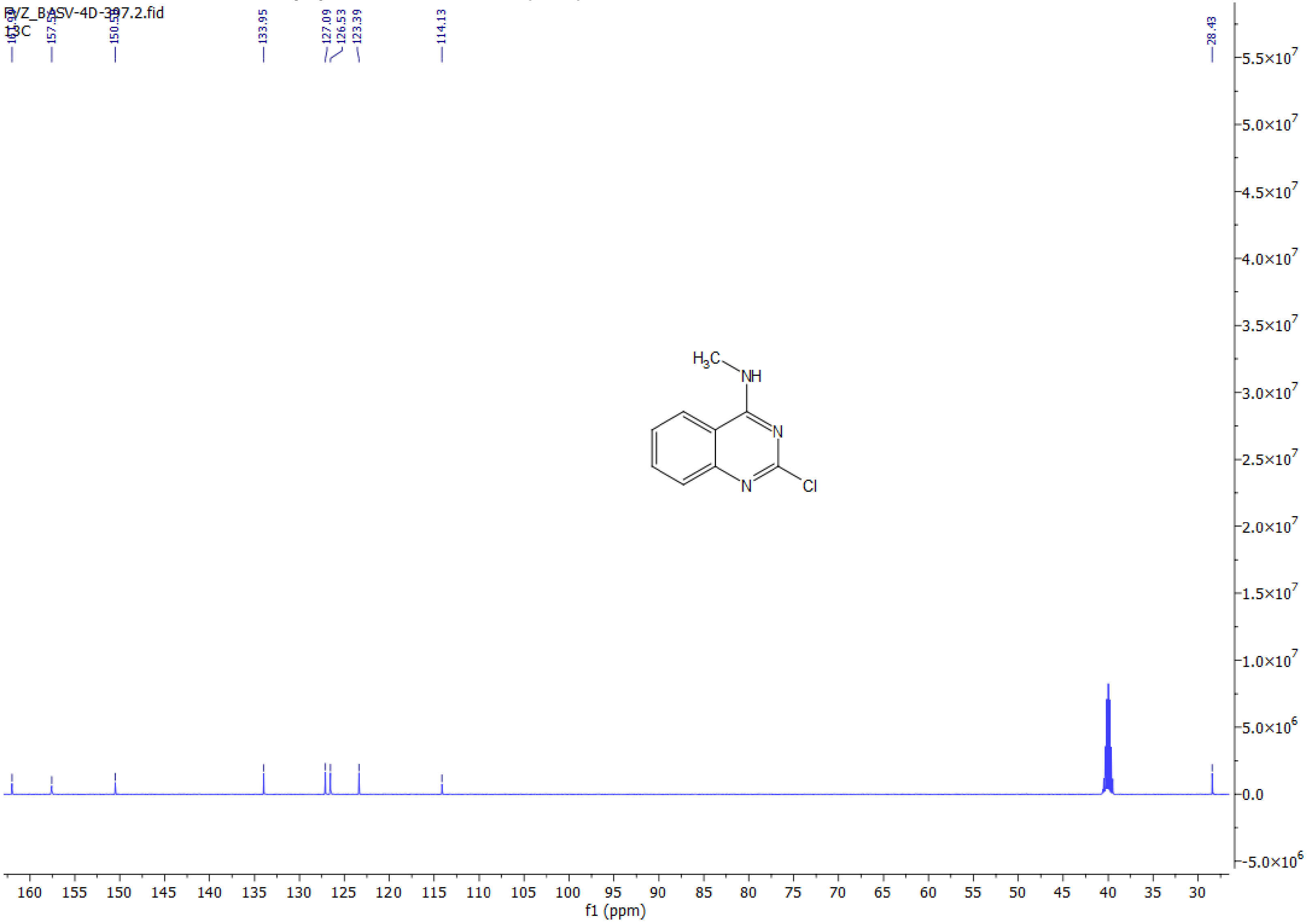

Fig. S6. ^{1}H NMR of 2-chloro-*N*-ethylquinazolin-4-amine (I-5c)

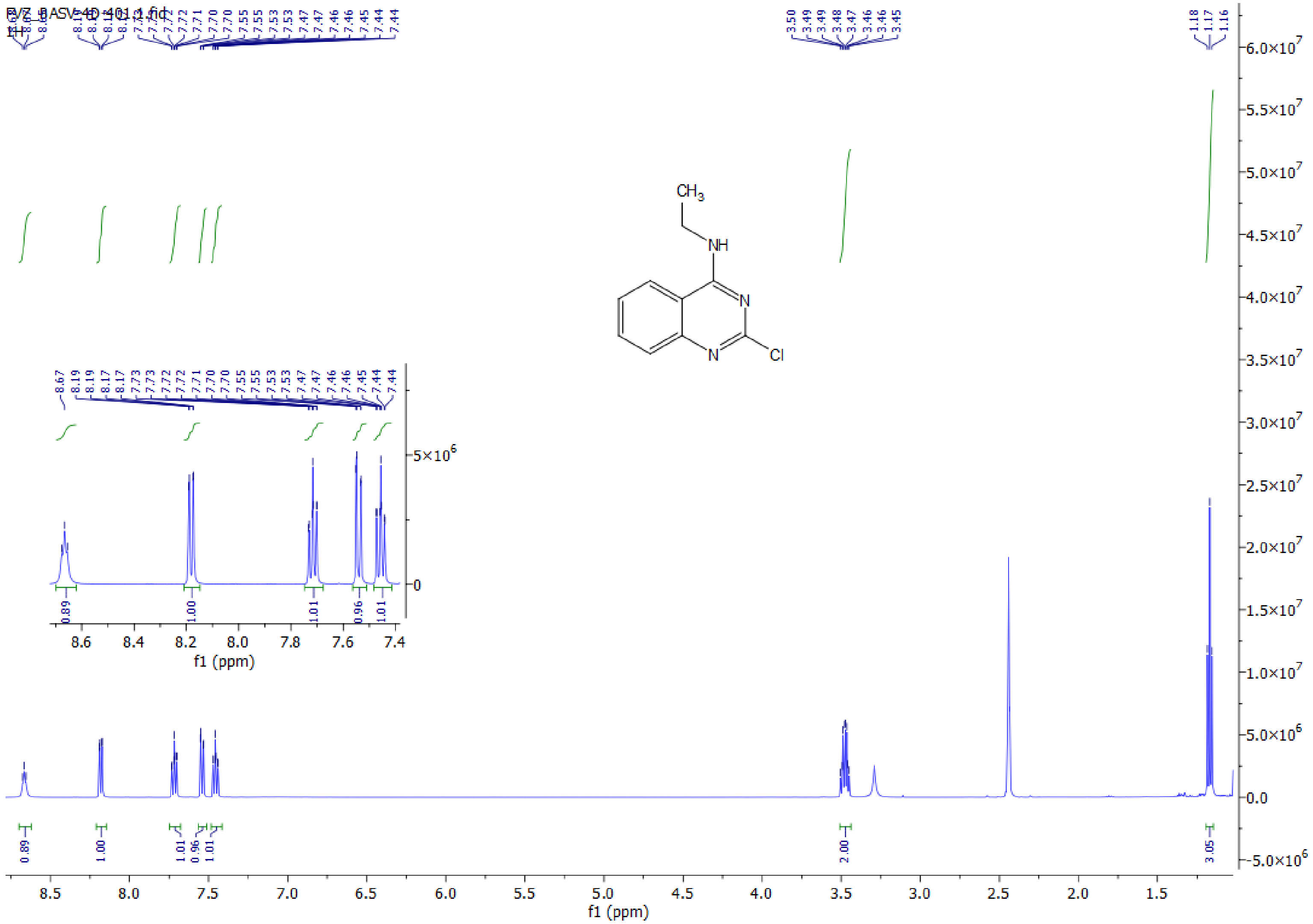

Fig. S7. ^{13}C NMR of 2-chloro-*N*-ethylquinazolin-4-amine (I-5c)

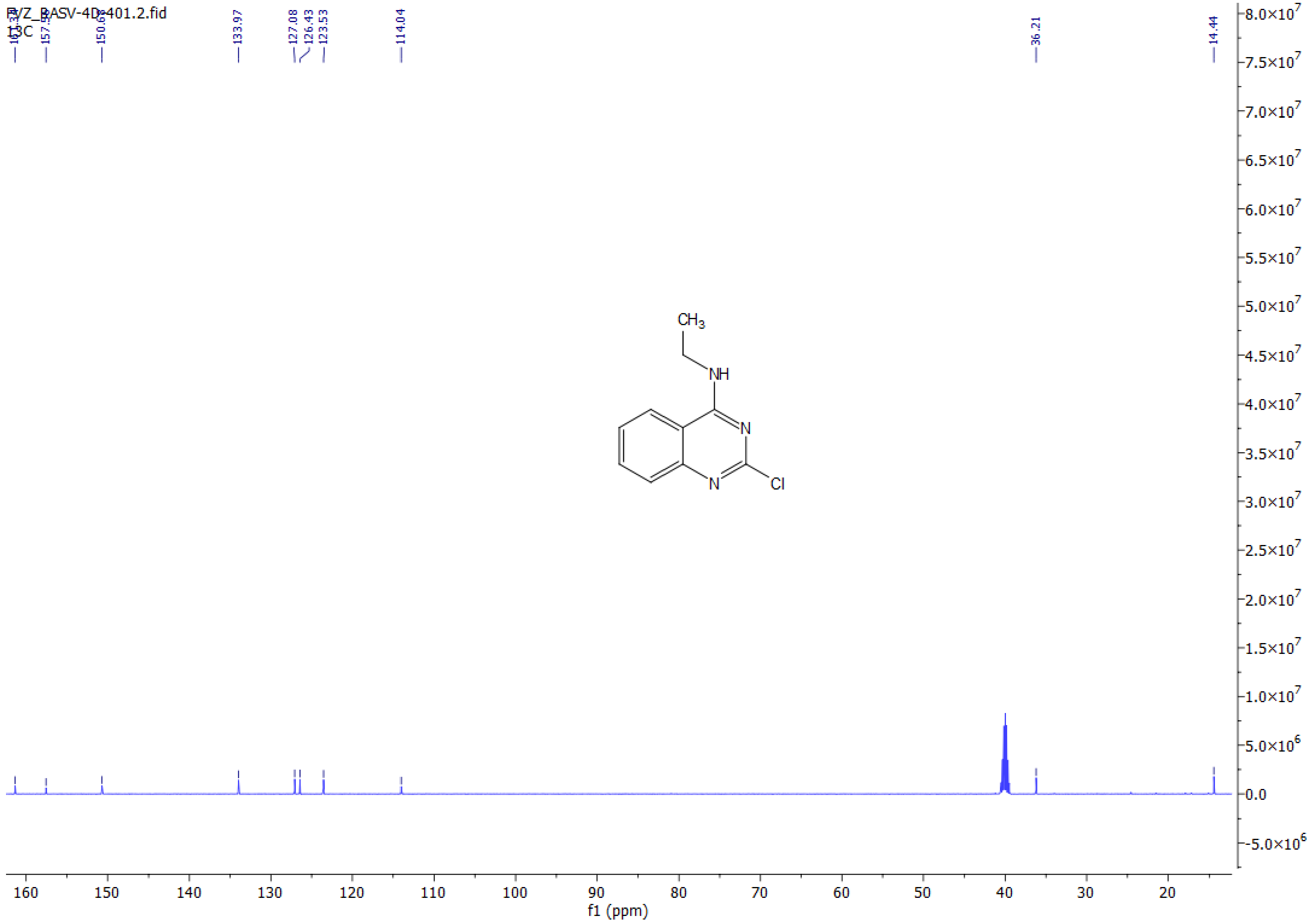

Fig. S8. ^{1}H NMR of 2-chloro-*N*-(propan-2-yl)quinazolin-4-amine (I-5d)

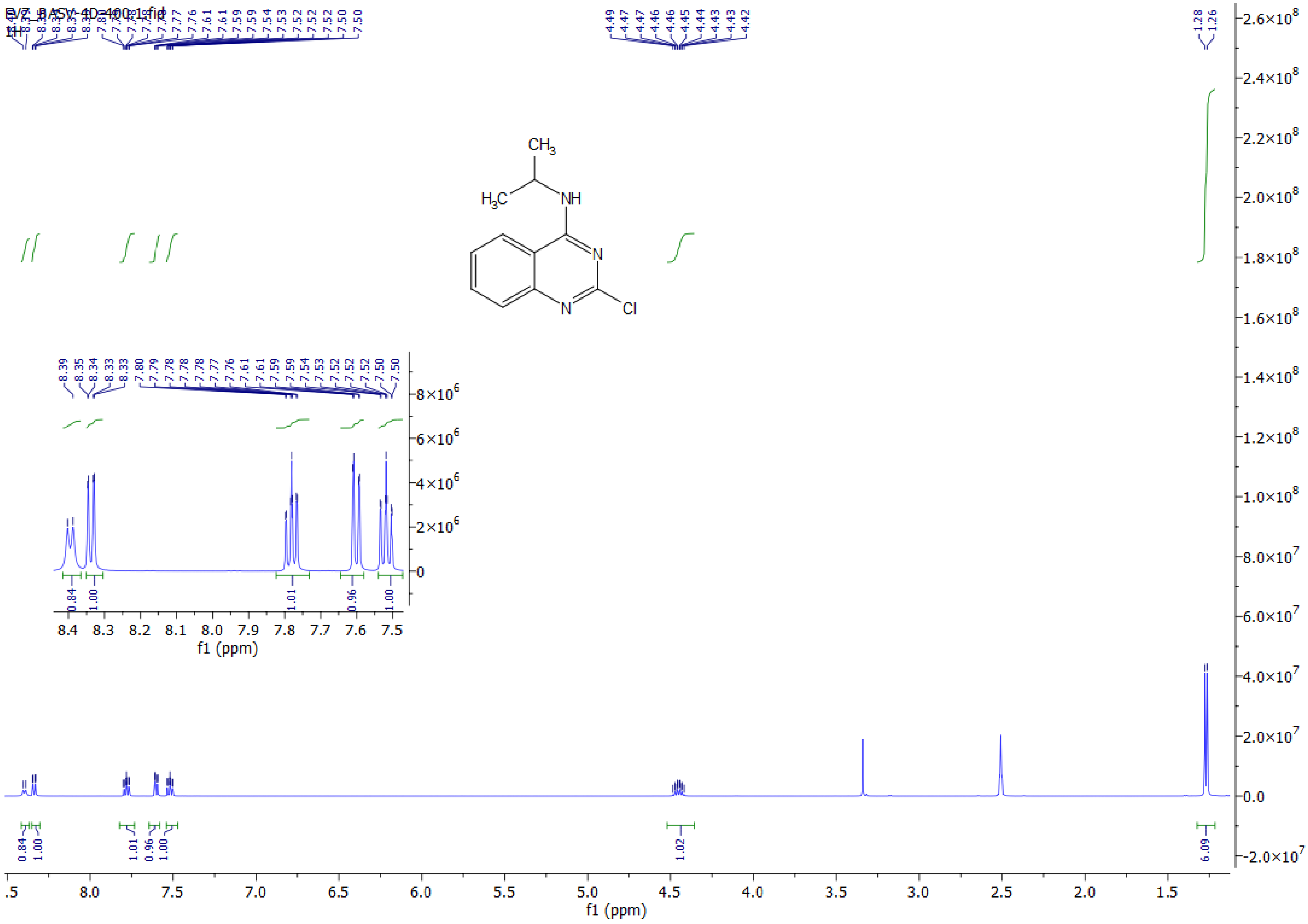

Fig. S9. ^{13}C NMR of 2-chloro-*N*-(propan-2-yl)quinazolin-4-amine (I-5d)

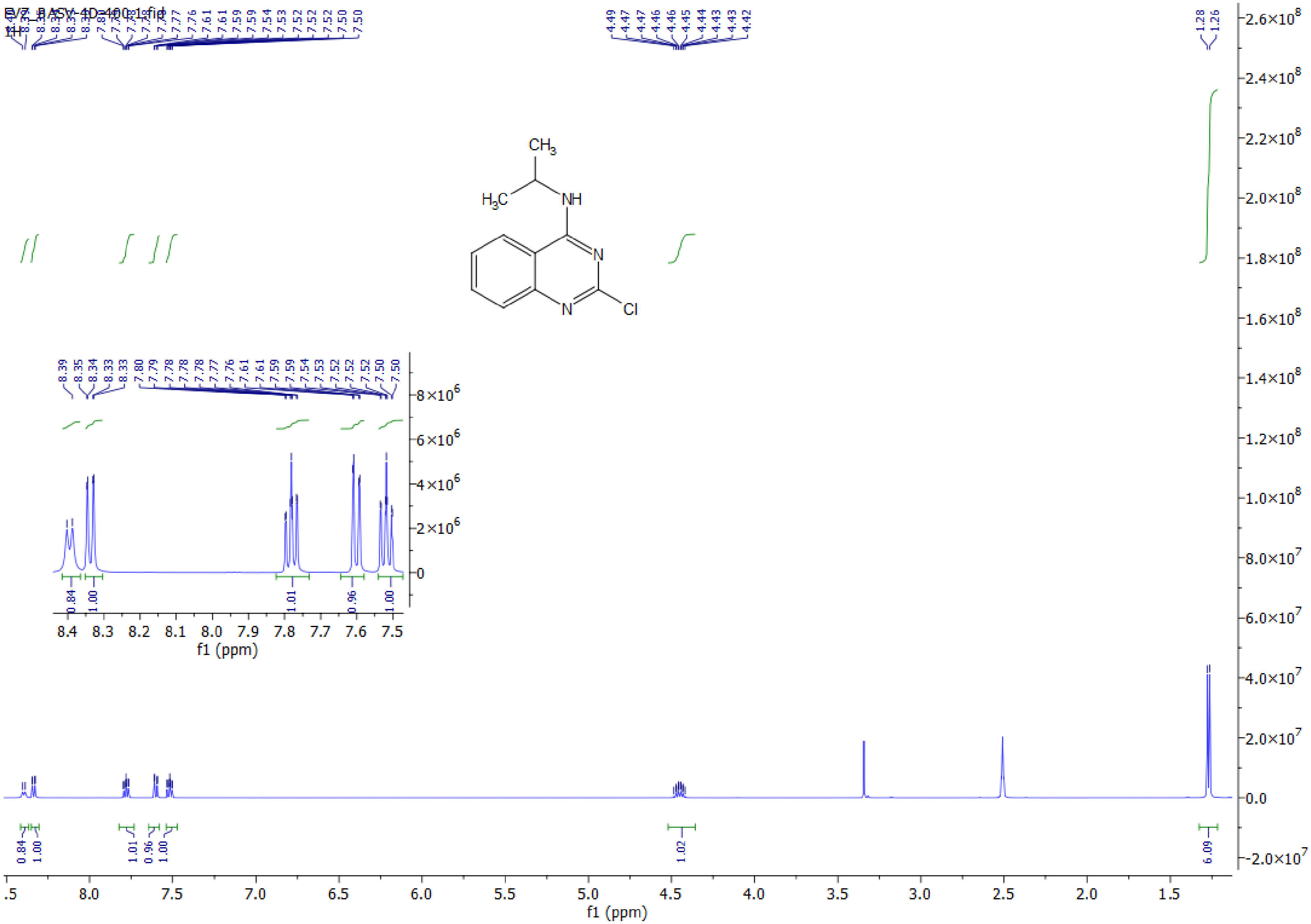

Fig. S10. ^{1}H NMR of *N*-butyl-2-chloroquinazolin-4-amine (I-5e)

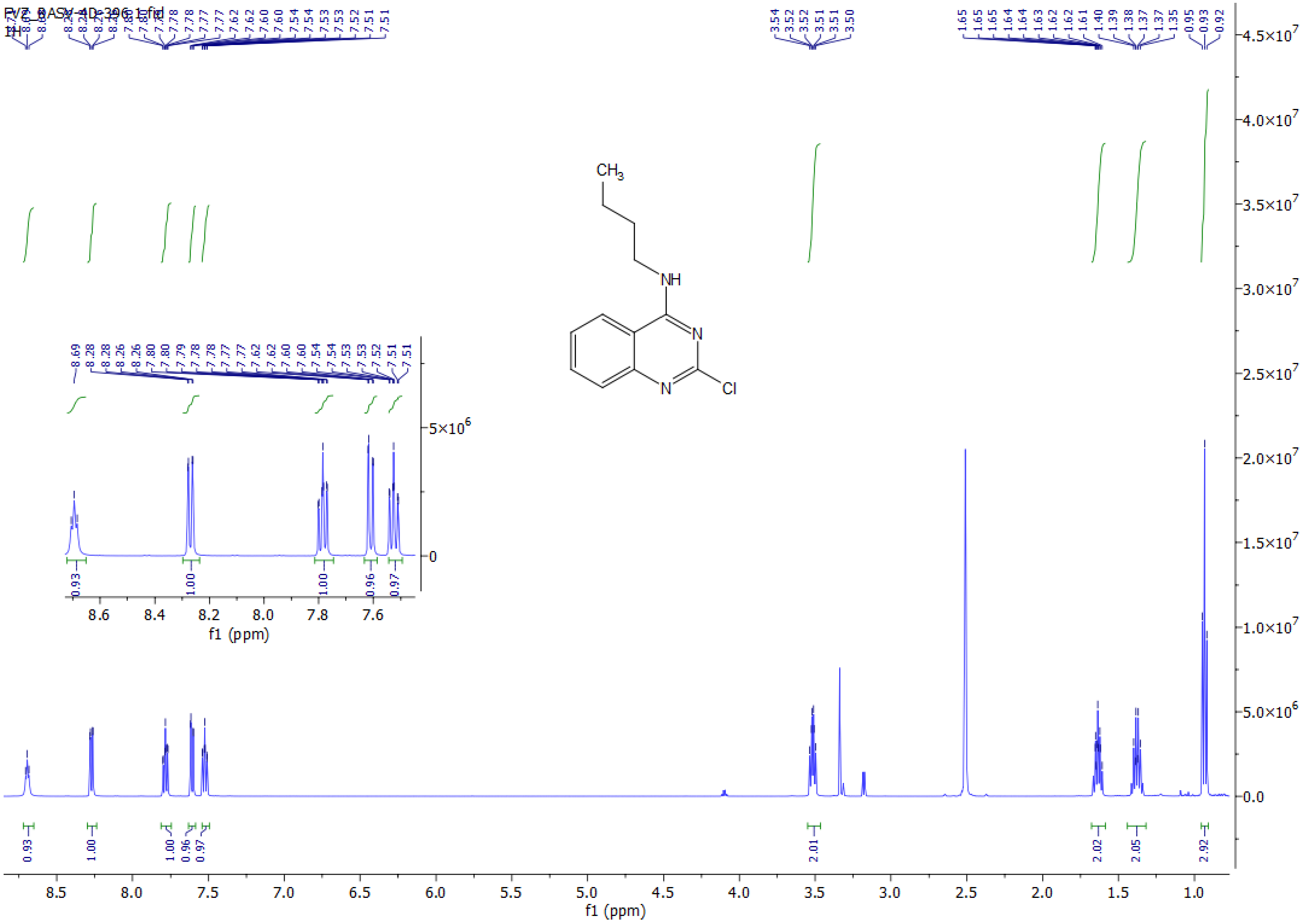

Fig. S11. ^{13}C NMR of *N*-butyl-2-chloroquinazolin-4-amine (I-5e)

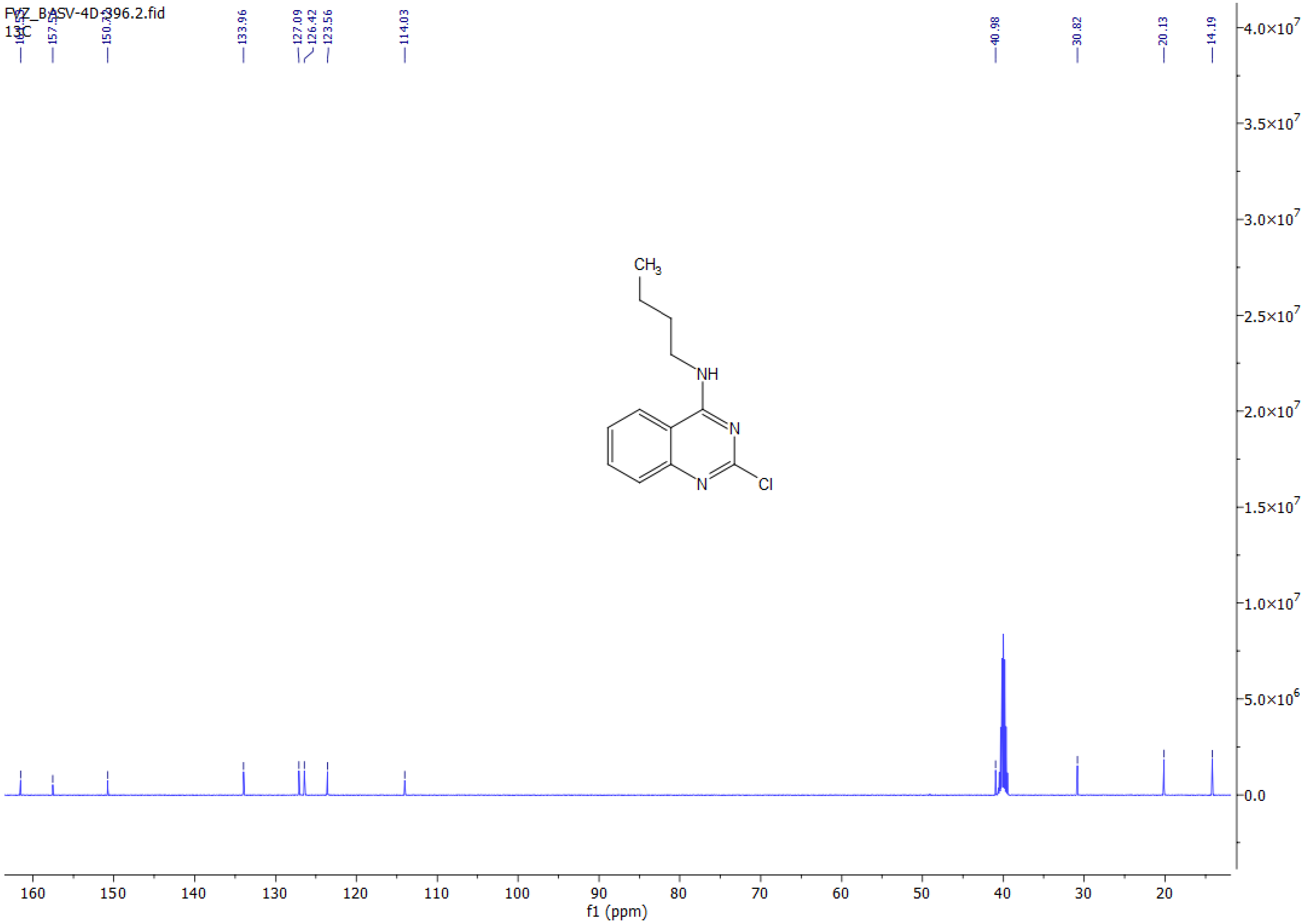

Fig. S12. ^{1}H NMR of 2-chloro-*N*-(2-methoxyethyl)quinazolin-4-amine (I-5f)

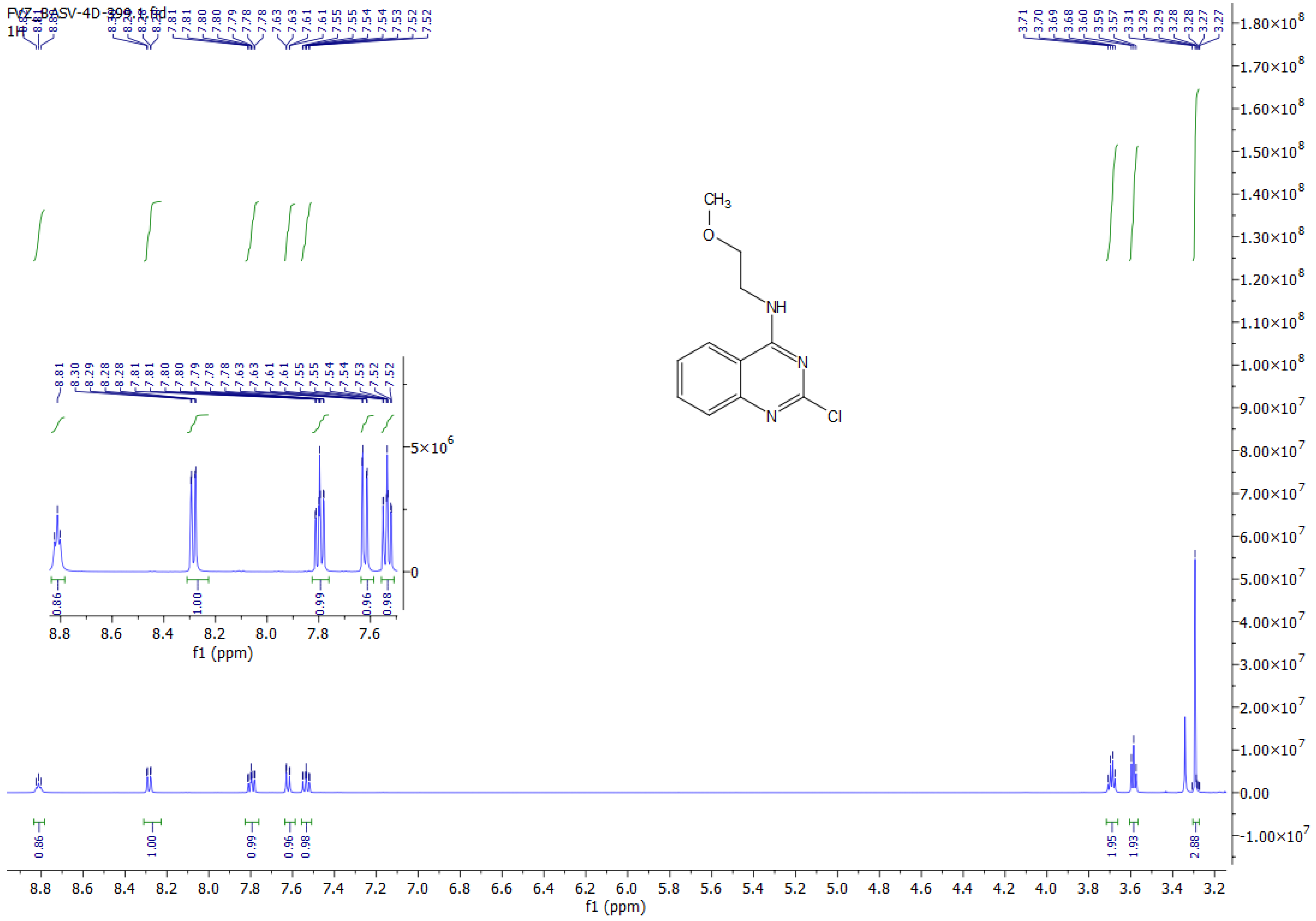

Fig. S13. ^{13}C NMR of 2-chloro-*N*-(2-methoxyethyl)quinazolin-4-amine (I-5f)

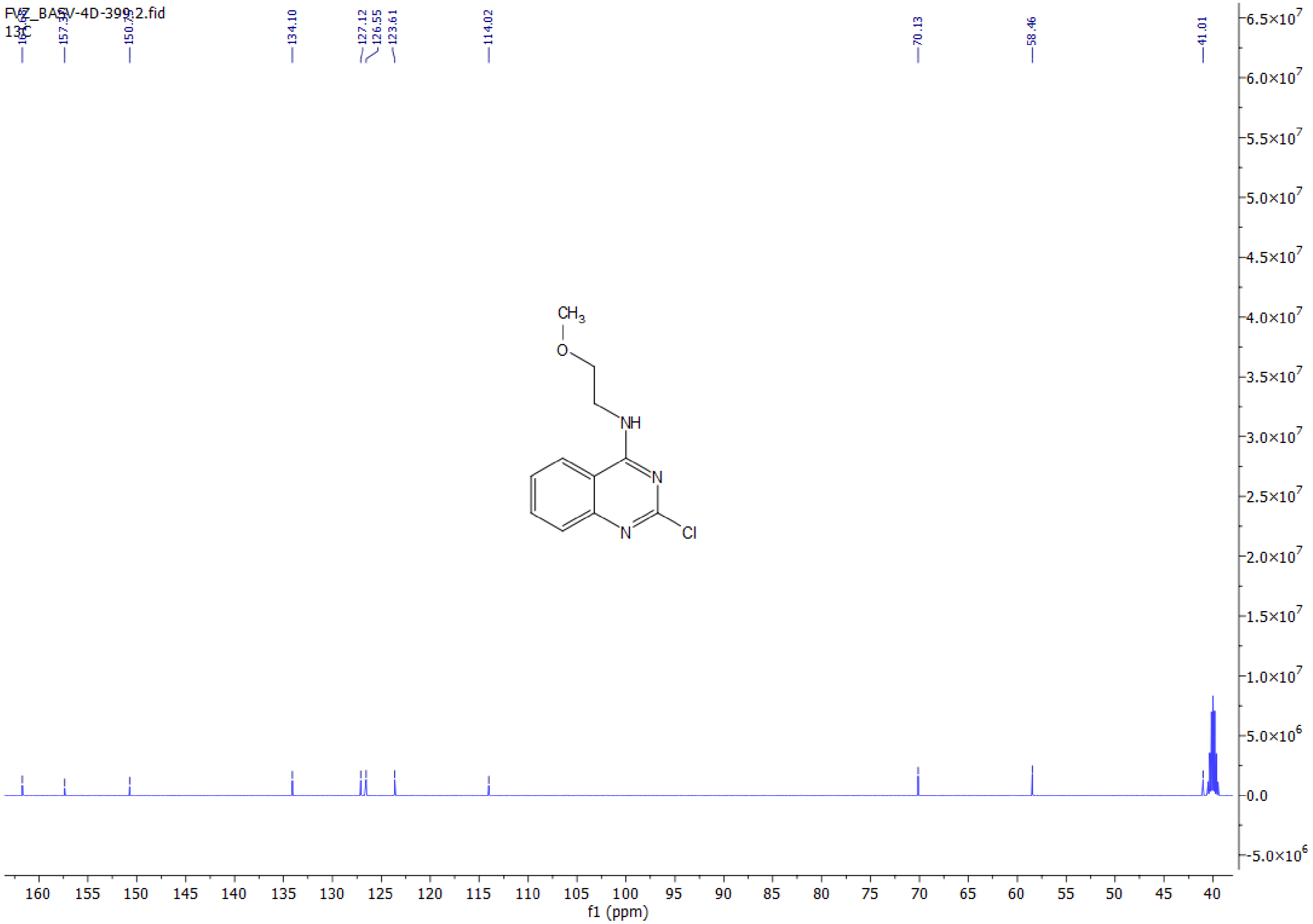

Fig. S14. ^{1}H NMR of 2,7-dichloroquinazolin-4-amine (II-5a)

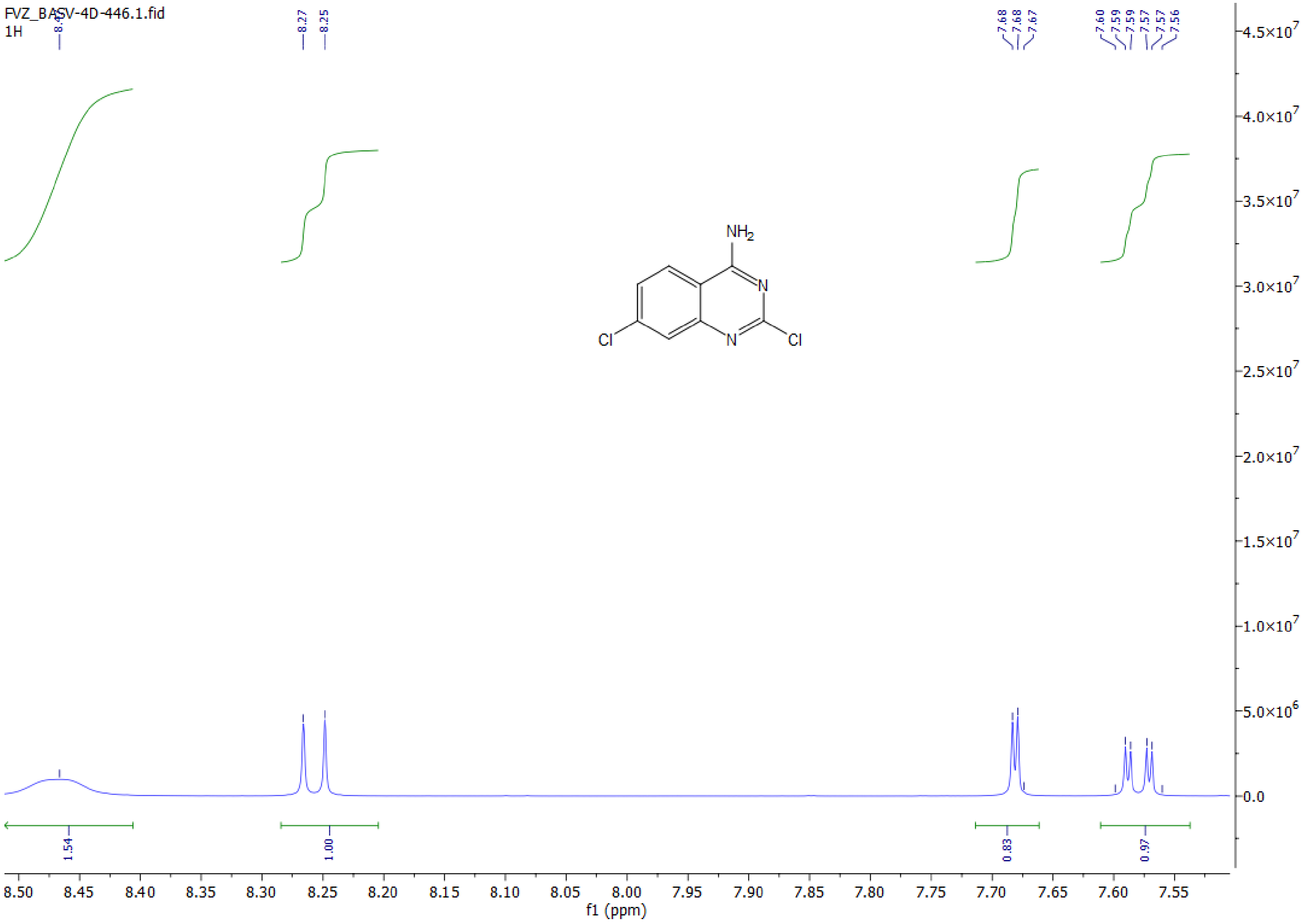

Fig. S15. ^{13}C **NMR of 2,7-dichloroquinazolin-4-amine (II-5a)**

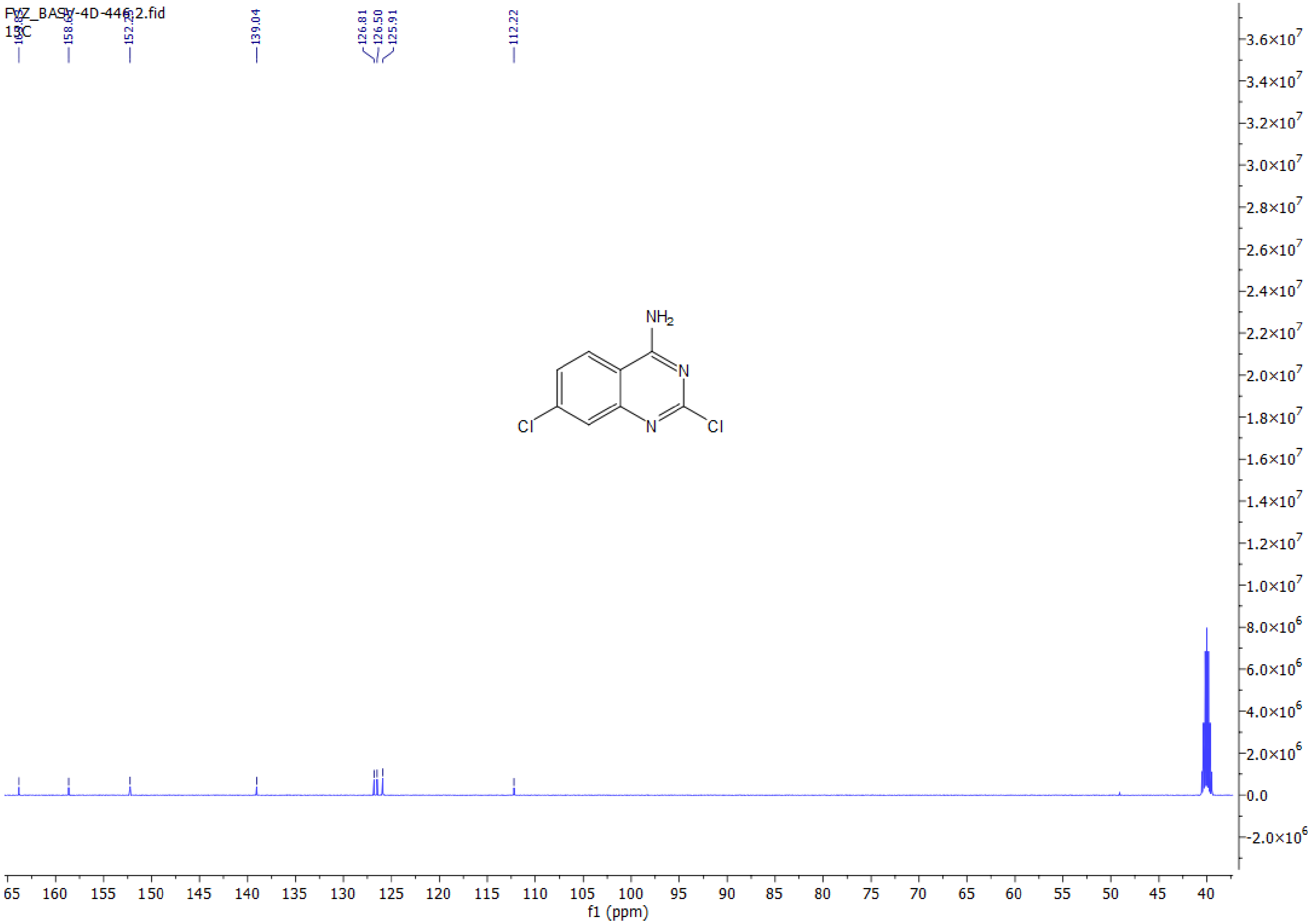

Fig. S16. ^{1}H NMR of 2,7-dichloro-*N*-methylquinazolin-4-amine (II-5b)

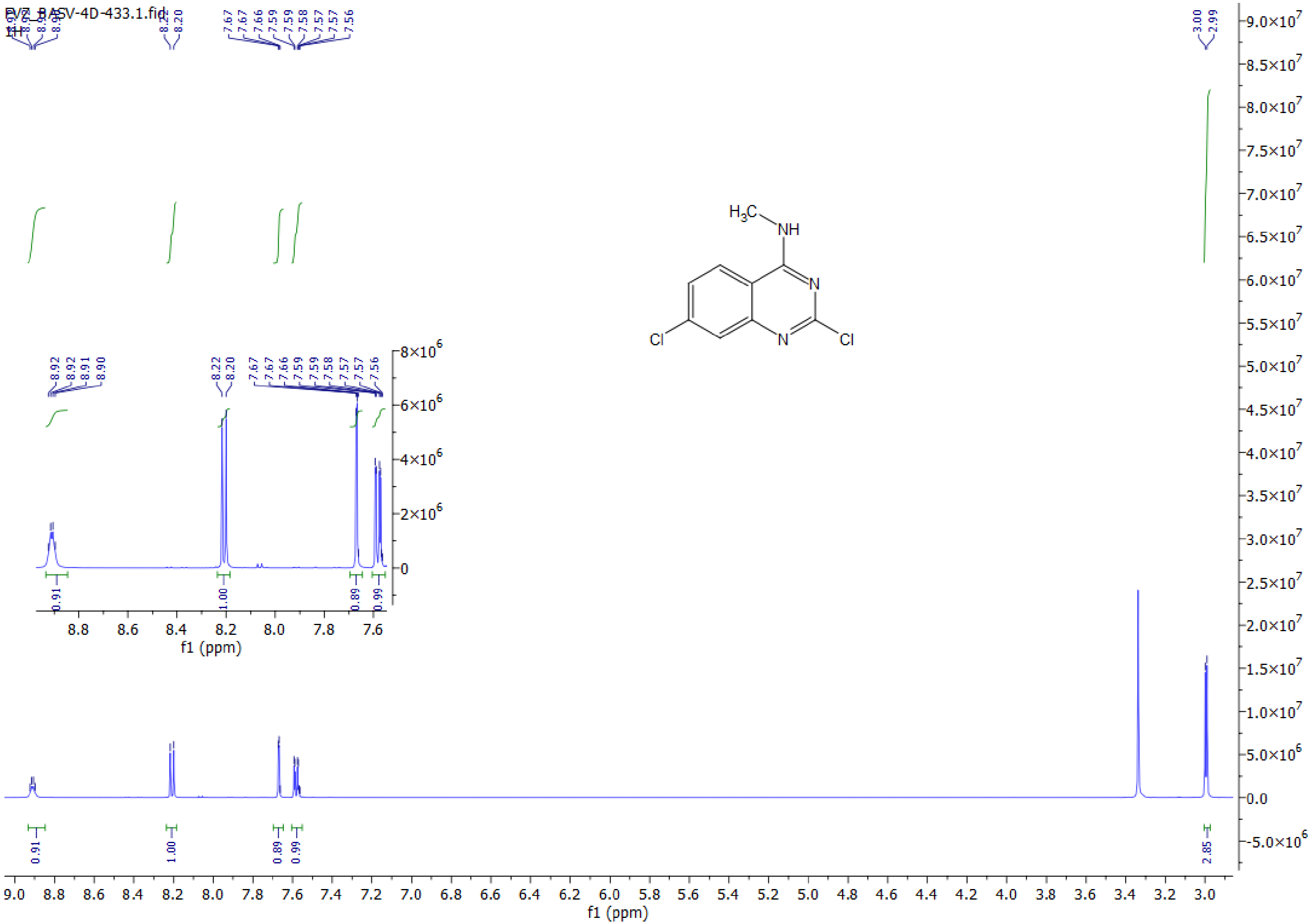

Fig. S17. ^{13}C NMR of 2,7-dichloro-*N*-methylquinazolin-4-amine (II-5b)

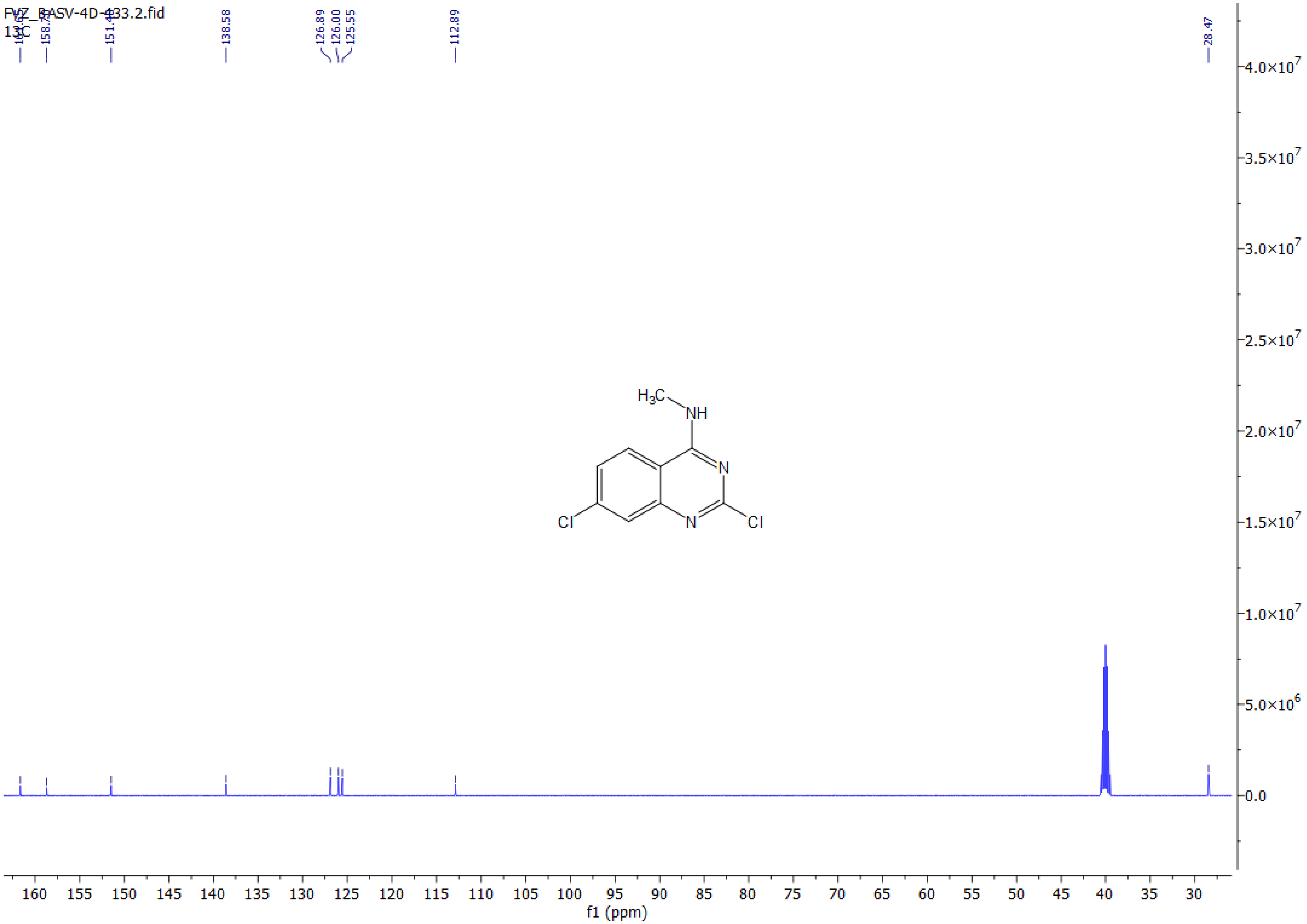

Fig. S18. ^{1}H NMR of 2,7-dichloro-*N*-ethylquinazolin-4-amine (II-5c)

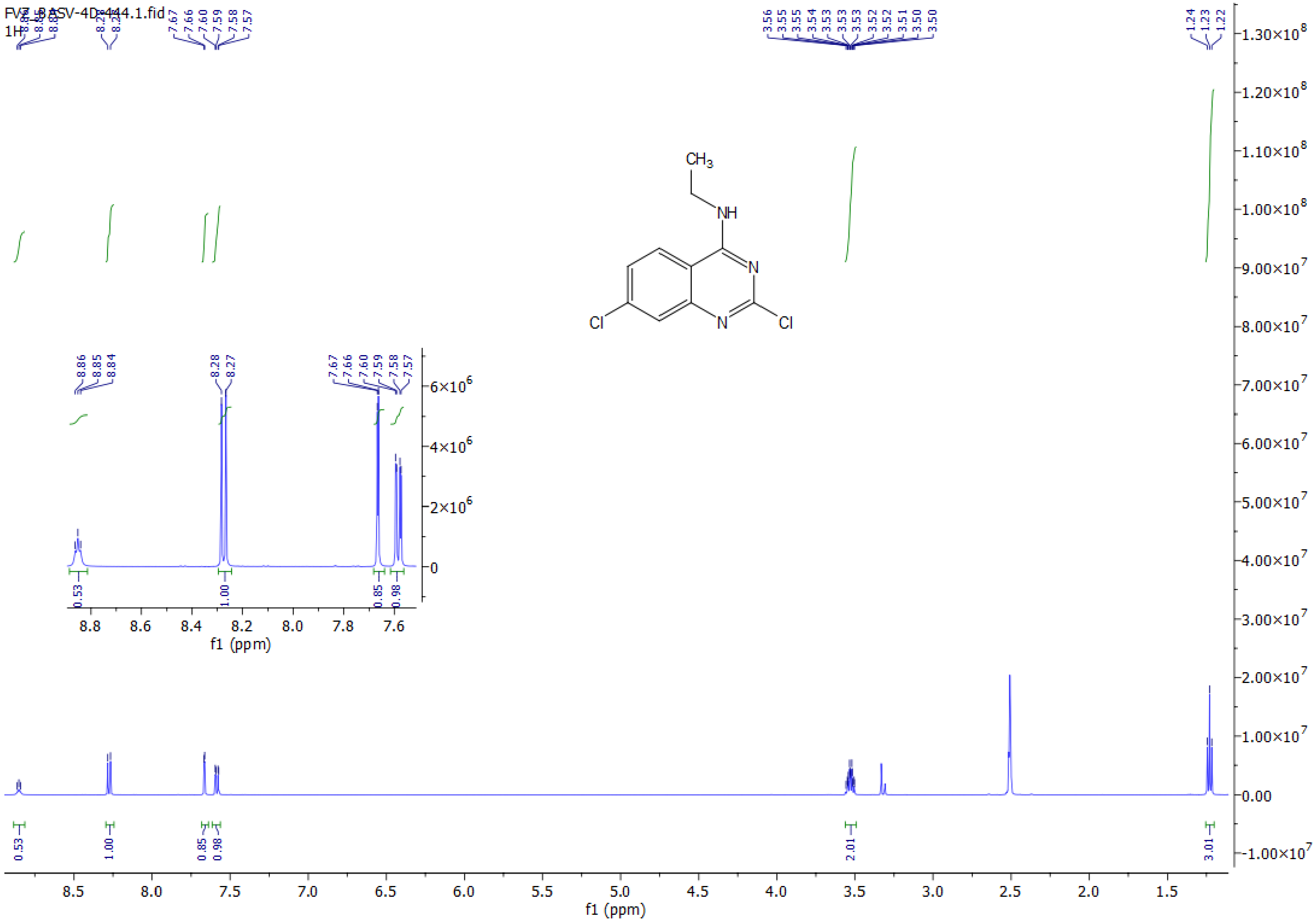

Fig. S19. ^{13}C **NMR of 2,7-dichloro-*N*-ethylquinazolin-4-amine (II-5c)**

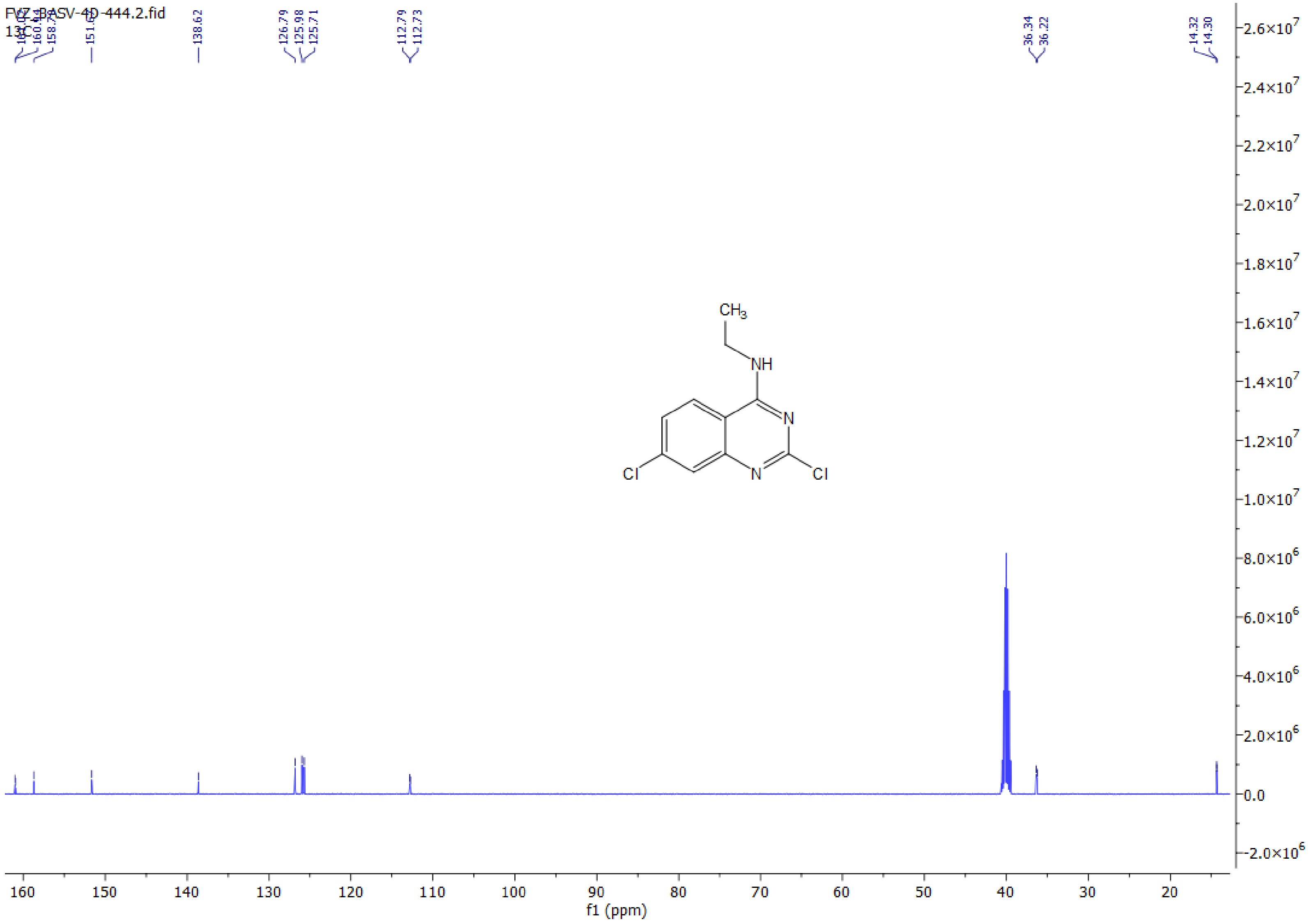

Fig. S20. ^{1}H NMR of 2,7-dichloro-*N*-(propan-2-yl)quinazolin-4-amine (II-5d)

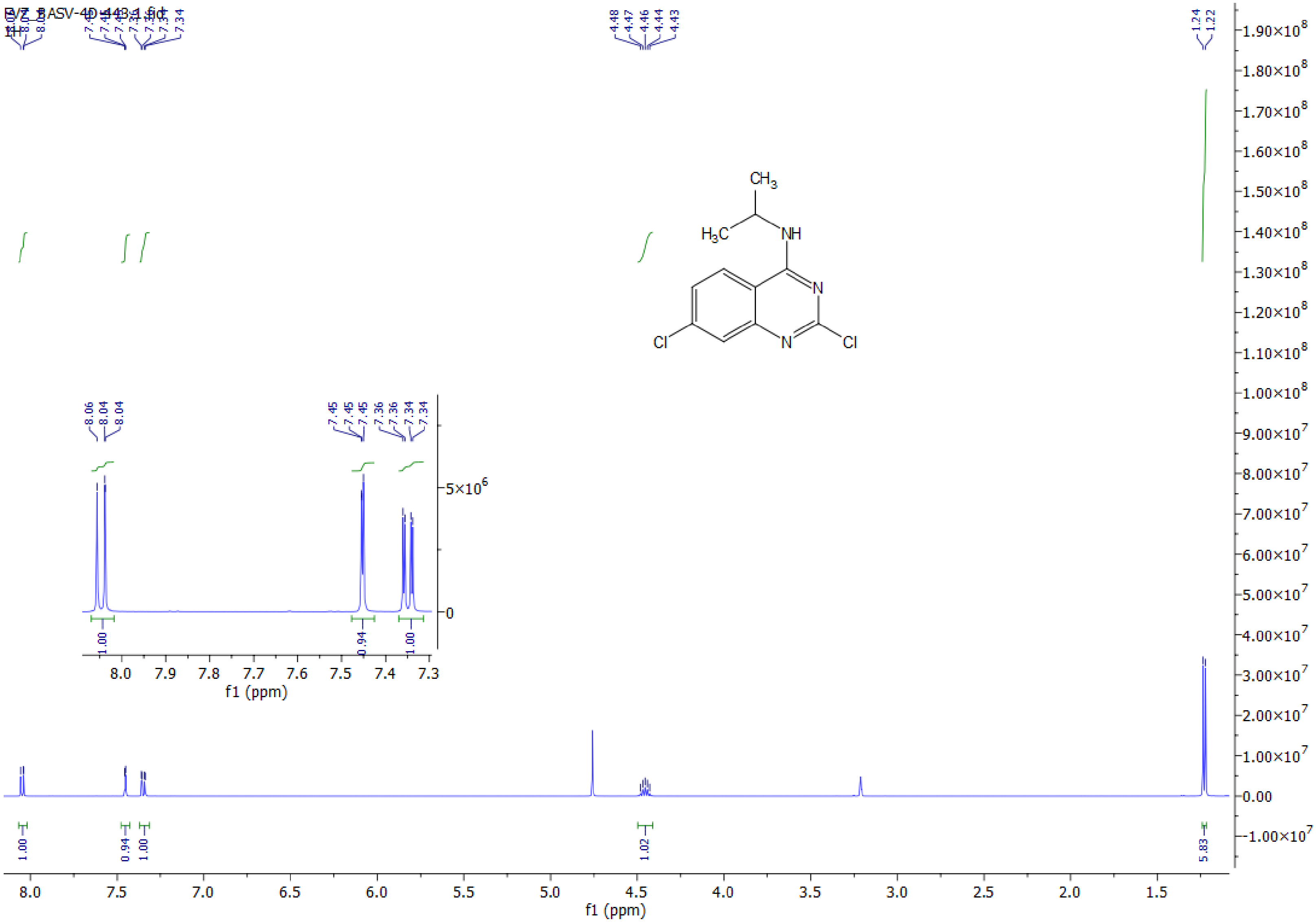

Fig. S21. ^{13}C NMR of 2,7-dichloro-*N*-(propan-2-yl)quinazolin-4-amine (II-5d)

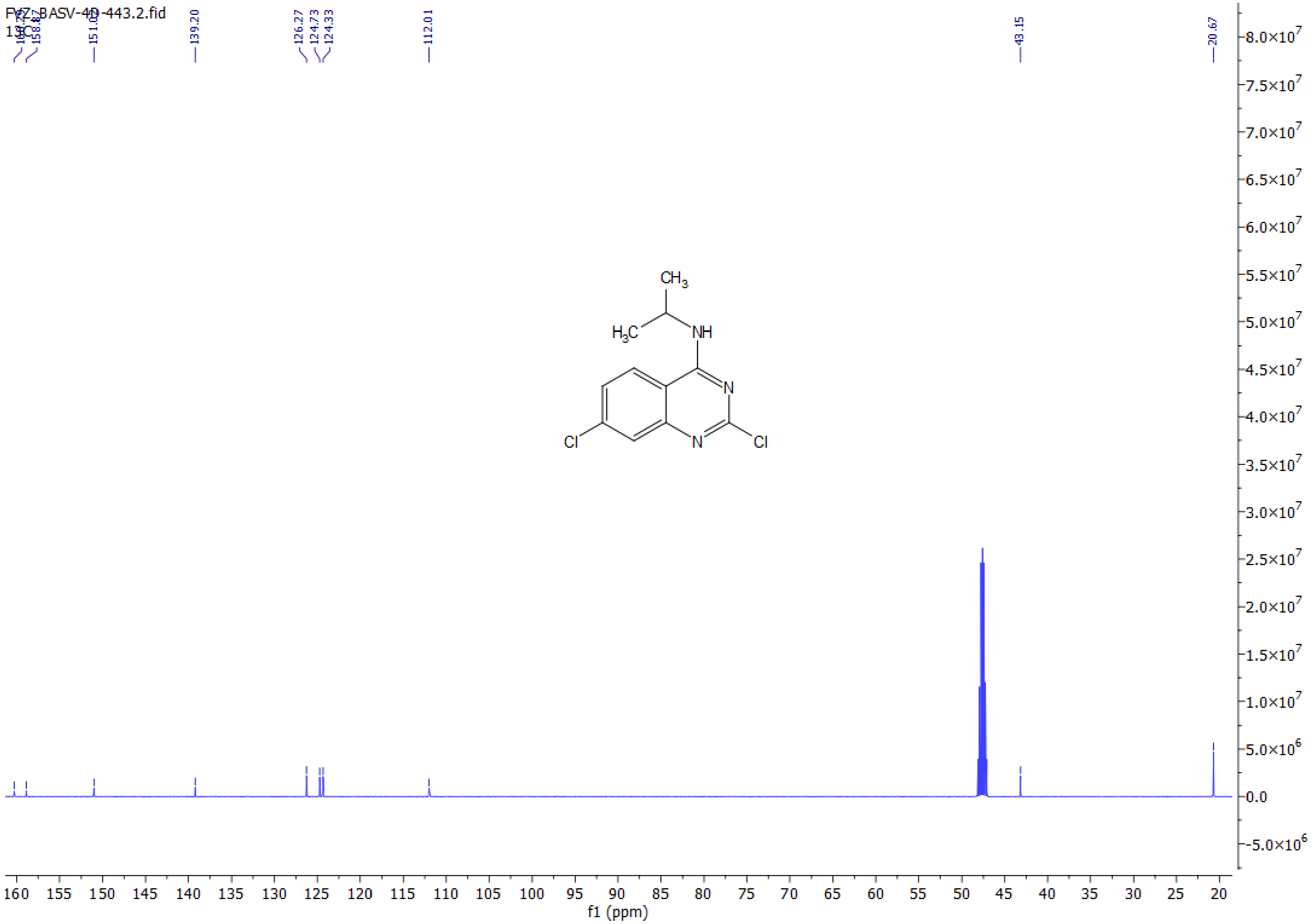

Fig. S22. ^{1}H NMR of *N*-butyl-2,7-dichloroquinazolin-4-amine (II-5e)

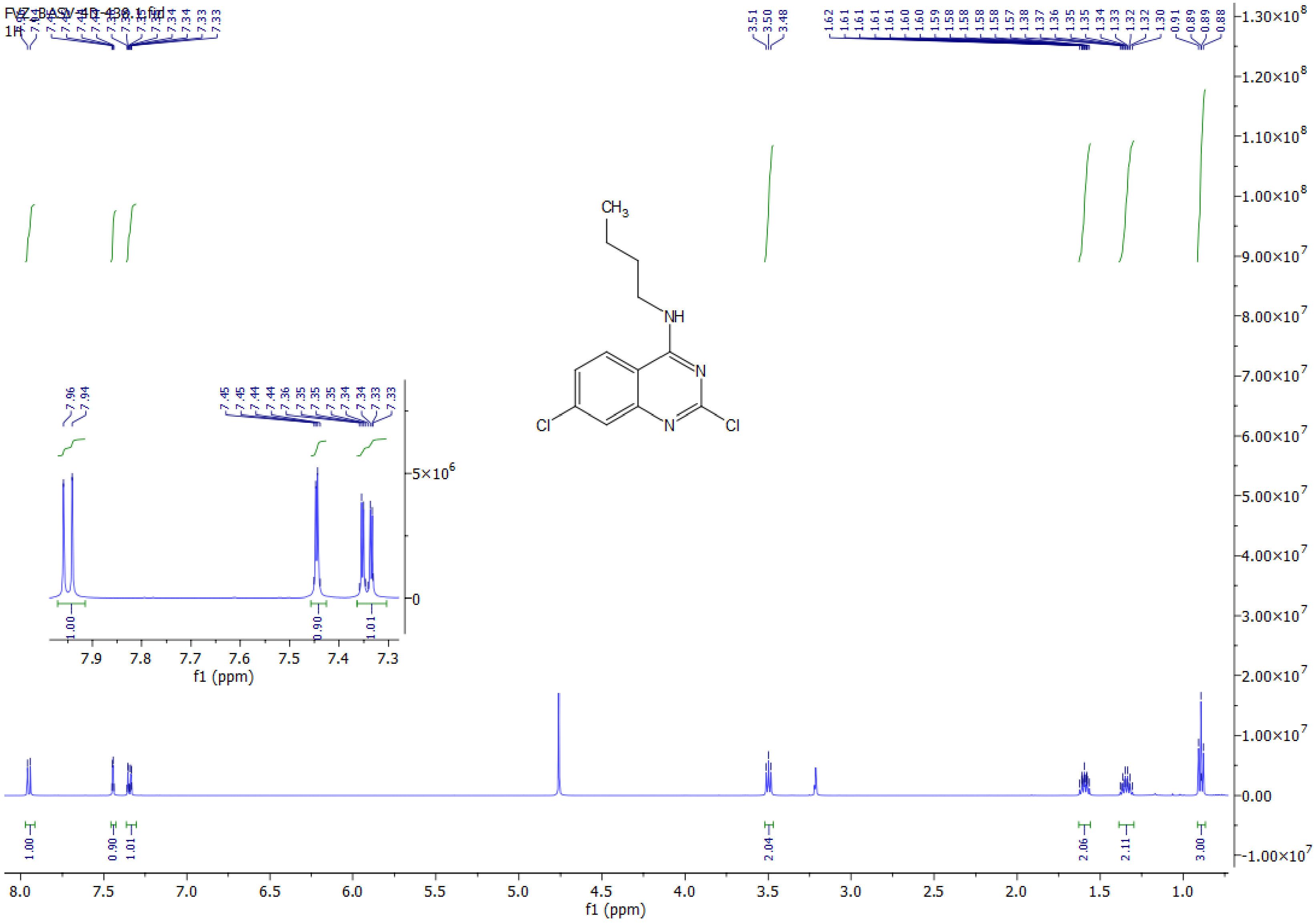

Fig. S23. ^{13}C NMR of *N*-butyl-2,7-dichloroquinazolin-4-amine (II-5e)

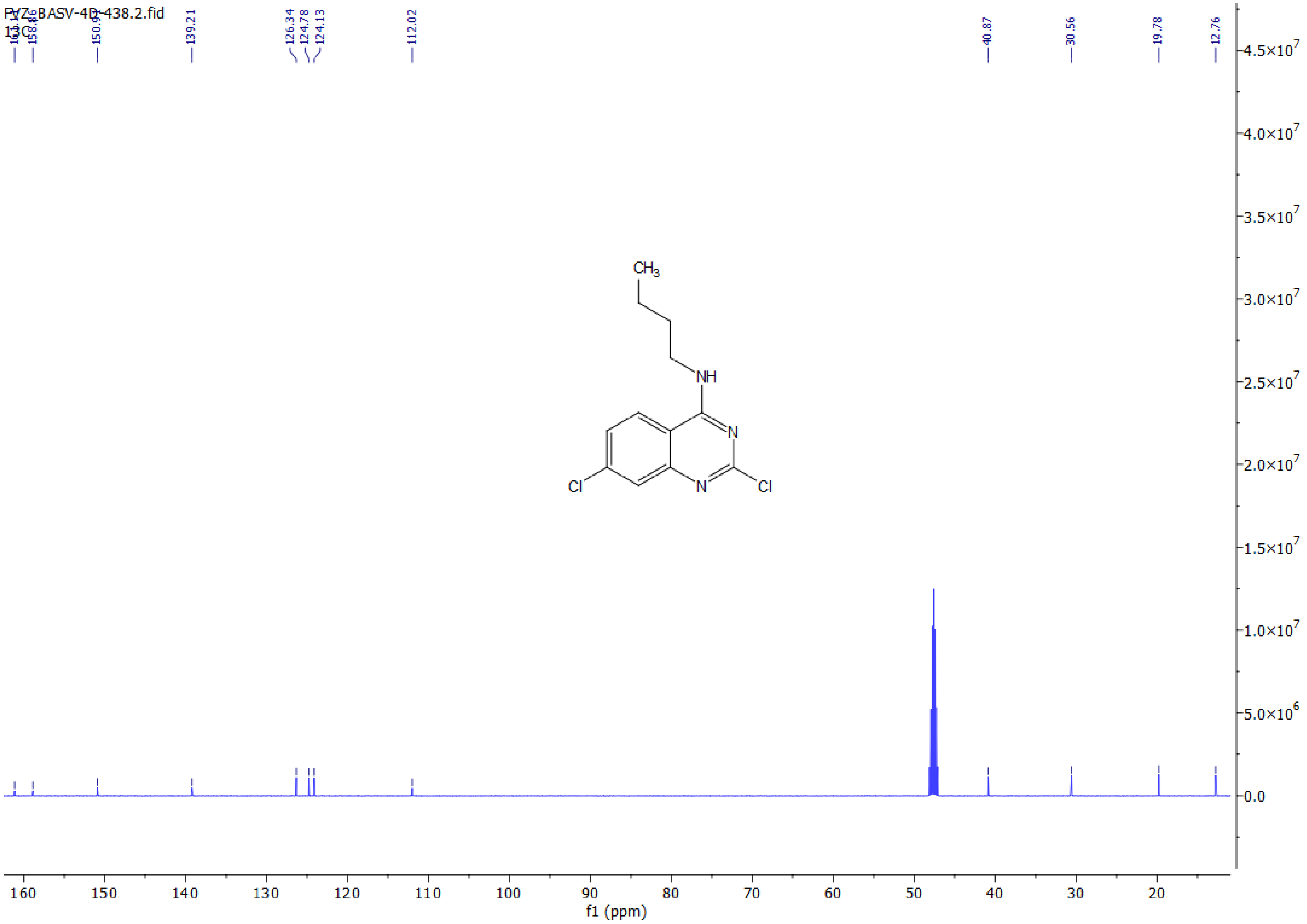

Fig. S24. ^{1}**H NMR of 2,7-dichloro-*N*-(2-methoxyethyl)quinazolin-4-amine (II-5f)**

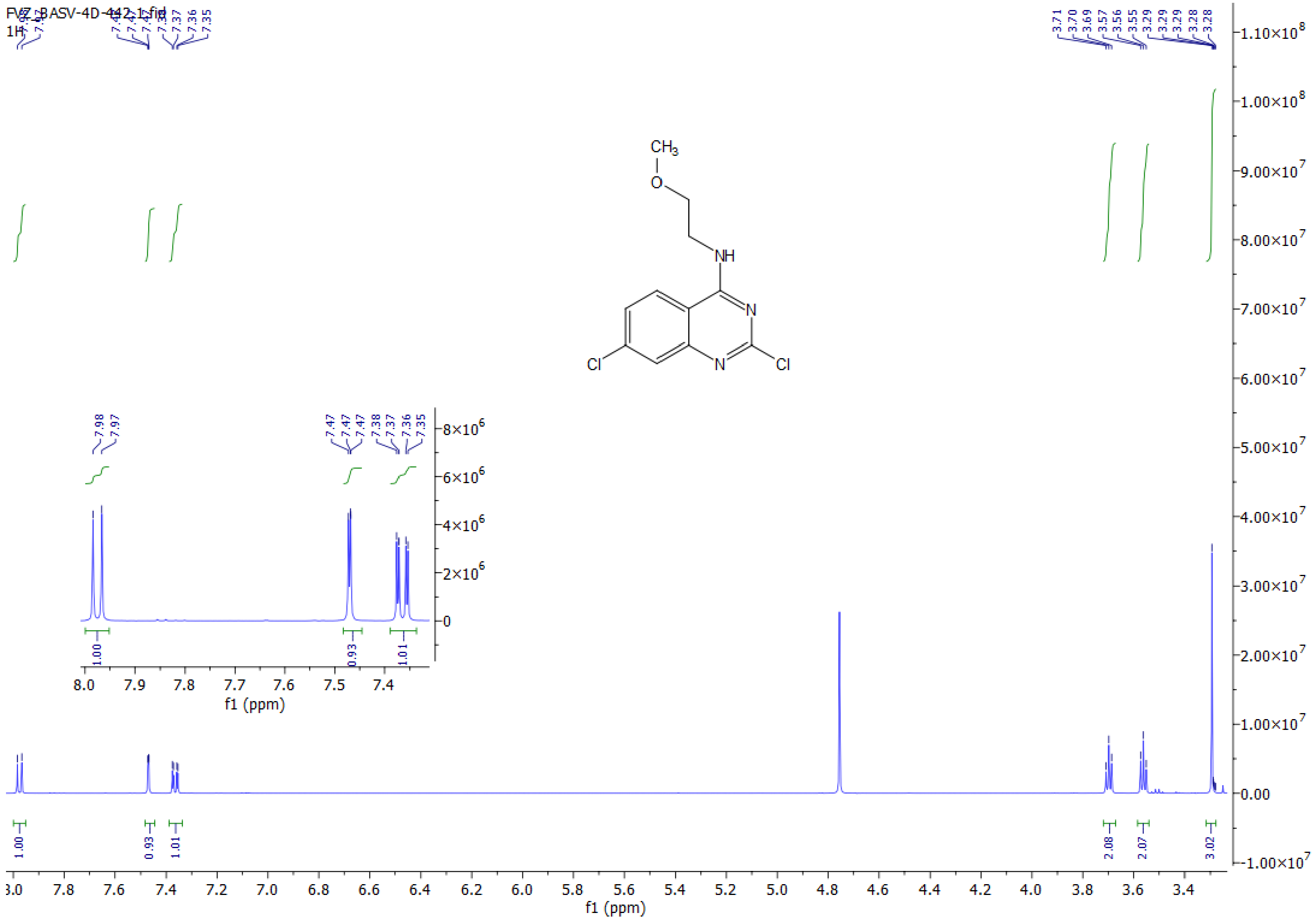

Fig. S25. ^{13}C NMR of 2,7-dichloro-*N*-(2-methoxyethyl)quinazolin-4-amine (II-5f)

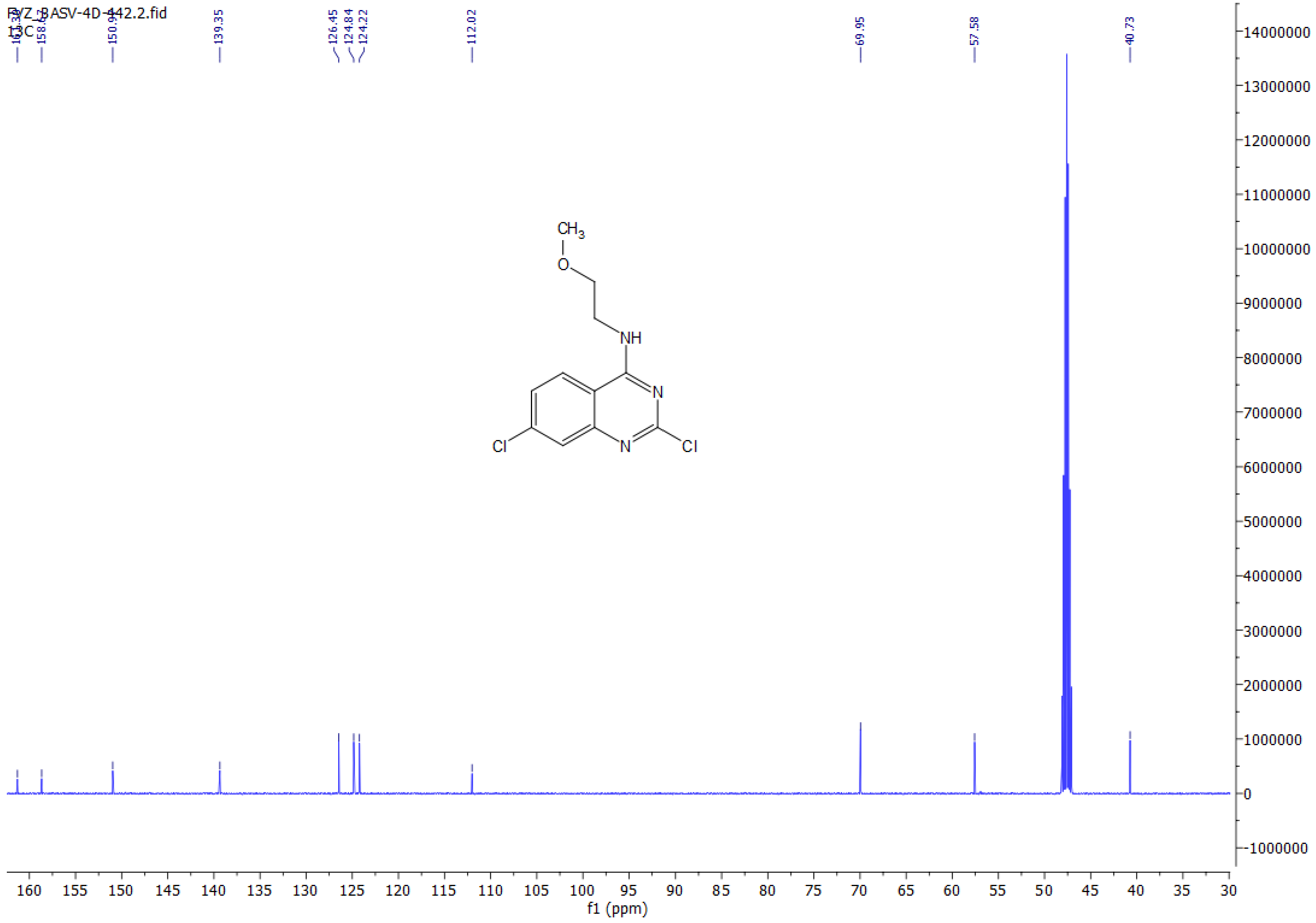

Fig. S26. ^{1}H NMR of 2,7-dichloro-*N*-cyclohexylquinazolin-4-amine (II-5h)

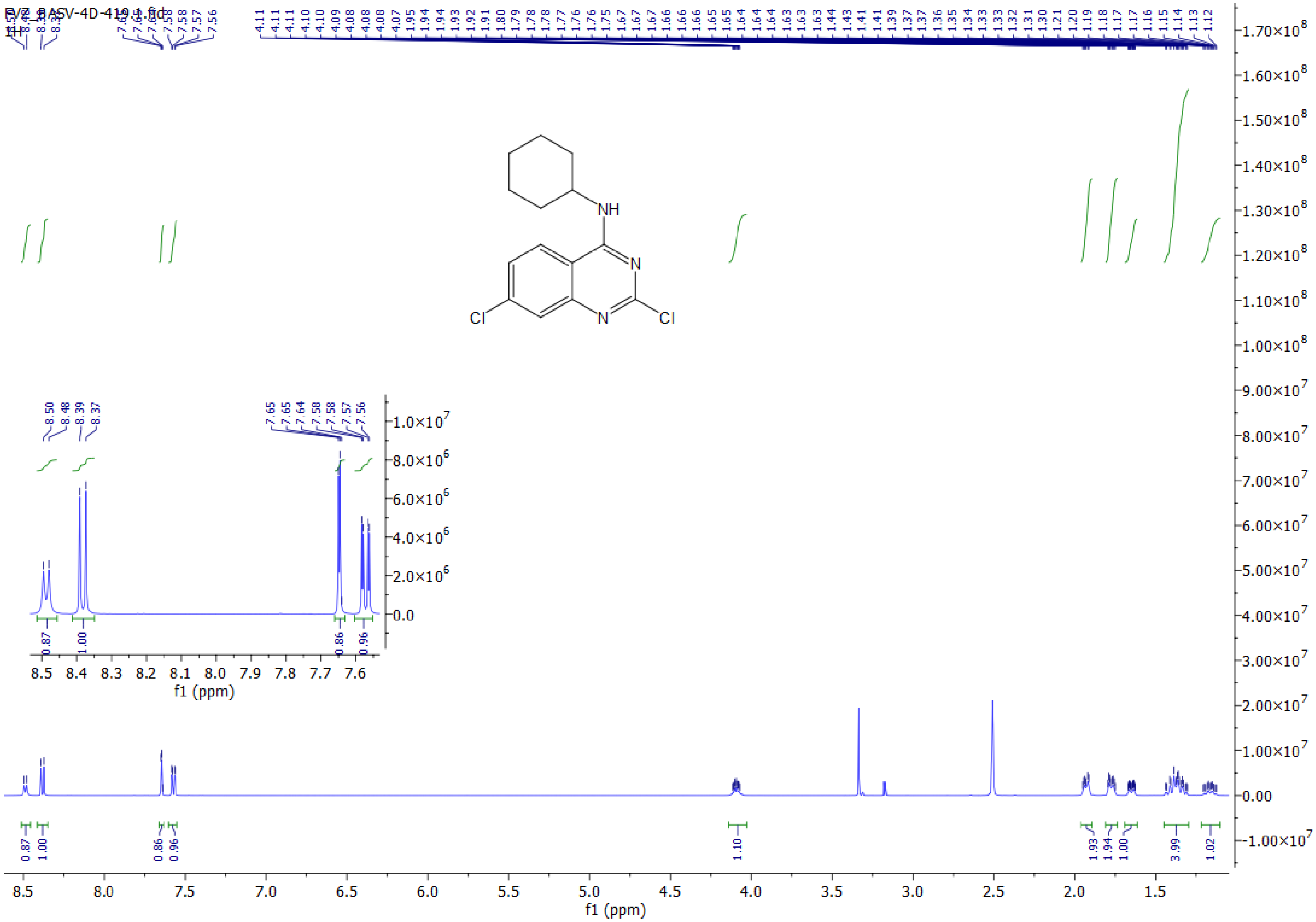

Fig. S27. ^{13}C NMR of 2,7-dichloro-*N*-cyclohexylquinazolin-4-amine (II-5h)

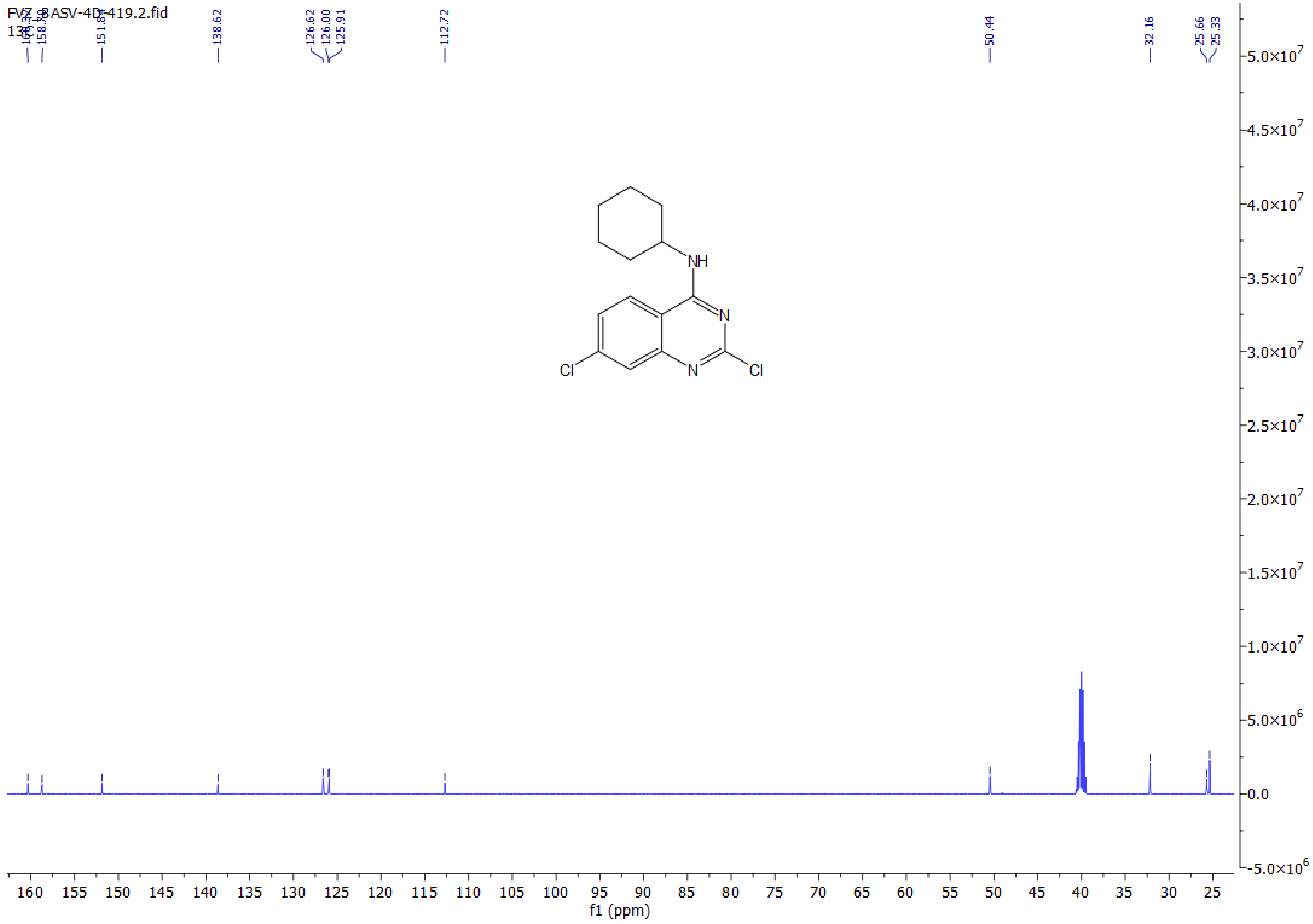

Fig. S28. ¹H NMR of 2-chloro-6-methoxyquinazolin-4-amine (III-5a)

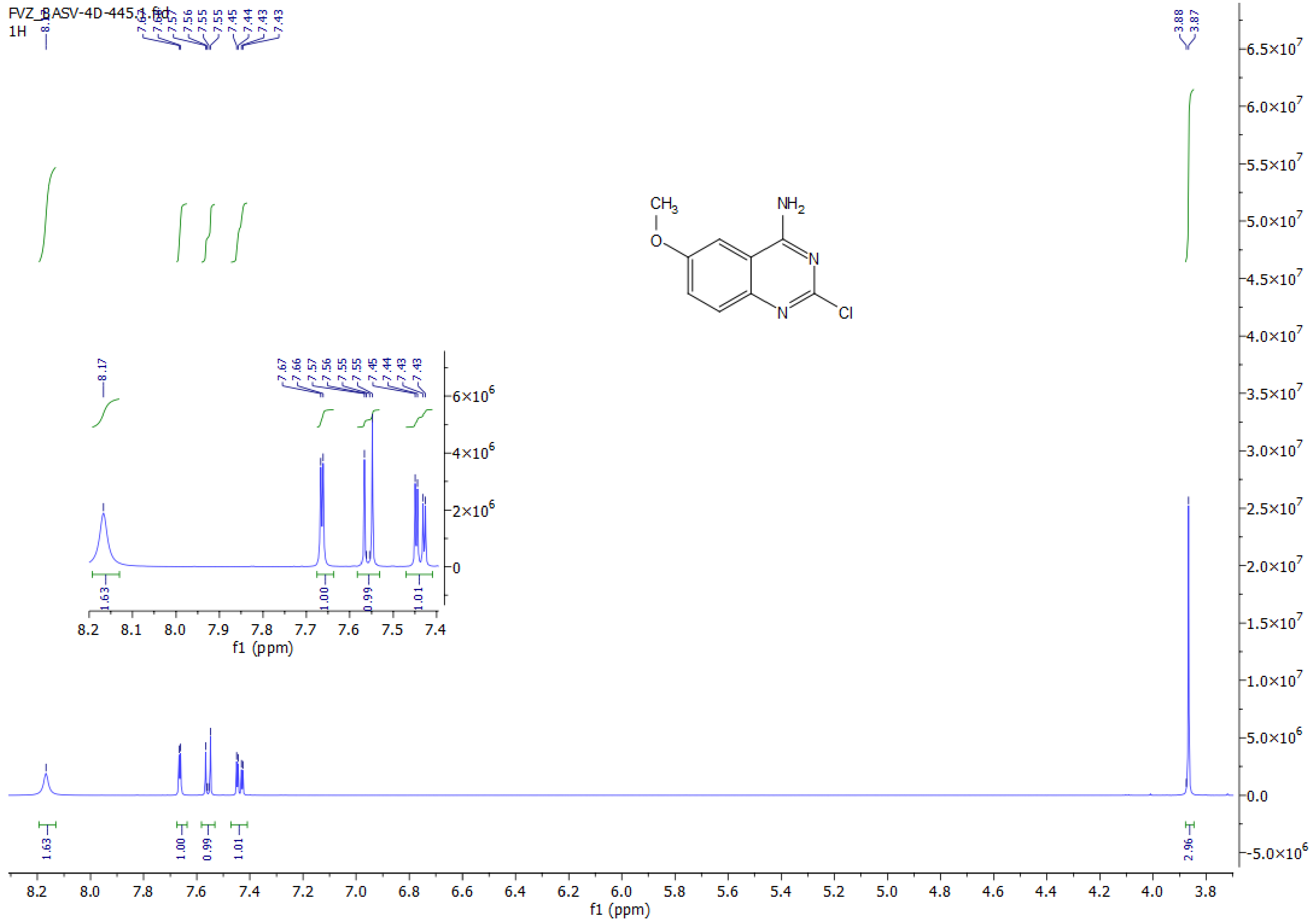

Fig. S29. ^{13}C NMR of 2-chloro-6-methoxyquinazolin-4-amine (III-5a)

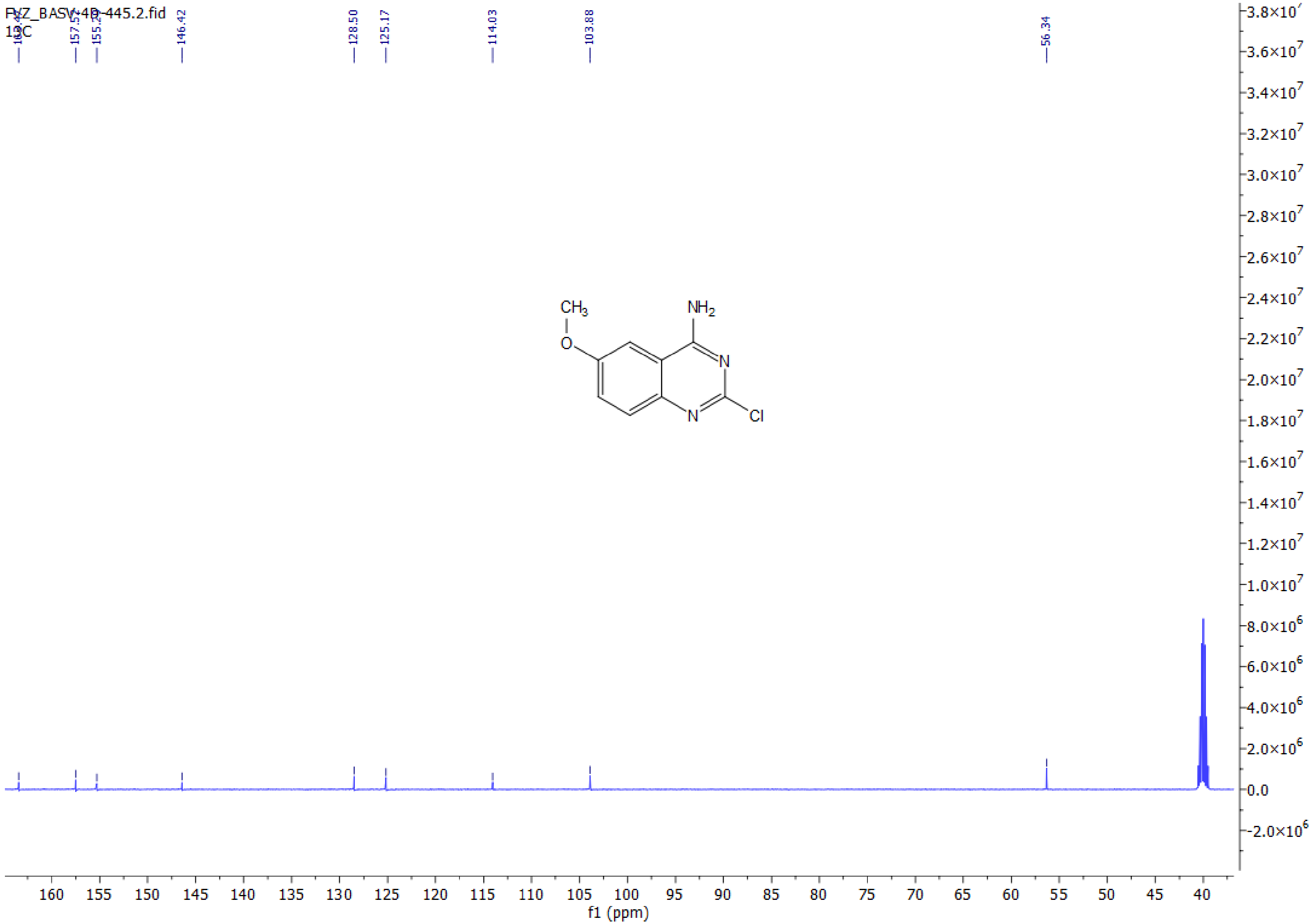

Fig. S30. ^{1}H NMR of 2-chloro-6-methoxy-*N*-methylquinazolin-4-amine (III-5b)

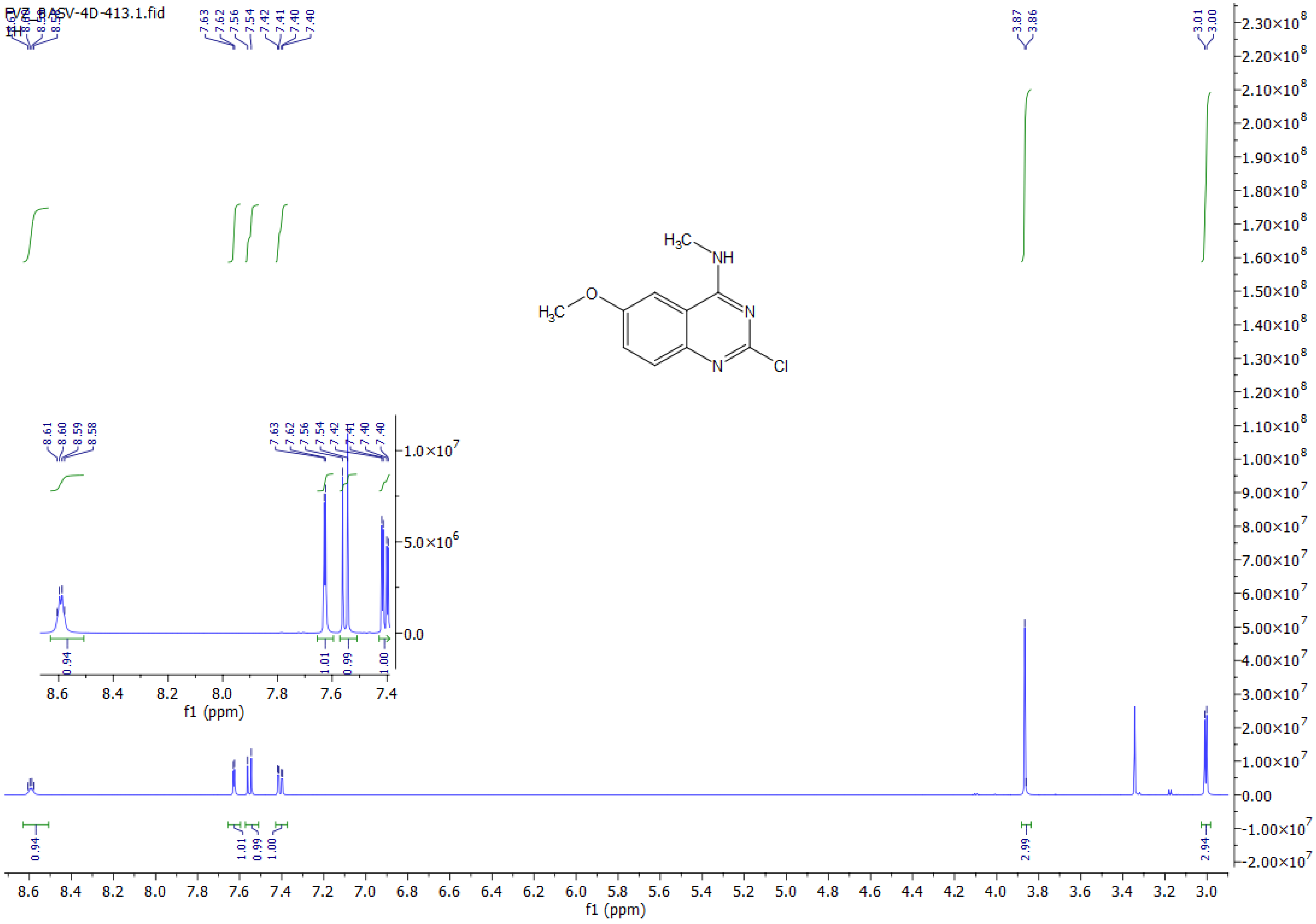

Fig. S31. ^{13}C NMR of 2-chloro-6-methoxy-*N*-methylquinazolin-4-amine (III-5b)

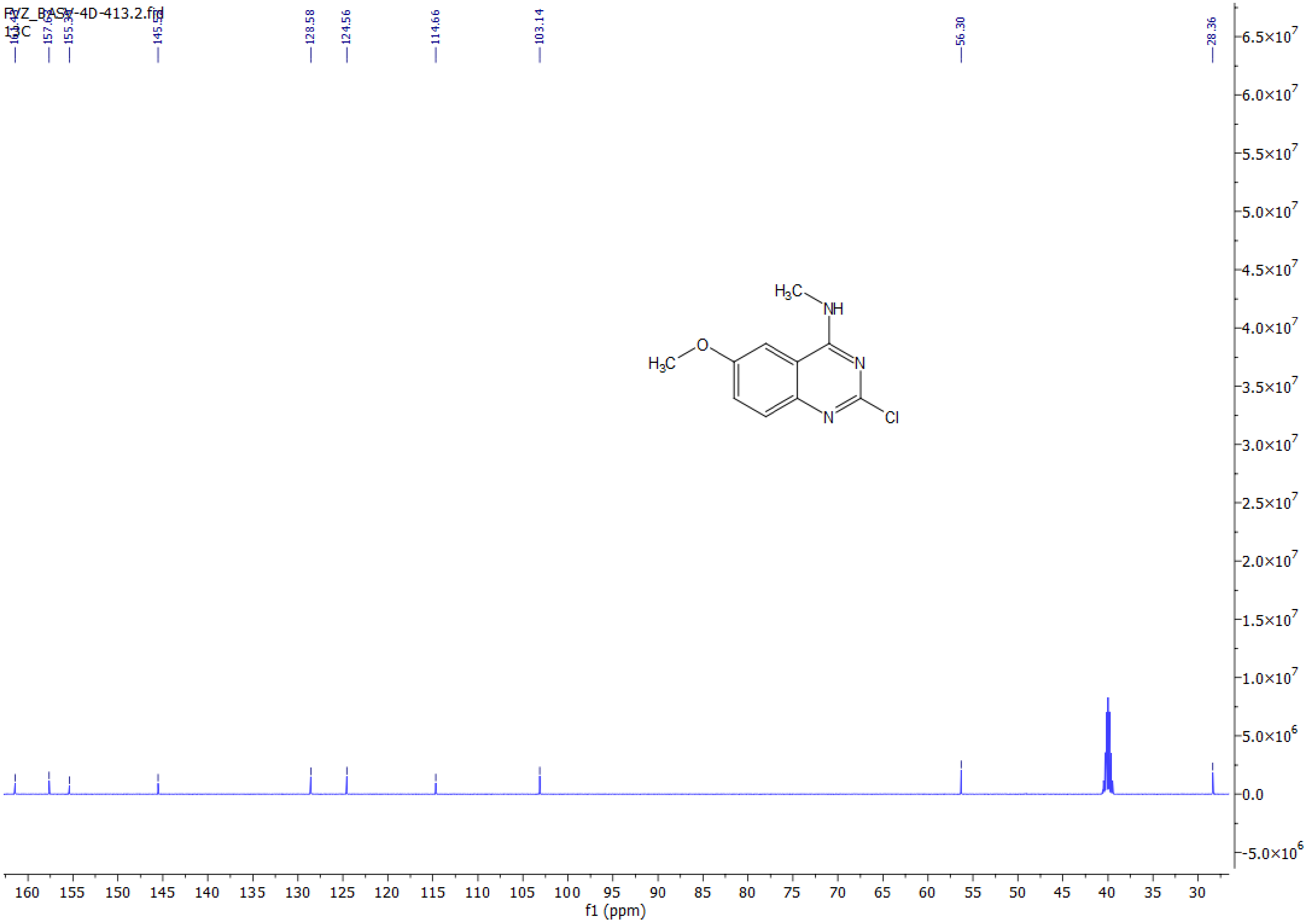

Fig. S32. ^{1}H NMR of 2-chloro-*N*-ethyl-6-methoxyquinazolin-4-amine (III-5c)

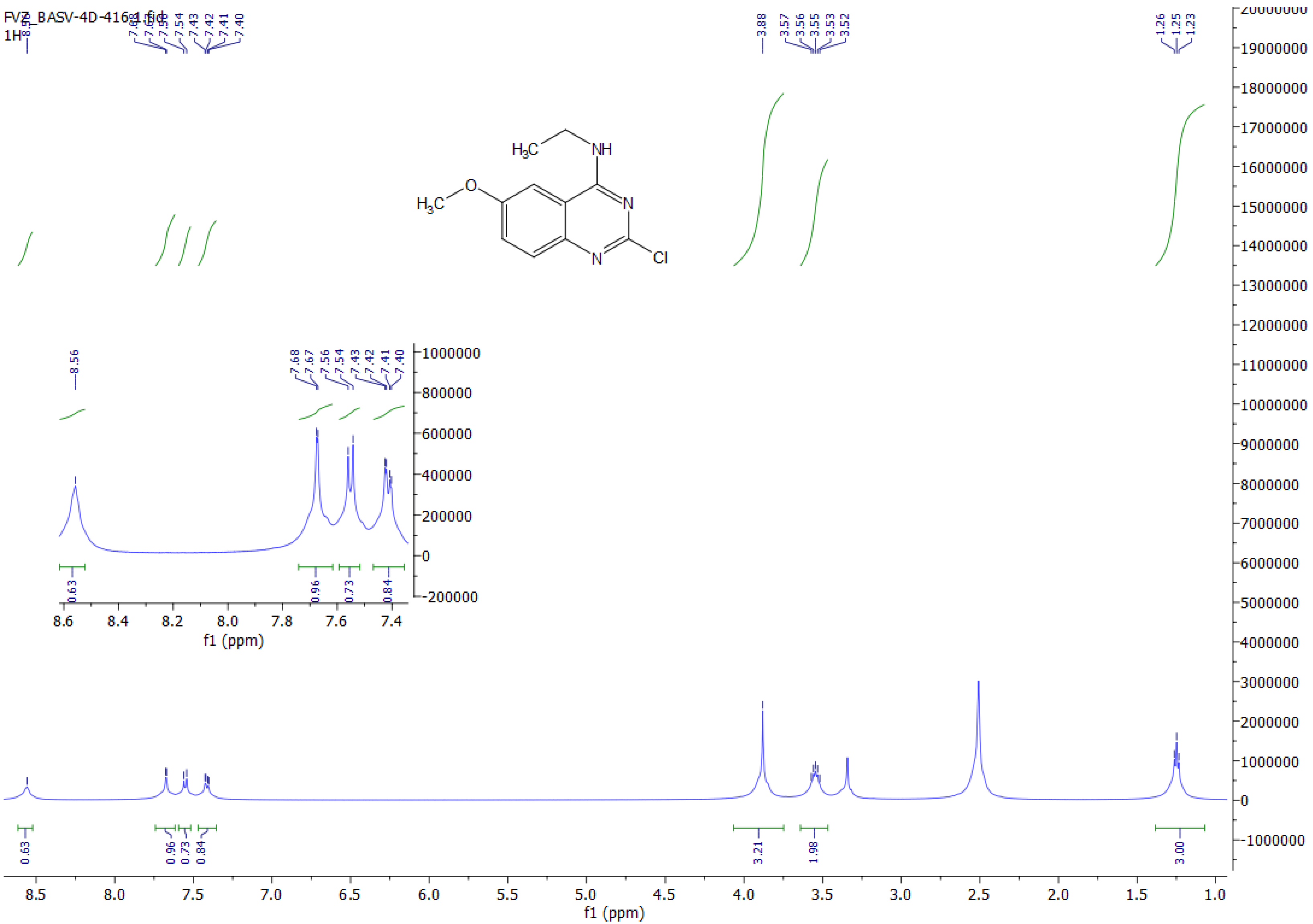

Fig. S33. ^{13}C NMR of 2-chloro-*N*-ethyl-6-methoxyquinazolin-4-amine (III-5c)

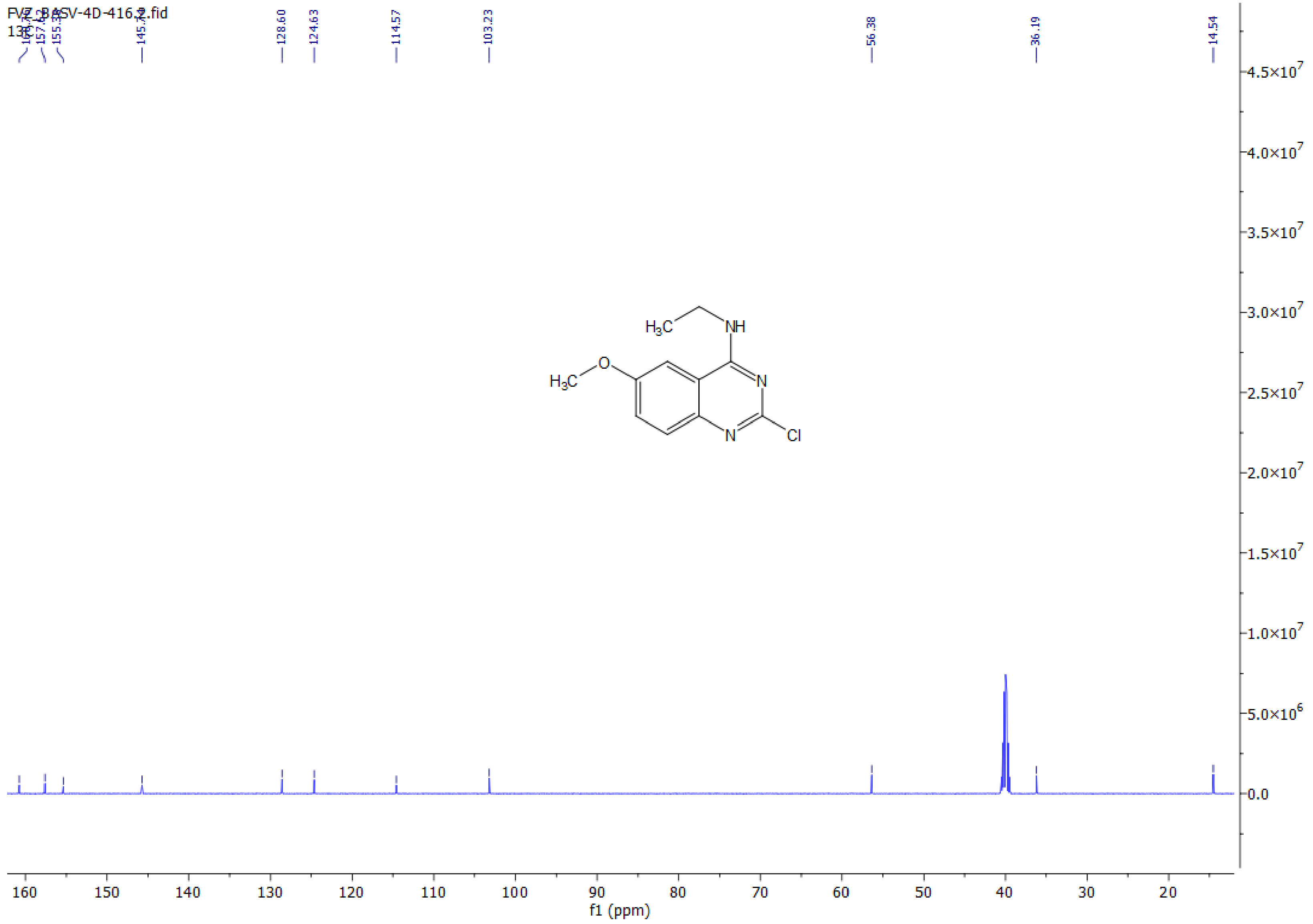

Fig. S34. ^{1}H NMR of 2-chloro-6-methoxy-*N*-(propan-2-yl)quinazolin-4-amine (III-5d)

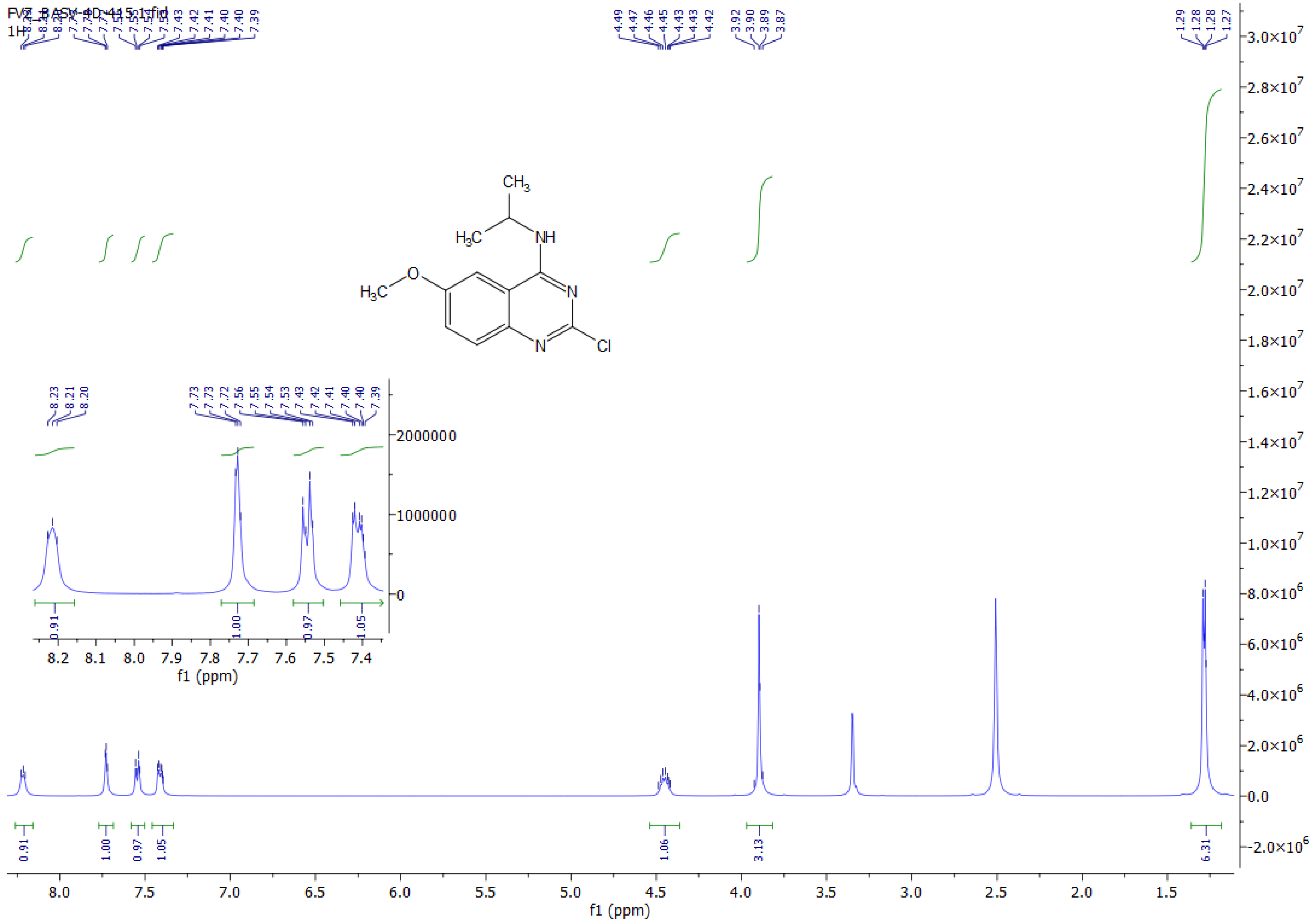

Fig. S35. ^{13}C NMR of 2-chloro-6-methoxy-*N*-(propan-2-yl)quinazolin-4-amine (III-5d)

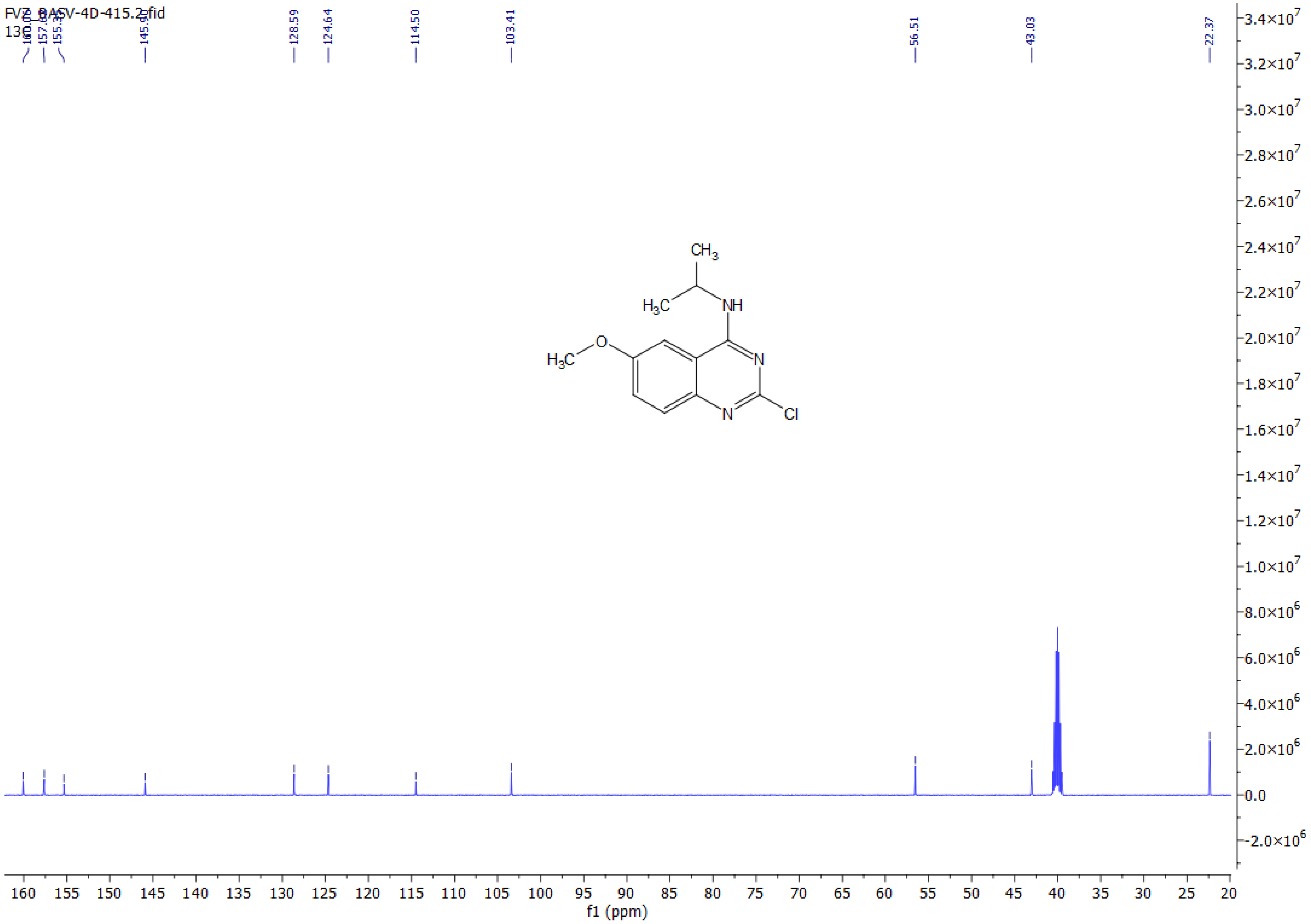

Fig. S36. ^{1}H NMR of *N*-butyl-2-chloro-6-methoxyquinazolin-4-amine (III-5e)

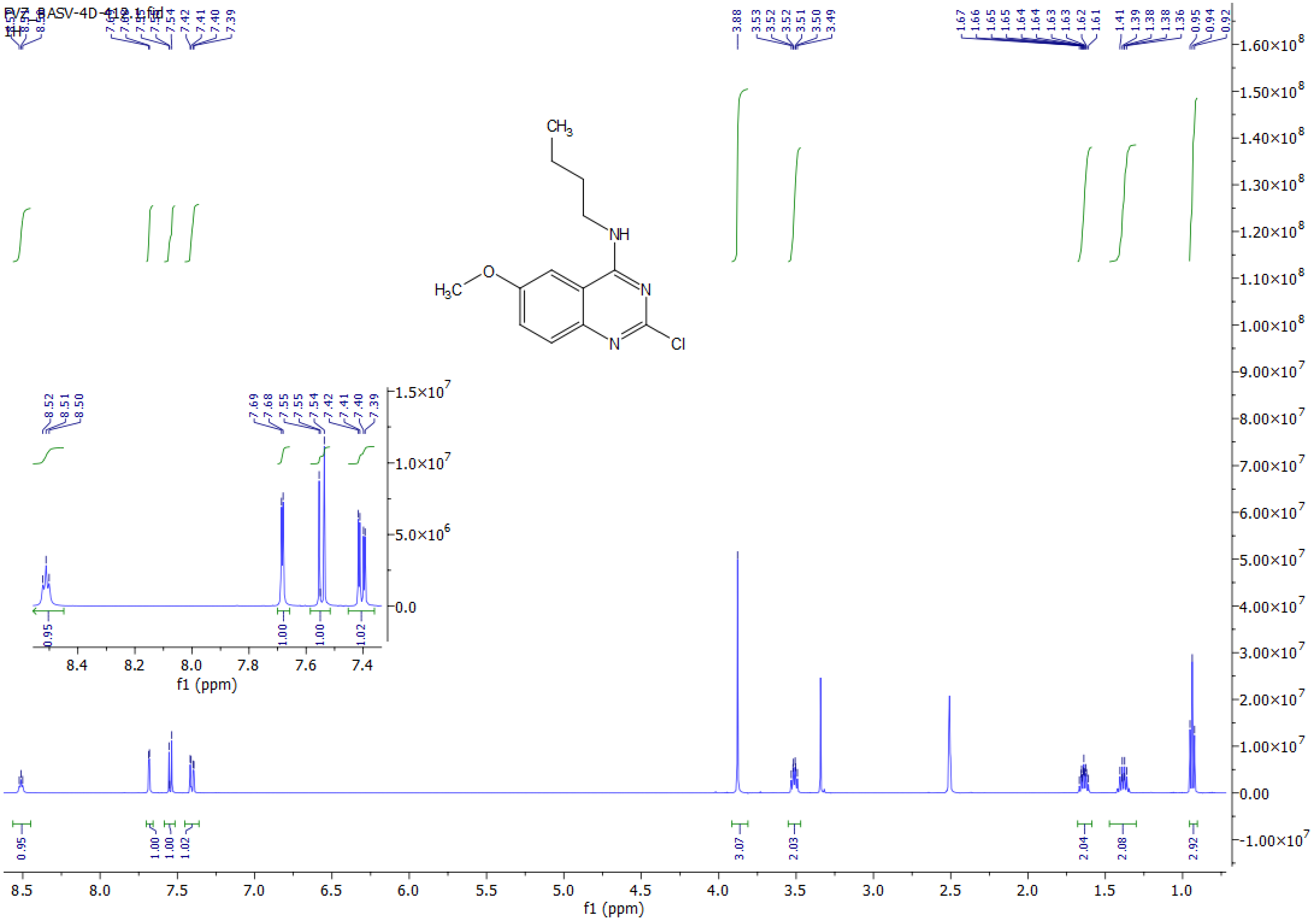

Fig. S37. ^{13}C NMR of *N*-butyl-2-chloro-6-methoxyquinazolin-4-amine (III-5e)

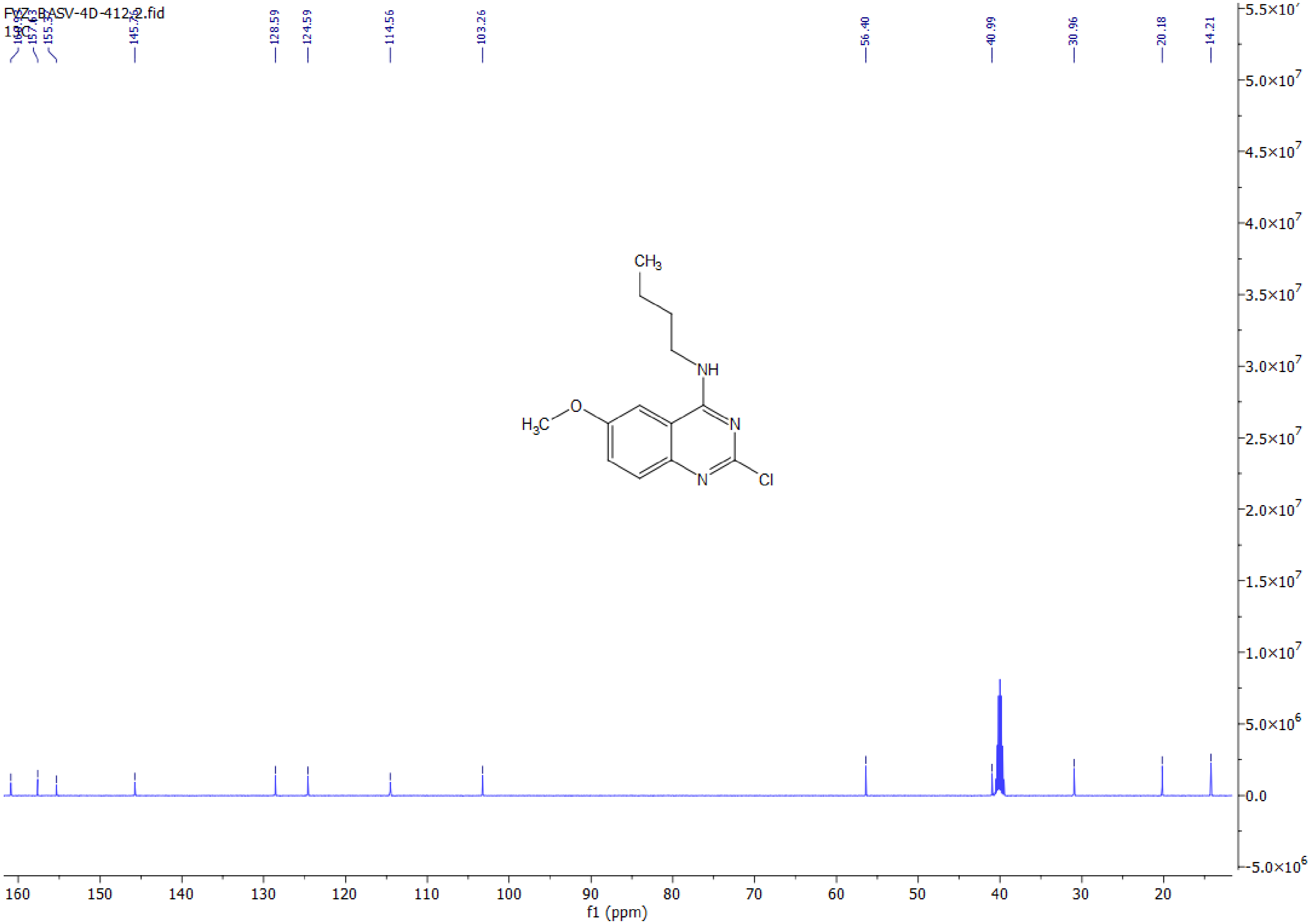

Fig. S38. ^{1}**H NMR of 2-chloro-*N*-cyclopropyl-6-methoxyquinazolin-4-amine (III-5g)**

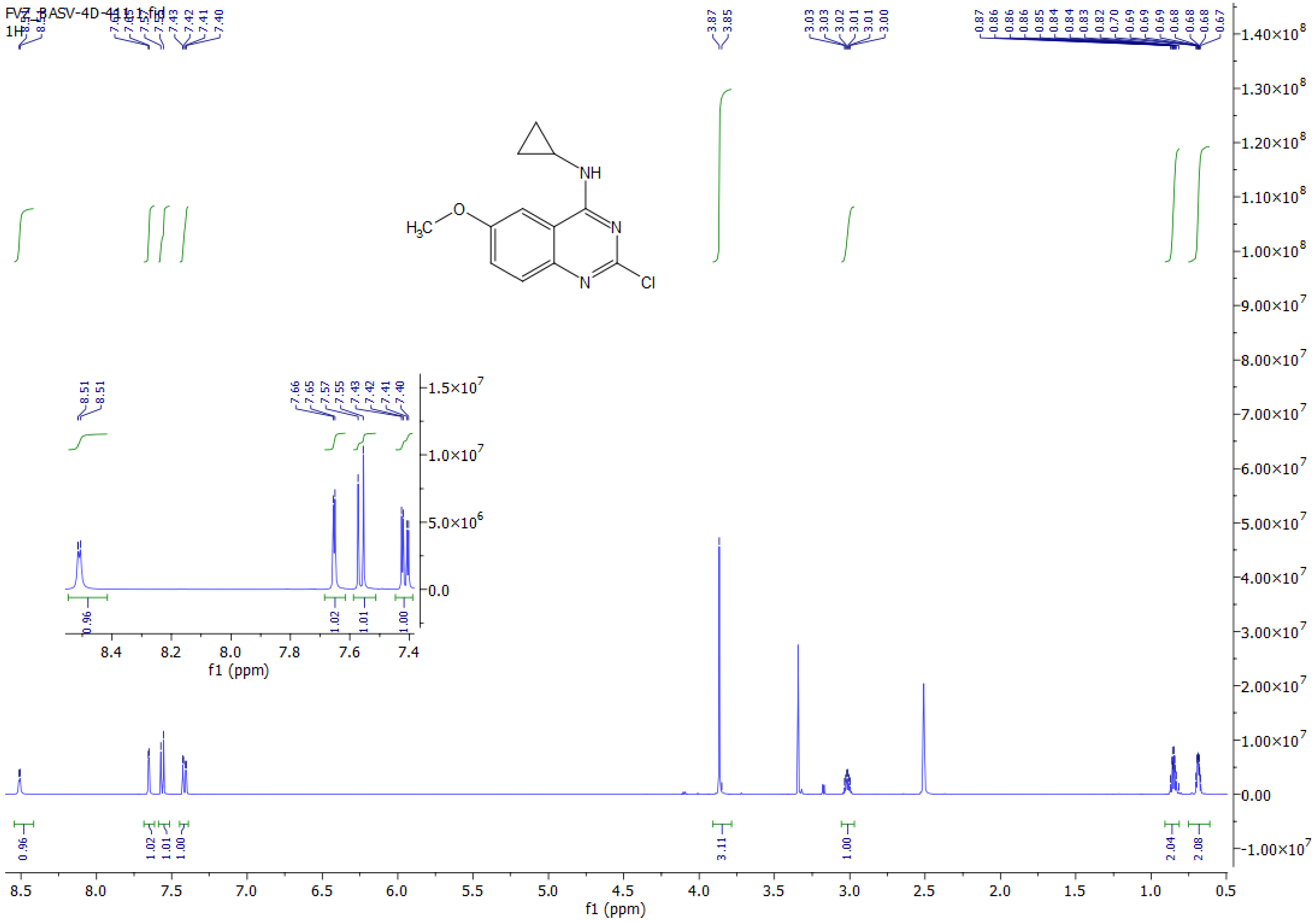

Fig. S39. ^{13}C NMR of 2-chloro-*N*-cyclopropyl-6-methoxyquinazolin-4-amine (III-5g)

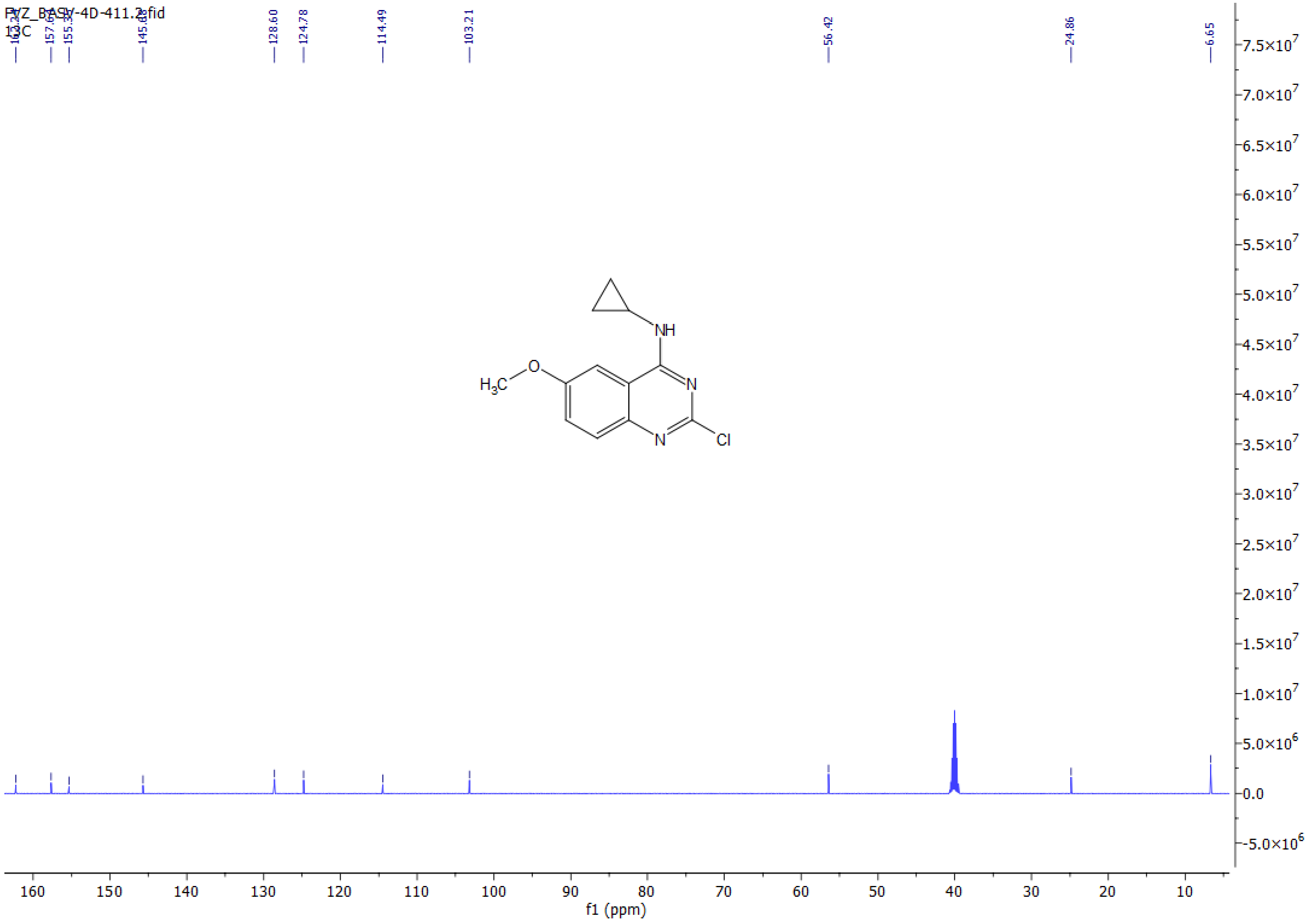

Fig. S40. ^{1}H NMR of 2-chloro-*N*-cyclohexyl-6-methoxyquinazolin-4-amine (III-5h)

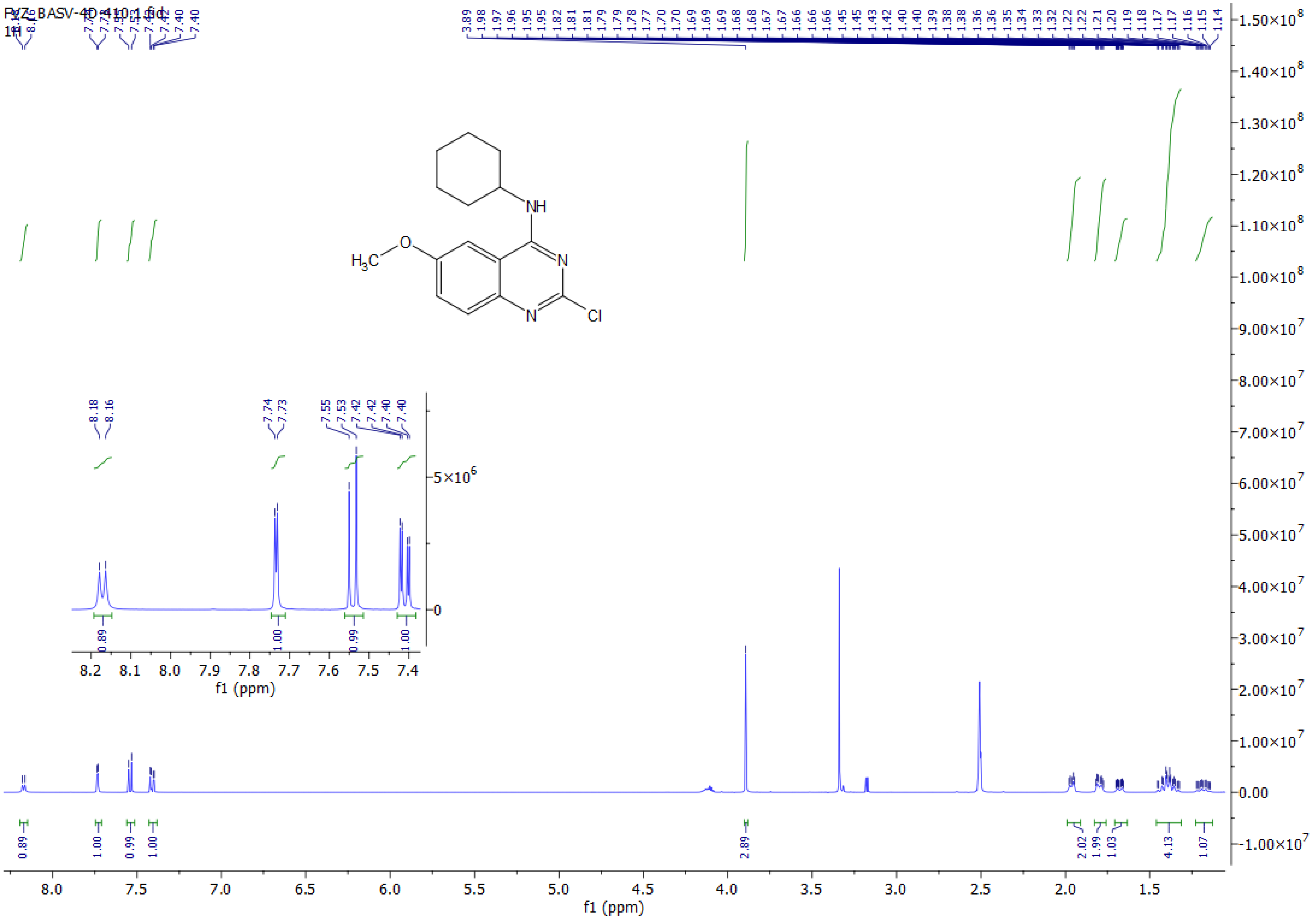

Fig. S41. ^{13}C NMR of 2-chloro-*N*-cyclohexyl-6-methoxyquinazolin-4-amine (III-5h)

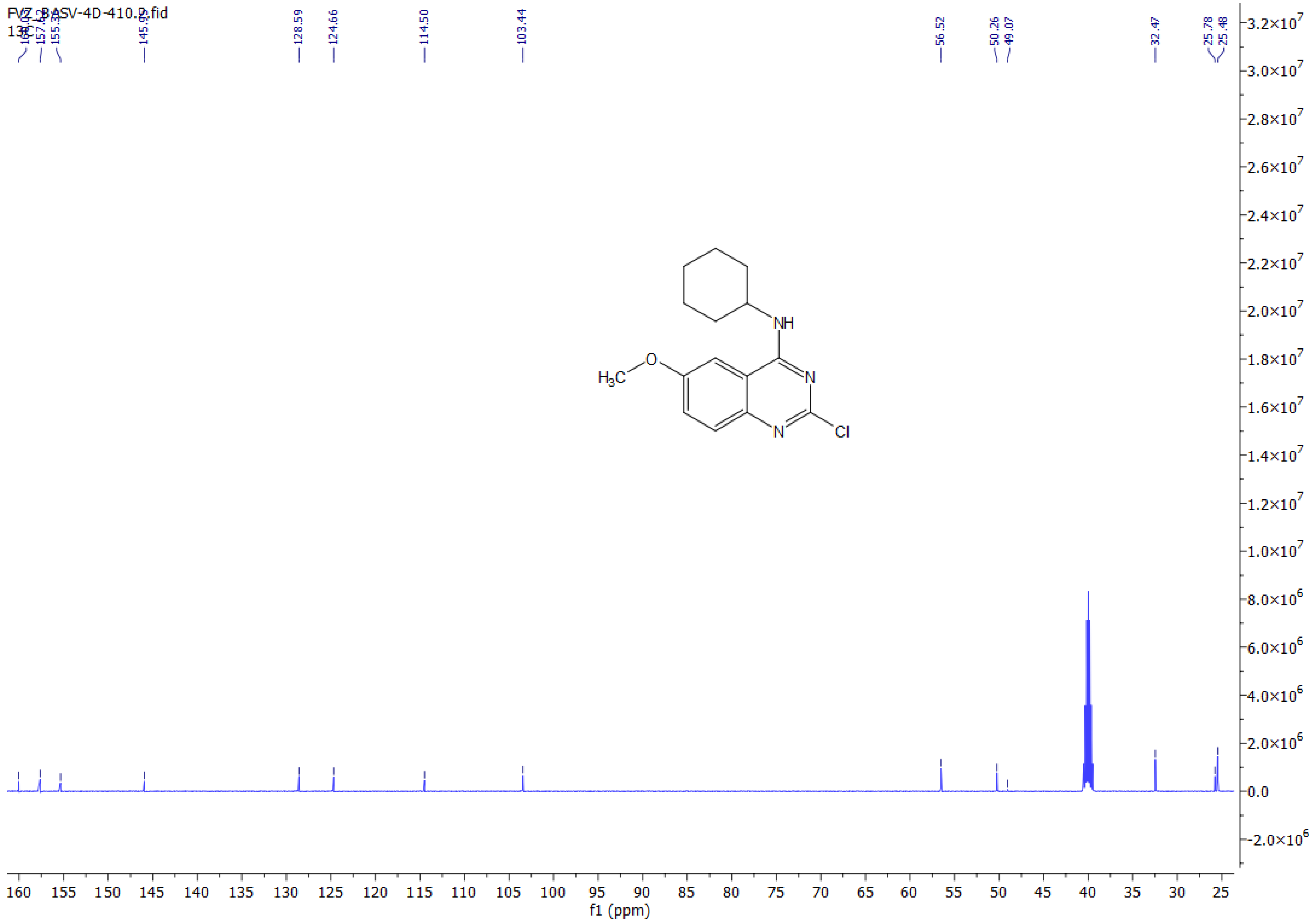

Fig. S42. ^{1}H NMR of ***N2*-methyl-*N2*-(prop-2-yn-1-yl)quinazoline-2,4-diamine (I-6a)**

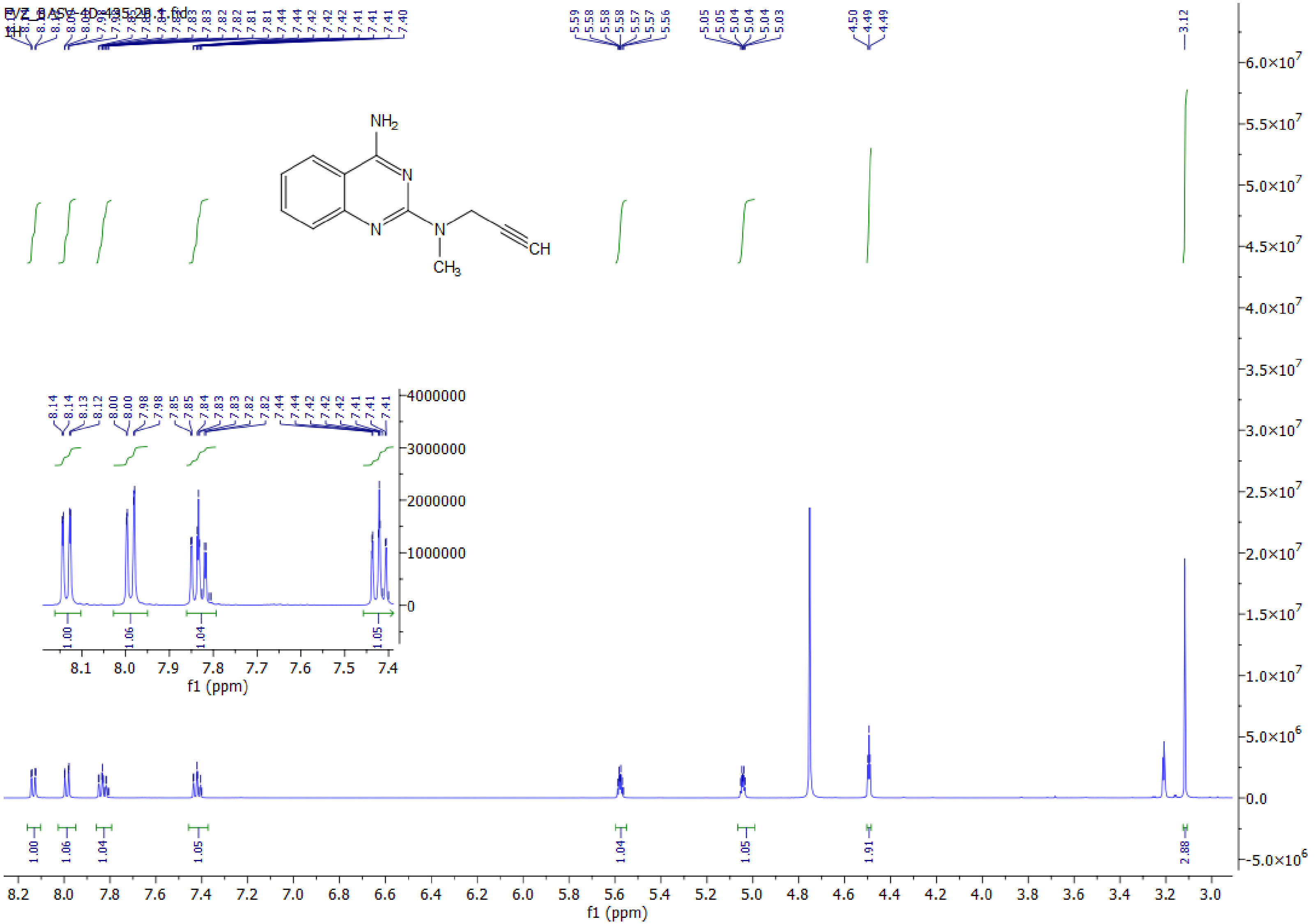

Fig. S43. ^{13}C NMR of *N2*-methyl-*N2*-(prop-2-yn-1-yl)quinazoline-2,4-diamine (I-6a)

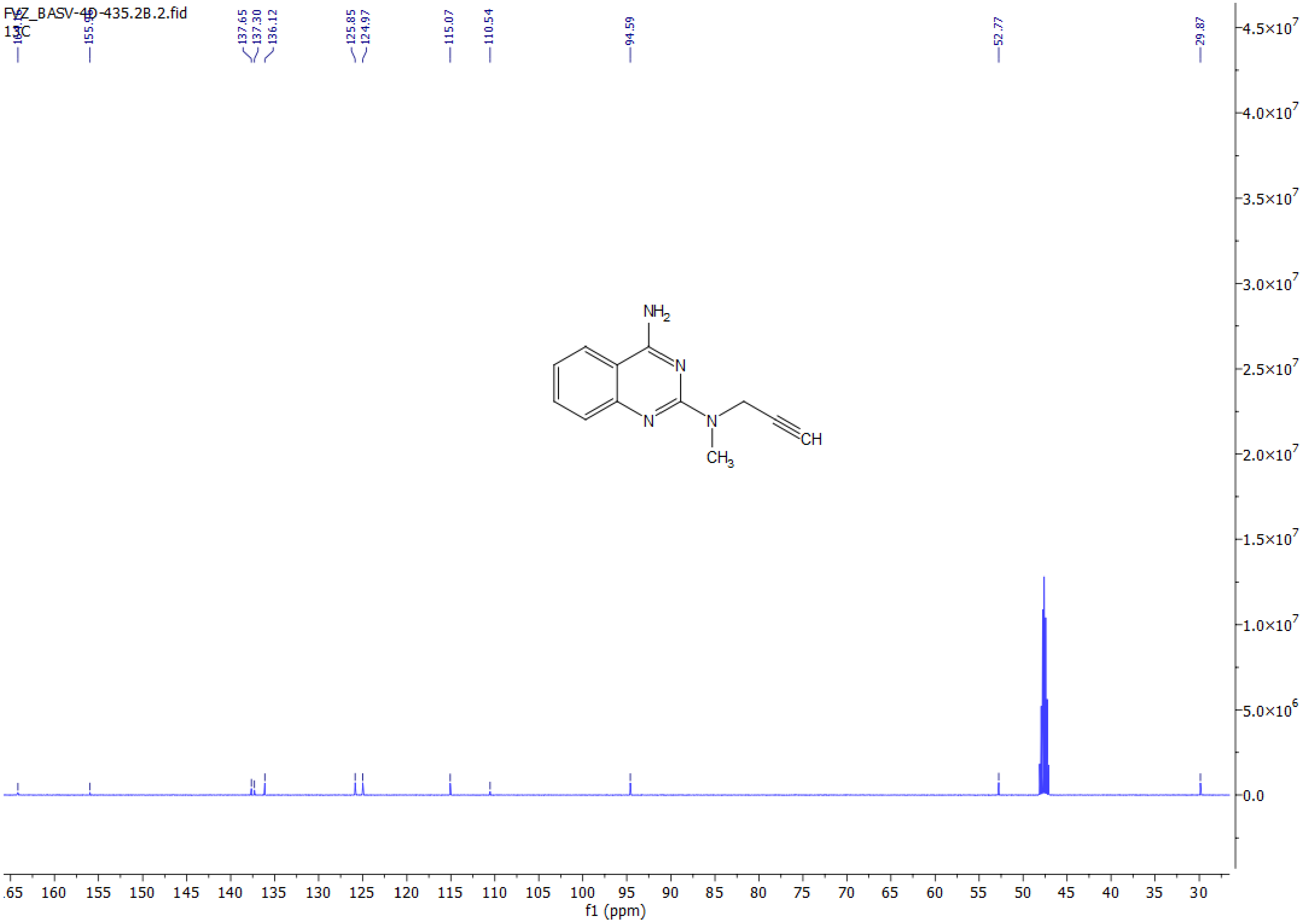

Fig. S44. ^{1}H NMR of *N2*,*N4*-dimethyl-*N2*-(prop-2-yn-1-yl)quinazoline-2,4-diamine (I-6b)

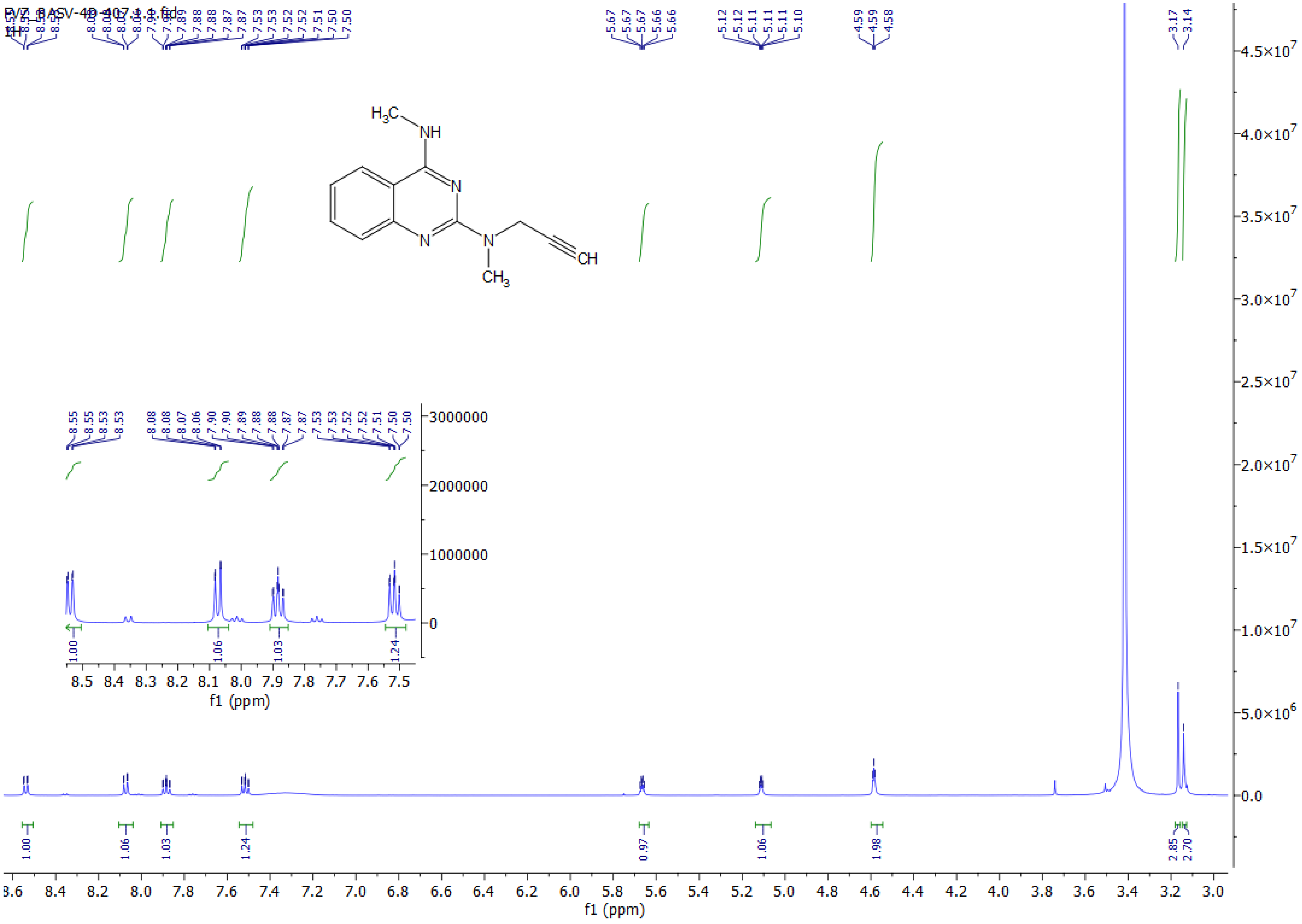

Fig. S45. ^{13}**C NMR of *N2*,*N4*-dimethyl-*N2*-(prop-2-yn-1-yl)quinazoline-2,4-diamine (I-6b)**

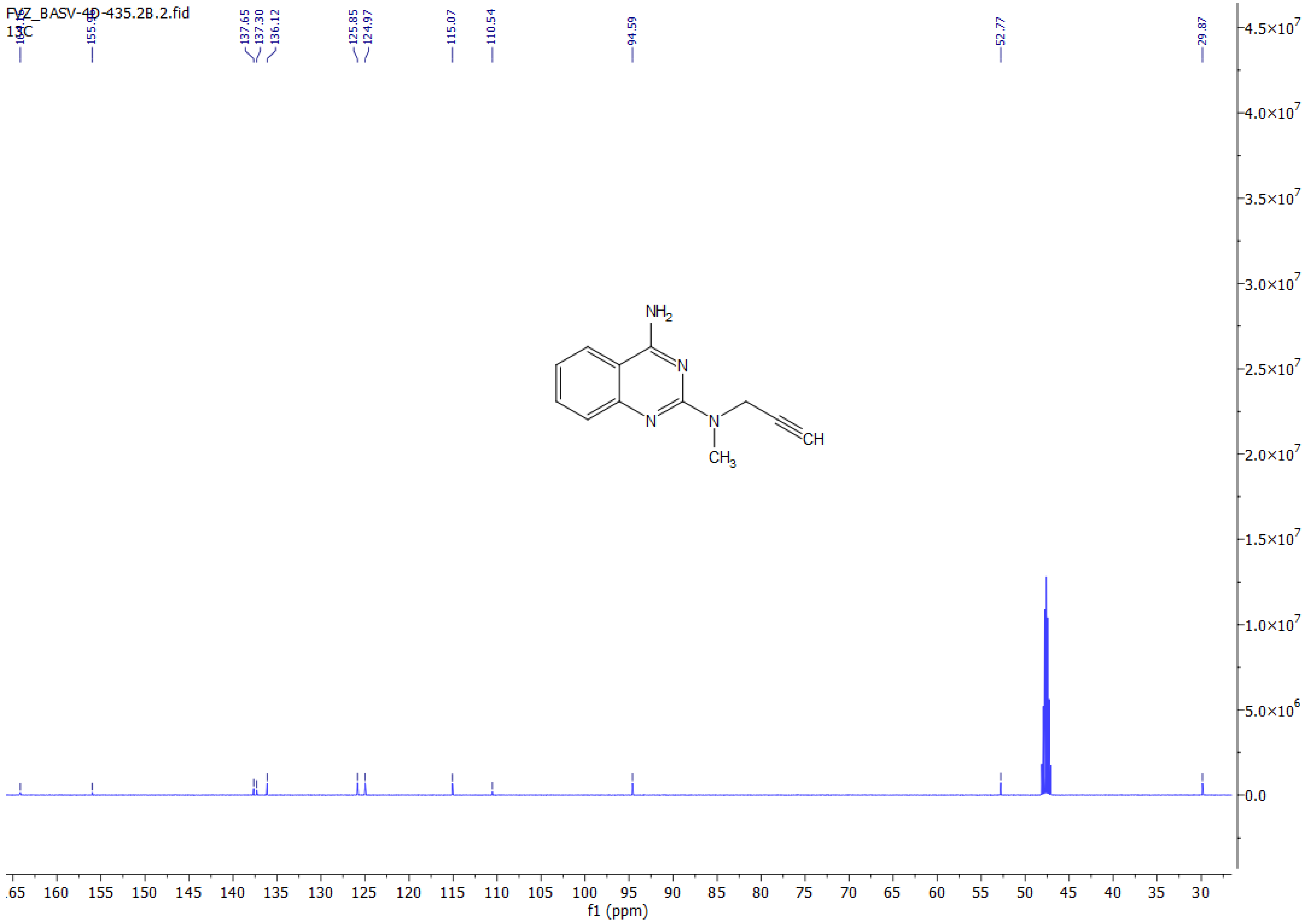

Fig. S46. ^{1}H NMR of *N4*-ethyl-*N2*-methyl-*N2*-(prop-2-yn-1-yl)quinazoline-2,4-diamine (I-6c)

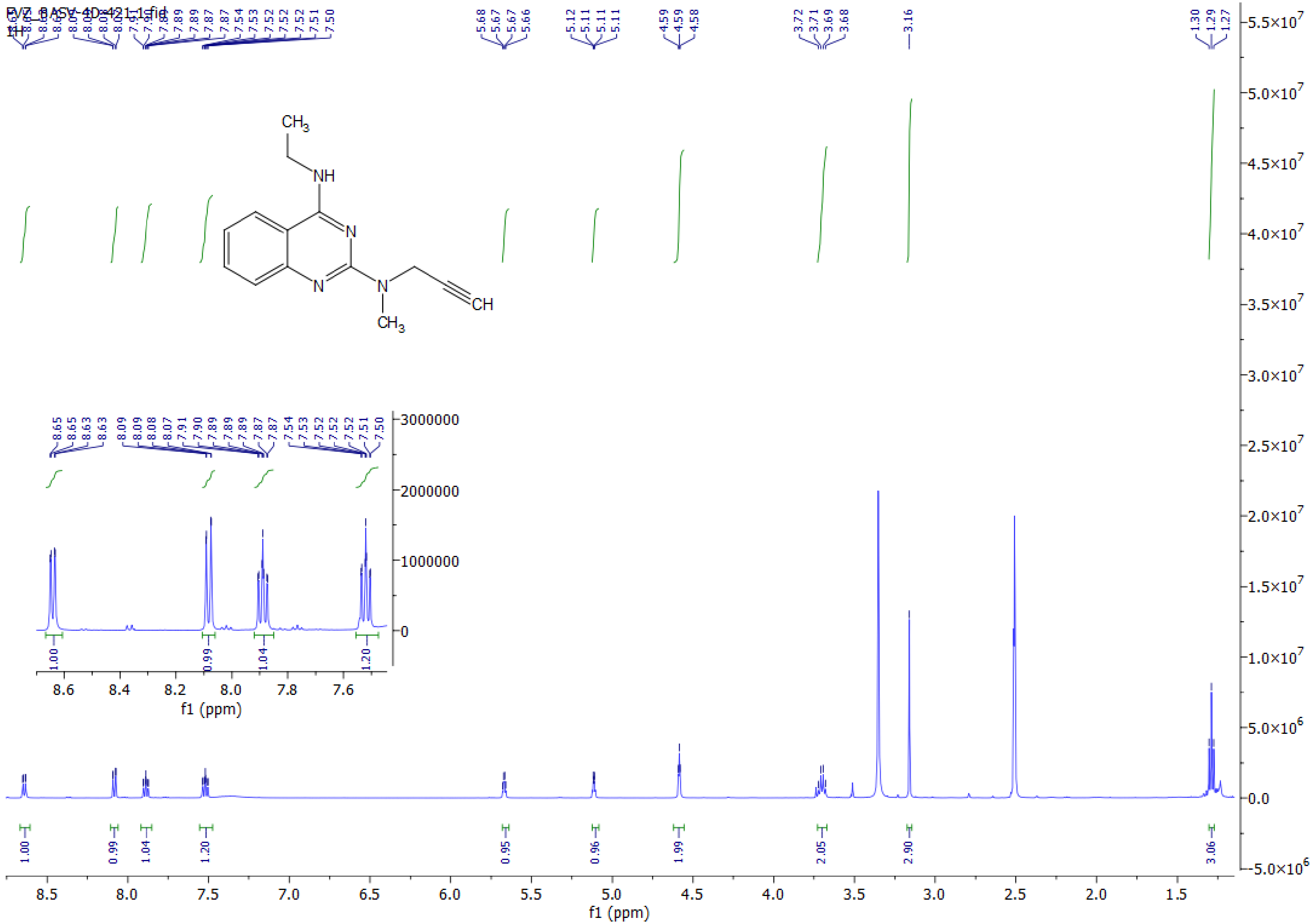

Fig. S47. ^{13}C NMR of *N4*-ethyl-*N2*-methyl-*N2*-(prop-2-yn-1-yl)quinazoline-2,4-diamine (I-6c)

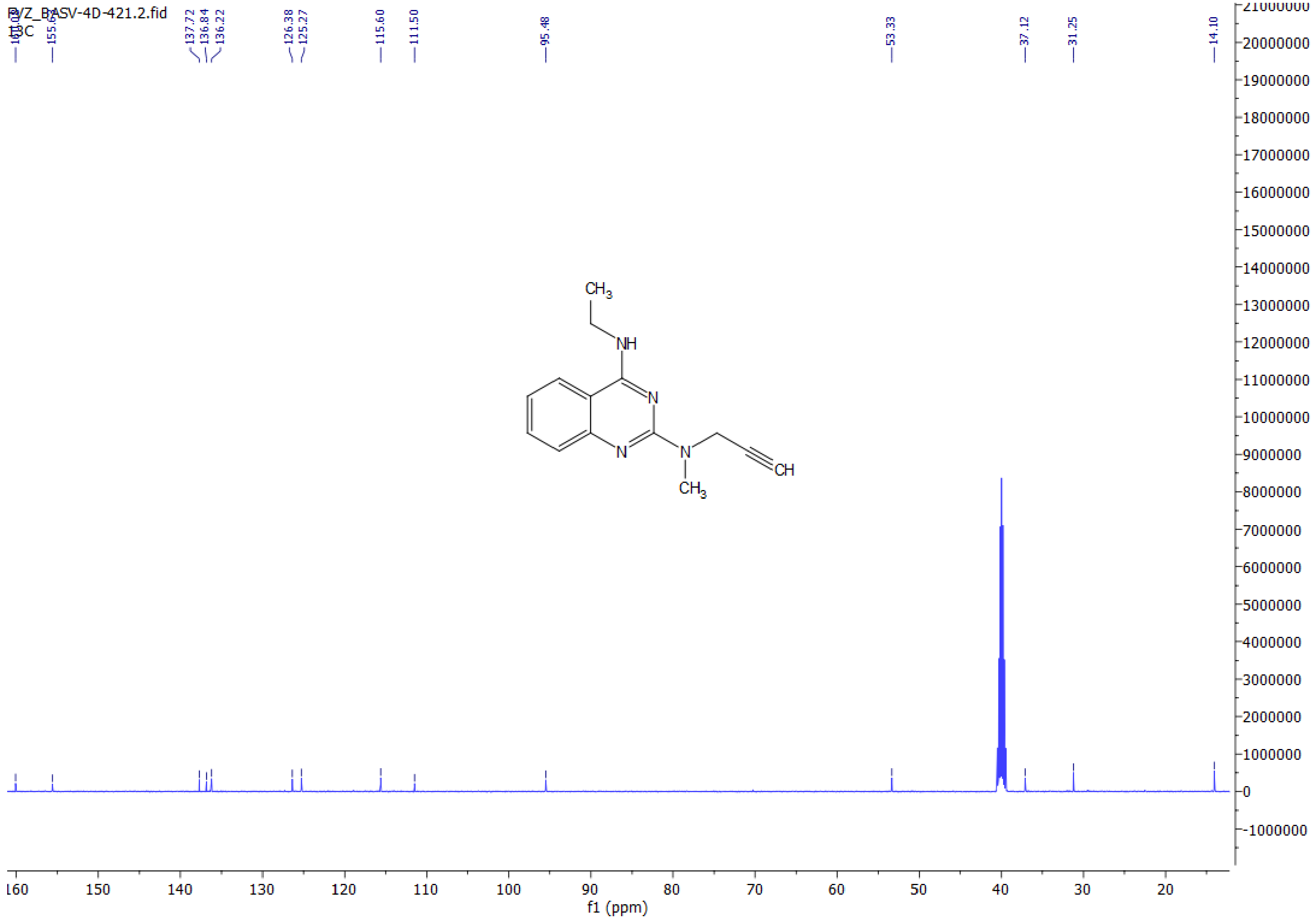

Fig. S48. ^{1}H NMR of *N2*-methyl-*N2*-(prop-2-yn-1-yl)-*N4*-(propan-2-yl)quinazoline-2,4-diamine (I-6d)

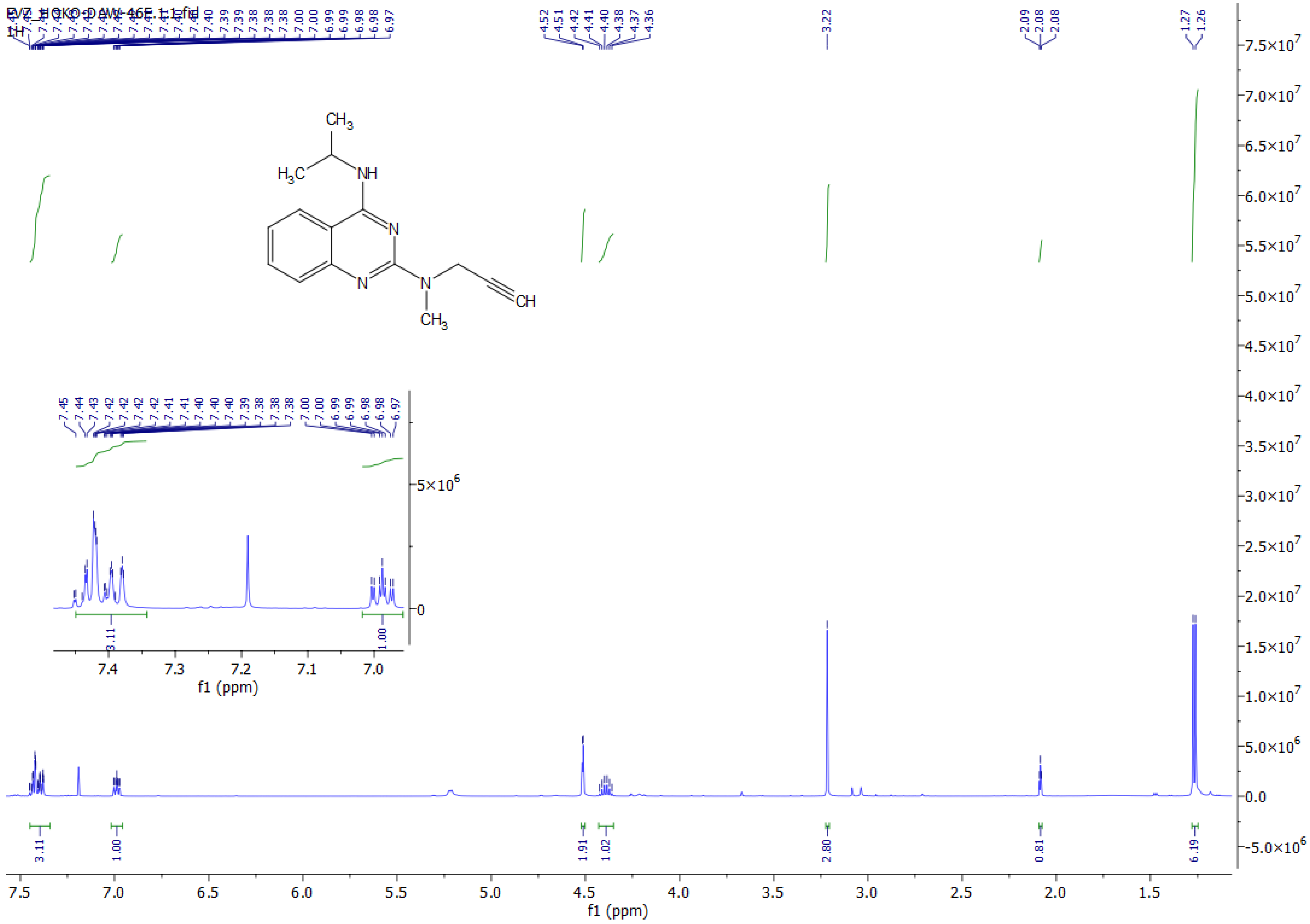

Fig. S49. ^{13}C NMR of *N2*-methyl-*N2*-(prop-2-yn-1-yl)-*N4*-(propan-2-yl)quinazoline-2,4-diamine (I-6d)

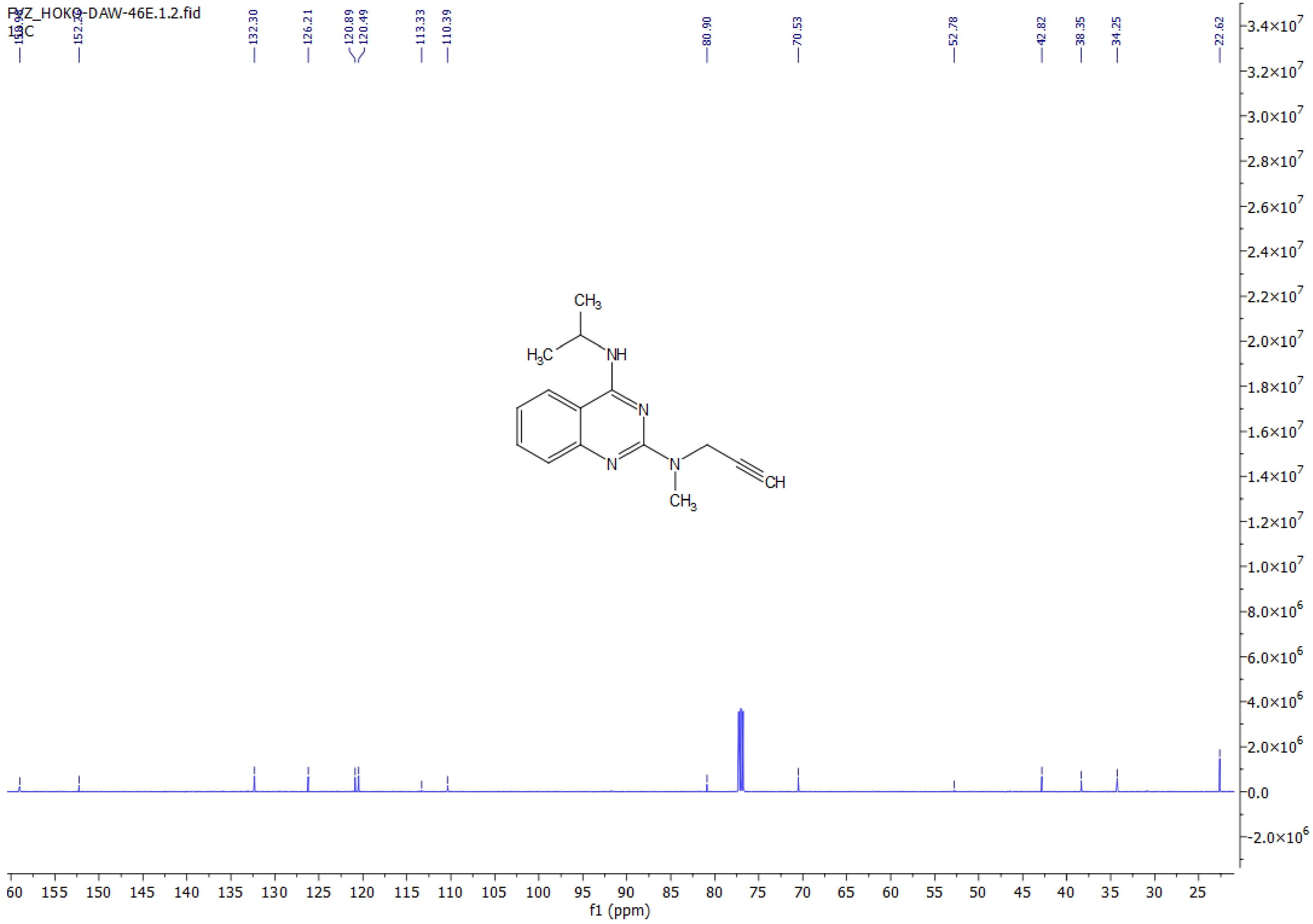

Fig. S50. ^{1}H NMR of *N4*-butyl-*N2*-methyl-*N2*-(prop-2-yn-1-yl)quinazoline-2,4-diamine (I-6e)

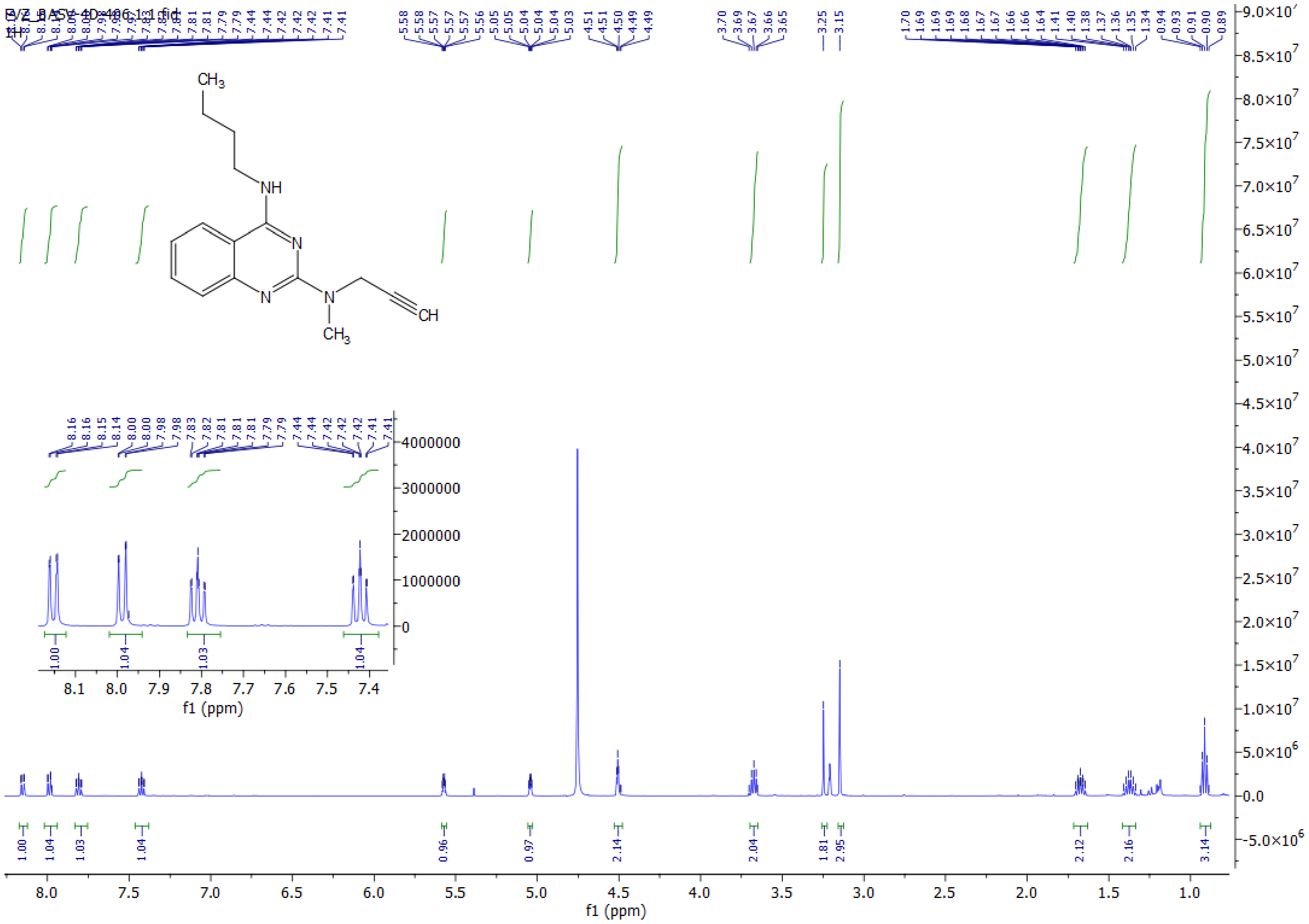

Fig. S51. ^{13}C NMR of *N4*-butyl-*N2*-methyl-*N2*-(prop-2-yn-1-yl)quinazoline-2,4-diamine (I-6e)

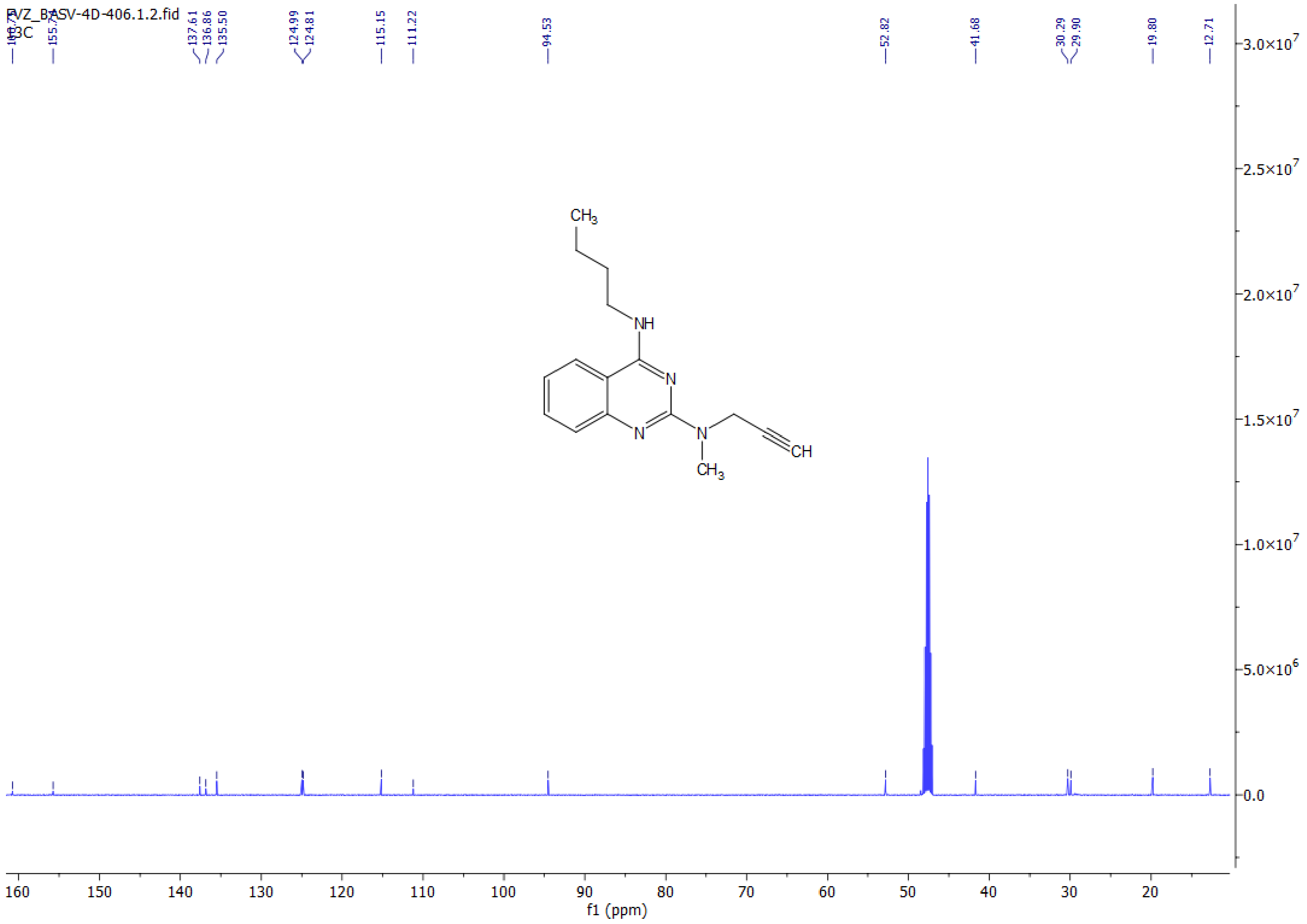

Fig. S52. ^{1}H NMR of *N4*-(2-methoxyethyl)-*N2*-methyl-*N2*-(prop-2-yn-1-yl)quinazoline-2,4-diamine (I-6f)

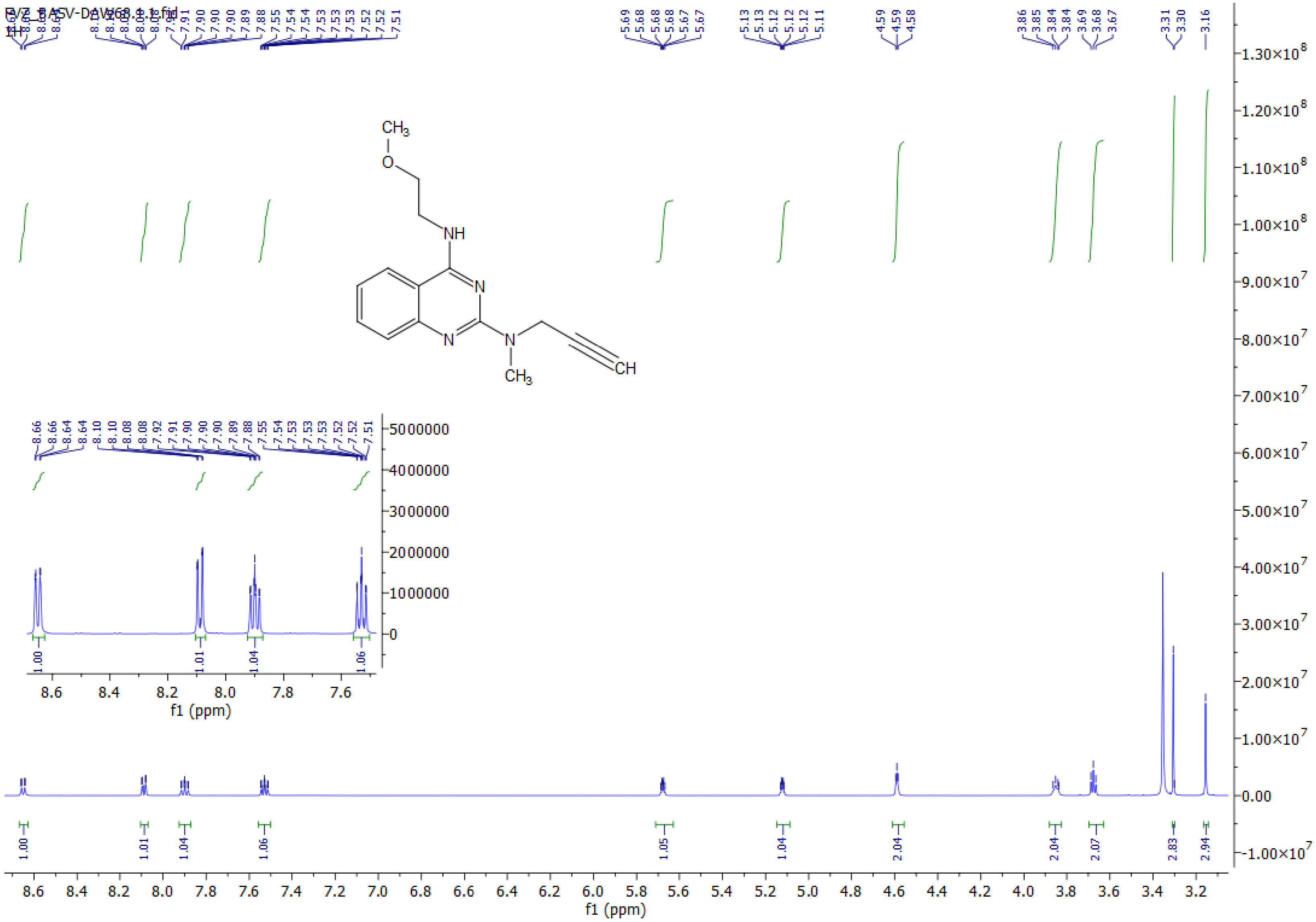

Fig. S53. ^{13}C NMR of *N4*-(2-methoxyethyl)-*N2*-methyl-*N2*-(prop-2-yn-1-yl)quinazoline-2,4-diamine (I-6f)

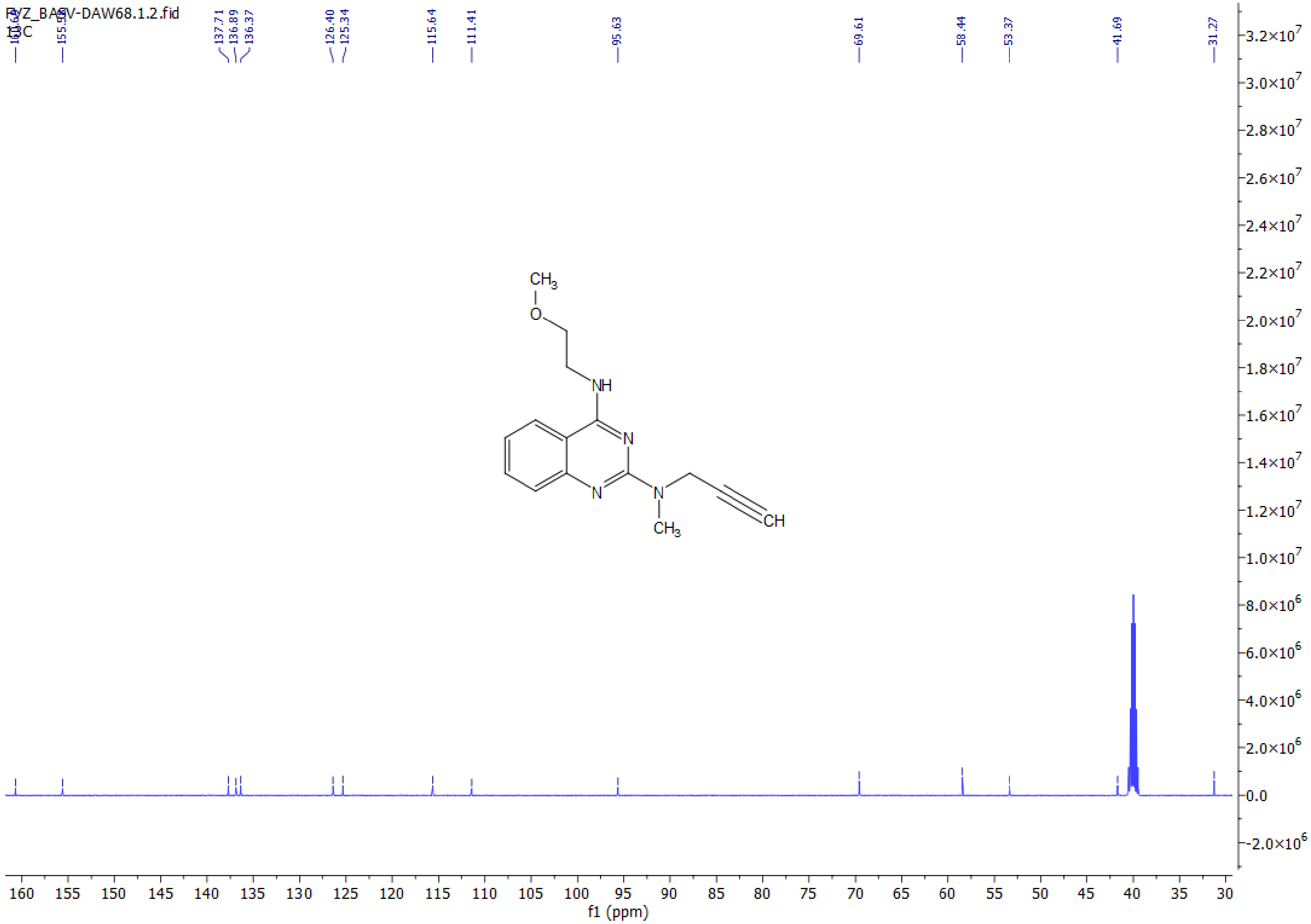

Fig. S54. ^{1}H NMR of *N4*-cyclopropyl-*N2*-methyl-*N2*-(prop-2-yn-1-yl)quinazoline-2,4-diamine (I-6g)

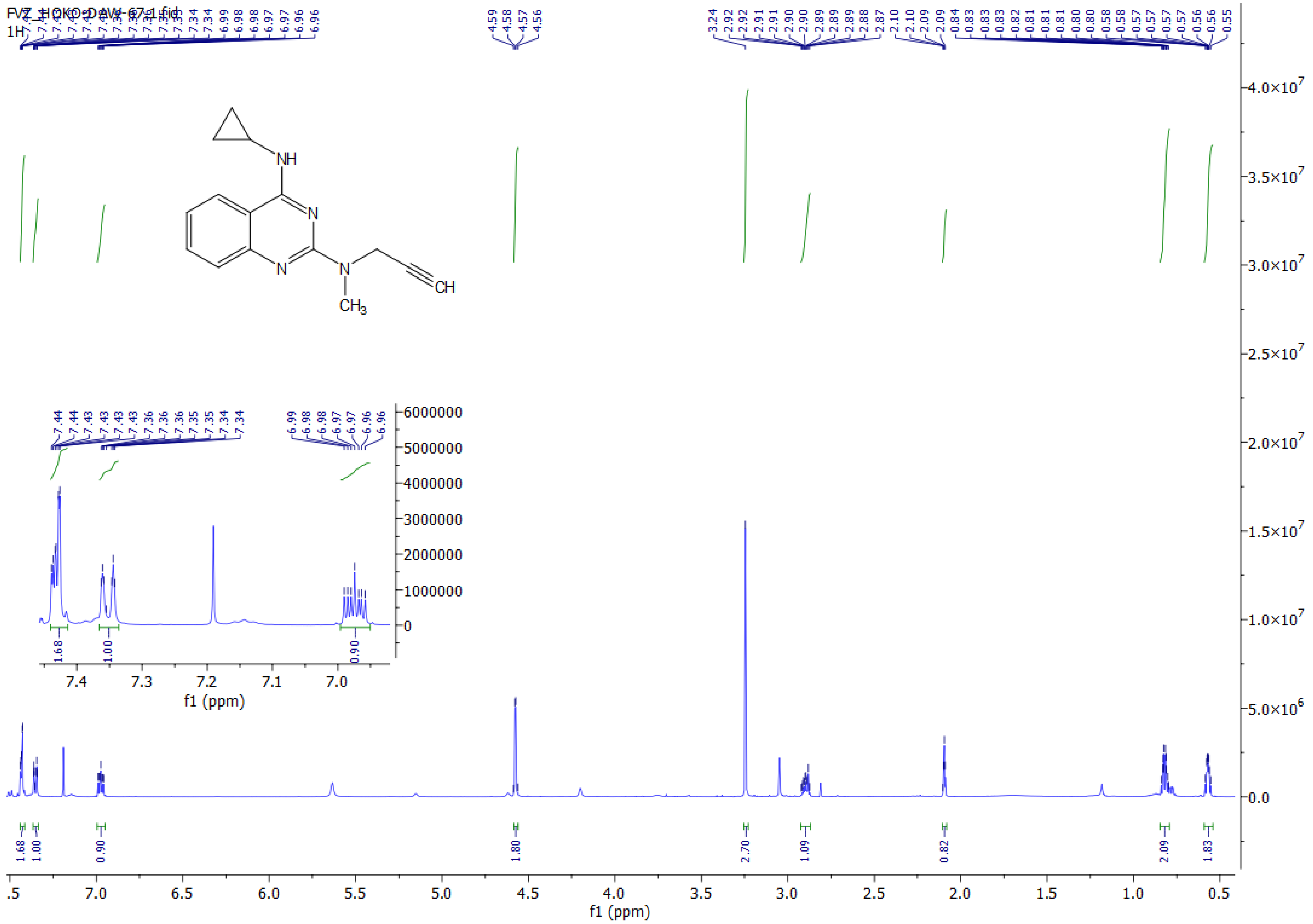

Fig. S55. ^{13}C NMR of *N4*-cyclopropyl-*N2*-methyl-*N2*-(prop-2-yn-1-yl)quinazoline-2,4-diamine (I-6g)

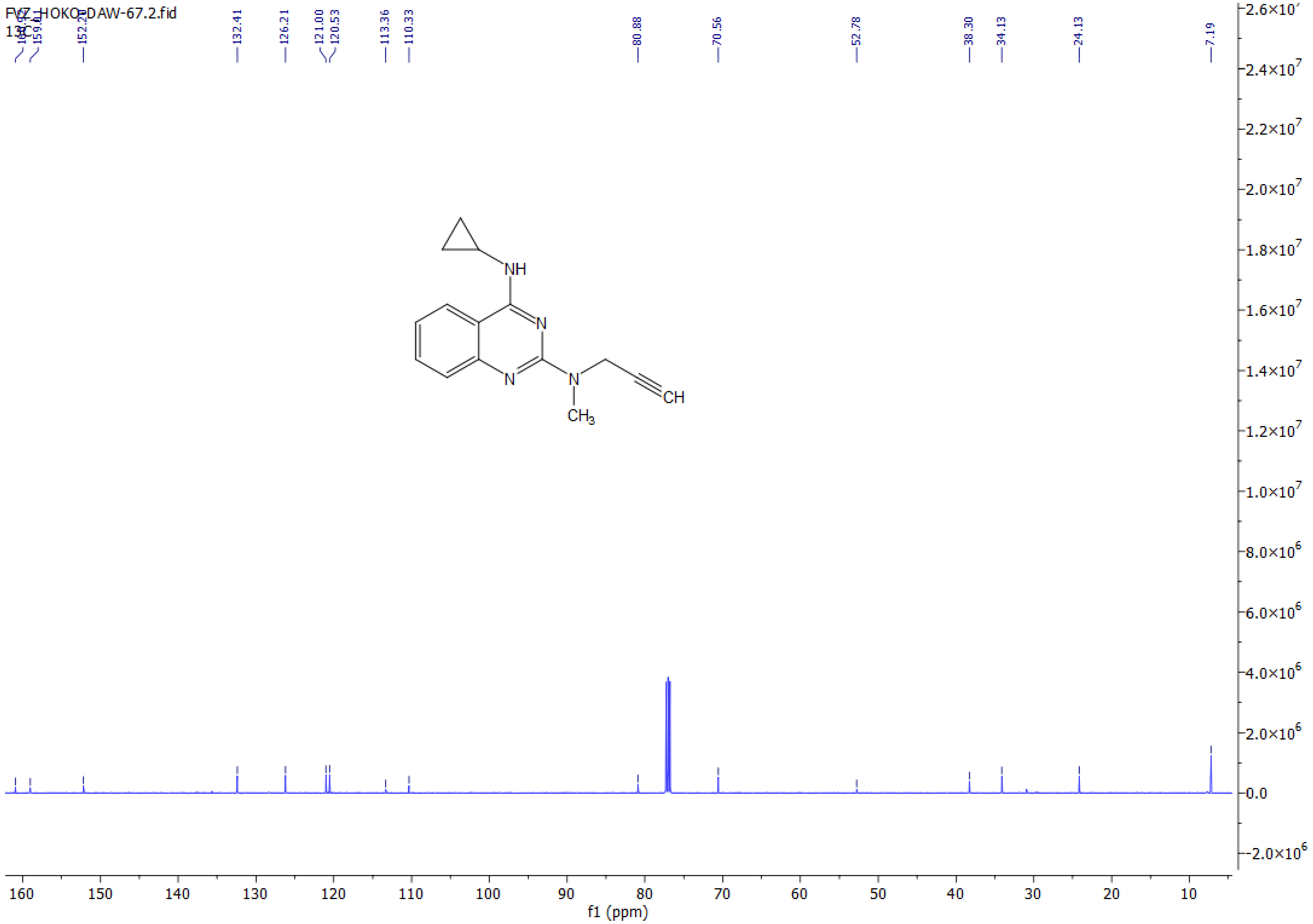

Fig. S56. ^{1}H NMR of *N4*-cyclohexyl-*N2*-methyl-*N2*-(prop-2-yn-1-yl)quinazoline-2,4-diamine (I-6h)

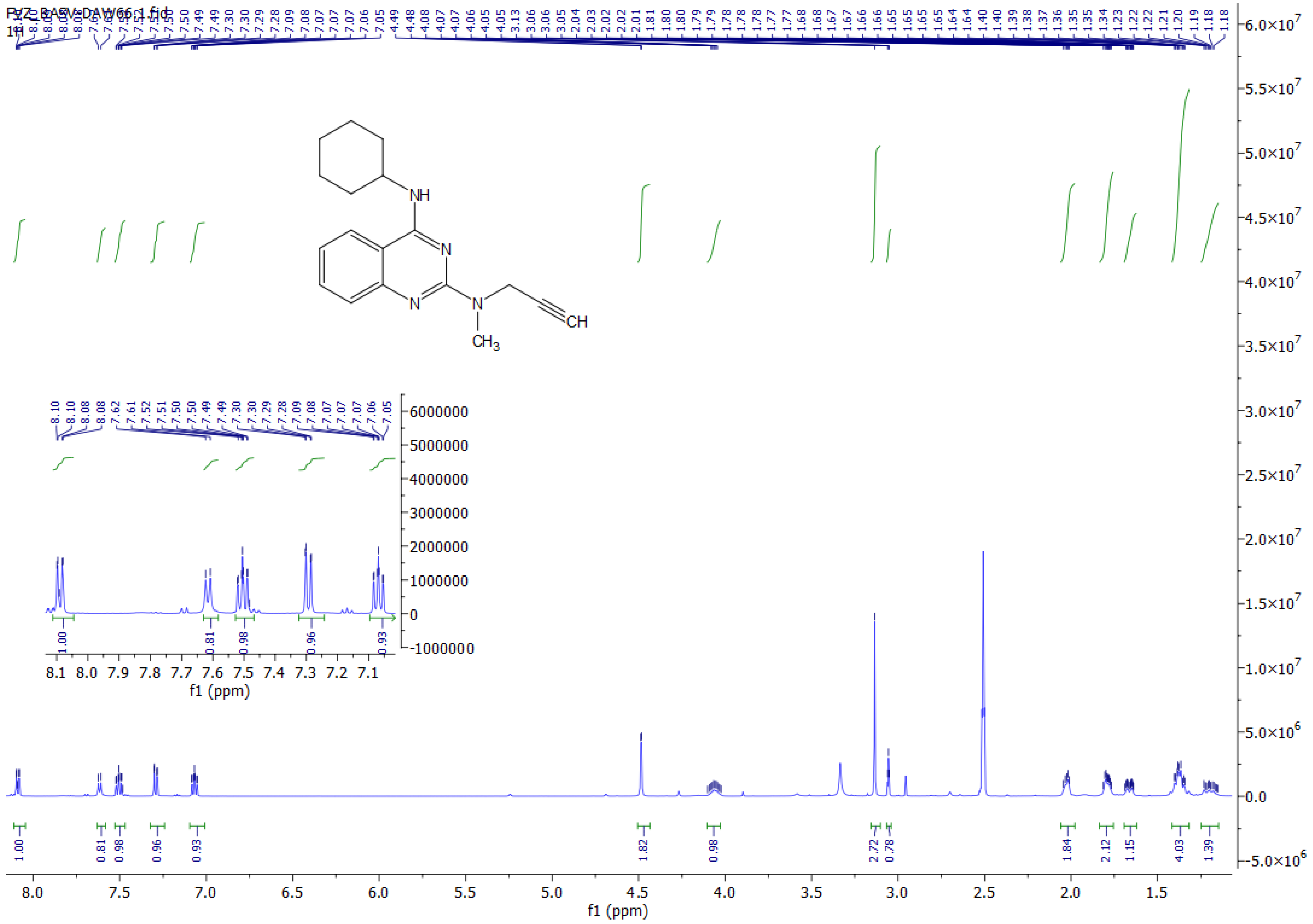

Fig. S57. ^{13}C NMR of *N4*-cyclohexyl-*N2*-methyl-*N2*-(prop-2-yn-1-yl)quinazoline-2,4-diamine (I-6h)

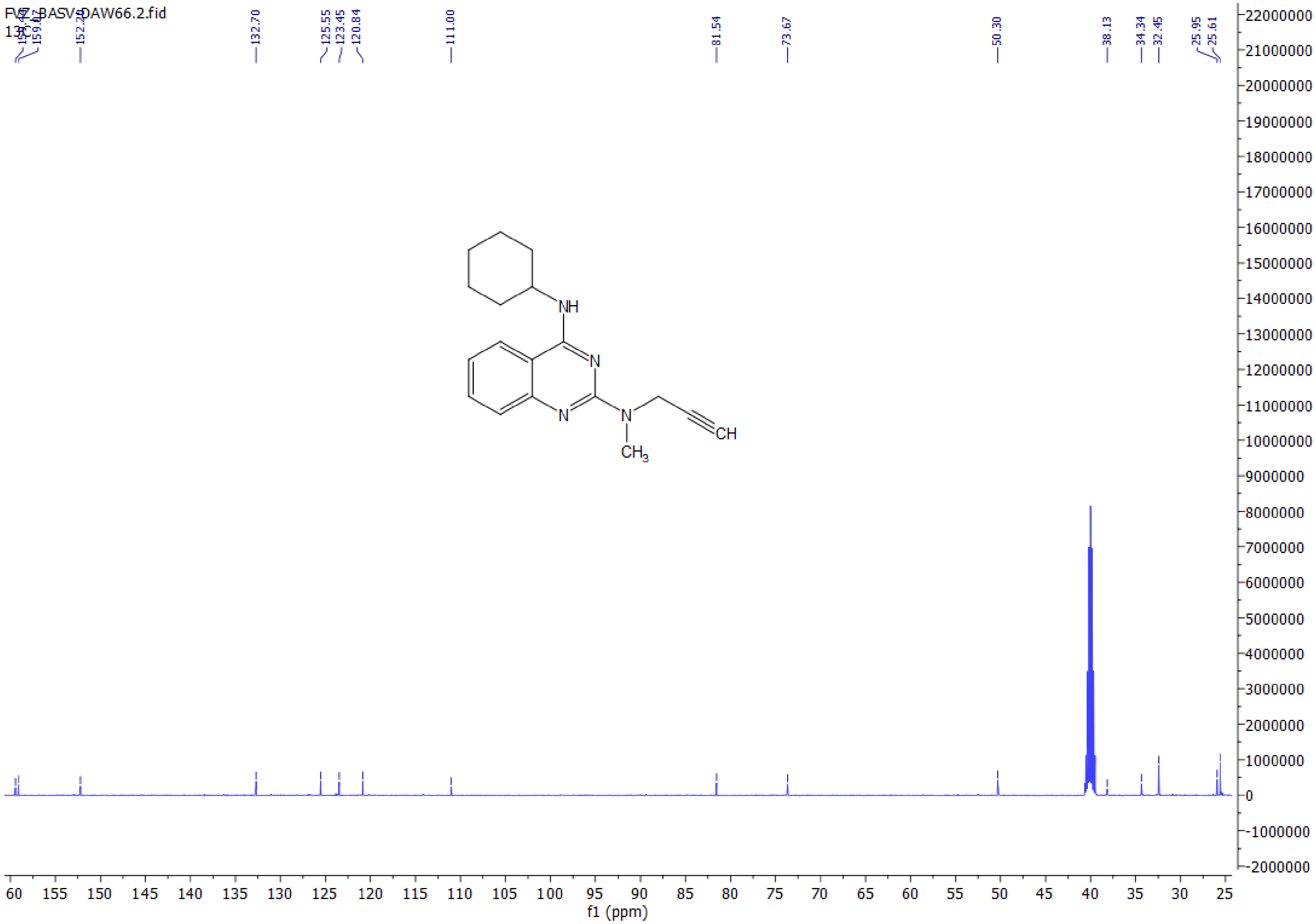

Fig. S58. ^{1}H **NMR of 7-chloro-*N2*-methyl-*N2*-(prop-2-yn-1-yl)quinazoline-2,4-diamine (II-6a)**

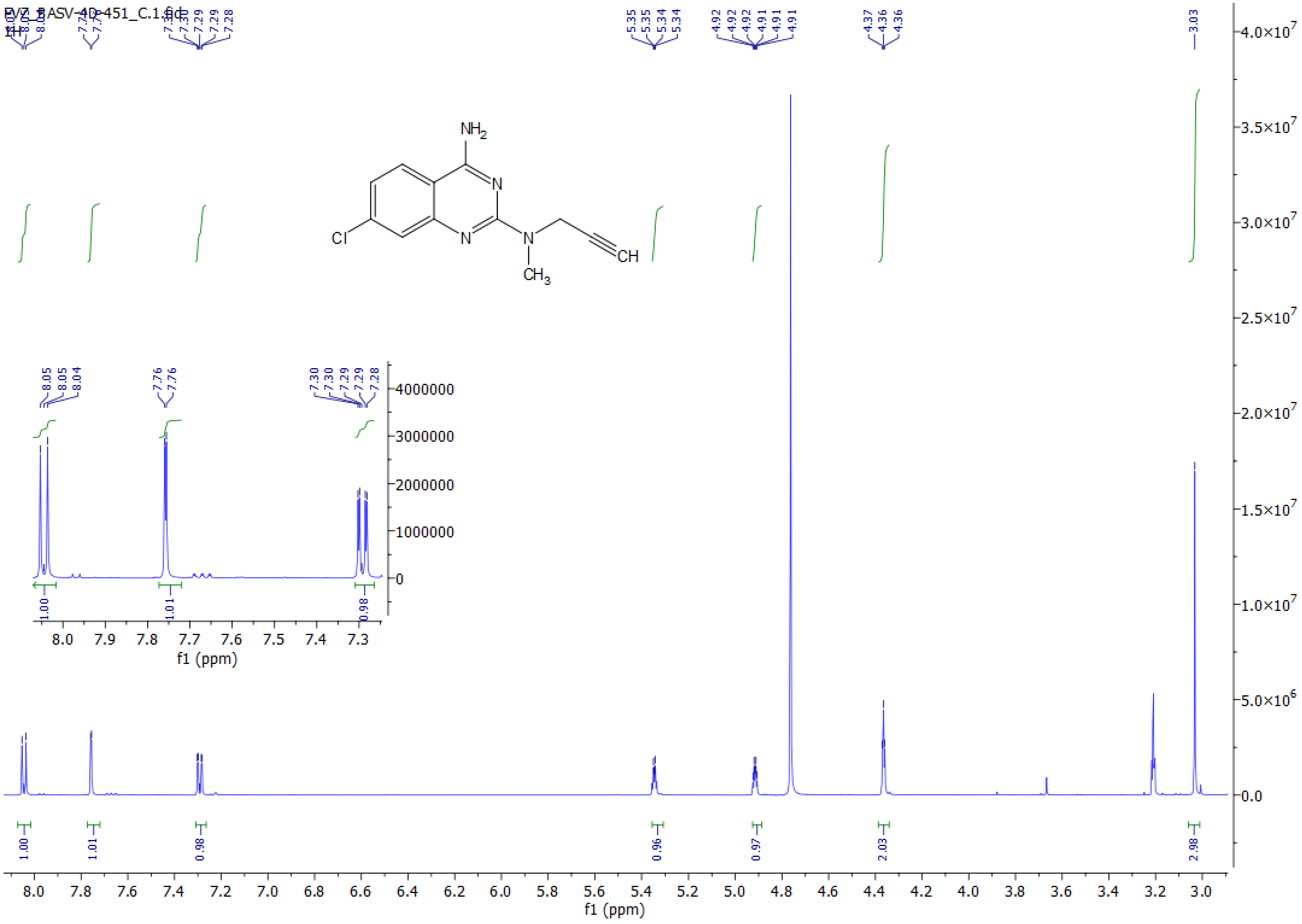

Fig. S59. ^{13}C NMR of 7-chloro-*N2*-methyl-*N2*-(prop-2-yn-1-yl)quinazoline-2,4-diamine (II-6a)

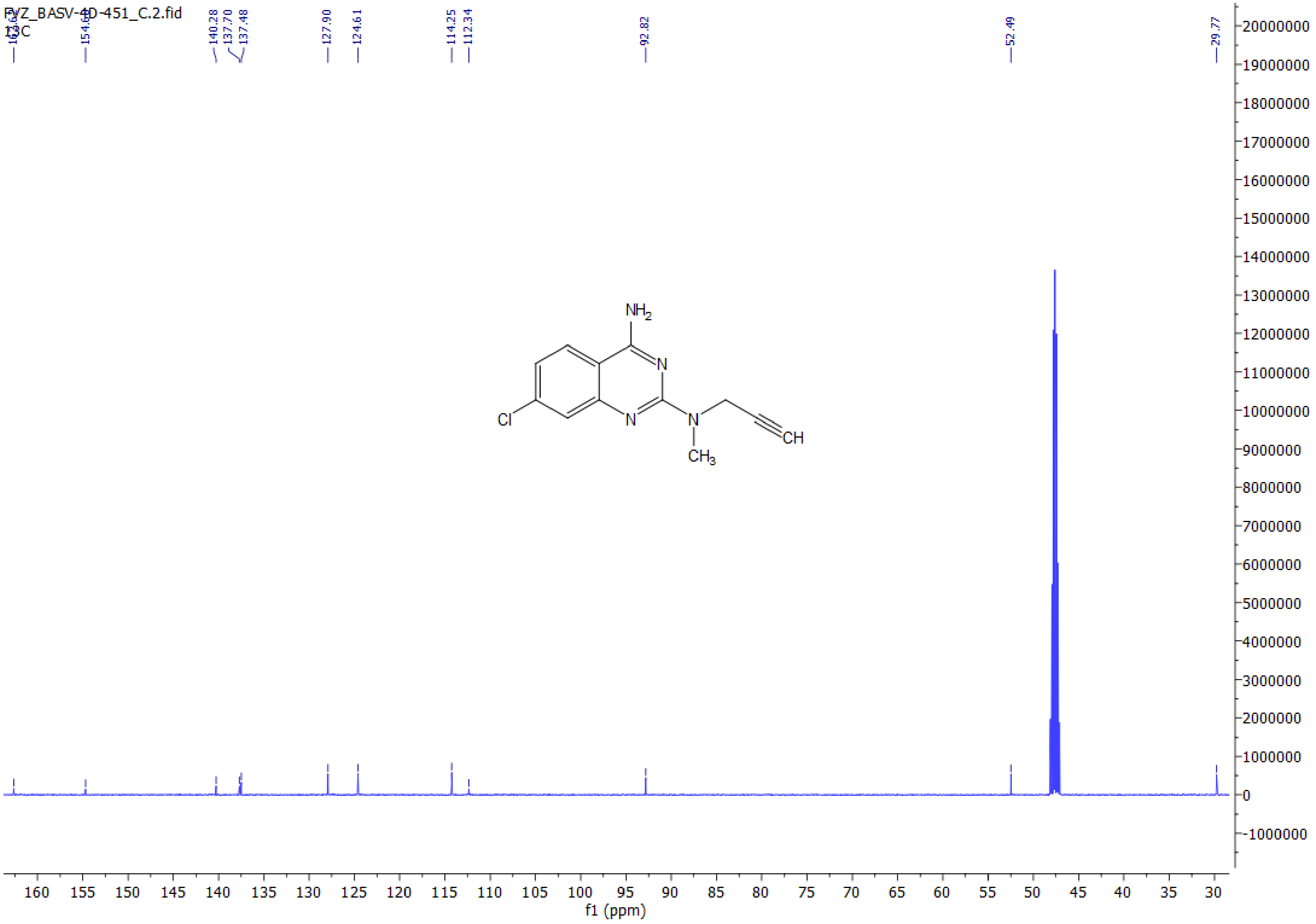

Fig. S60. ^{1}H NMR of 7-chloro-*N2,N4*-dimethyl-*N2*-(prop-2-yn-1-yl)quinazoline-2,4-diamine (II-6b)

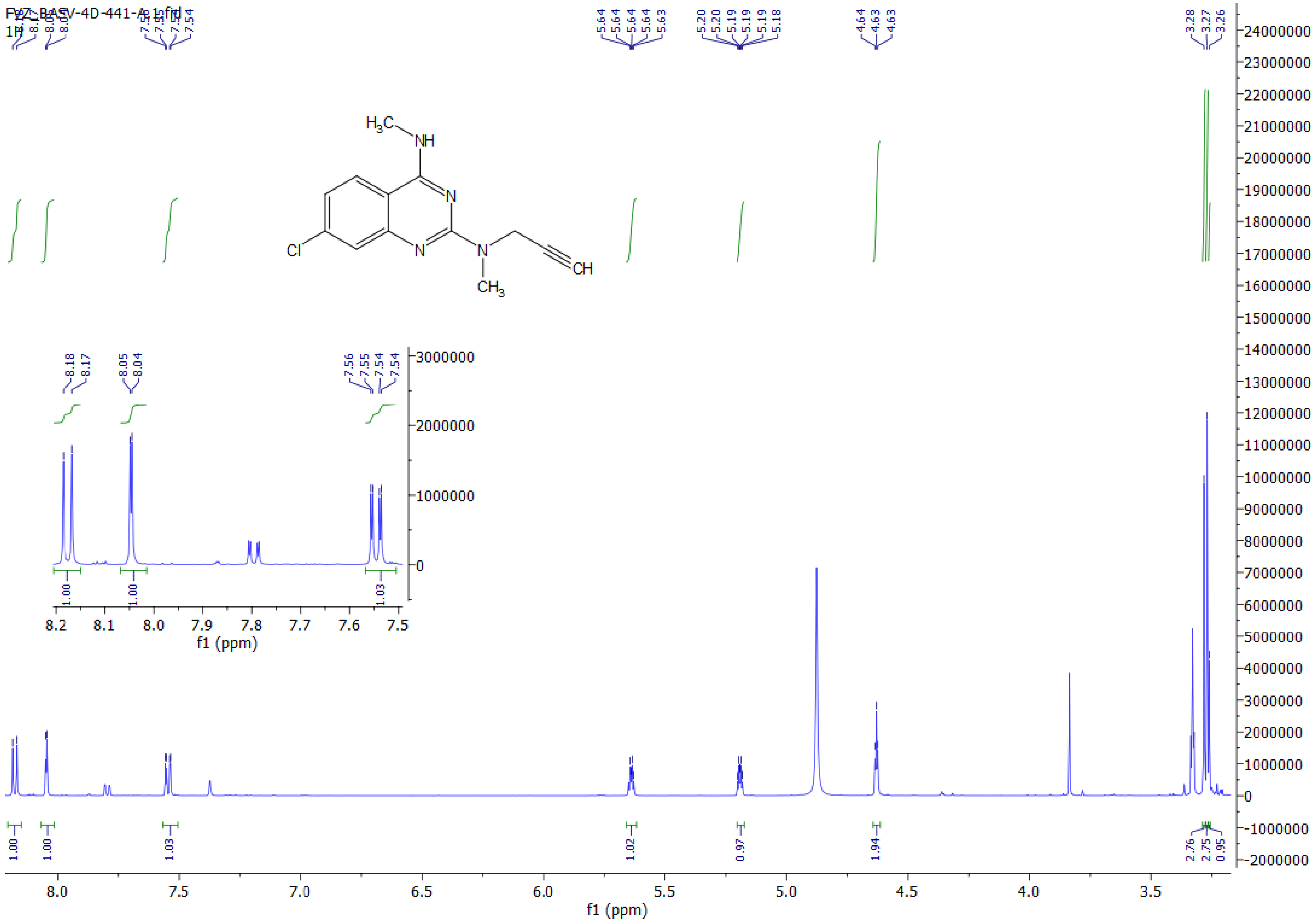

Fig. S61. ^{13}C NMR of 7-chloro-*N2*,*N4*-dimethyl-*N2*-(prop-2-yn-1-yl)quinazoline-2,4-diamine (II-6b)

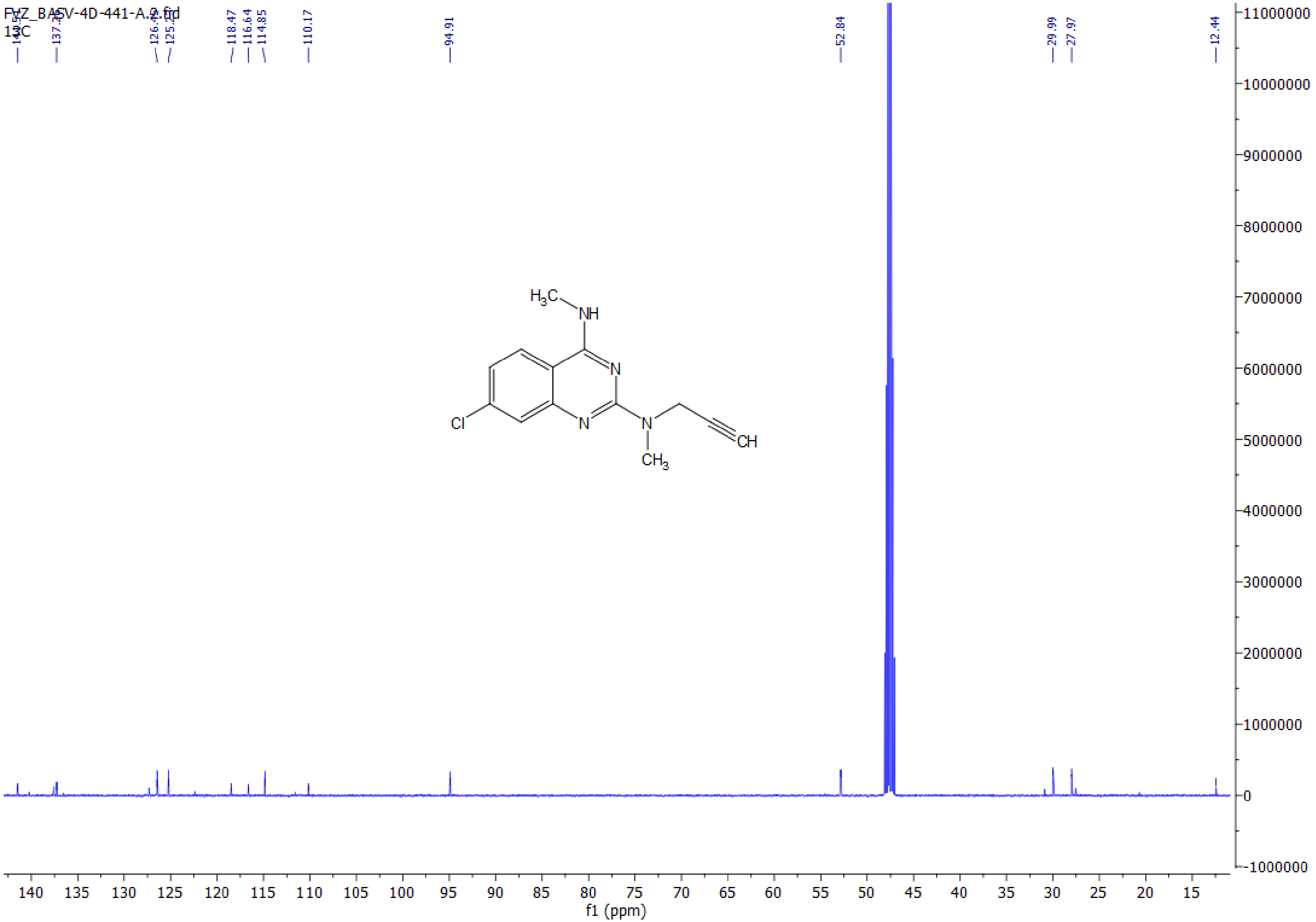

Fig. S62. ^{1}H NMR of 7-chloro-*N4*-ethyl-*N2*-methyl-*N2*-(prop-2-yn-1-yl)quinazoline-2,4-diamine (II-6c)

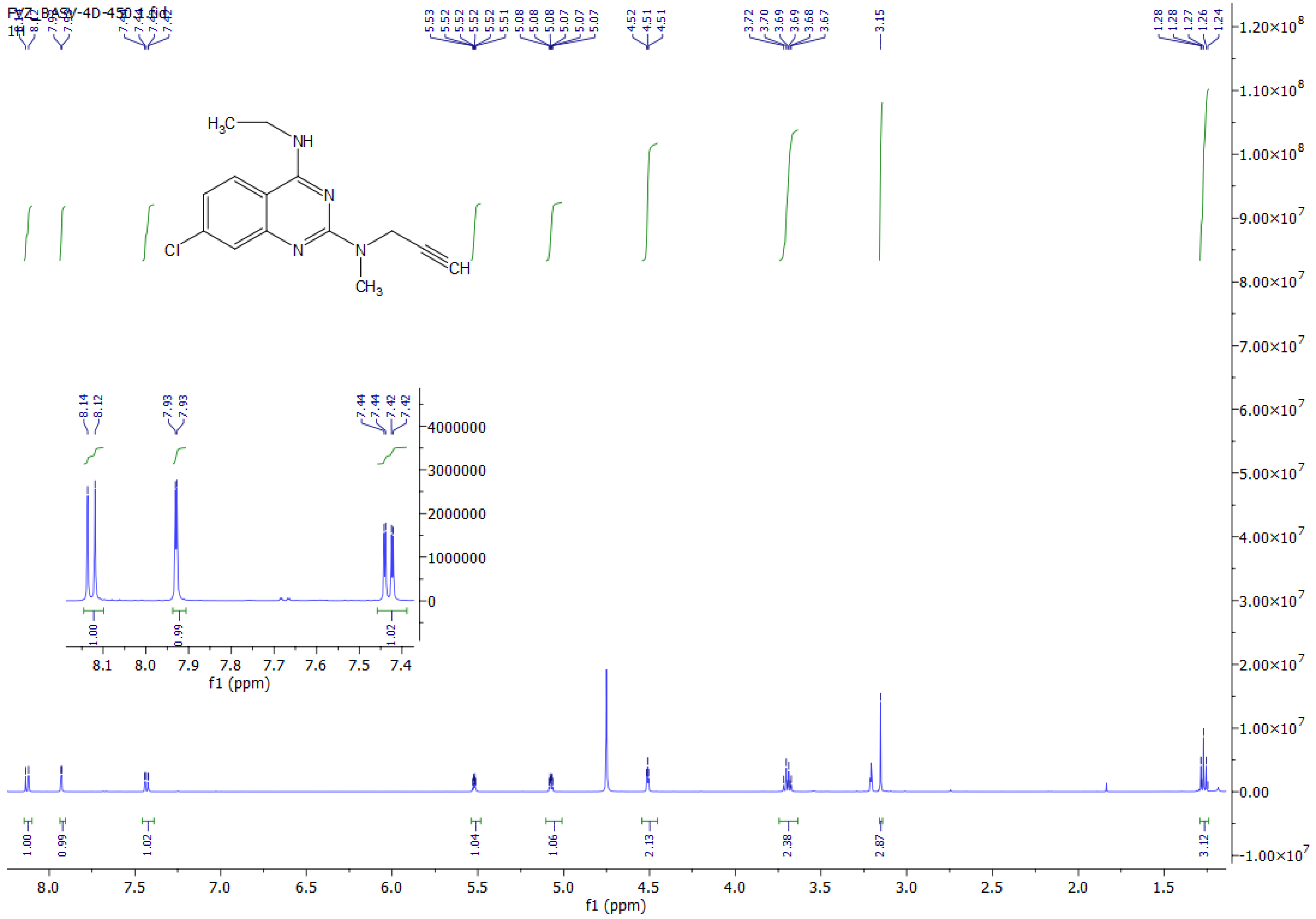

Fig. S63. ^{13}C NMR of 7-chloro-*N4*-ethyl-*N2*-methyl-*N2*-(prop-2-yn-1-yl)quinazoline-2,4-diamine (II-6c)

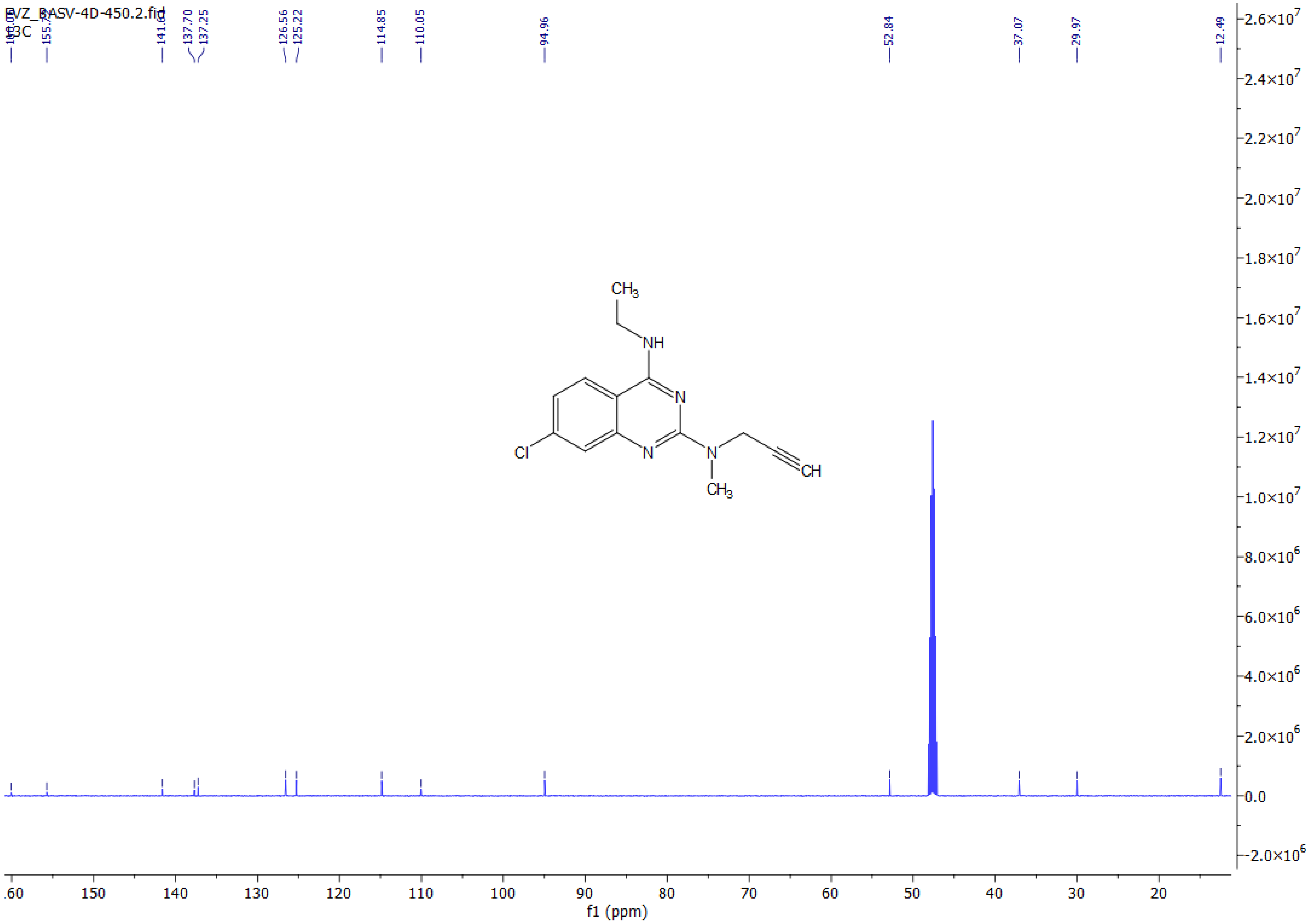

Fig. S64. ^{1}H **NMR of 7-chloro-*N2*-methyl-*N2*-(prop-2-yn-1-yl)-*N4*-(propan-2-yl)quinazoline-2,4-diamine (II-6d)**

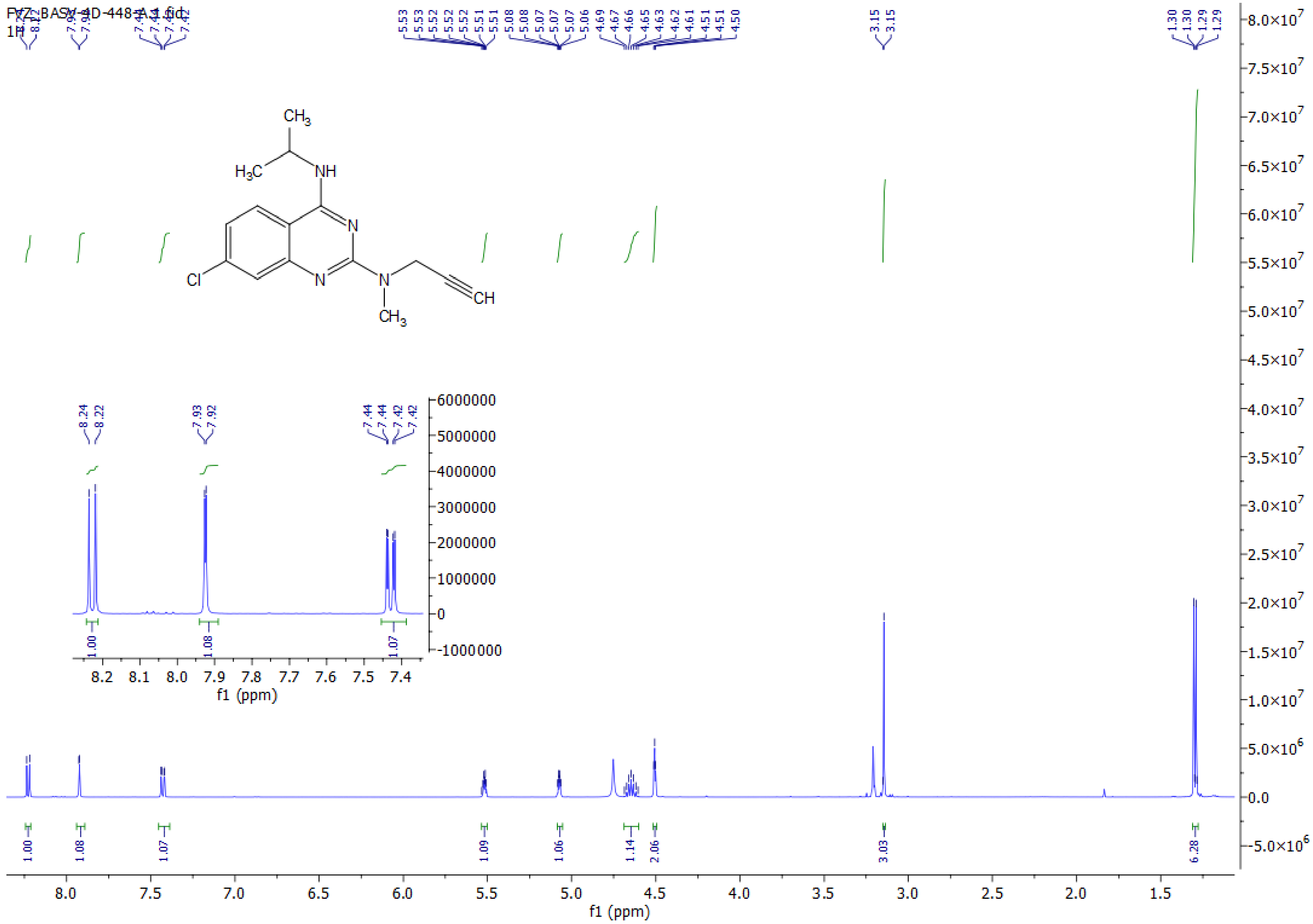

Fig. S65. ^{13}C NMR of 7-chloro-*N2*-methyl-*N2*-(prop-2-yn-1-yl)-*N4*-(propan-2-yl)quinazoline-2,4-diamine (II-6d)

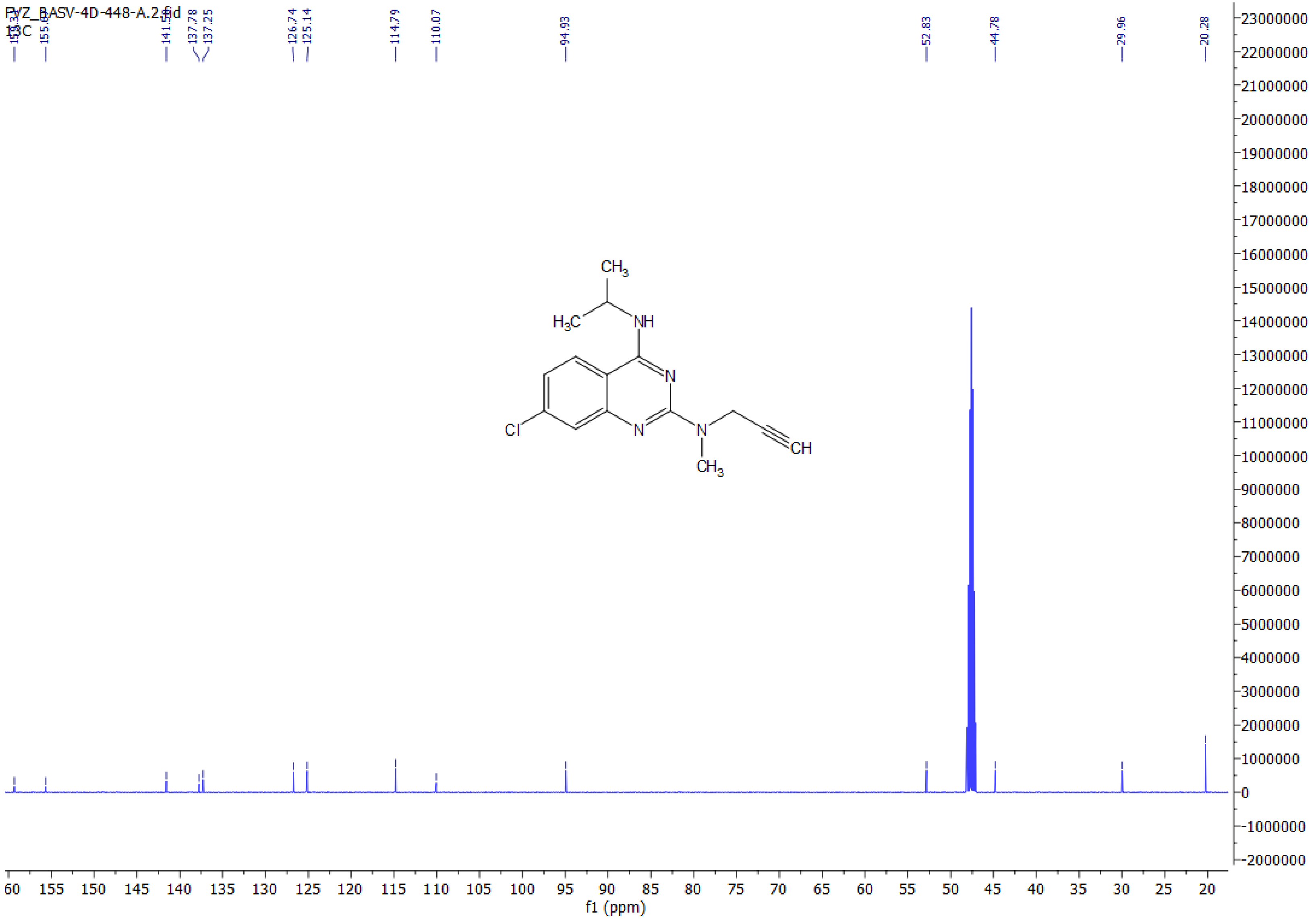

Fig. S66. ^{1}H NMR of *N4*-butyl-7-chloro-*N2*-methyl-*N2*-(prop-2-yn-1-yl)quinazoline-2,4-diamine (II-6e)

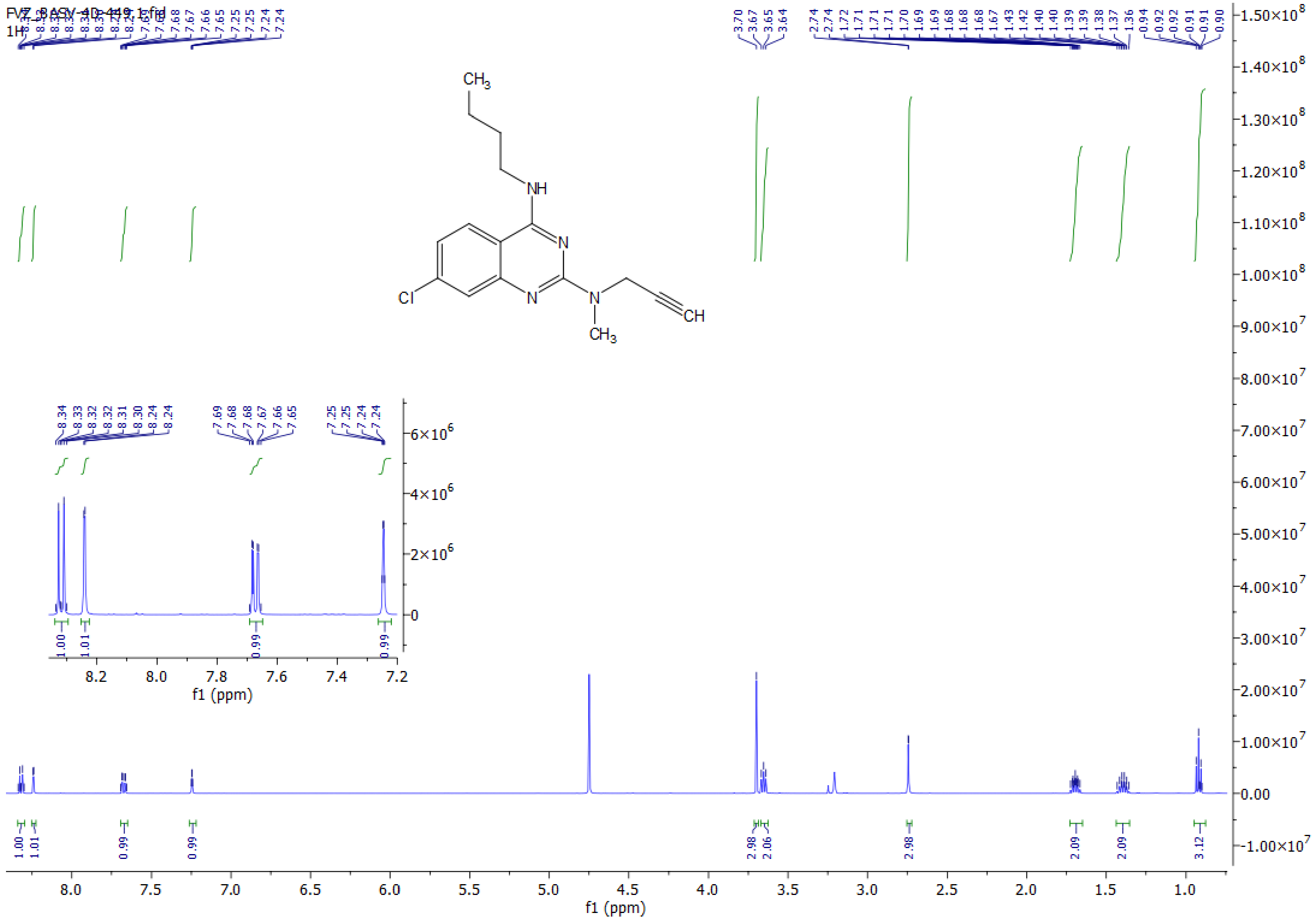

Fig. S67. ^{13}C NMR of *N4*-butyl-7-chloro-*N2*-methyl-*N2*-(prop-2-yn-1-yl)quinazoline-2,4-diamine (II-6e)

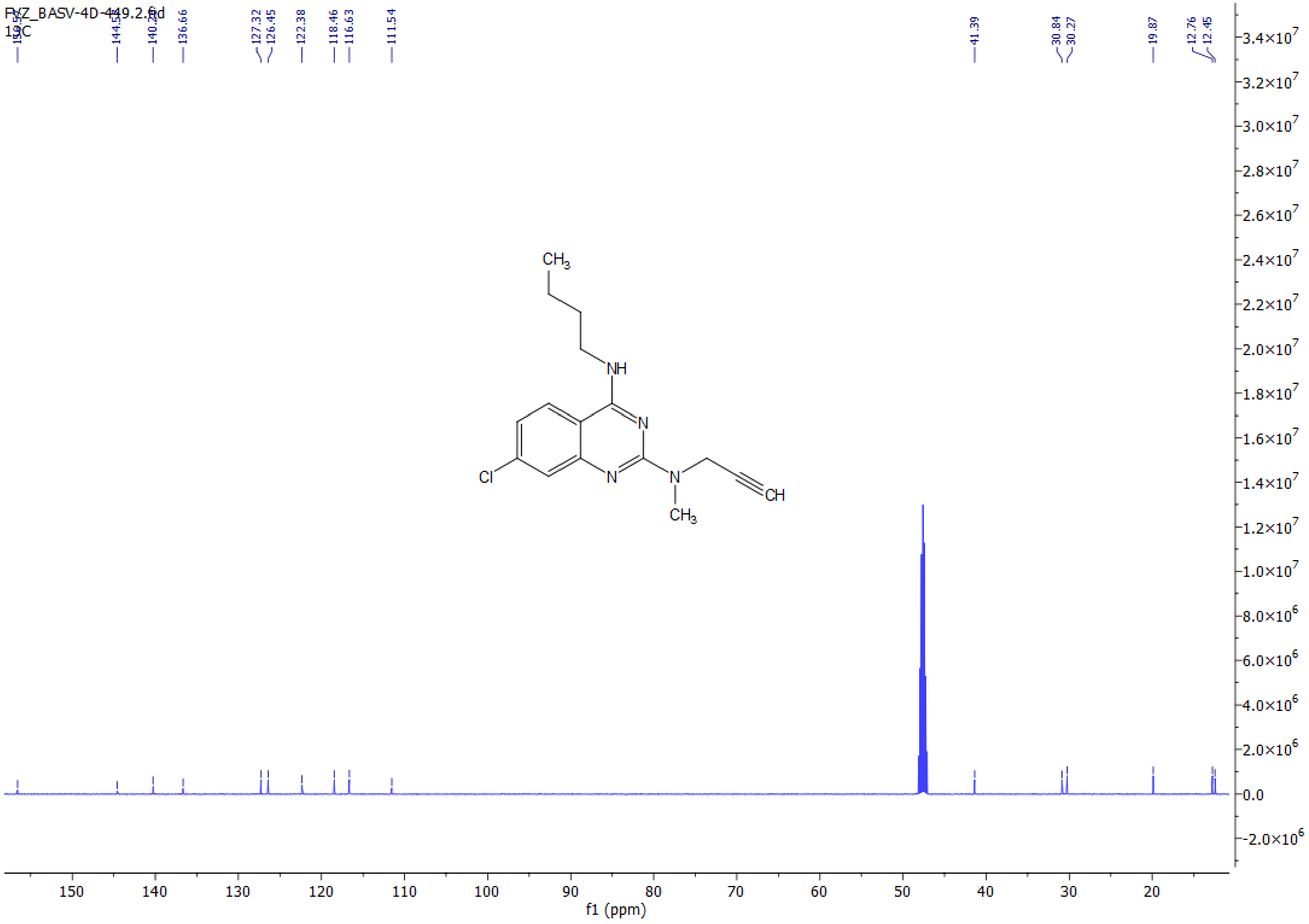

Fig. S68. ^{1}**H NMR of 7-chloro-*N4*-(2-methoxyethyl)-*N2*-methyl-*N2*-(prop-2-yn-1-yl)quinazoline-2,4-diamine (II-6f)**

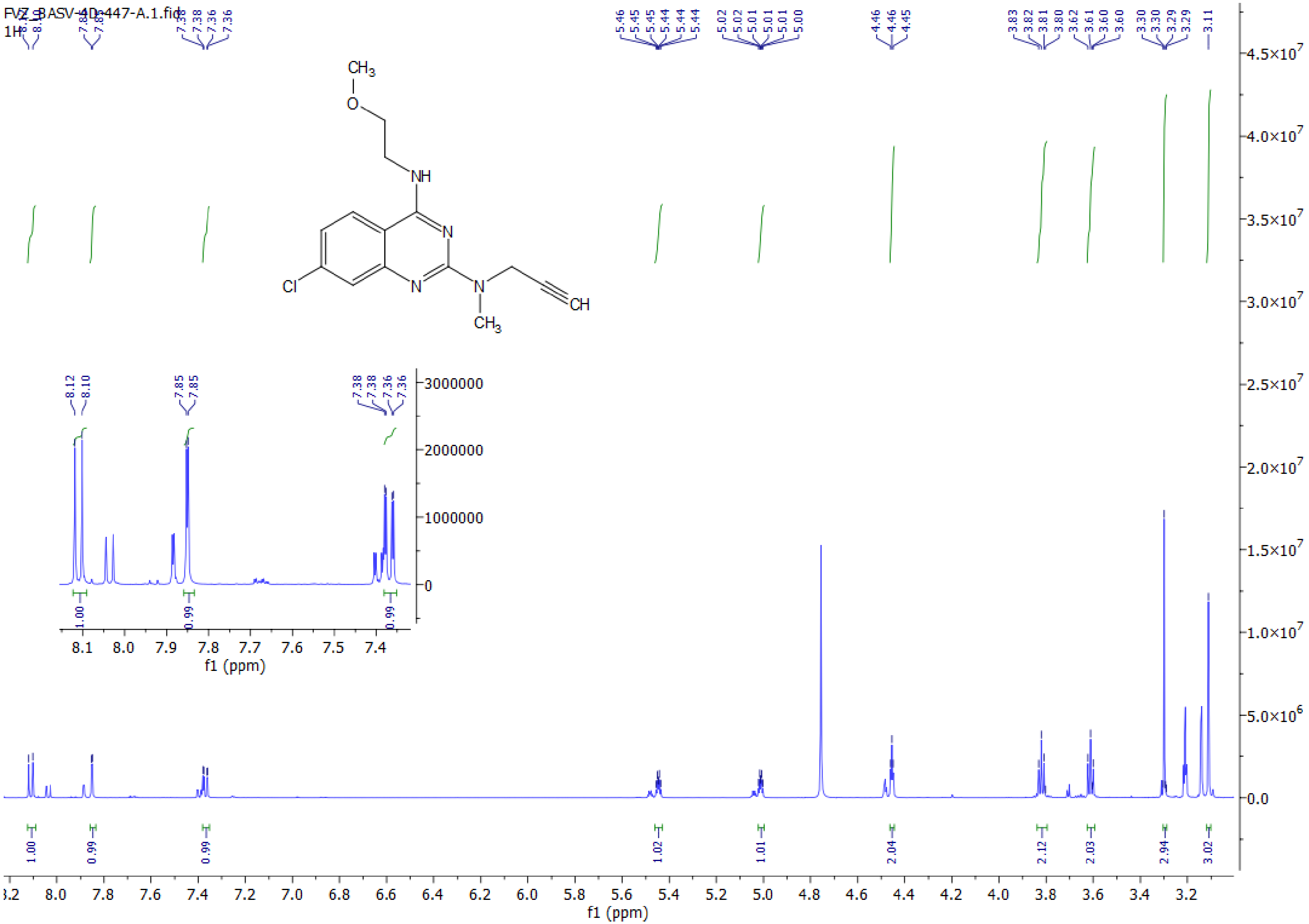

Fig. S69. ^{13}C NMR of 7-chloro-*N4*-(2-methoxyethyl)-*N2*-methyl-*N2*-(prop-2-yn-1-yl)quinazoline-2,4-diamine (II-6f)

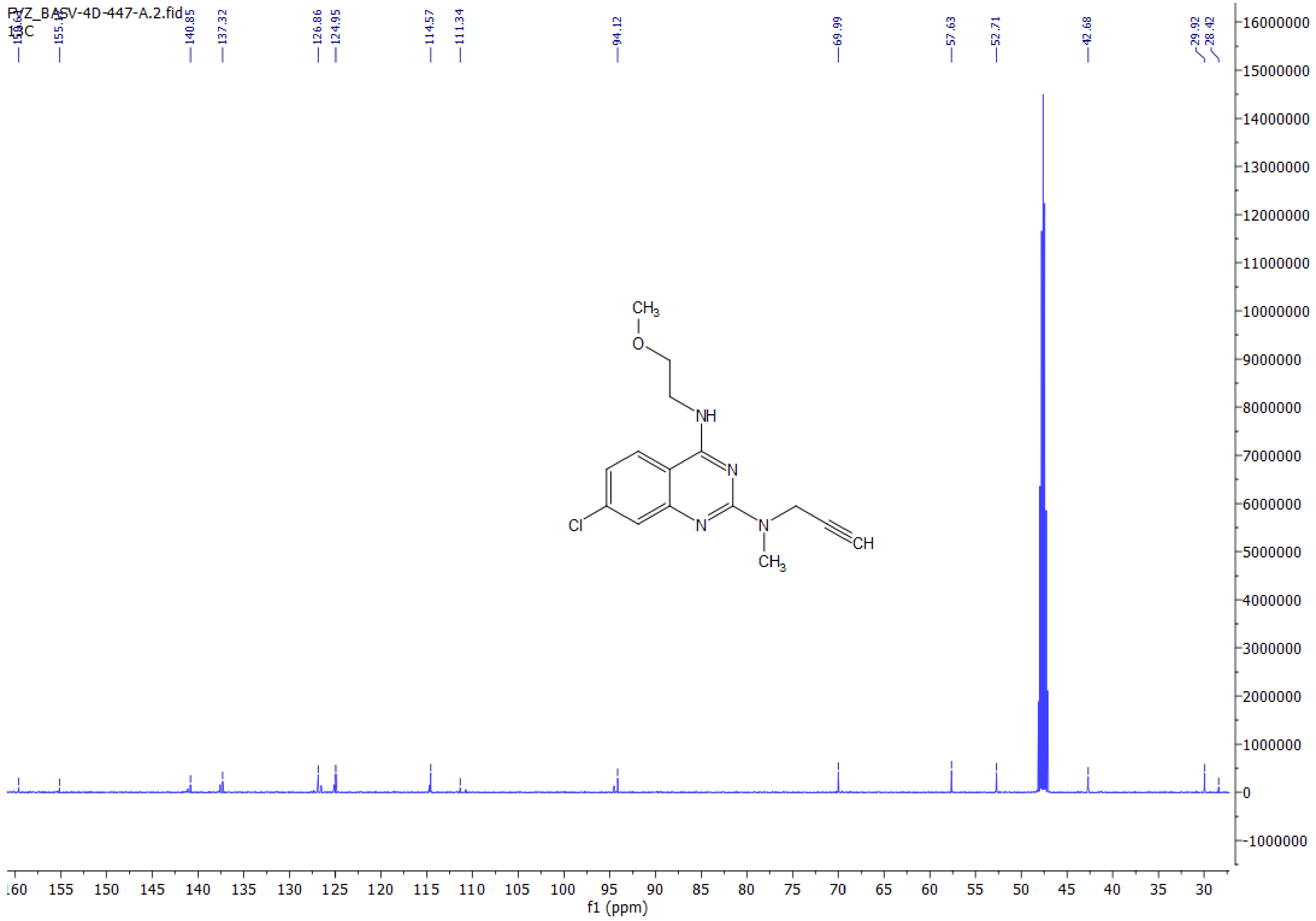

Fig. S70. ^{1}H NMR of 7-chloro-*N4*-cyclopropyl-*N2*-methyl-*N2*-(prop-2-yn-1-yl)quinazoline-2,4-diamine (II-6g)

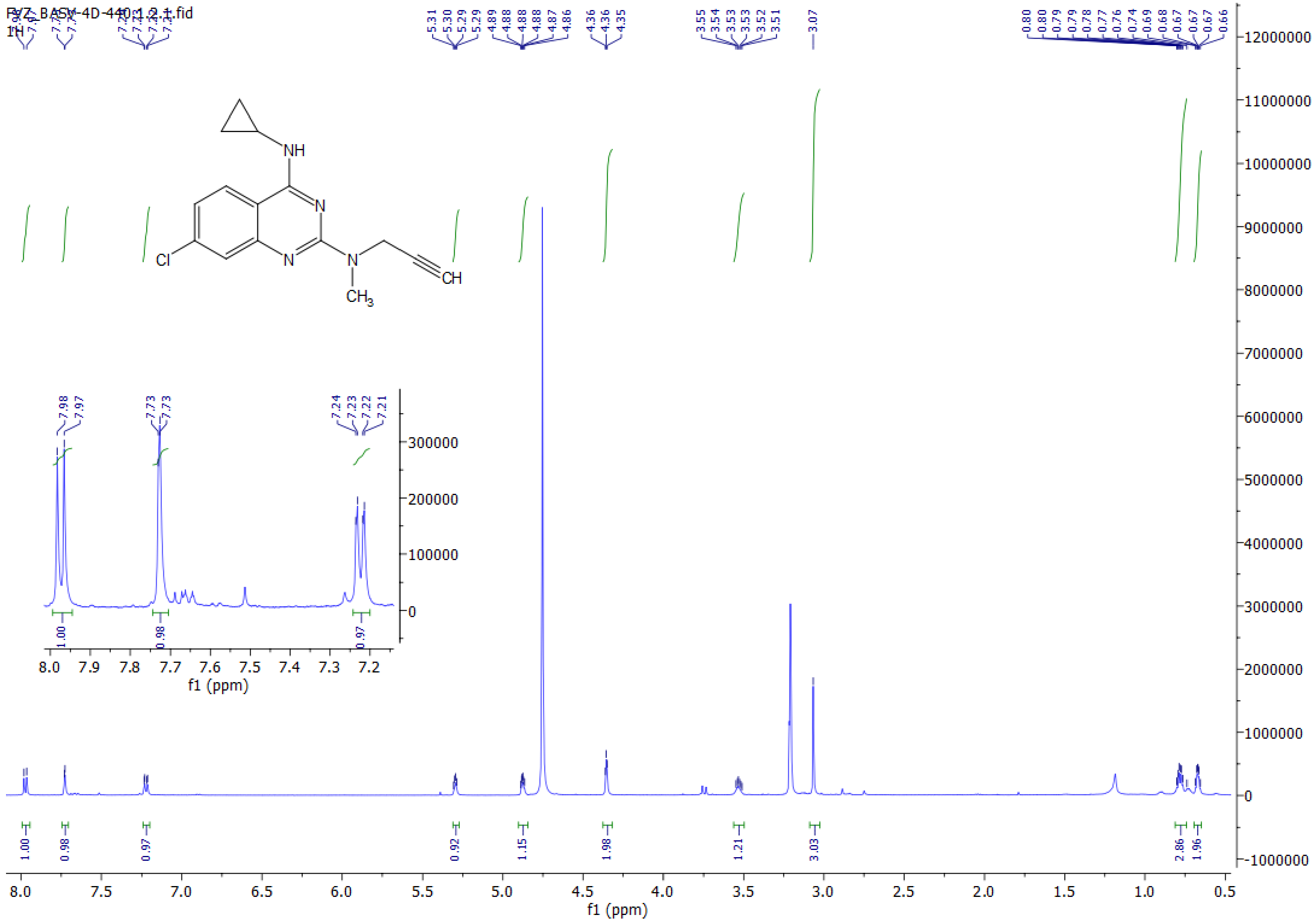

Fig. S71. ^{13}C NMR of 7-chloro-*N4*-cyclopropyl-*N2*-methyl-*N2*-(prop-2-yn-1-yl)quinazoline-2,4-diamine (II-6g)

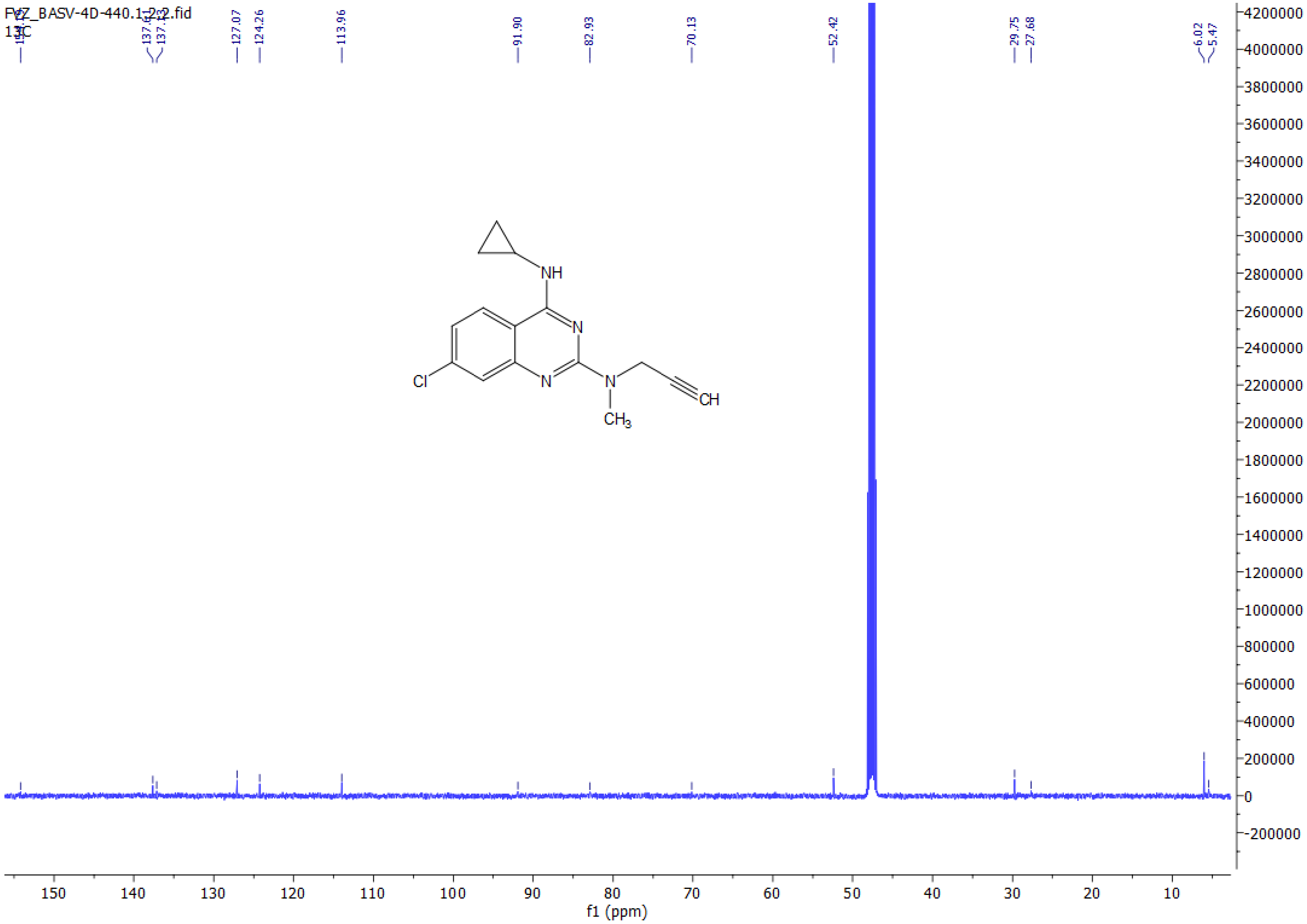

Fig. S72. ^{1}H NMR of 7-chloro-*N4*-cyclohexyl-*N2*-methyl-*N2*-(prop-2-yn-1-yl)quinazoline-2,4-diamine (II-6h)

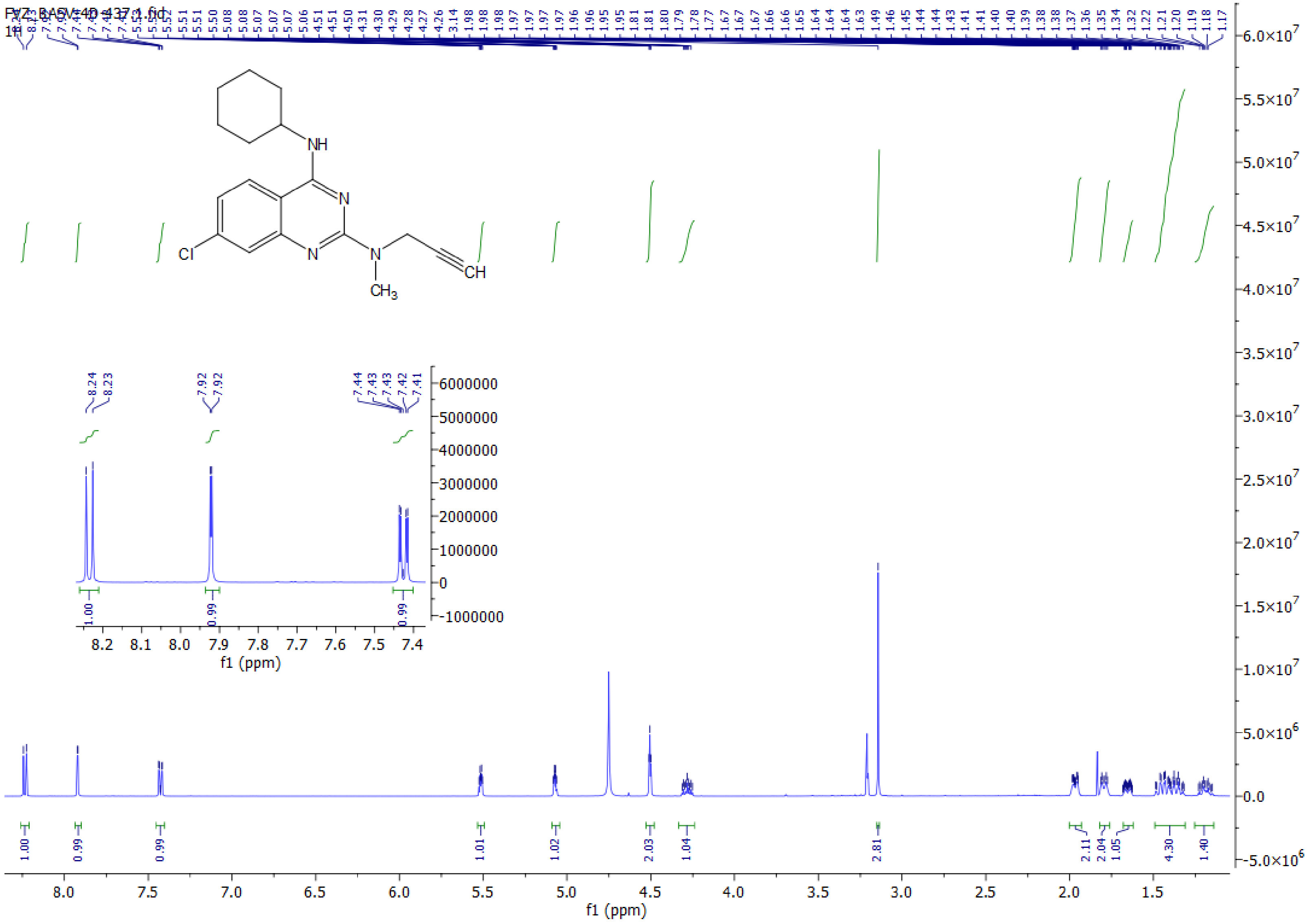

Fig. S73. ^{13}**C NMR of 7-chloro-*N4*-cyclohexyl-*N2*-methyl-*N2*-(prop-2-yn-1-yl)quinazoline-2,4-diamine (II-6h)**

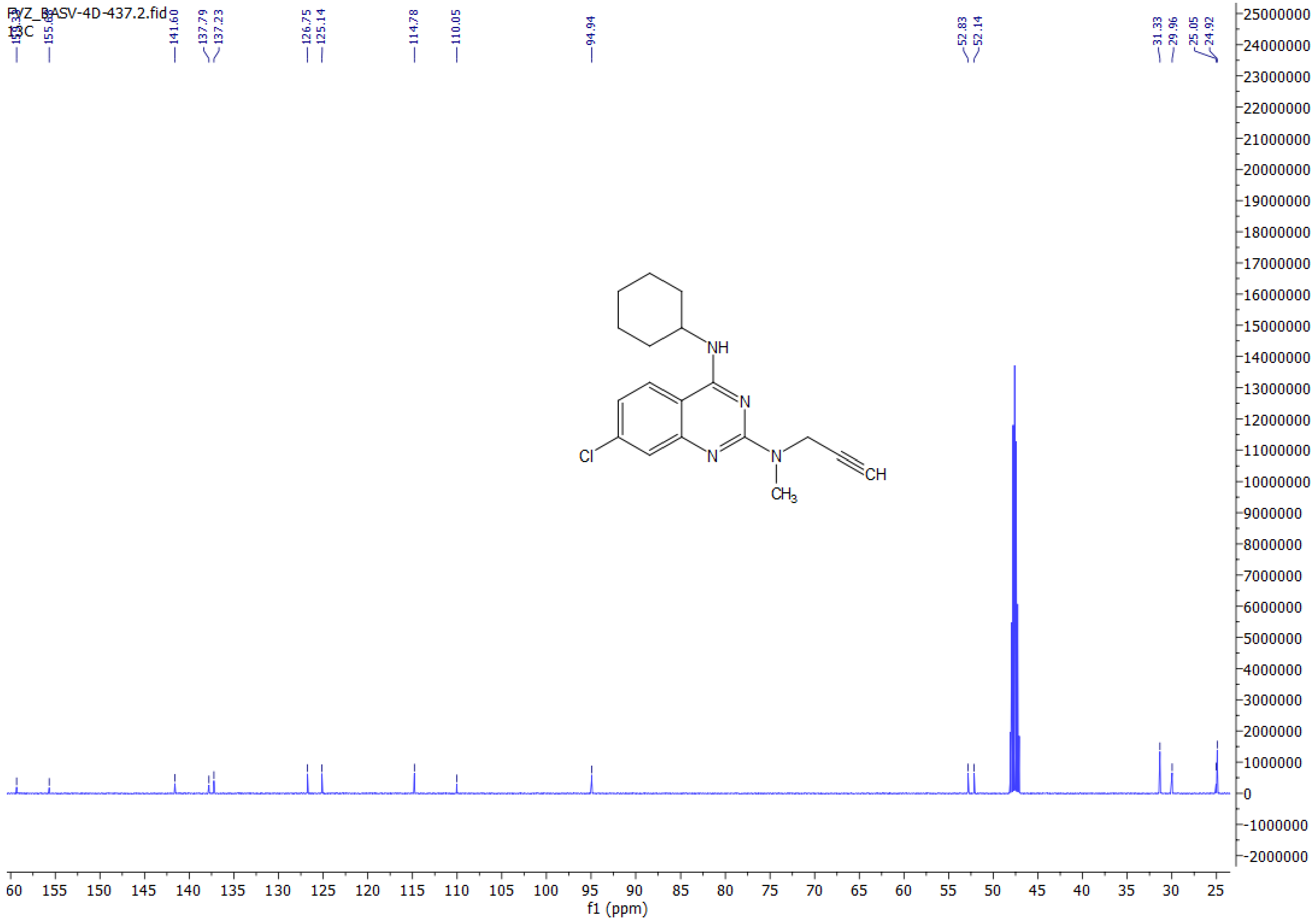

Fig. S74. ^{1}H NMR of 6-methoxy-*N2*-methyl-*N2*-(prop-2-yn-1-yl)quinazoline-2,4-diamine (III-6a)

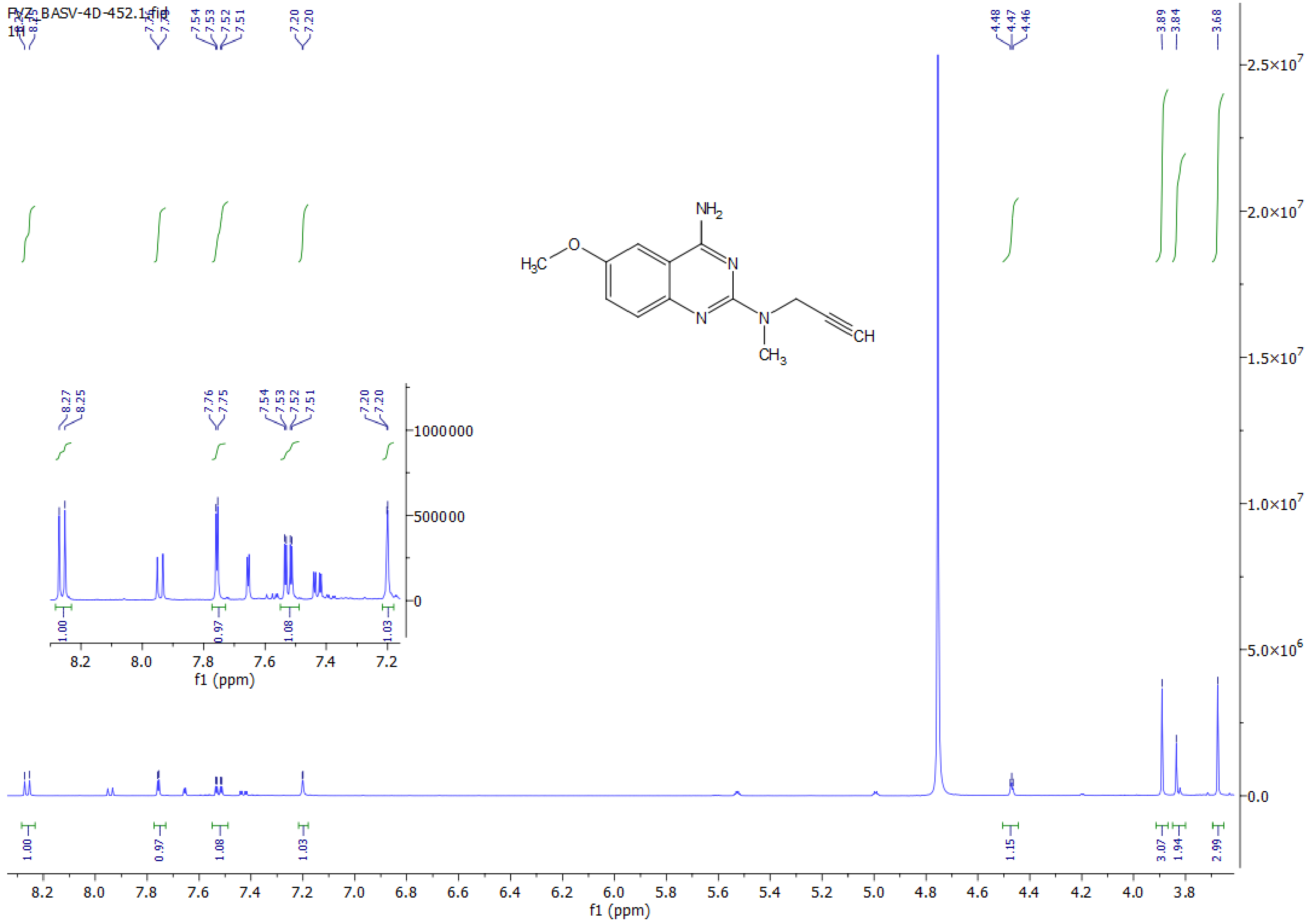

Fig. *S*75. ^{13}C NMR of 6-methoxy-*N2*-methyl-*N2*-(prop-2-yn-1-yl)quinazoline-2,4-diamine (III-6a)

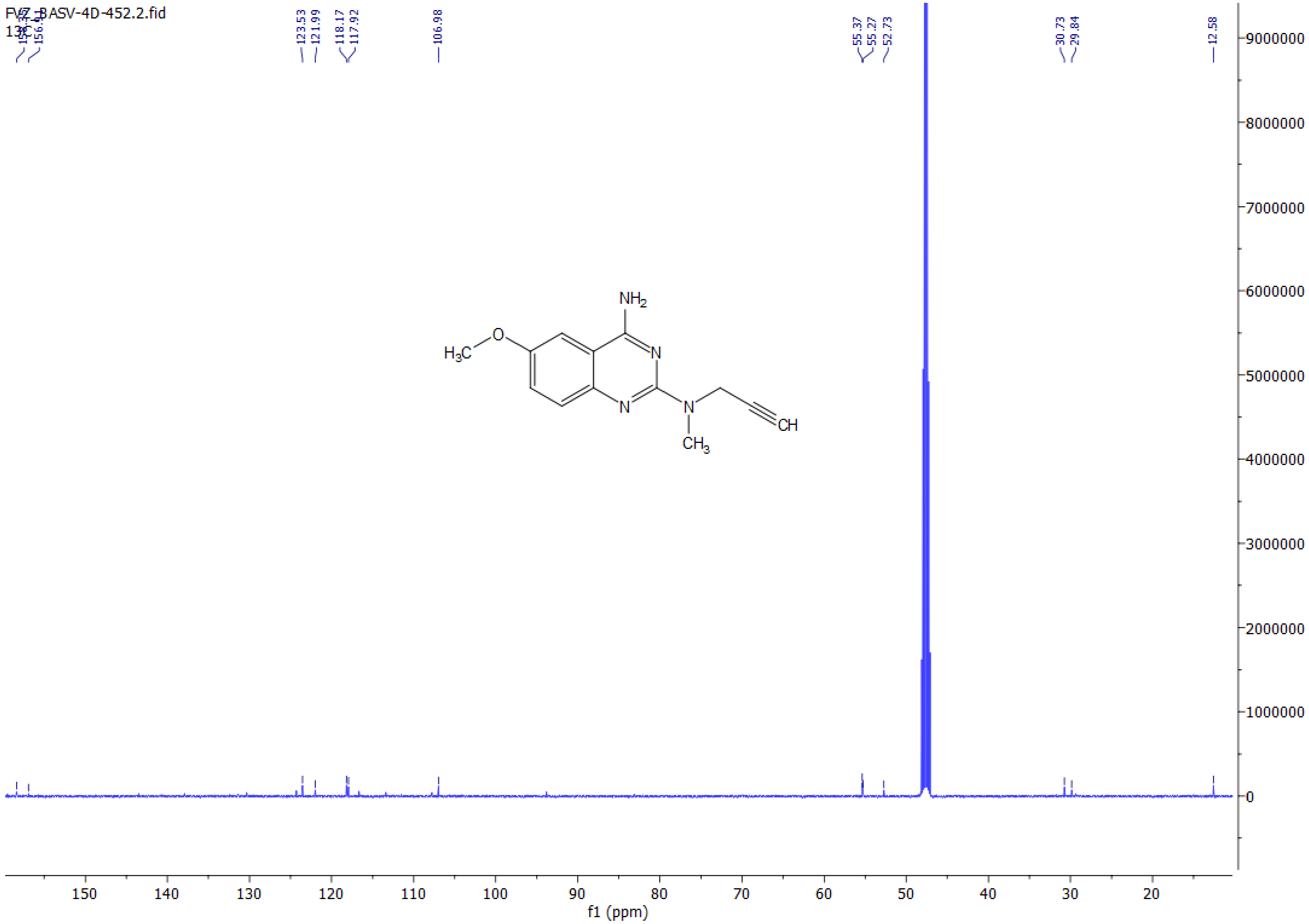

Fig. S76. ^{1}H NMR of 6-methoxy-*N2*,*N4*-dimethyl-*N2*-(prop-2-yn-1-yl)quinazoline-2,4-diamine (III-6b)

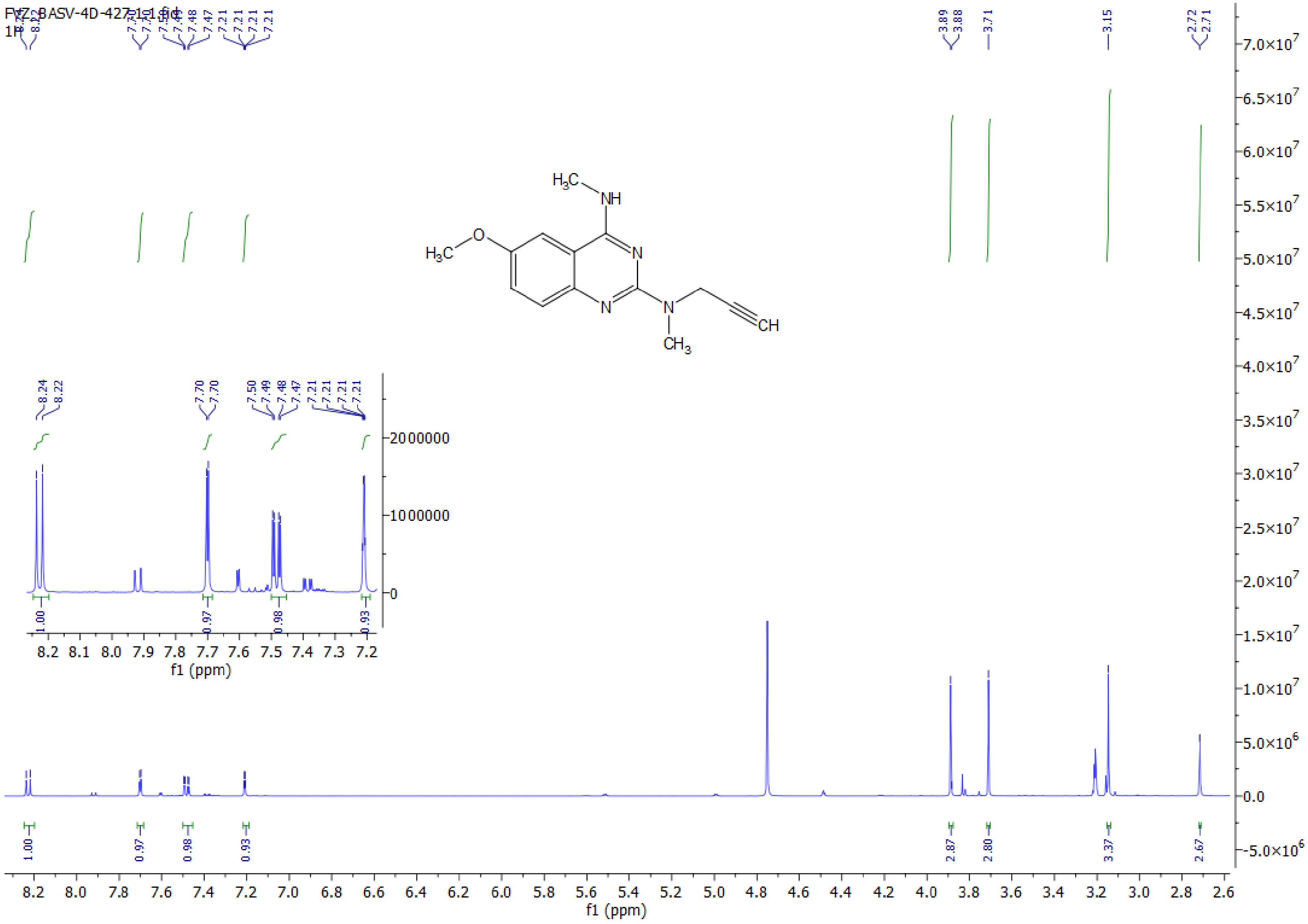

Fig. S77. ^{13}C NMR of 6-methoxy-*N2*,*N4*-dimethyl-*N2*-(prop-2-yn-1-yl)quinazoline-2,4-diamine (III-6b)

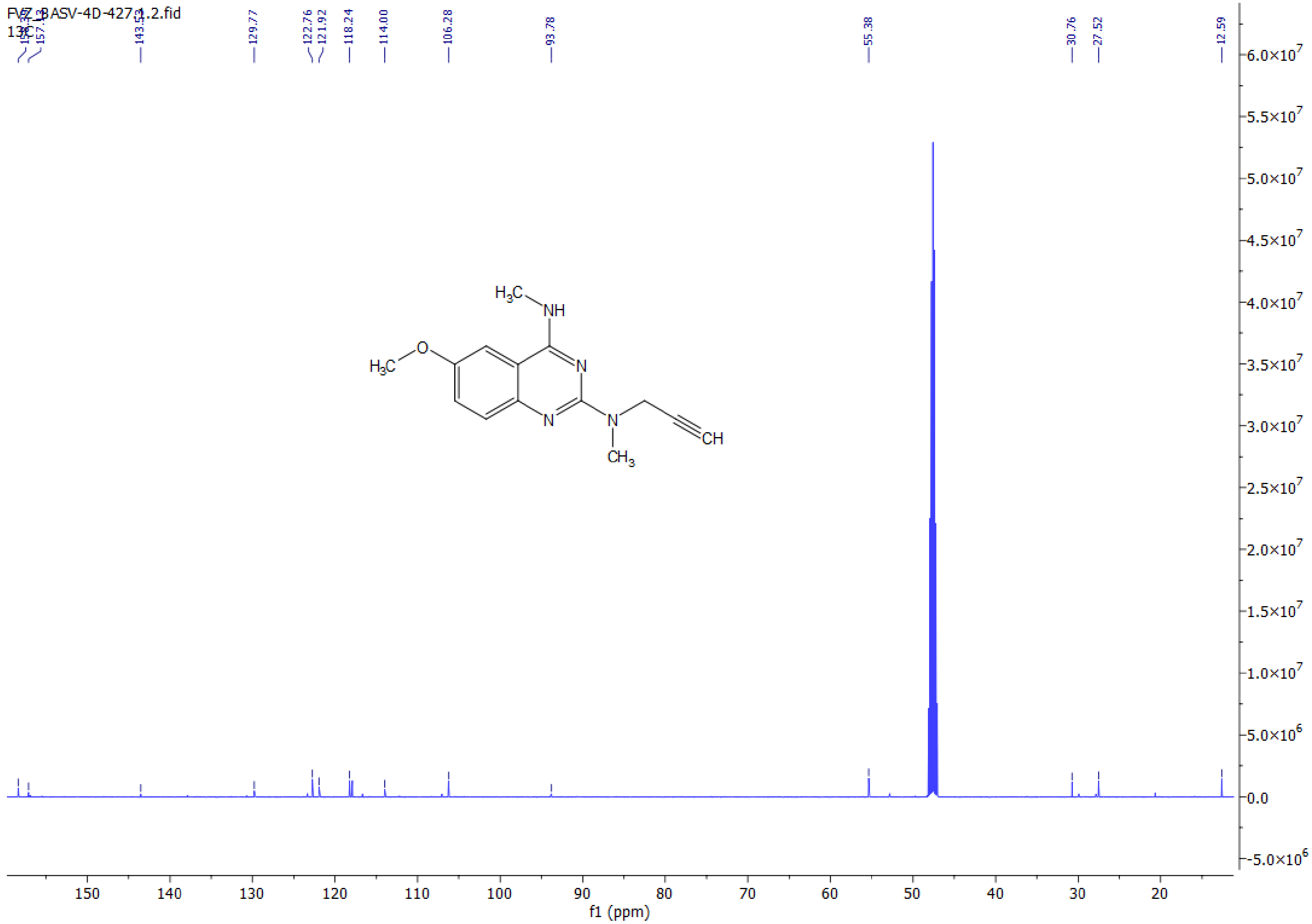

Fig. S78. ^{1}H NMR of *N4*-ethyl-6-methoxy-*N2*-methyl-*N2*-(prop-2-yn-1-yl)quinazoline-2,4-diamine (III-6c)

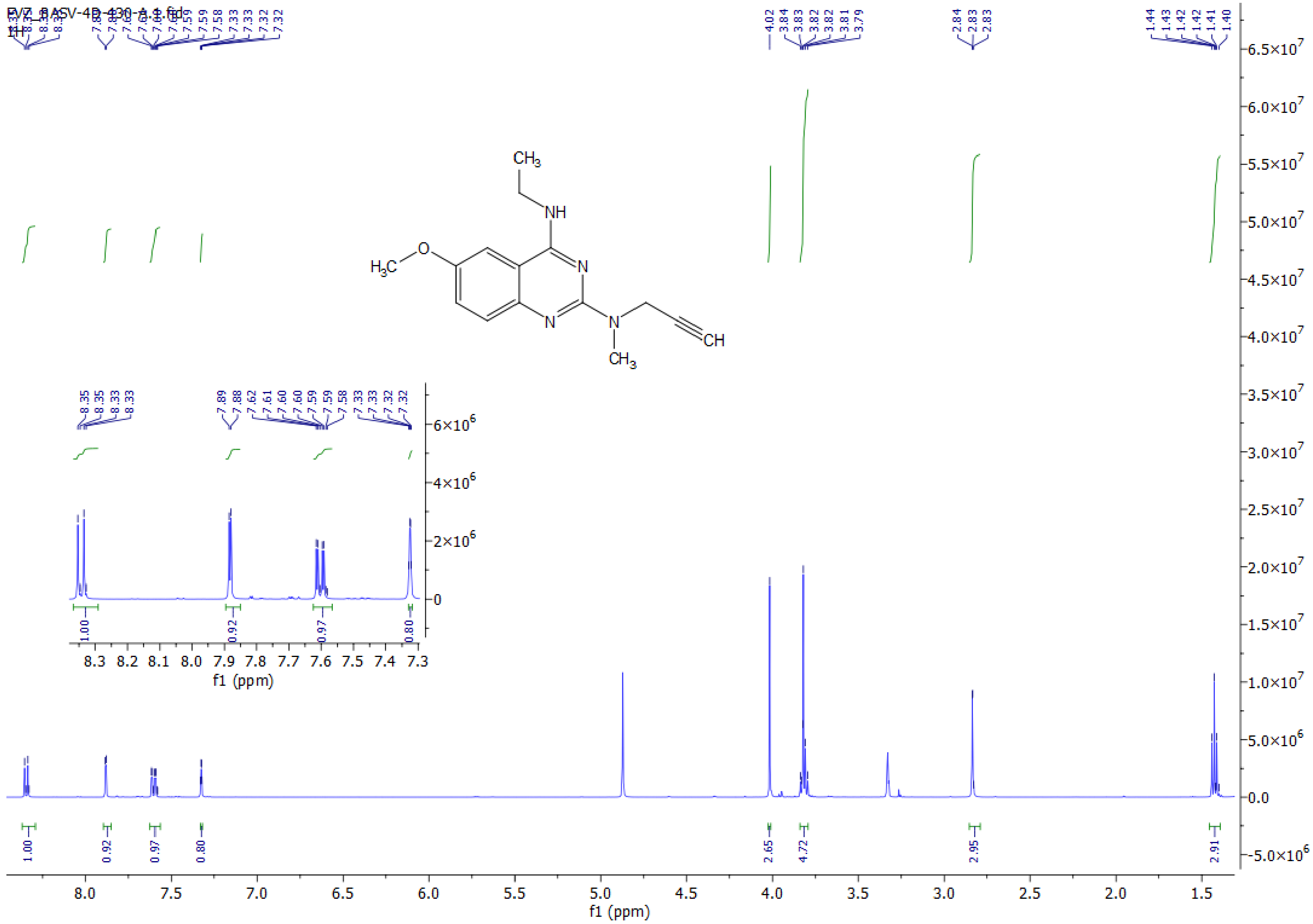

Fig. S79. ^{13}C NMR of *N4*-ethyl-6-methoxy-*N2*-methyl-*N2*-(prop-2-yn-1-yl)quinazoline-2,4-diamine (III-6c)

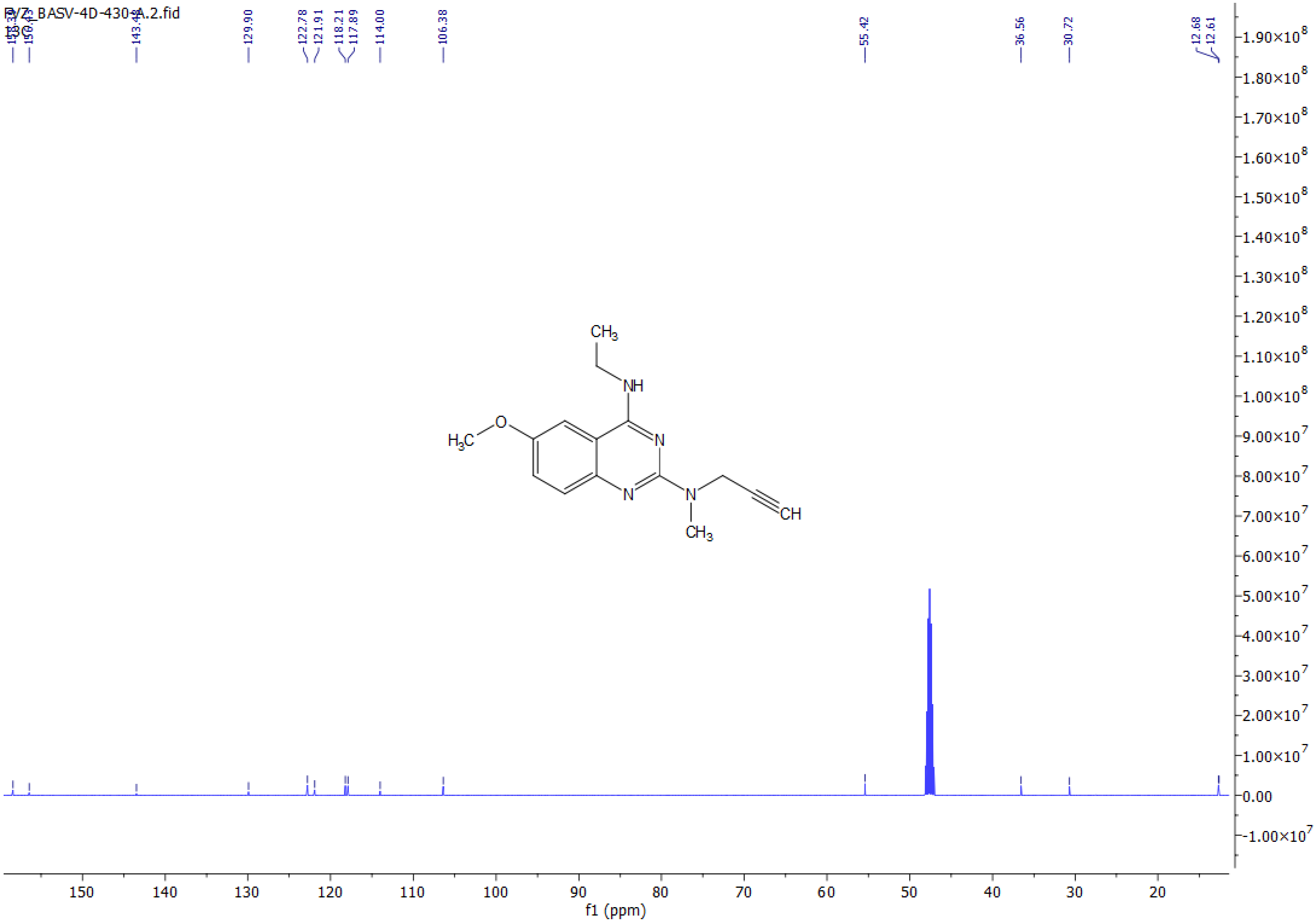

Fig. S80. ^{1}H NMR of -methoxy-*N2*-methyl-*N2*-(prop-2-yn-1-yl)-*N4*-(propan-2-yl)quinazoline-2,4-diamine (III-6d)

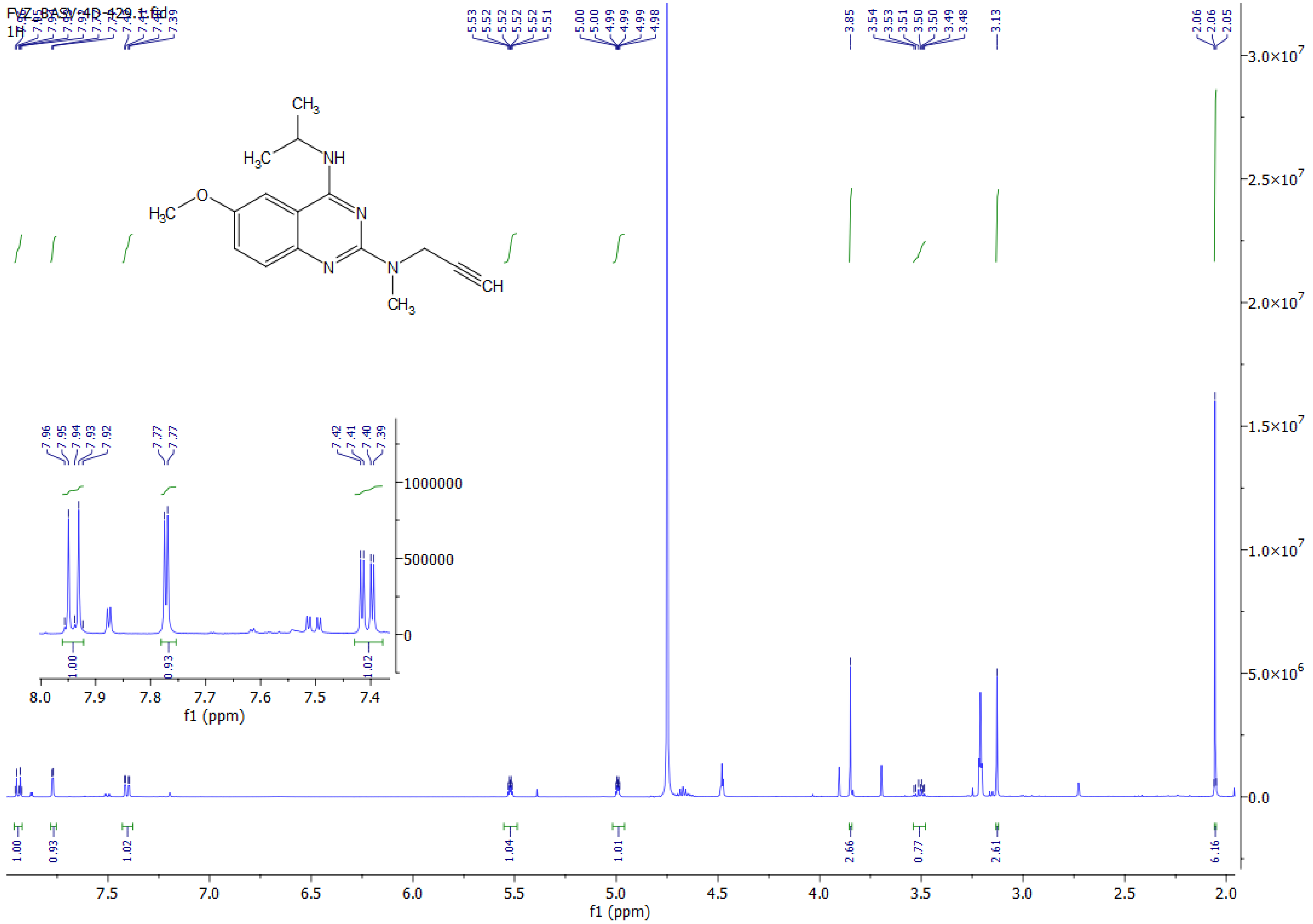

Fig. S81. ^{13}C NMR of -methoxy-*N2*-methyl-*N2*-(prop-2-yn-1-yl)-*N4*-(propan-2-yl)quinazoline-2,4-diamine (III-6d)

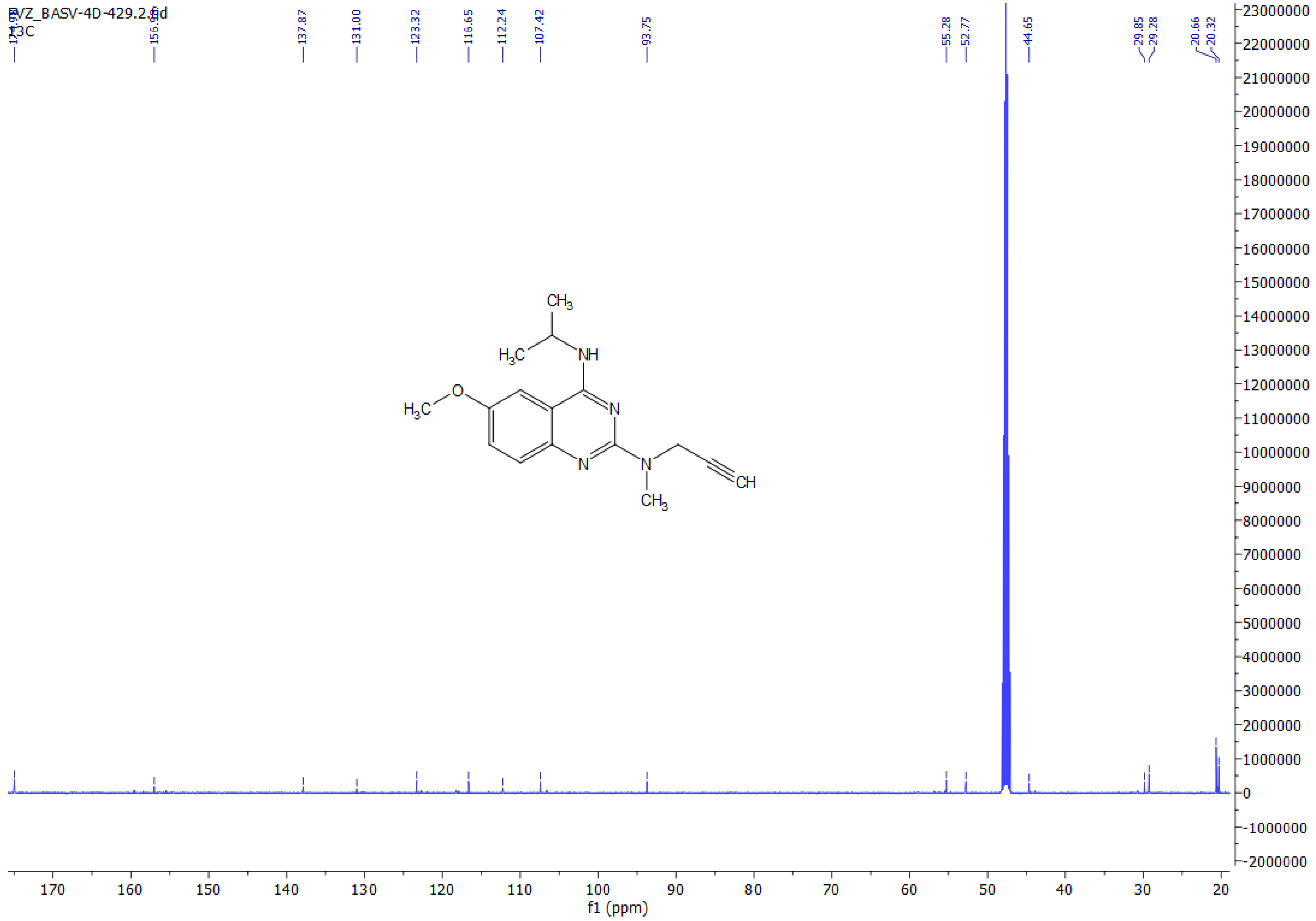

Fig. S82. ^{1}H NMR of *N4*-butyl-6-methoxy-*N2*-methyl-*N2*-(prop-2-yn-1-yl)quinazoline-2,4-diamine (III-6e)

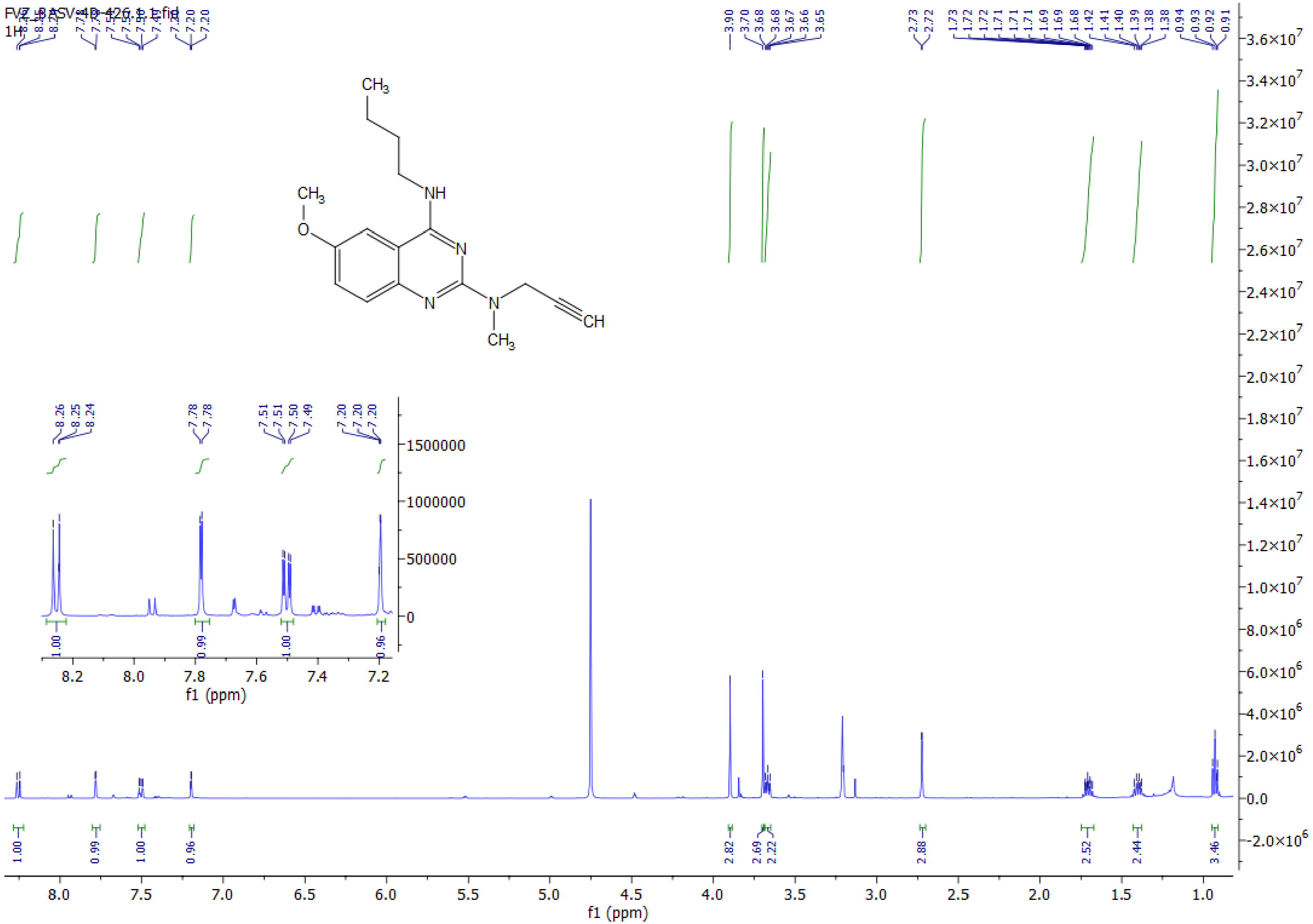

Fig. S83. ^{13}C NMR of *N4*-butyl-6-methoxy-*N2*-methyl-*N2*-(prop-2-yn-1-yl)quinazoline-2,4-diamine (III-6e)

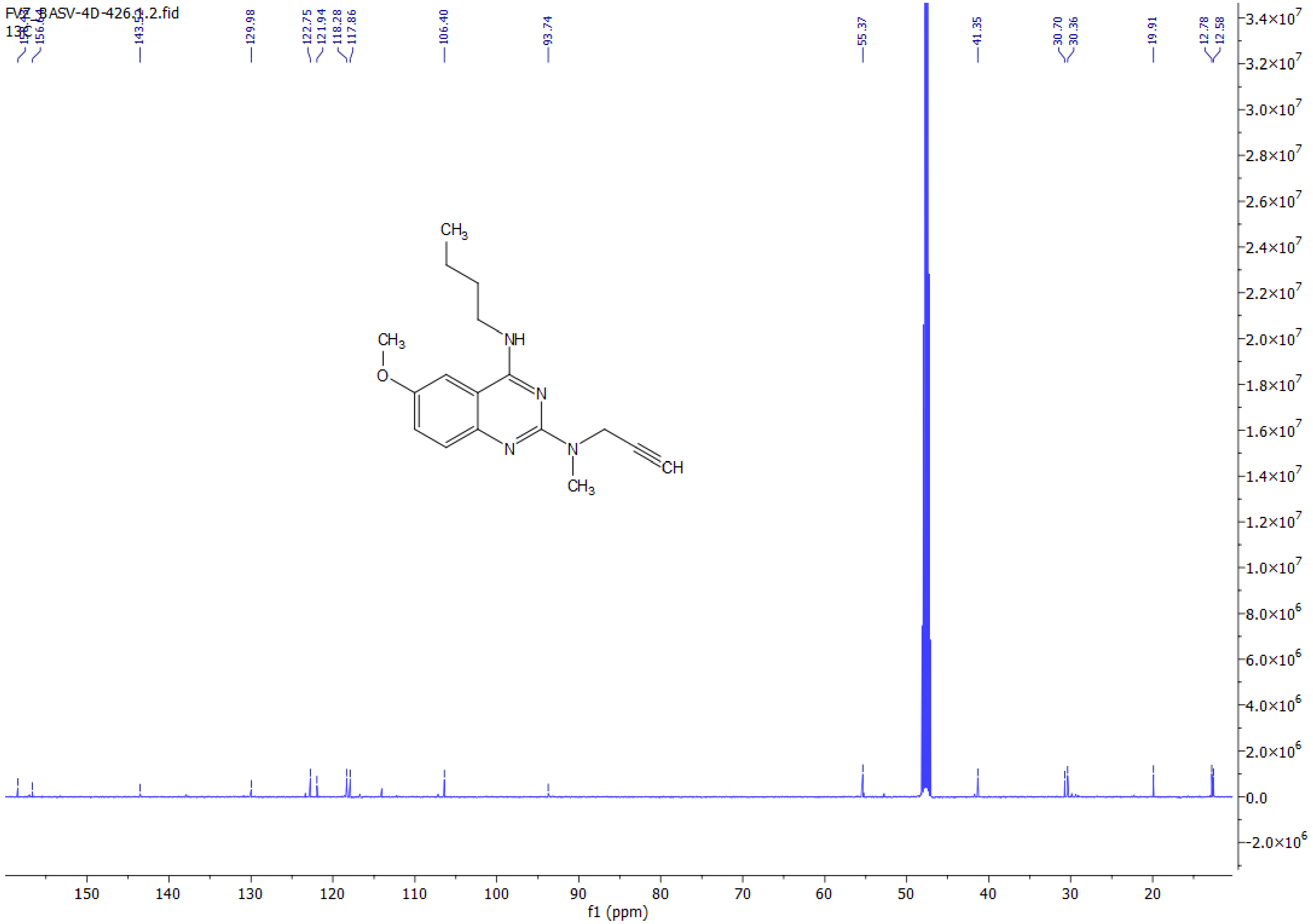

Fig. S84. ^{1}H NMR of 6-methoxy-*N4*-(2-methoxyethyl)-*N2*-methyl-*N2*-(prop-2-yn-1-yl)quinazoline-2,4-diamine (III-6f)

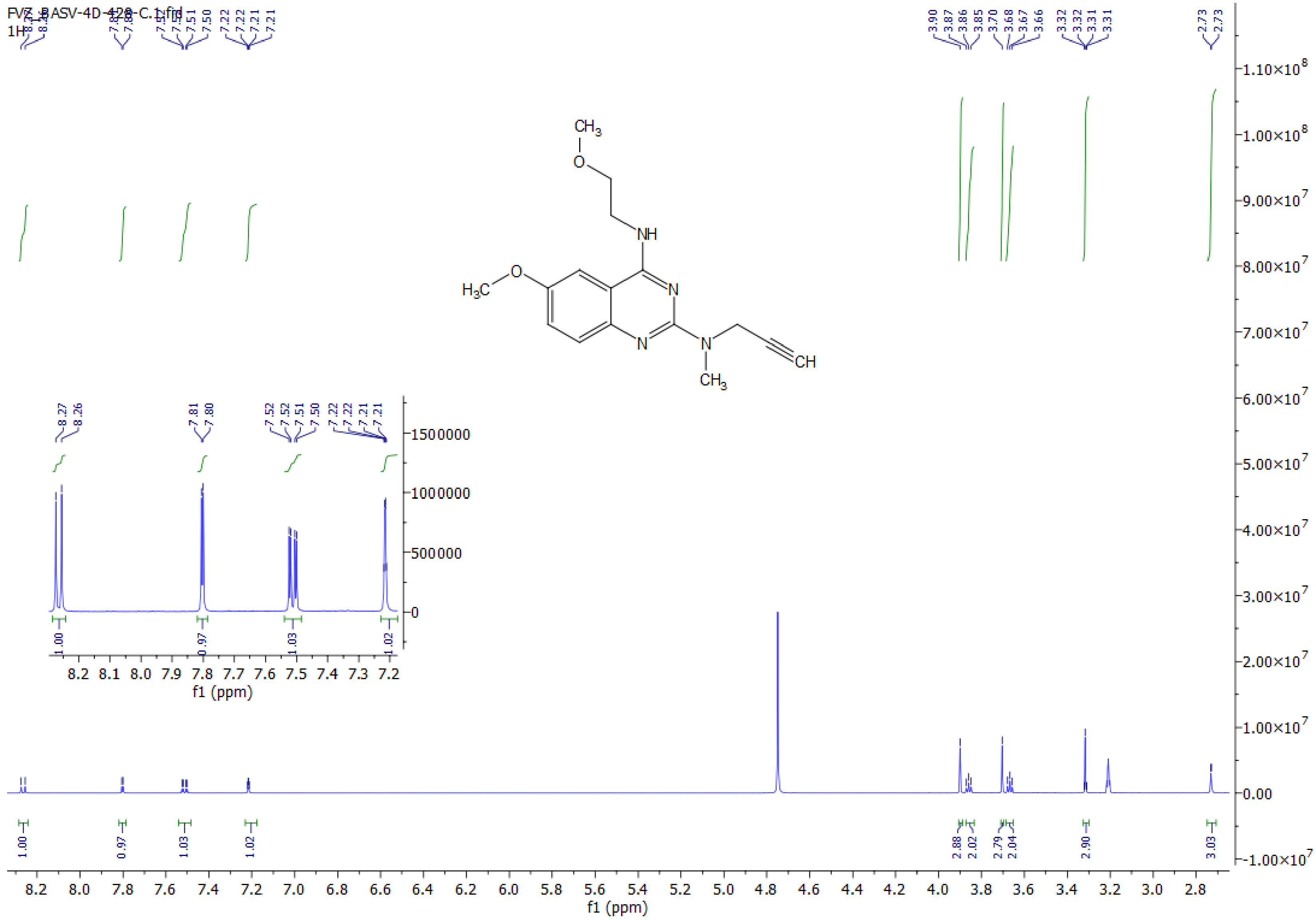

Fig. S85. ^{13}C **NMR of 6-methoxy-*N4*-(2-methoxyethyl)-*N2*-methyl-*N2*-(prop-2-yn-1-yl)quinazoline-2,4-diamine (III-6f)**

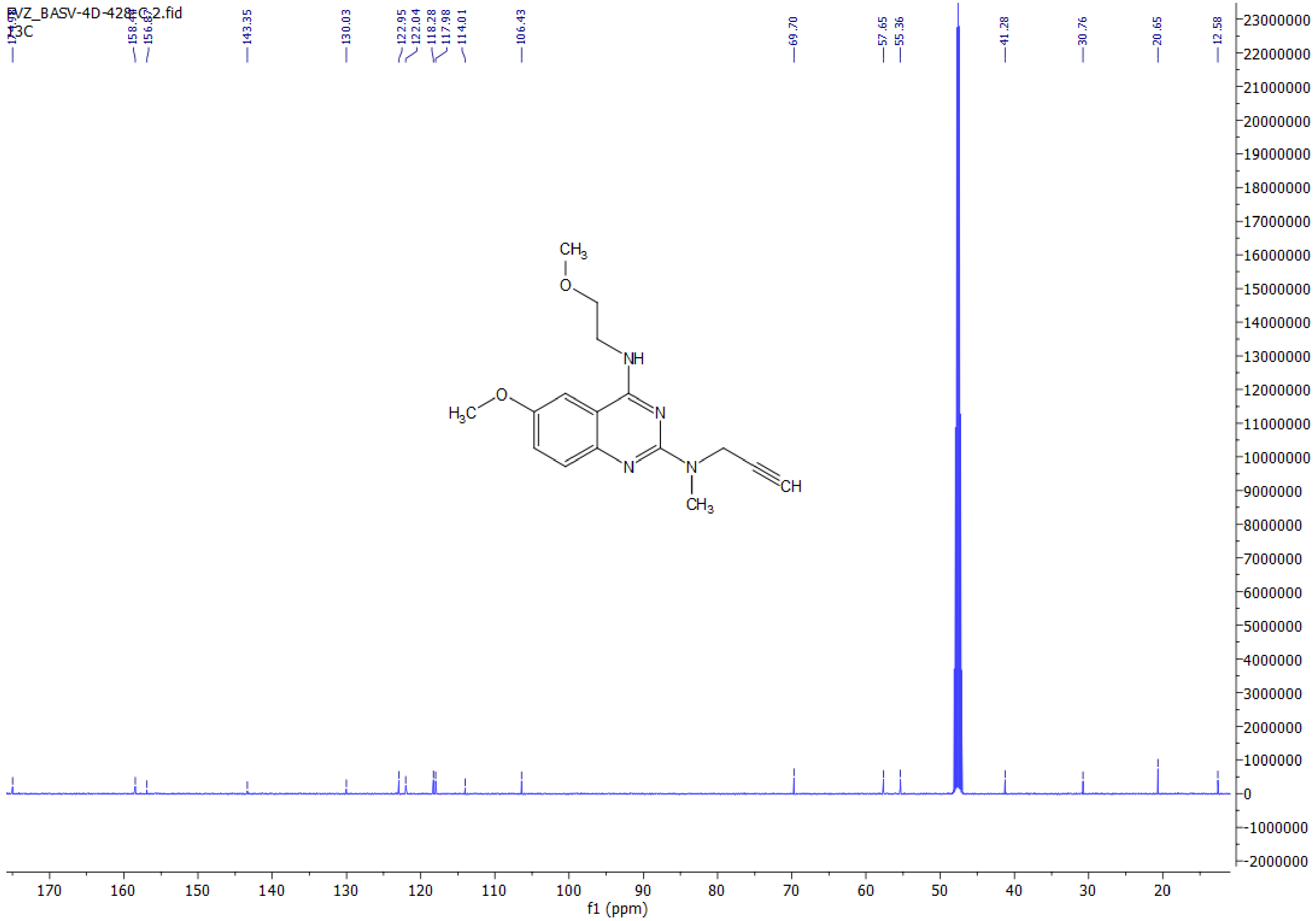

Fig. S86. ^{1}H **NMR of *N4*-cyclopropyl-6-methoxy-*N2*-methyl-*N2*-(prop-2-yn-1-yl)quinazoline-2,4-diamine (III-6g)**

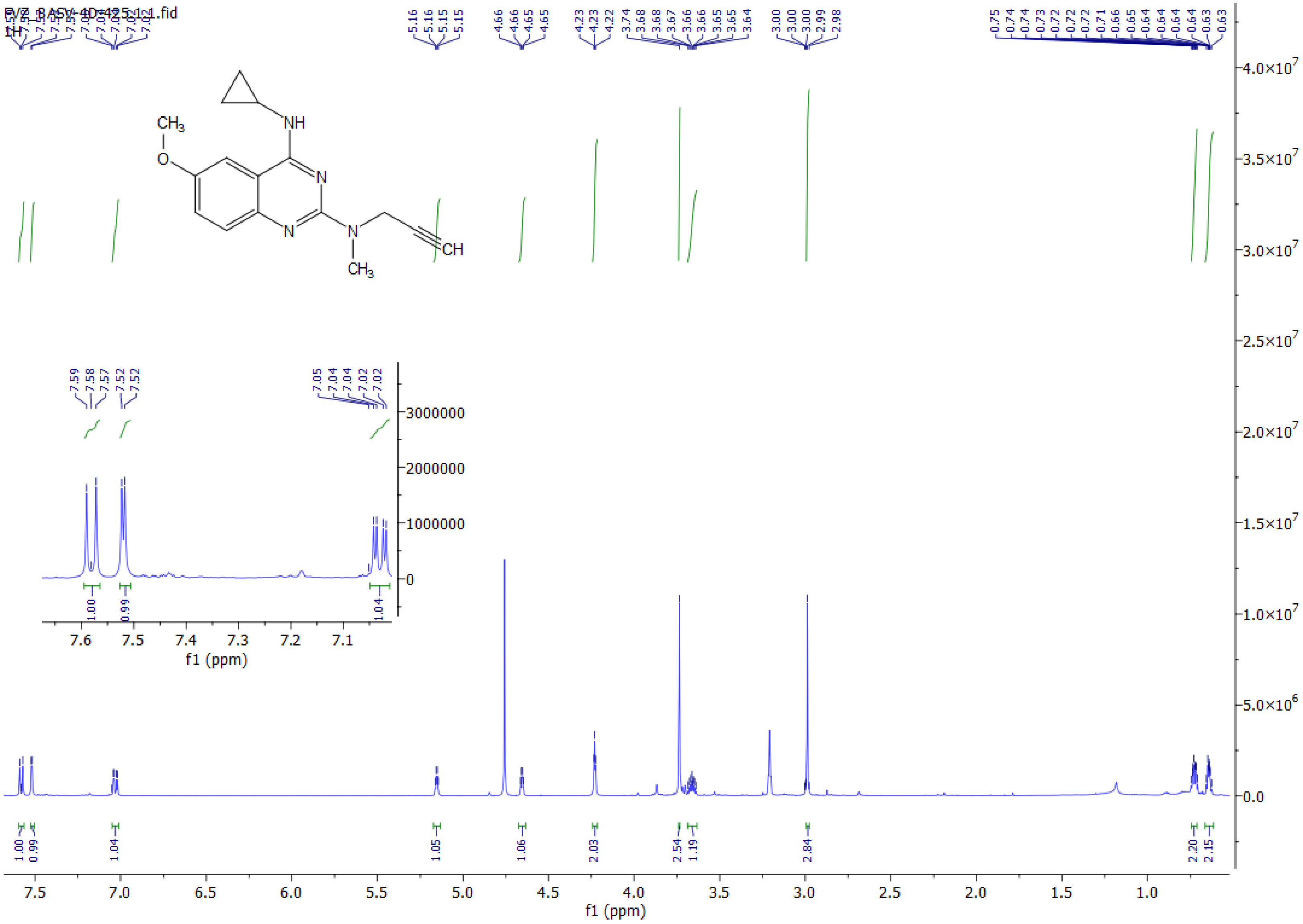

Fig. S87. ^{13}C NMR of *N4*-cyclopropyl-6-methoxy-*N2*-methyl-*N2*-(prop-2-yn-1-yl)quinazoline-2,4-diamine (III-6g)

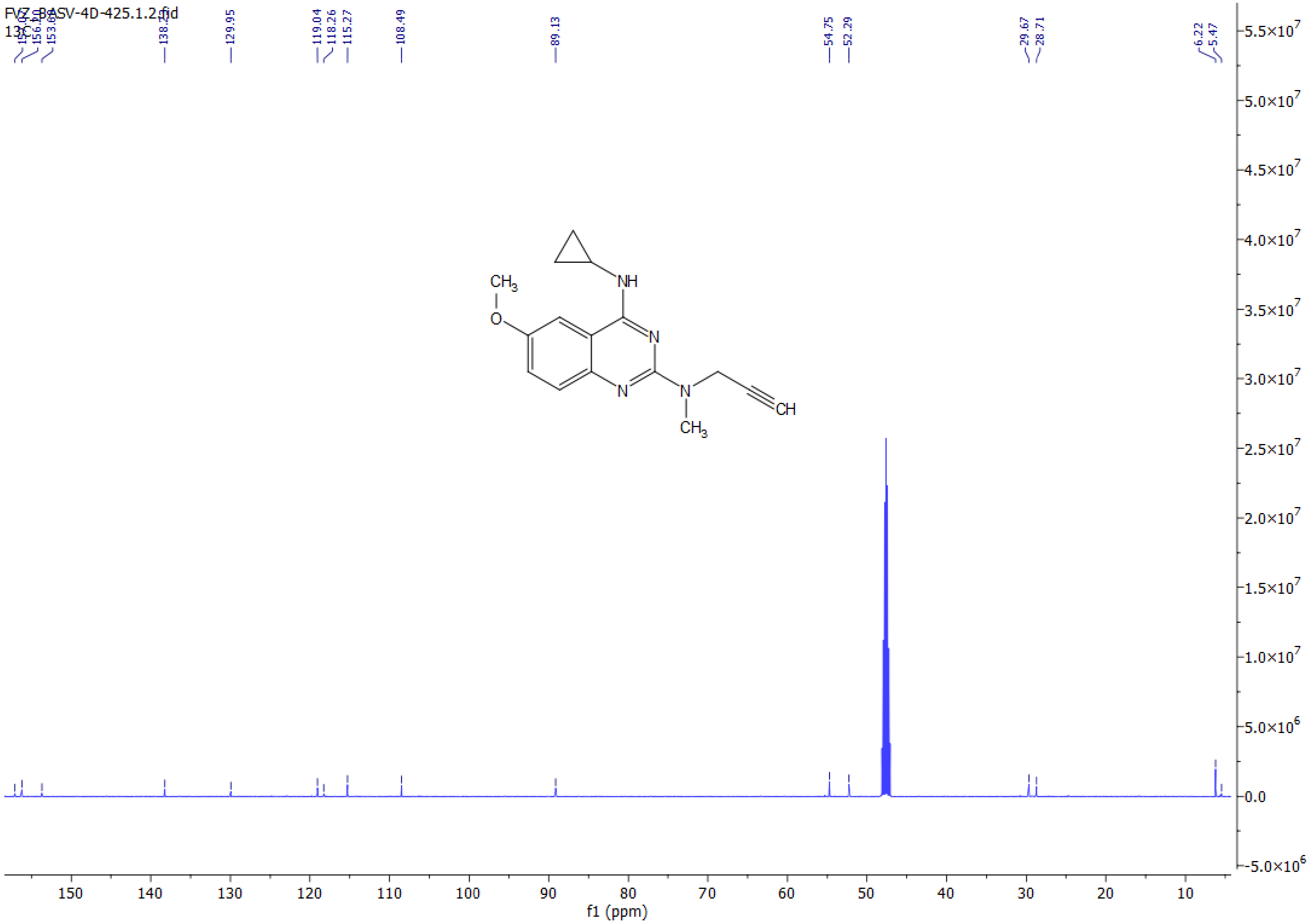

Fig. S88. ^{1}H NMR of *N4*-cyclohexyl-6-methoxy-*N2*-methyl-*N2*-(prop-2-yn-1-yl)quinazoline-2,4-diamine (III-6h)

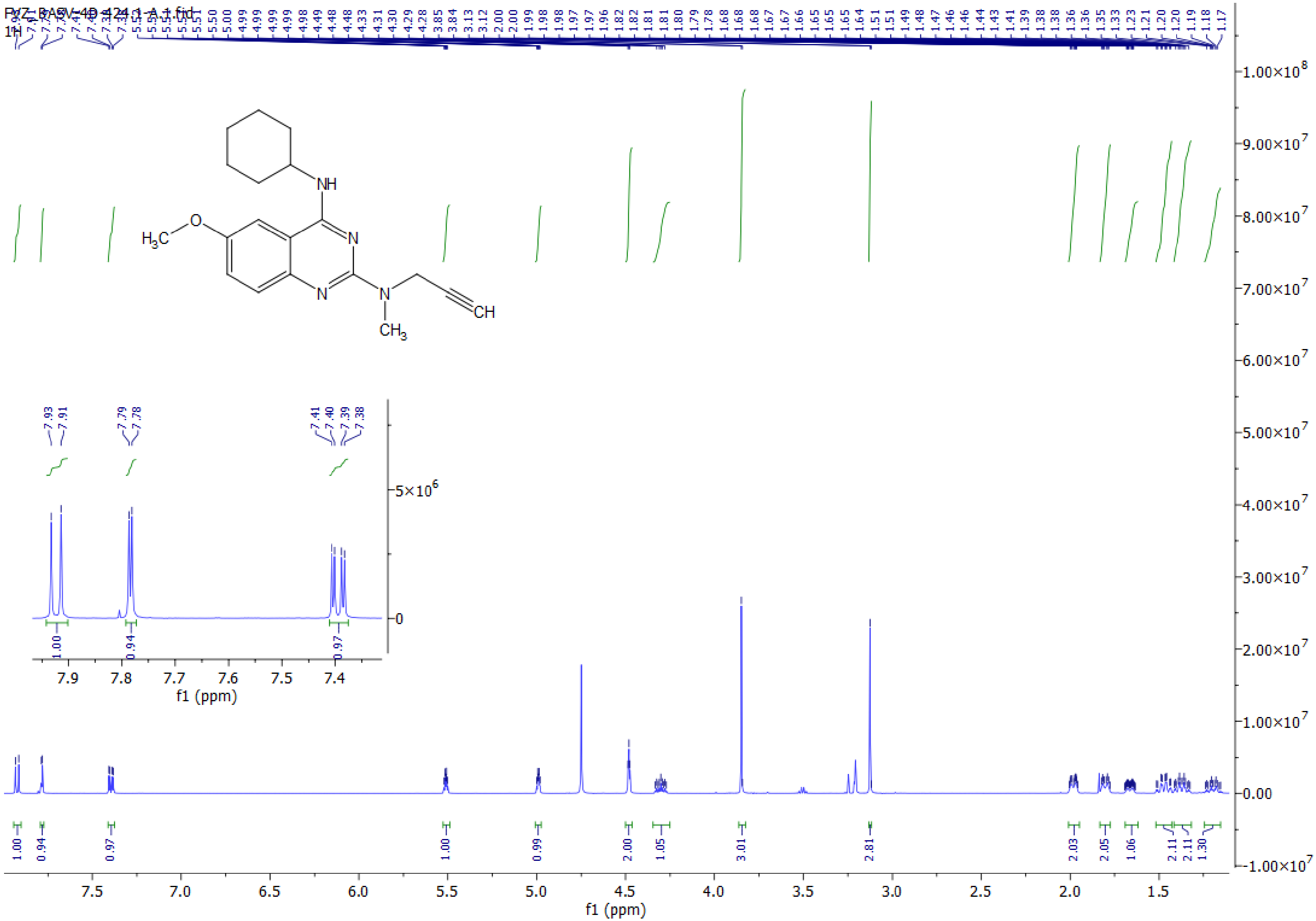

Fig. S89. ^{13}C **NMR of *N4*-cyclohexyl-6-methoxy-*N2*-methyl-*N2*-(prop-2-yn-1-yl)quinazoline-2,4-diamine (III-6h)**

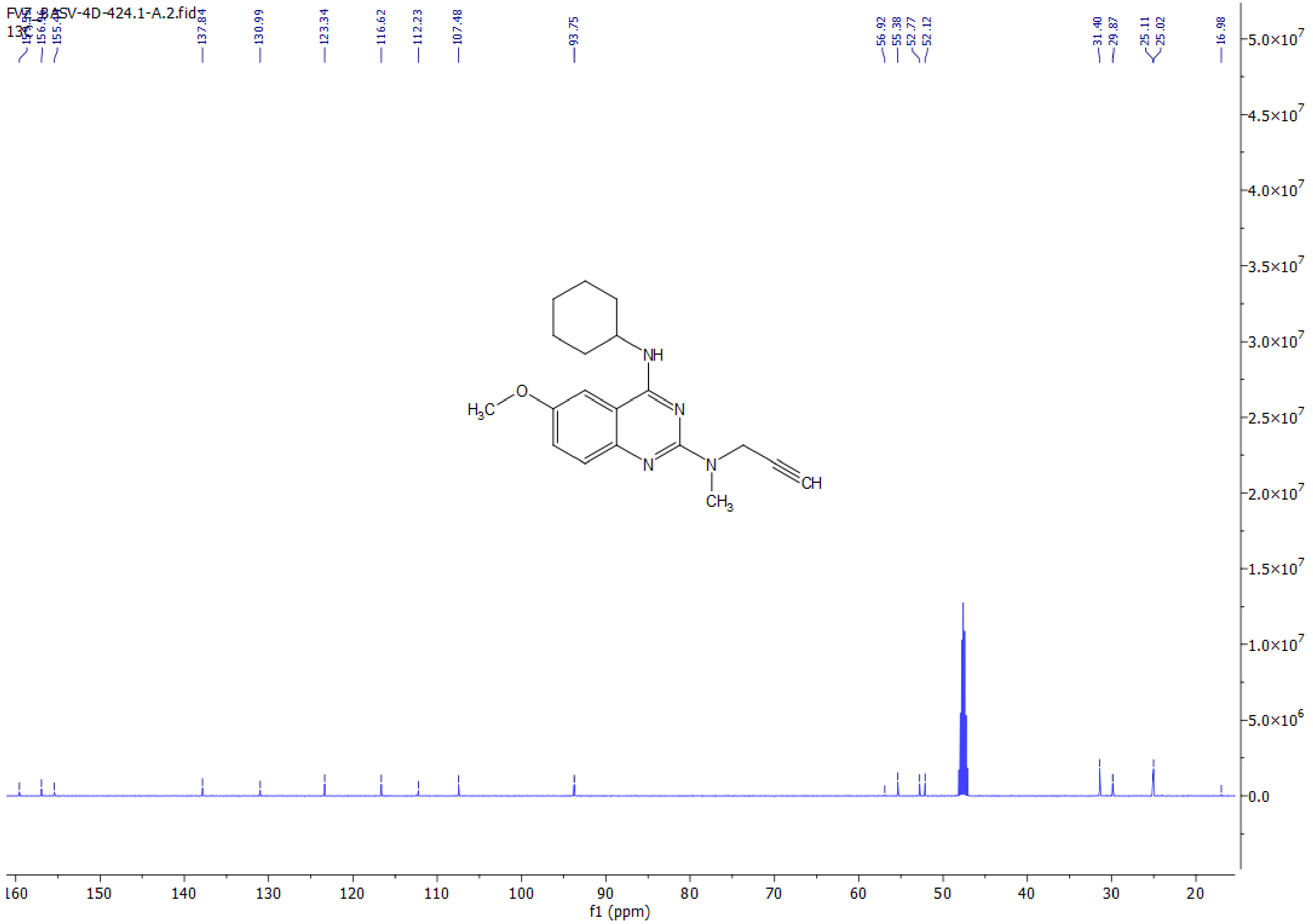

5. HPLC Analysis of Compounds

High performance liquid chromatography (HPLC) with mass spectrometry (MS) was used to determine high resolution mass spectra (HRMS) and purity of compounds.

LC-MS Instrumentation

The system used in this study was Dionex Ultimate 3000 UHPLC: RS LPG quaternary Pump, RS Column Compartment, RS Autosampler, Diode Array Detector, Chromeleon (version 7.2.9 build 11323) software with Q Exactive Plus Orbitrap mass spectrometer with Thermo Xcalibur (version 4.3.73.11) software (Thermo Fisher Scientific, Waltham, MA, USA). Settings of heated electrospray source were: Spray voltage 3.5 kV, Capillary temperature: 260 °C, Sheath gas: 50 arbitrary units, Auxiliary gas: 12.5 arbitrary units, Spare gas: 2.5 arbitrary units, Probe heater temperature: 300 °C, Max spray current: 100 μA, S-lens RF Level: 50.

HRMS and purity determination

High resolution mass spectra and sample purities were obtained by HPLC gradient method with detection by UV and MS. C18 column (Kinetex EVO C18, 2.1x50 mm, 1.7 μm, Phenomenex, Torrance, CA, USA) was used in this study. Mobile phase A was ultrapure water of ASTM I type (resistance 18.2 MΩ.cm at 25°C) prepared by Barnstead Smart2Pure 3 UV/UF apparatus (Thermo Fisher Scientific, Waltham, MA, USA) with 0.1 % (v/v) formic acid (LC-MS grade, VWR, Radnor, PA, USA); mobile phase B was acetonitrile (MS grade, VWR, Radnor, PA, USA) with 0.1 % (v/v) of formic acid. The flow was constant at 0.4 ml/min. Method started with 0.3 minute of isocratic flow of 5 % B, then the gradient of B rose to 100 % B in 3 min and remained constant at 100 % B for 0.7 min. Then the composition went back to 5 % B and equilibrated for 3.5 min. Samples were dissolved in methanol (LC-MS grade, VWR, Radnor, PA, USA) with concentration 1 mg/mL and sample injection was 1 μL. Purity was determined from UV spectra measured at wavelength 254 nm. HRMS was determined in total ion current spectra from mass spectrometer in positive mode.

Fig. S90. **HRMS spectrum for *N2*-methyl-*N2*-(prop-2-yn-1-yl)quinazoline-2,4-diamine (I-6a)**

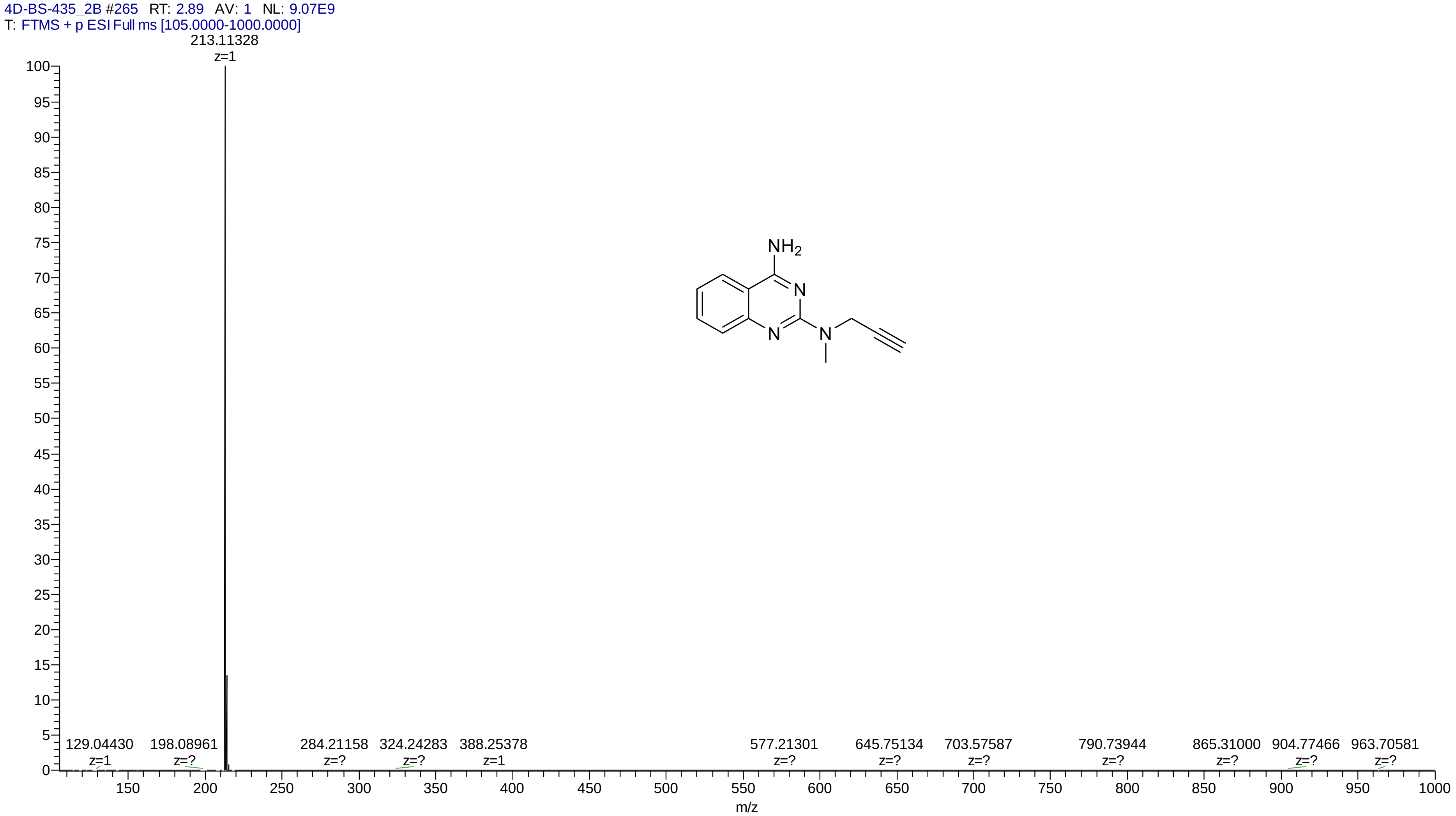

Fig. S91. **LC-UV chromatogram for *N2*-methyl-*N2*-(prop-2-yn-1-yl)quinazoline-2,4-diamine (I-6a)**

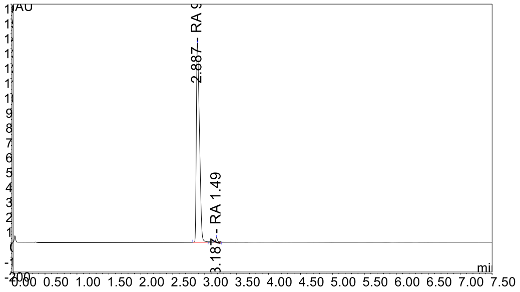

Fig. S92. **HRMS spectrum for *N2,N4*-dimethyl-*N2*-(prop-2-yn-1-yl)quinazoline-2,4-diamine (I-6b)**

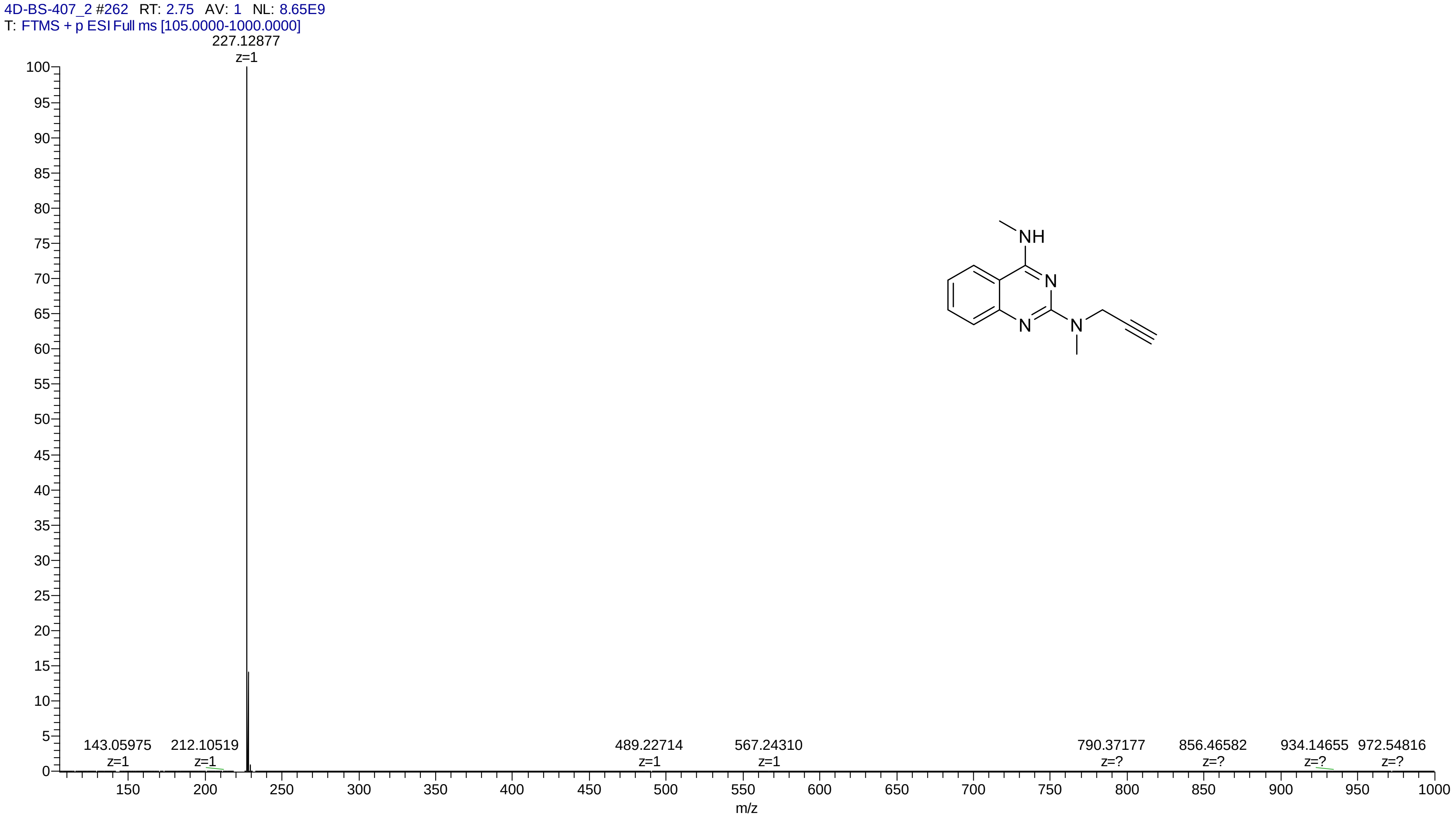

Fig. S93. **LC-UV chromatogram for *N2*,*N4*-dimethyl-*N2*-(prop-2-yn-1-yl)quinazoline-2,4-diamine (I-6b)**

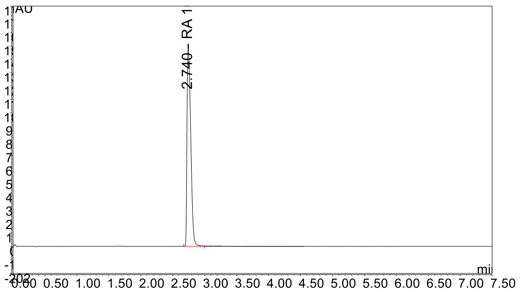

Fig. S94. HRMS spectrum for *N4*-ethyl-*N2*-methyl-*N2*-(prop-2-yn-1-yl)quinazoline-2,4-diamine (I-6c)

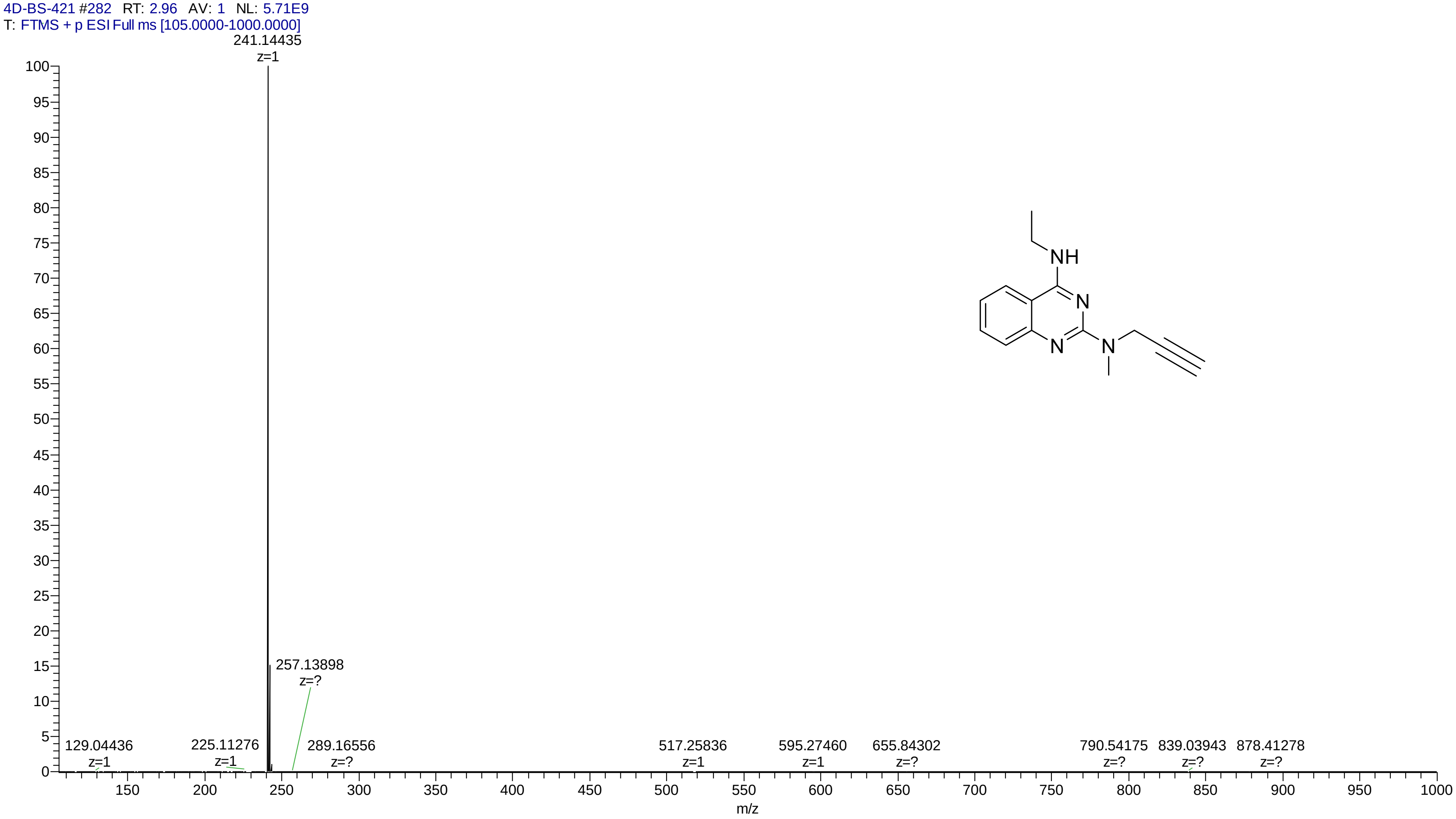

Fig. S95. **LC-UV chromatogram for *N4*-ethyl-*N2*-methyl-*N2*-(prop-2-yn-1-yl)quinazoline-2,4-diamine (I-6c)**

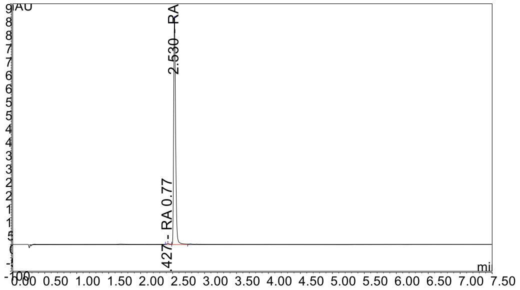

Fig. S96. **HRMS spectrum for *N2*-methyl-*N2*-(prop-2-yn-1-yl)-*N4*-(propan-2-yl)quinazoline-2,4-diamine (I-6d)**

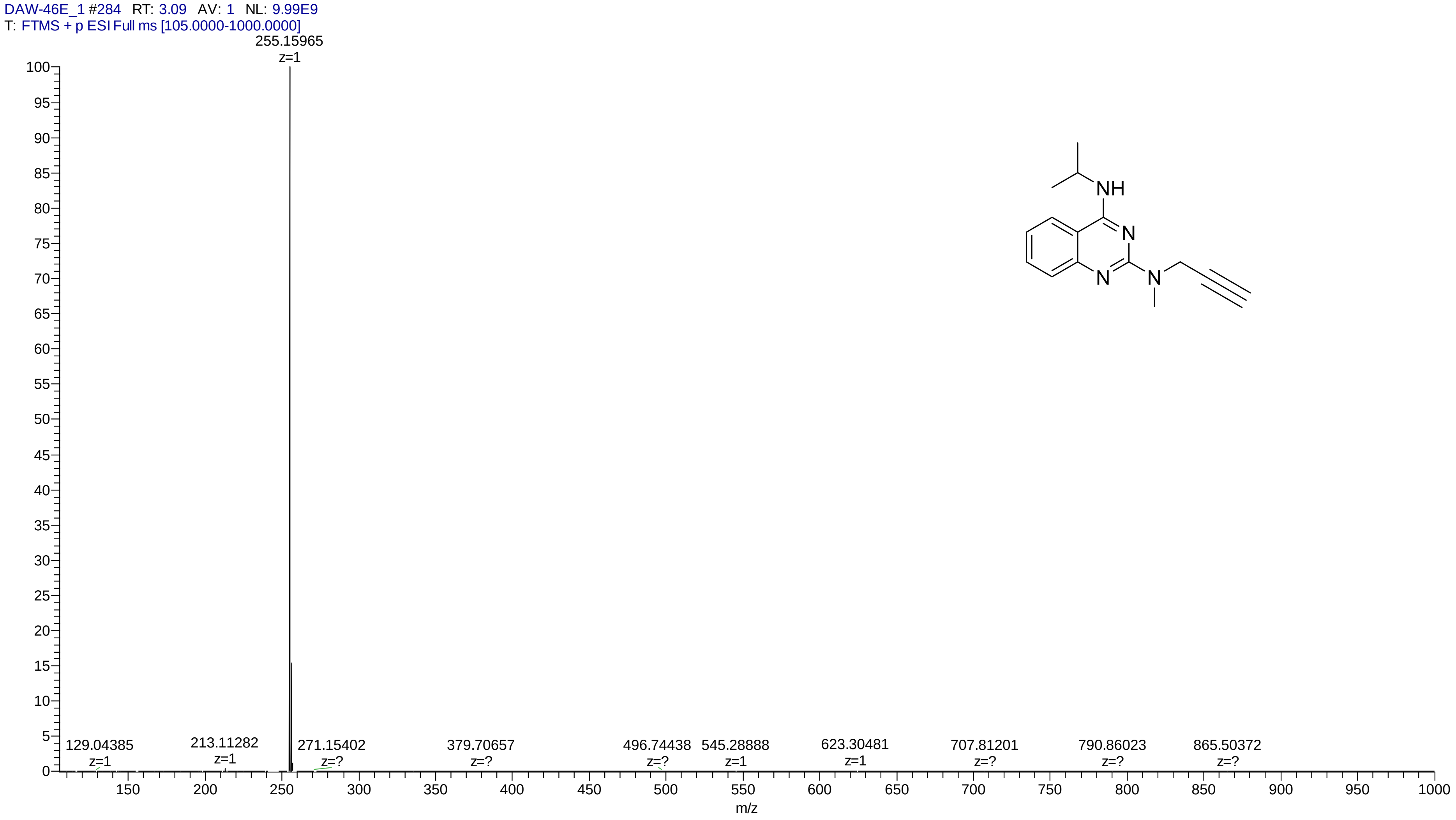

Fig. S97. **LC-UV chromatogram for *N2*-methyl-*N2*-(prop-2-yn-1-yl)-*N4*-(propan-2-yl)quinazoline-2,4-diamine (I-6d)**

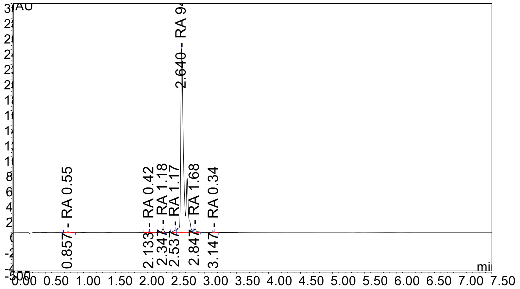

Fig. S98. **HRMS spectrum for *N4*-butyl-*N2*-methyl-*N2*-(prop-2-yn-1-yl)quinazoline-2,4-diamine (I-6e)**

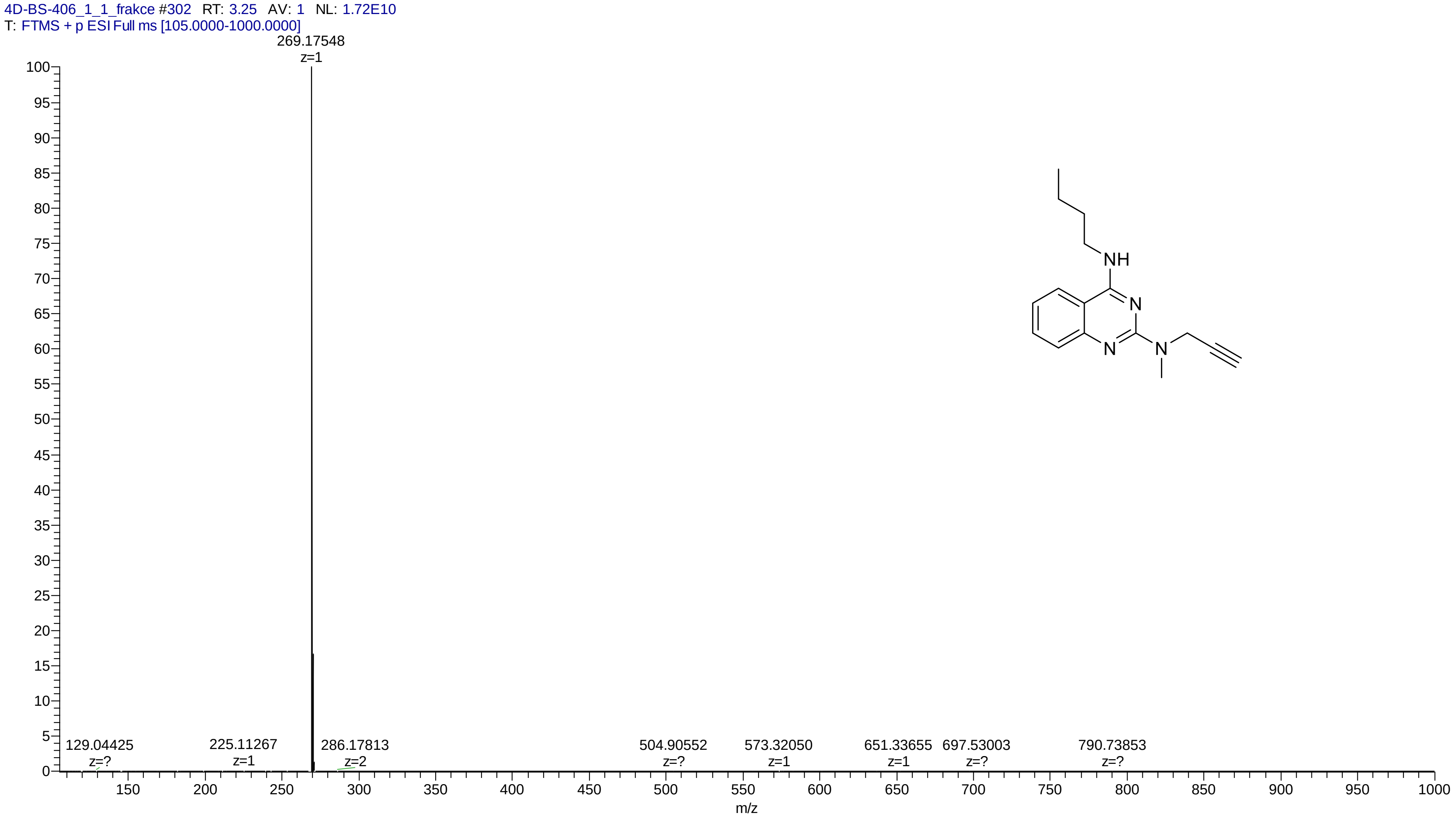

Fig. S99. **LC-UV chromatogram for *N4*-butyl-*N2*-methyl-*N2*-(prop-2-yn-1-yl)quinazoline-2,4-diamine (I-6e)**

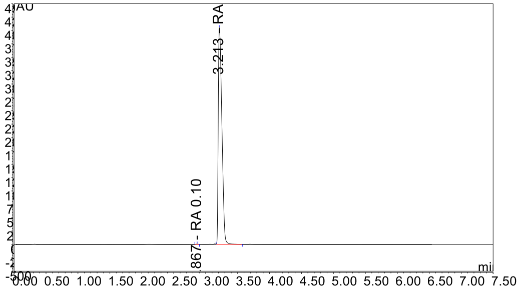

Fig. S100. HRMS spectrum for *N4*-(2-methoxyethyl)-*N2*-methyl-*N2*-(prop-2-yn-1-yl)quinazoline-2,4-diamine (I-6f)

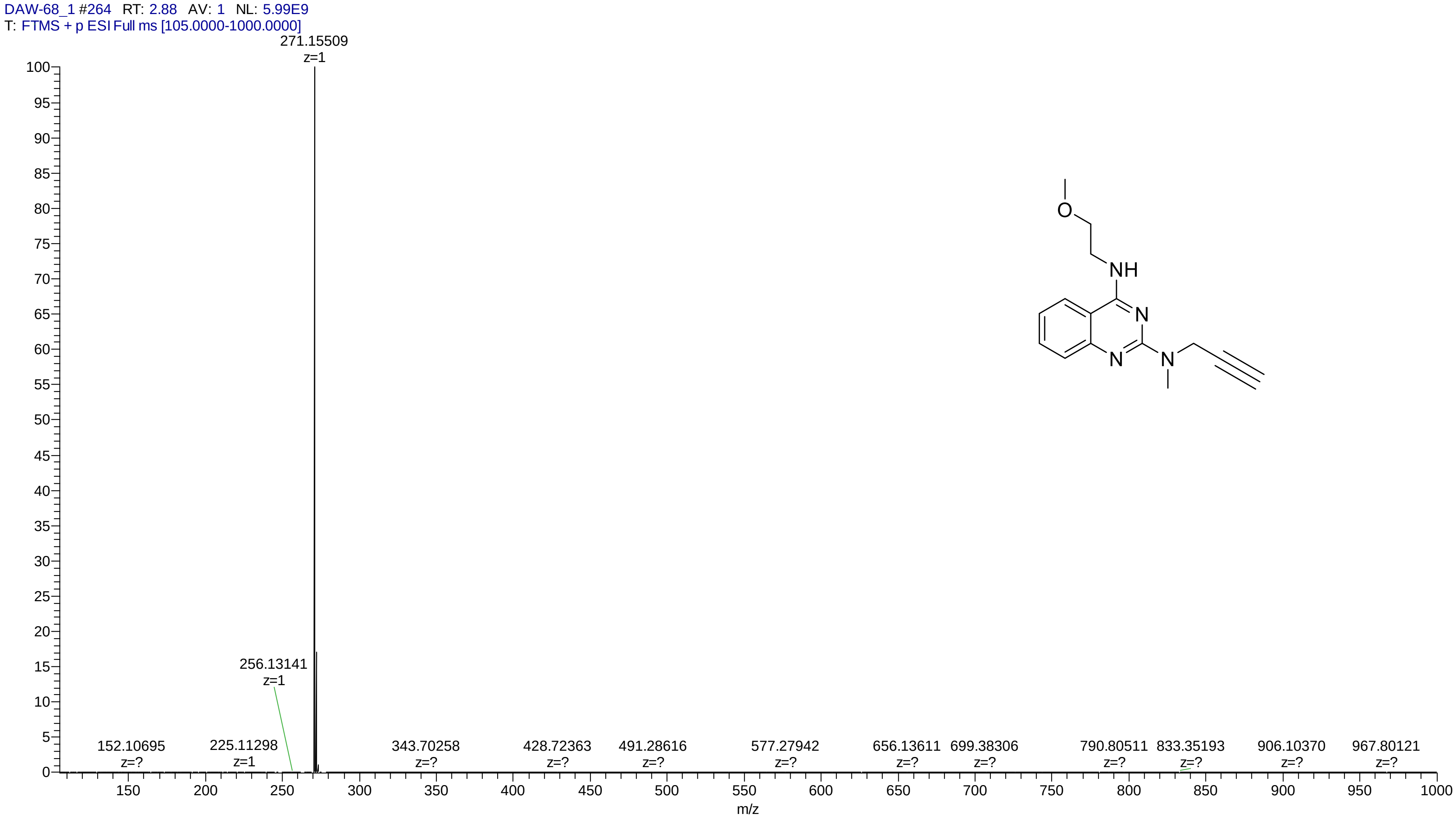

Fig. S101. **LC-UV chromatogram for *N4*-(2-methoxyethyl)-*N2*-methyl-*N2*-(prop-2-yn-1-yl)quinazoline-2,4-diamine (I-6f)**

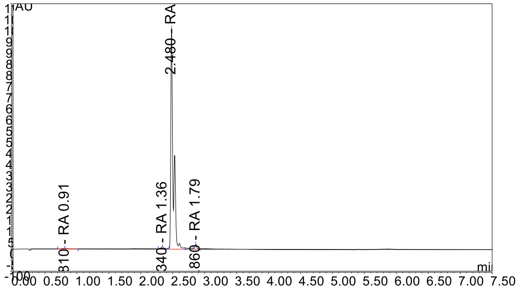

Fig. S102. **HRMS spectrum for *N4*-cyclopropyl-*N2*-methyl-*N2*-(prop-2-yn-1-yl)quinazoline-2,4-diamine (I-6g)**

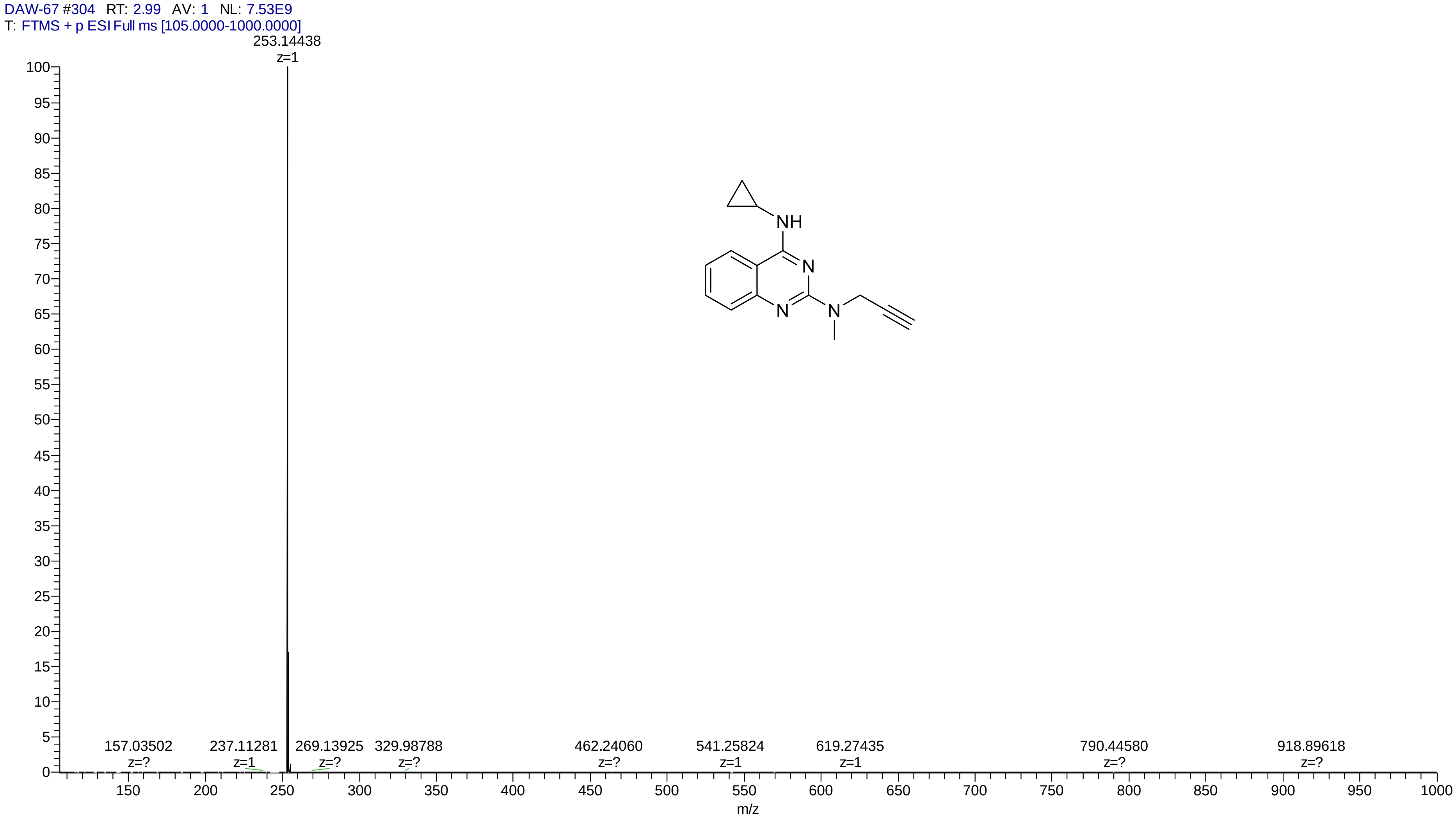

Fig. S103. **LC-UV chromatogram for *N4*-cyclopropyl-*N2*-methyl-*N2*-(prop-2-yn-1-yl)quinazoline-2,4-diamine (I-6g)**

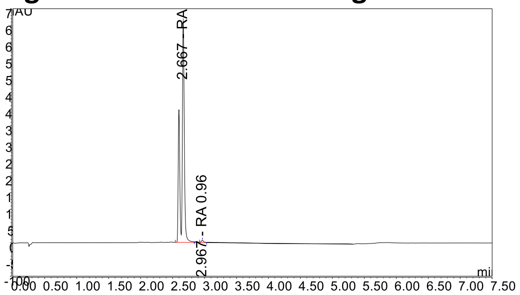

Fig. S104. HRMS spectrum for *N4*-cyclohexyl-*N2*-methyl-*N2*-(prop-2-yn-1-yl)quinazoline-2,4-diamine (I-6h)

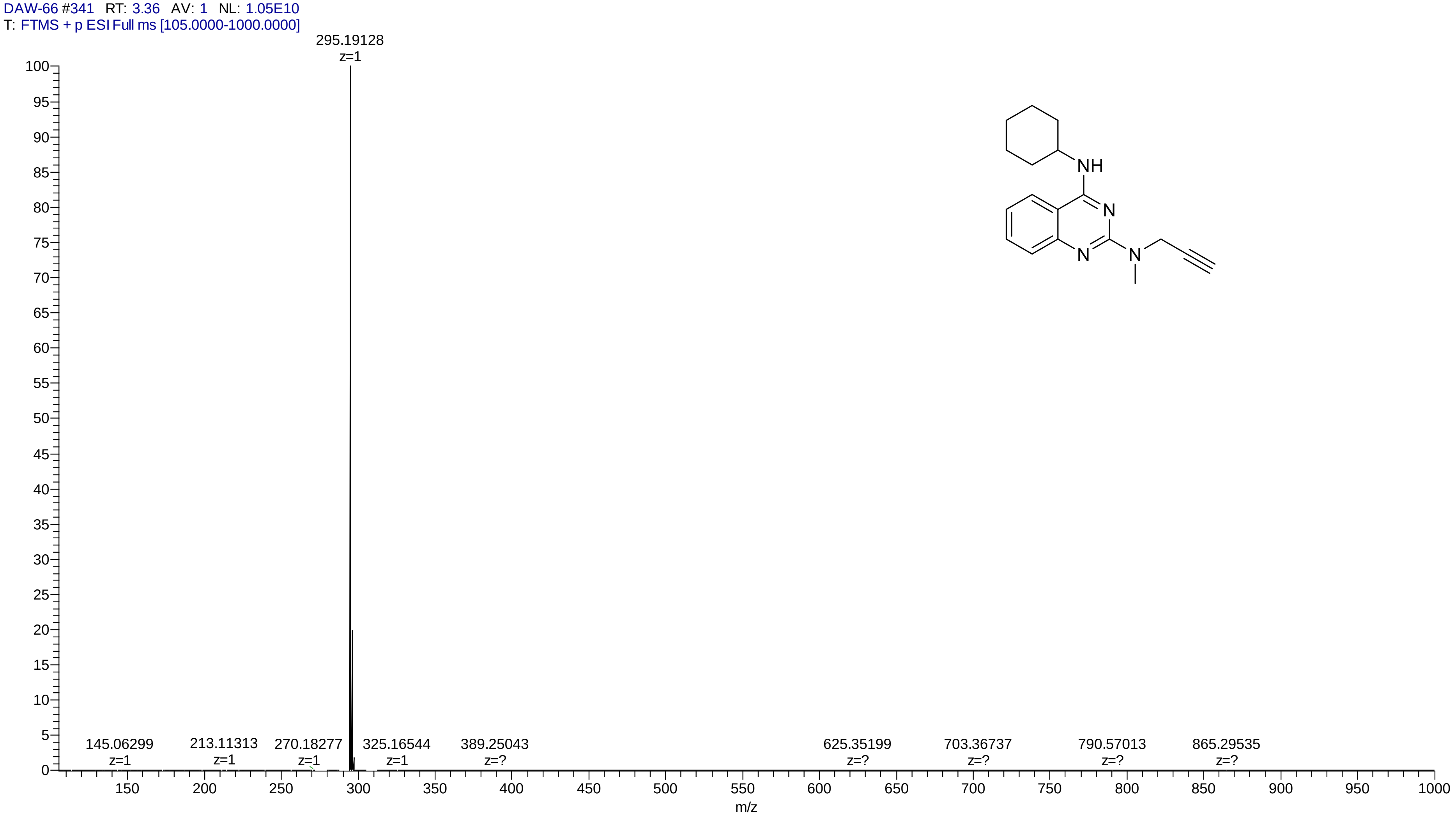

Fig. S105. **LC-UV chromatogram for *N4*-cyclohexyl-*N2*-methyl-*N2*-(prop-2-yn-1-yl)quinazoline-2,4-diamine (I-6h)**

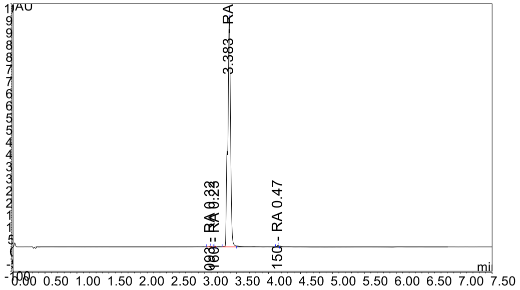

Fig. S106. **HRMS spectrum for 7-chloro-*N2*-methyl-*N2*-(prop-2-yn-1-yl)quinazoline-2,4-diamine (II-6a)**

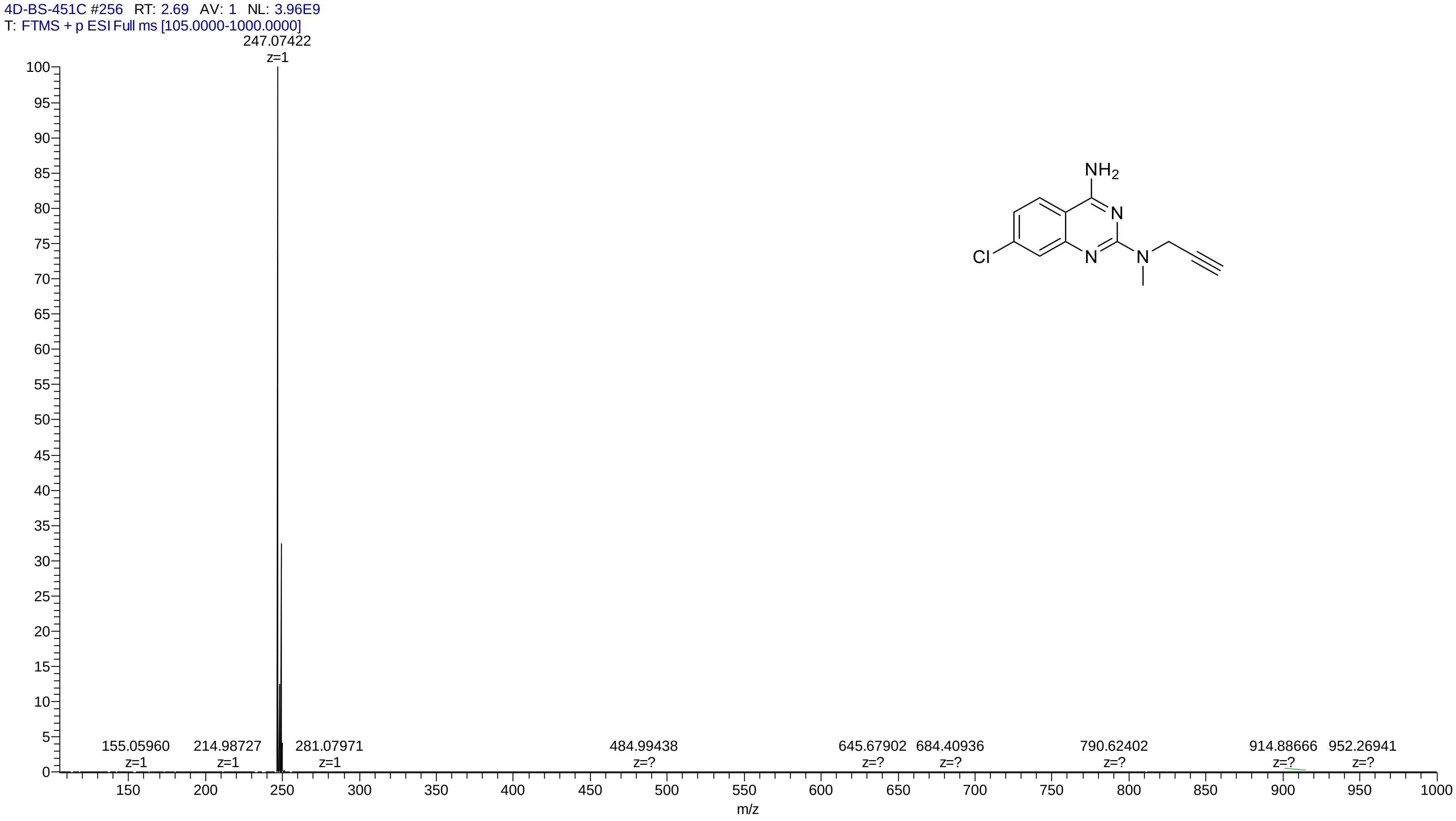

Fig. S107. **LC-UV chromatogram for 7-chloro-*N2*-methyl-*N2*-(prop-2-yn-1-yl)quinazoline-2,4-diamine (II-6a)**

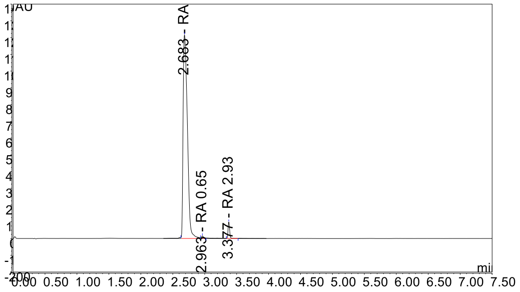

Fig. S108. **HRMS spectrum for 7-chloro-*N2*,*N4*-dimethyl-*N2*-(prop-2-yn-1-yl)quinazoline-2,4-diamine (II-6b)**

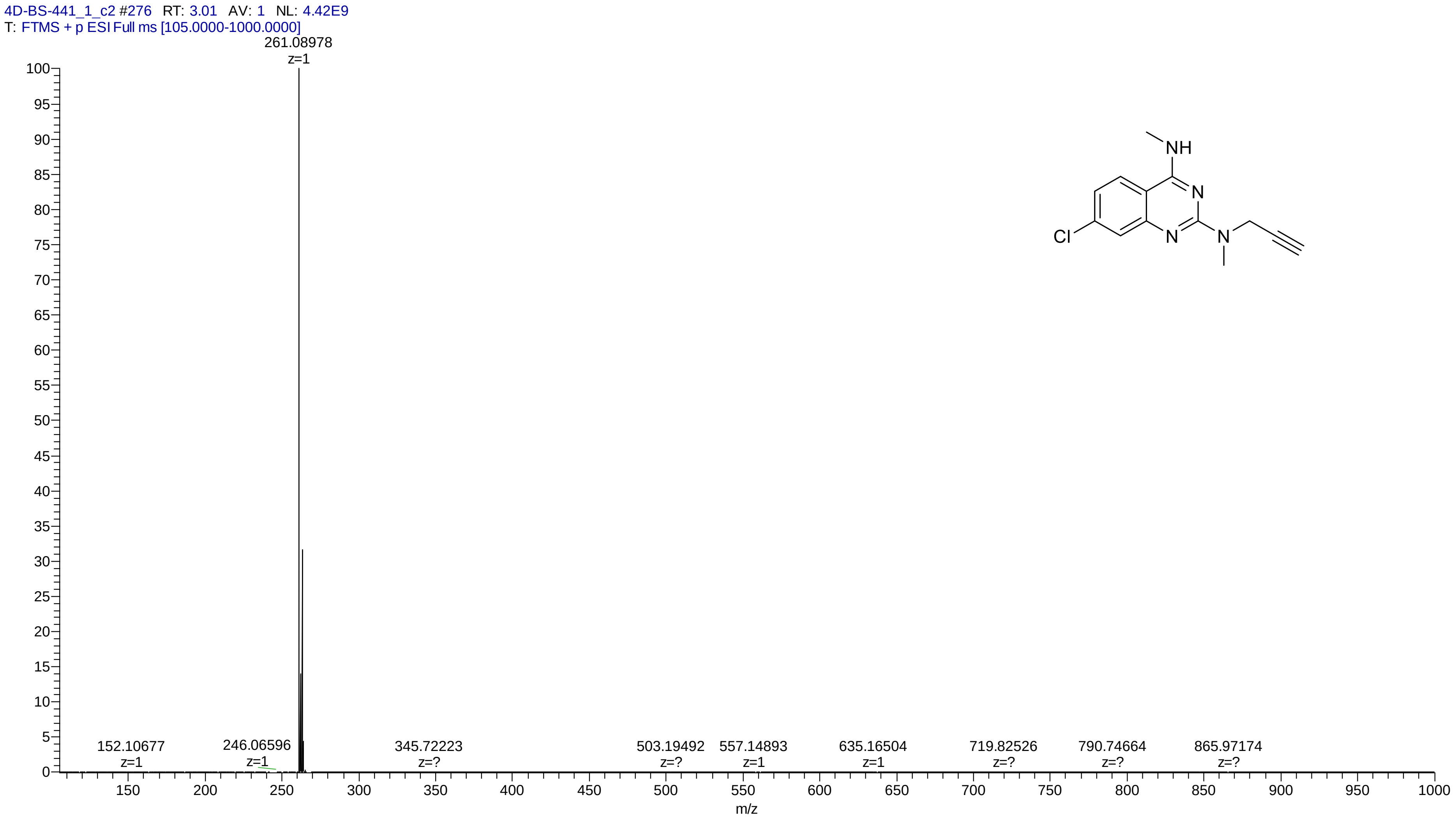

Fig. S109. LC-UV chromatogram for 7-chloro-*N2*,*N4*-dimethyl-*N2*-(prop-2-yn-1-yl)quinazoline-2,4-diamine (II-6b)

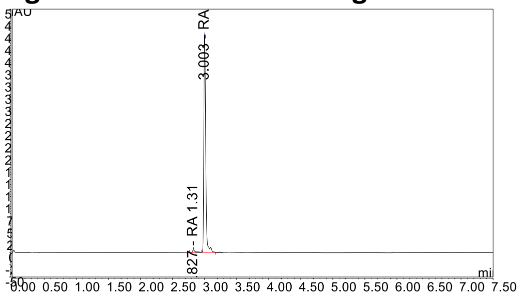

Fig. S110. **HRMS spectrum for 7-chloro-*N4*-ethyl-*N2*-methyl-*N2*-(prop-2-yn-1-yl)quinazoline-2,4-diamine (II-6c)**

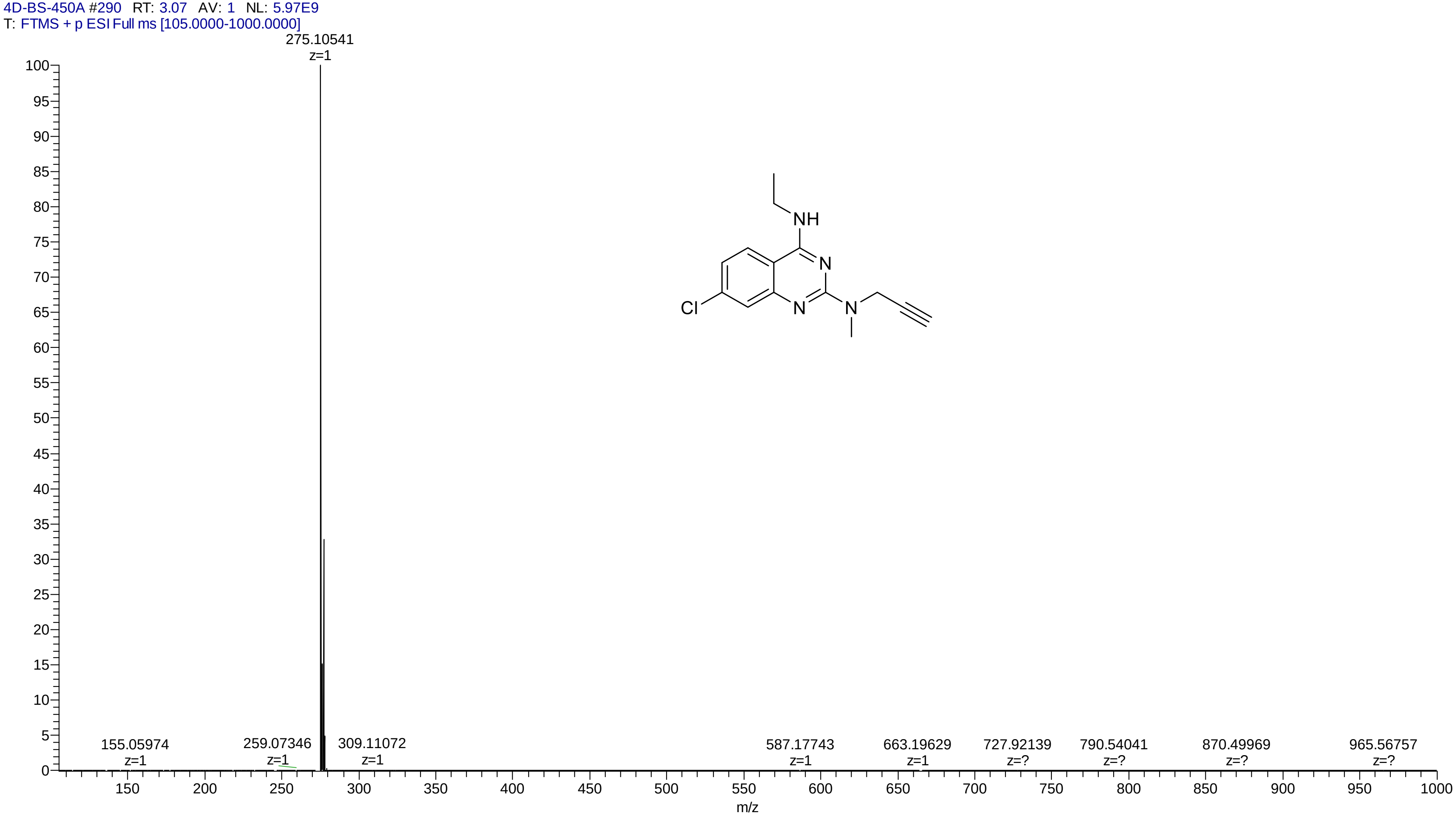

Fig. S111. **LC-UV chromatogram for 7-chloro-*N4*-ethyl-*N2*-methyl-*N2*-(prop-2-yn-1-yl)quinazoline-2,4-diamine (II-6c)**

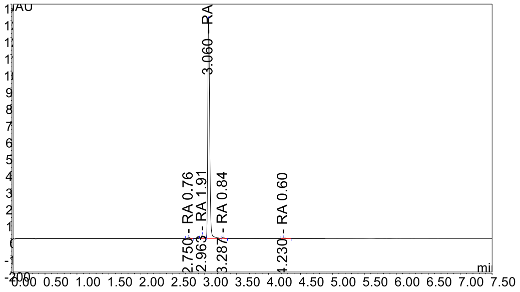

Fig. S112. **HRMS spectrum for 7-chloro-*N2*-methyl-*N2*-(prop-2-yn-1-yl)-*N4*-(propan-2-yl)quinazoline-2,4-diamine (II-6d)**

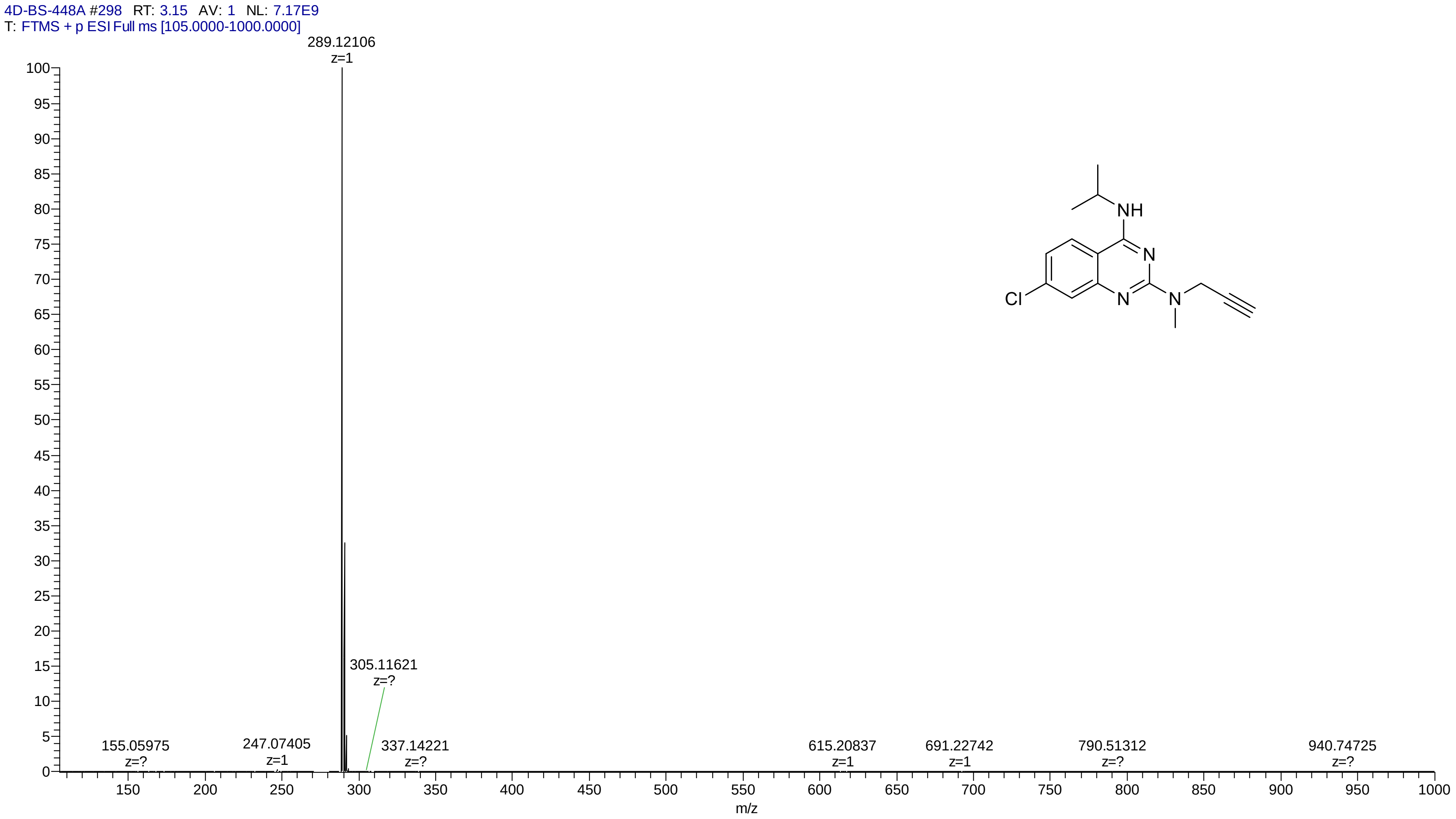

Fig. S113. LC-UV chromatogram for 7-chloro-*N2*-methyl-*N2*-(prop-2-yn-1-yl)-*N4*-(propan-2-yl)quinazoline-2,4-diamine (II-6d)

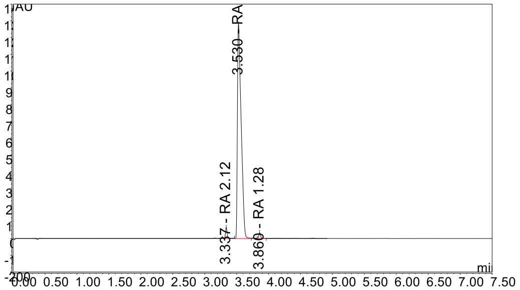

Fig. S114. **HRMS spectrum for *N4*-butyl-7-chloro-*N2*-methyl-*N2*-(prop-2-yn-1-yl)quinazoline-2,4-diamine (II-6e)**

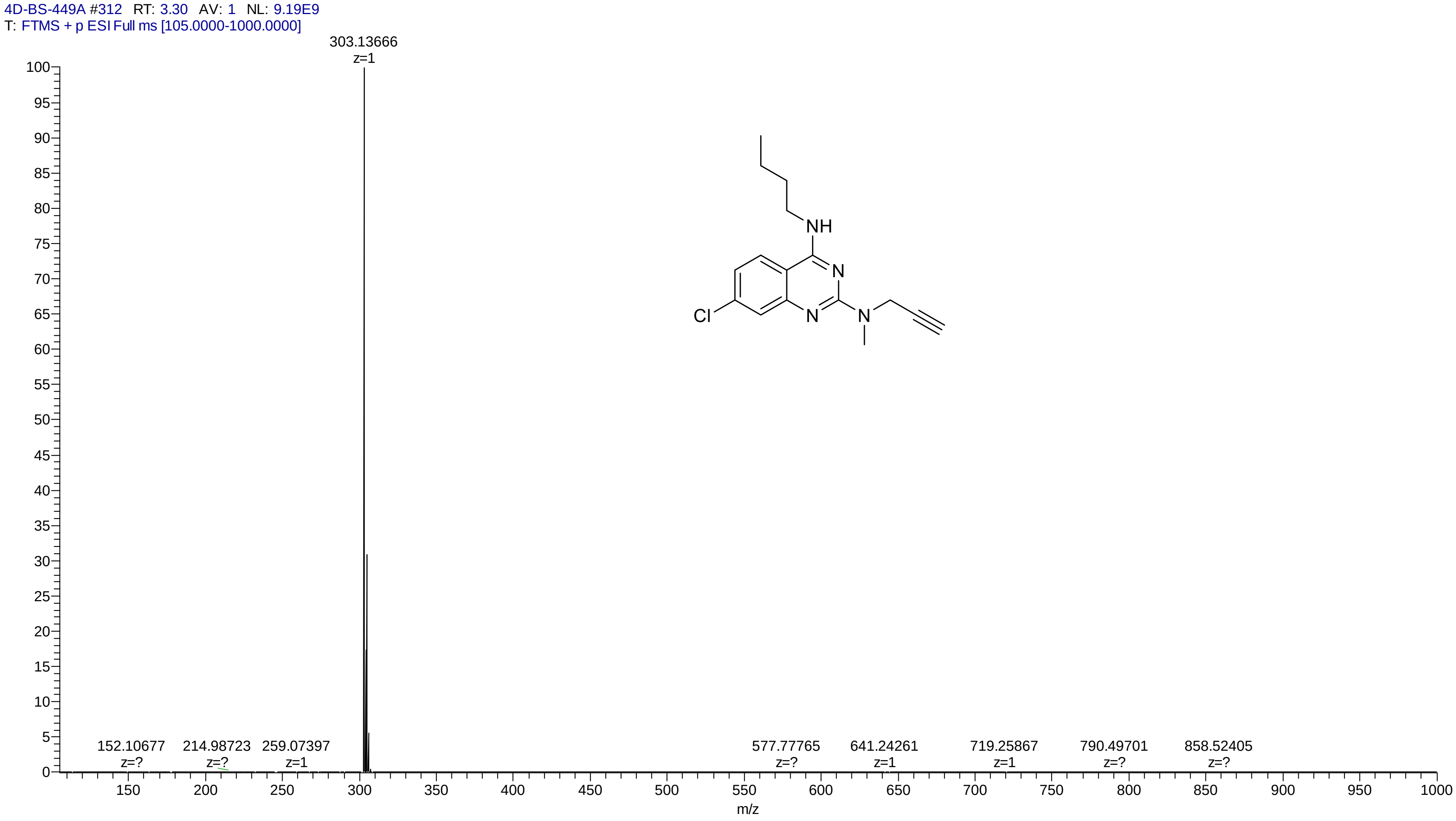

Fig. S115. **LC-UV chromatogram for *N4*-butyl-7-chloro-*N2*-methyl-*N2*-(prop-2-yn-1-yl)quinazoline-2,4-diamine (II-6e)**

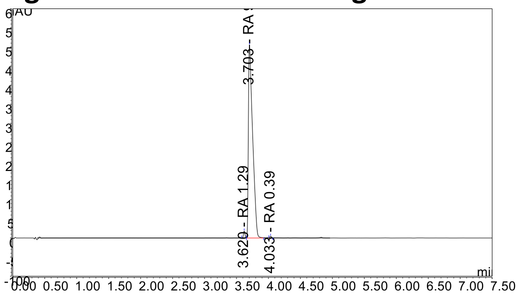

Fig. S116. **HRMS spectrum for 7-chloro-*N4*-(2-methoxyethyl)-*N2*-methyl-*N2*-(prop-2-yn-1-yl)quinazoline-2,4-diamine (II-6f)**

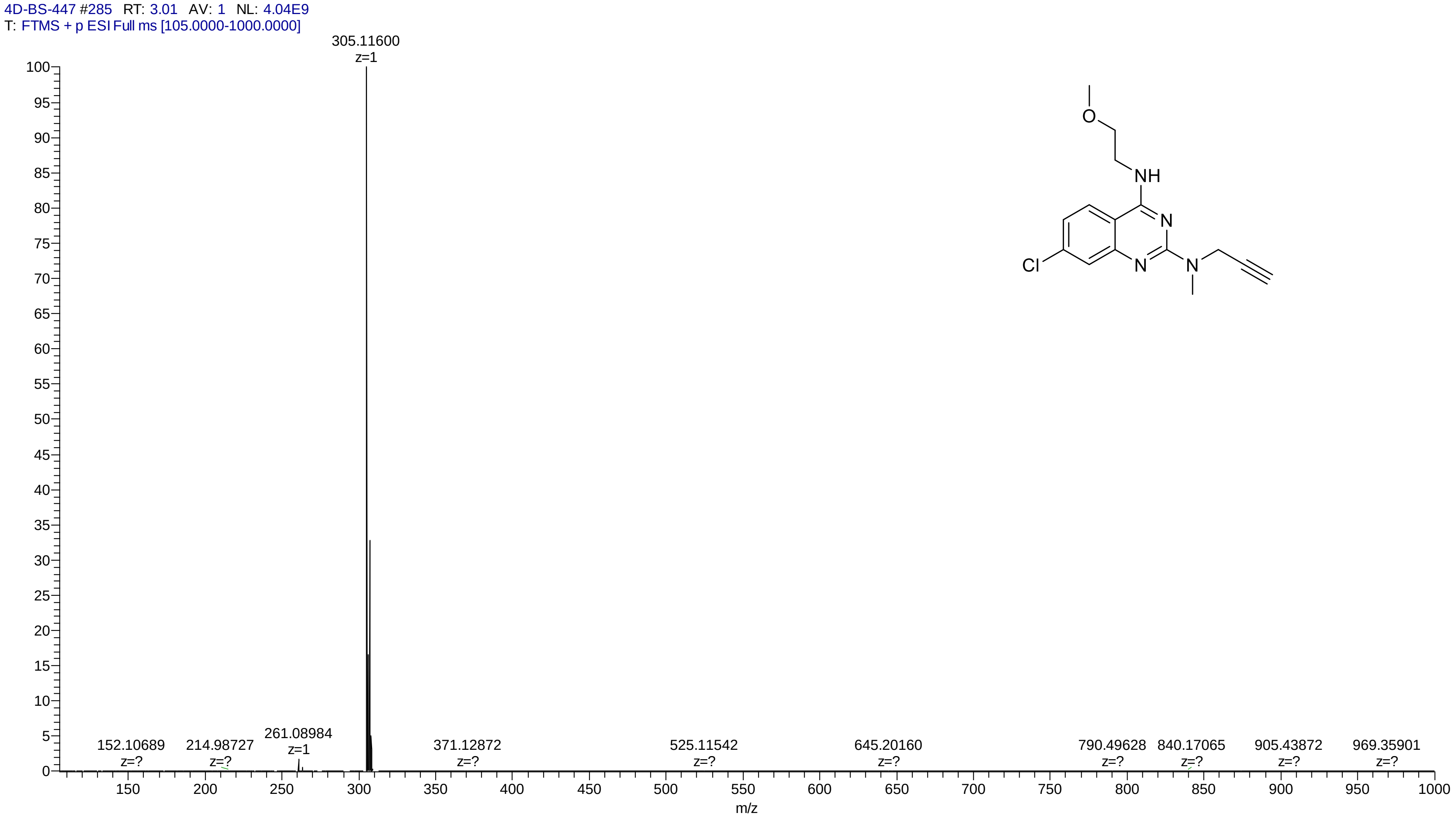

Fig. S117. **LC-UV chromatogram for 7-chloro-*N4*-(2-methoxyethyl)-*N2*-methyl-*N2*-(prop-2-yn-1-yl)quinazoline-2,4-diamine (II-6f)**

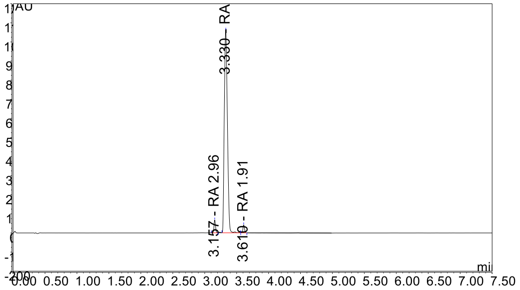

Fig. S118. **HRMS spectrum for 7-chloro-*N4*-cyclopropyl-*N2*-methyl-*N2*-(prop-2-yn-1-yl)quinazoline-2,4-diamine (II-6g)**

4D-BS-440_1A #315 RT: 3.11 AV: 1 NL: 3.22E9
T: FTMS + p ESI Full ms [105.0000-1000.0000]

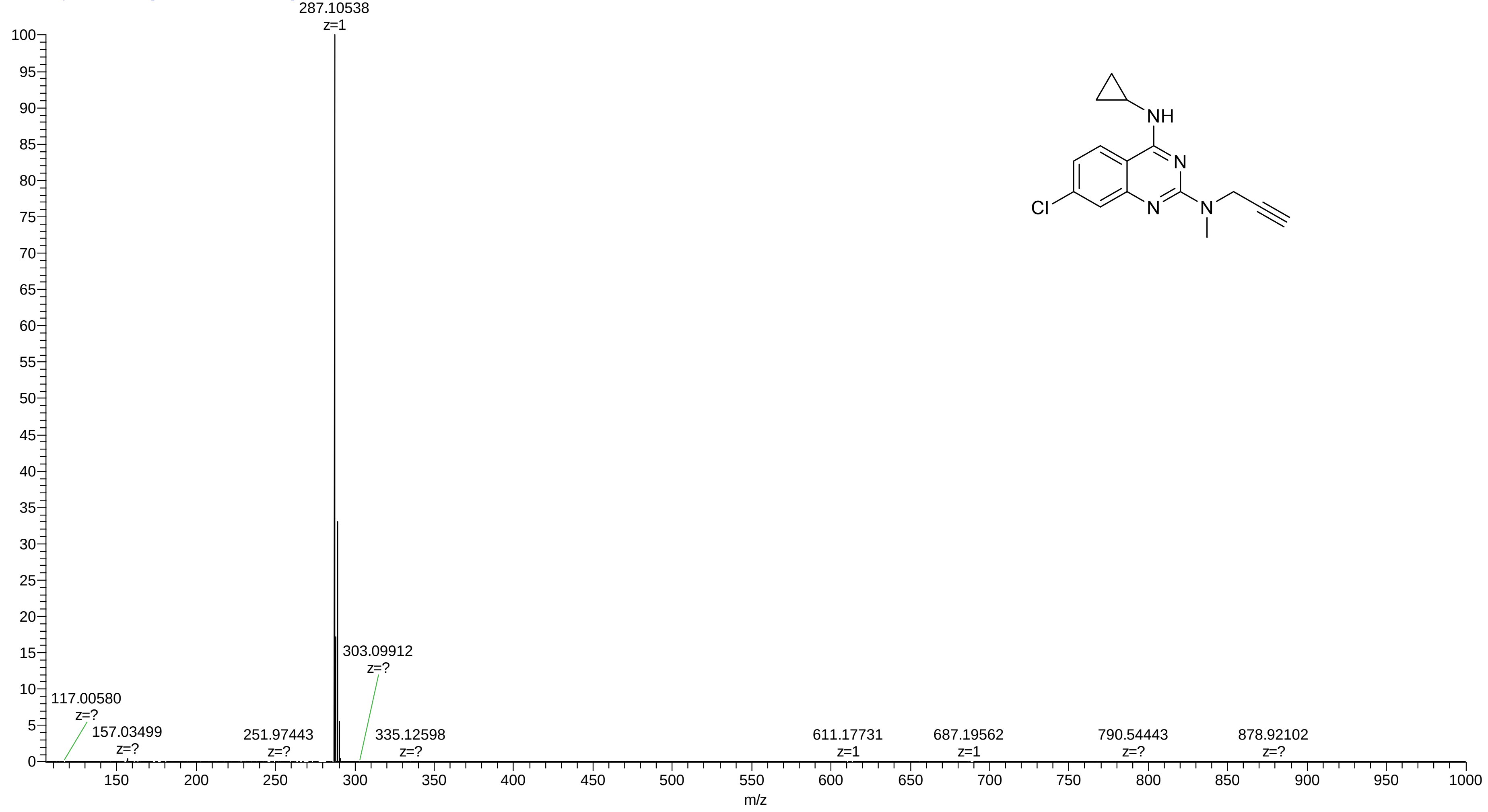

Fig. S119. **LC-UV chromatogram for 7-chloro-*N4*-cyclopropyl-*N2*-methyl-*N2*-(prop-2-yn-1-yl)quinazoline-2,4-diamine (II-6g)**

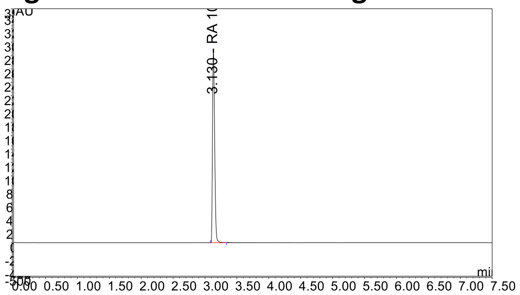

Fig. S120. HRMS spectrum for 7-chloro-*N4*-cyclohexyl-*N2*-methyl-*N2*-(prop-2-yn-1-yl)quinazoline-2,4-diamine (II-6h)

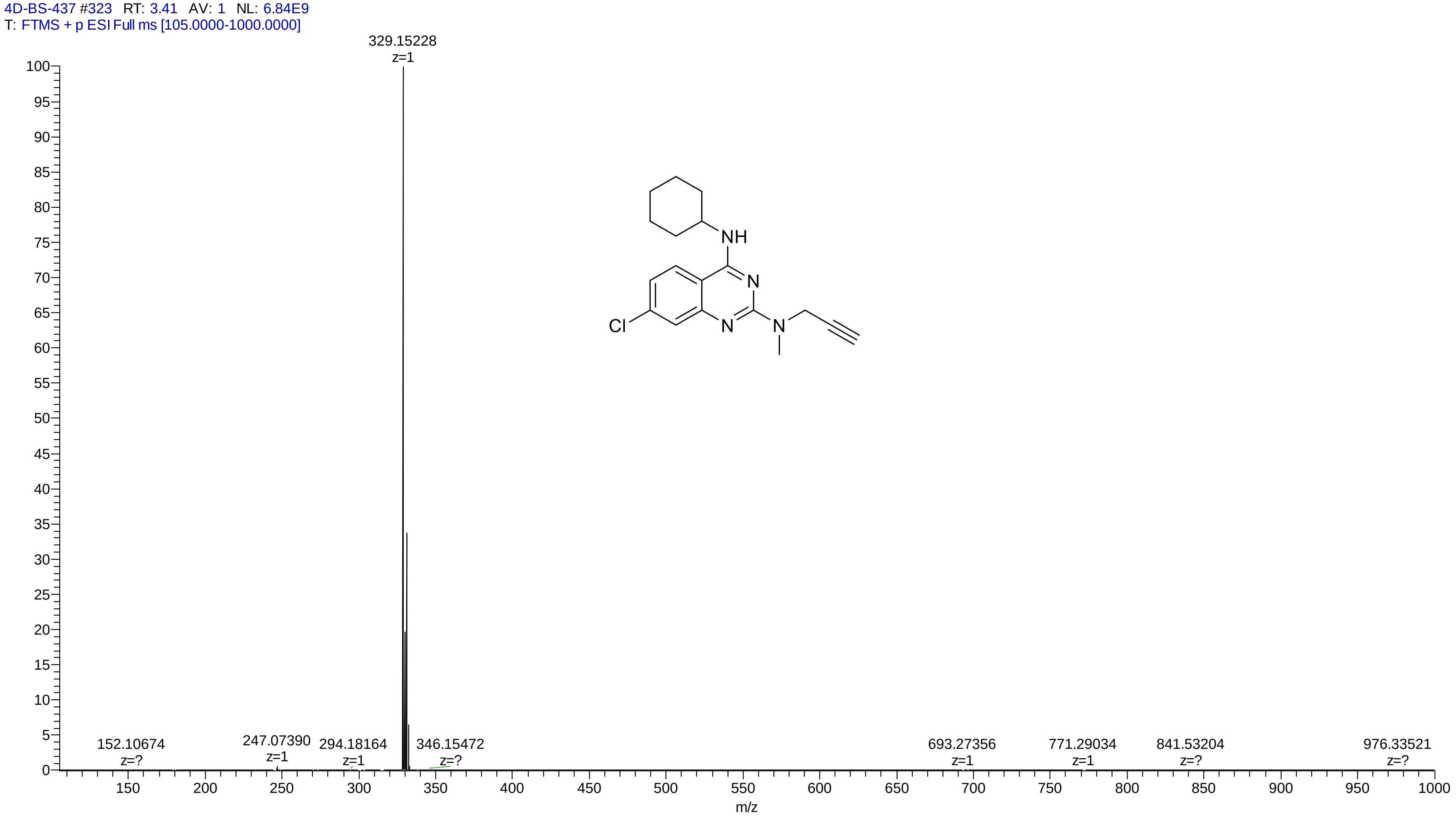

Fig. S121. LC-UV chromatogram for 7-chloro-*N4*-cyclohexyl-*N2*-methyl-*N2*-(prop-2-yn-1-yl)quinazoline-2,4-diamine (II-6h)

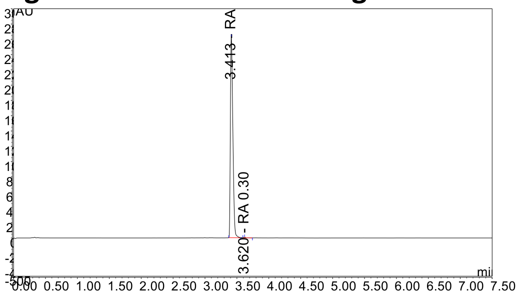

Fig. S122. **HRMS spectrum for 6-methoxy-*N2*-methyl-*N2*-(prop-2-yn-1-yl)quinazoline-2,4-diamine (III-6a)**

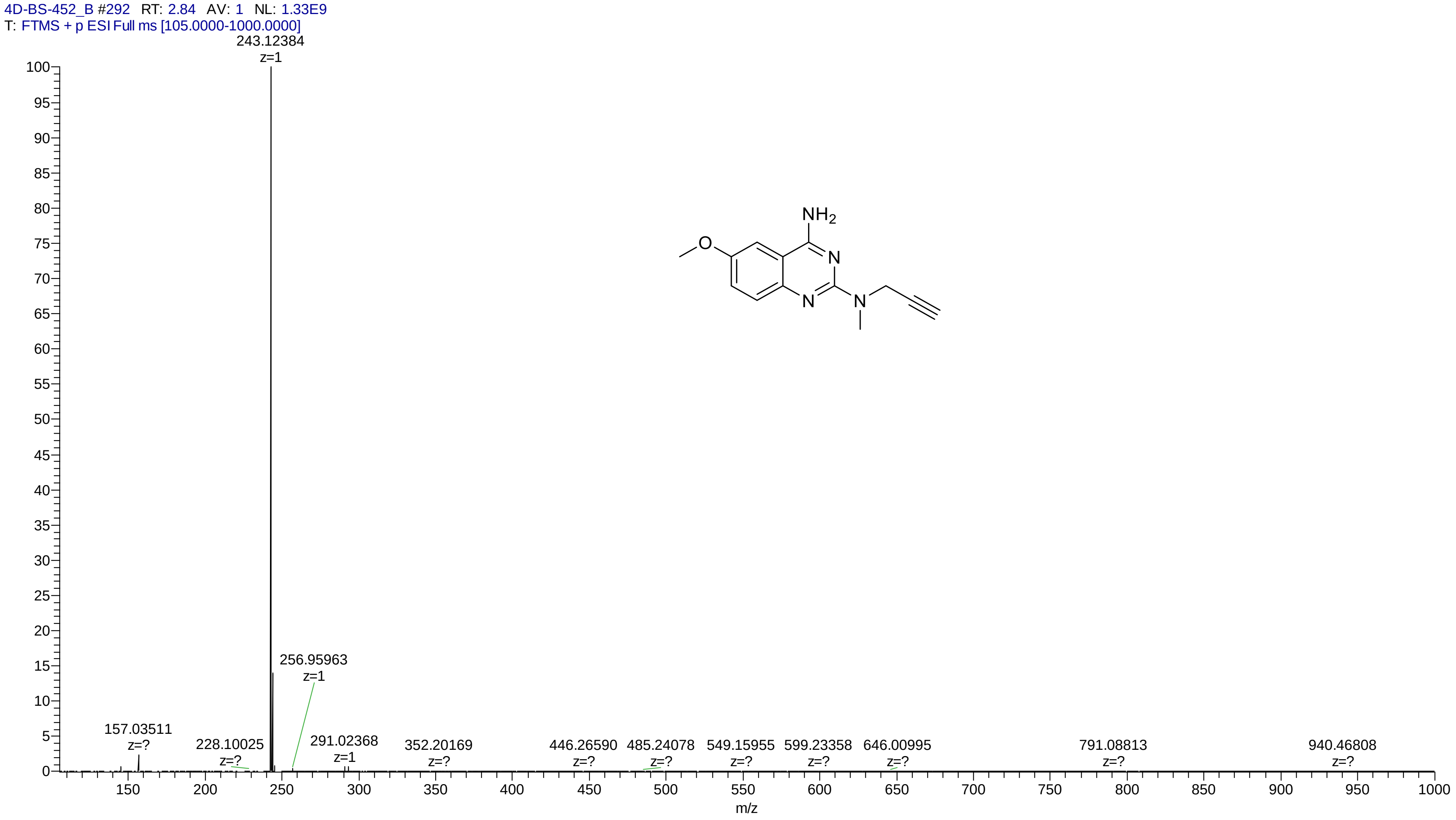

Fig. S123. **LC-UV chromatogram for 6-methoxy-*N2*-methyl-*N2*-(prop-2-yn-1-yl)quinazoline-2,4-diamine (III-6a)**

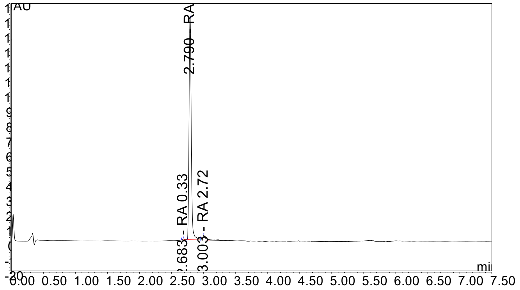

Fig. S124. **HRMS spectrum for 6-methoxy-*N2*,*N4*-dimethyl-*N2*-(prop-2-yn-1-yl)quinazoline-2,4-diamine (III-6b)**

4D-BS-427_1 #282 RT: 3.04 AV: 1 NL: 8.07E9
T: FTMS + p ESI Full ms [105.0000-1000.0000]

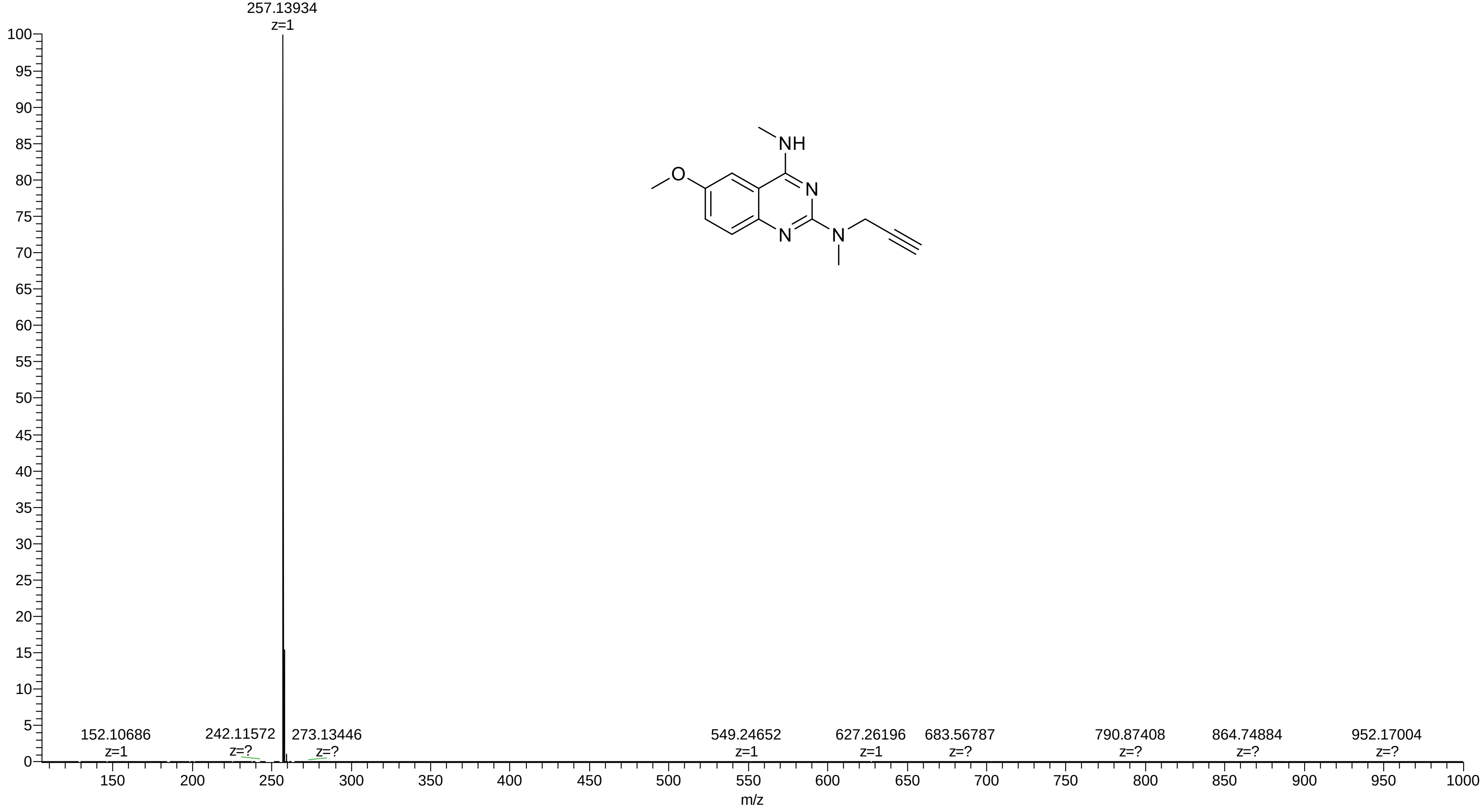

Fig. S125. **LC-UV chromatogram for 6-methoxy-*N2*,*N4*-dimethyl-*N2*-(prop-2-yn-1-yl)quinazoline-2,4-diamine (III-6b)**

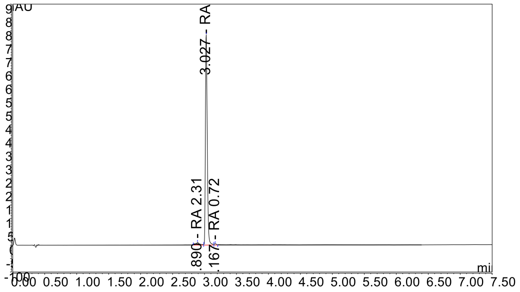

Fig. S126. **HRMS spectrum for *N4*-ethyl-6-methoxy-*N2*-methyl-*N2*-(prop-2-yn-1-yl)quinazoline-2,4-diamine (III-6c)**

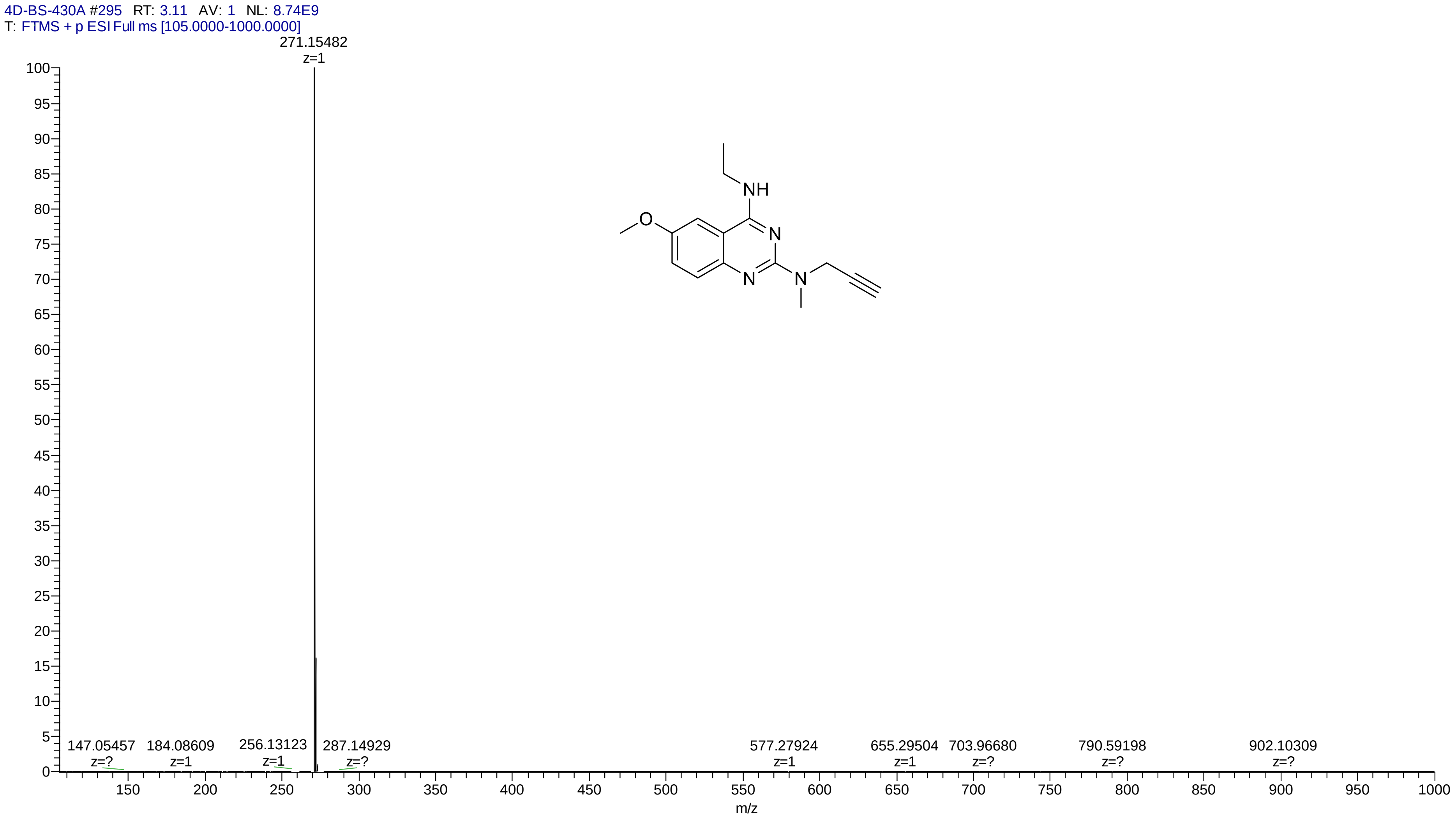

Fig. S127. LC-UV chromatogram for *N4*-ethyl-6-methoxy-*N2*-methyl-*N2*-(prop-2-yn-1-yl)quinazoline-2,4-diamine (III-6c)

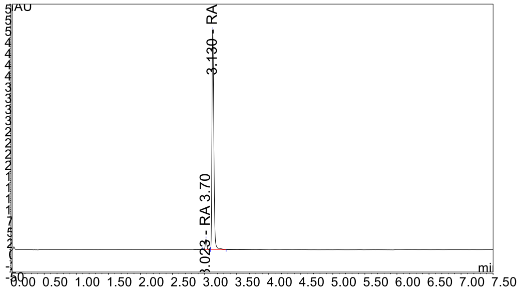

Fig. S128. HRMS spectrum for 6-methoxy-*N2*-methyl-*N2*-(prop-2-yn-1-yl)-*N4*-(propan-2-yl)quinazoline-2,4-diamine (III-6d)

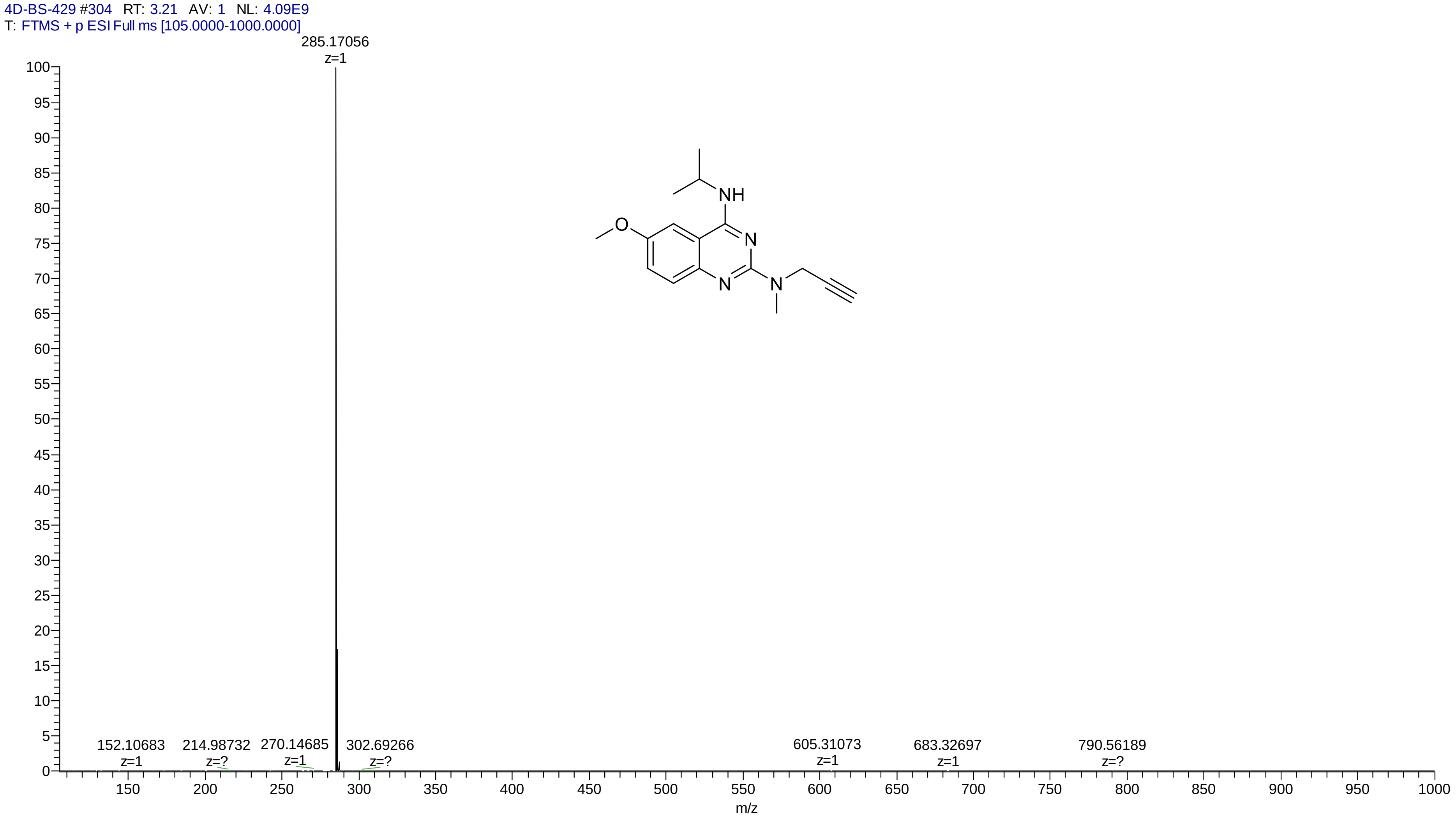

Fig. S129. LC-UV chromatogram for 6-methoxy-*N2*-methyl-*N2*-(prop-2-yn-1-yl)-*N4*-(propan-2-yl)quinazoline-2,4-diamine (III-6d)

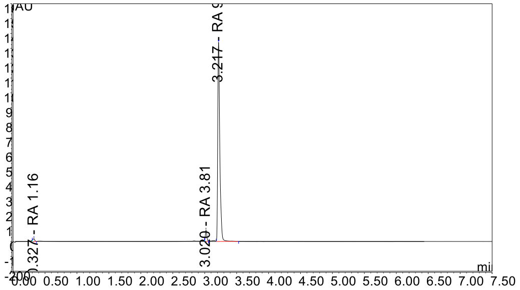

Fig. S130. **HRMS spectrum for *N4*-butyl-6-methoxy-*N2*-methyl-*N2*-(prop-2-yn-1-yl)quinazoline-2,4-diamine (III-6e)**

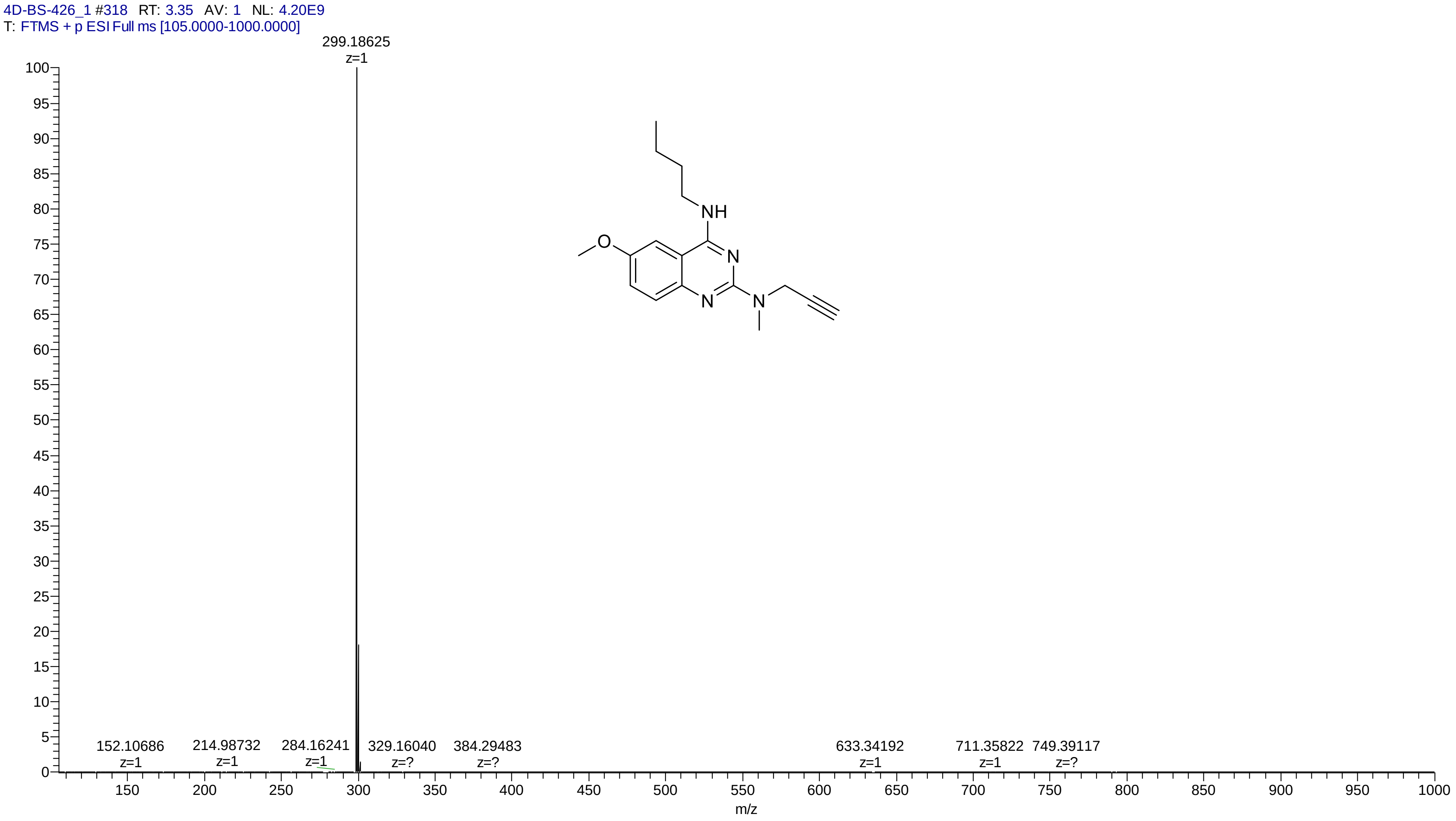

Fig. S131. LC-UV chromatogram for ***N4***-butyl-6-methoxy-***N2***-methyl-***N2***-(prop-2-yn-1-yl)quinazoline-2,4-diamine (III-6e)

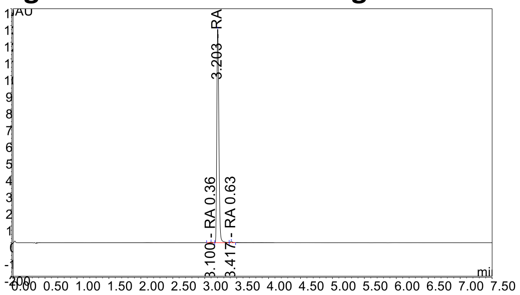

Fig. S132. HRMS spectrum for 6-methoxy-*N4*-(2-methoxyethyl)-*N2*-methyl-*N2*-(prop-2-yn-1-yl)quinazoline-2,4-diamine (III-6f)

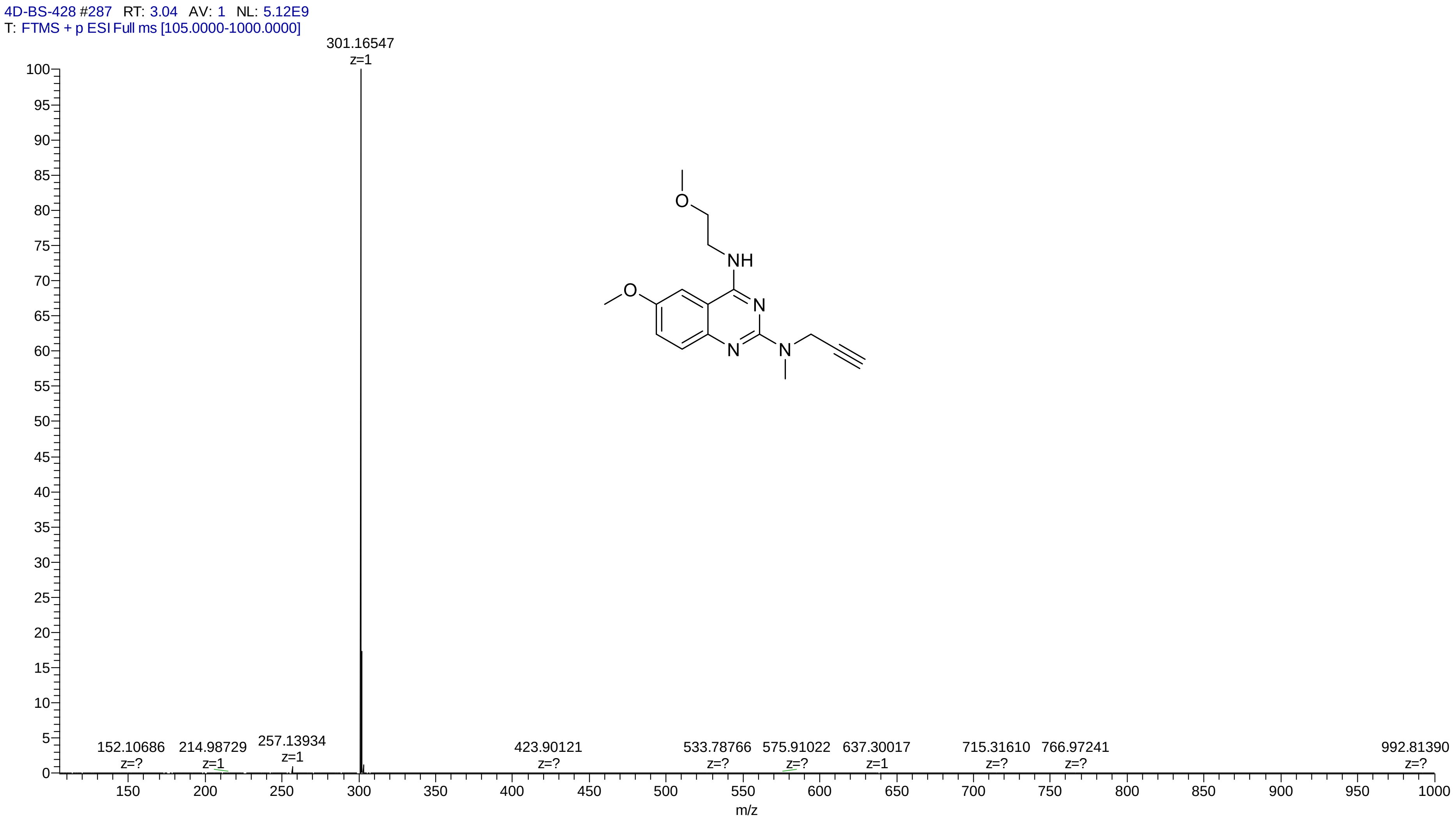

Fig. S133. **LC-UV chromatogram for 6-methoxy-*N4*-(2-methoxyethyl)-*N2*-methyl-*N2*-(prop-2-yn-1-yl)quinazoline-2,4-diamine (III-6f)**

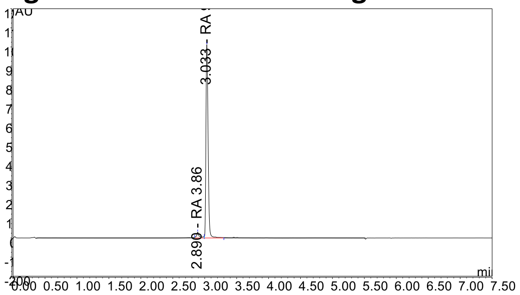

Fig. S134. **HRMS spectrum for *N4*-cyclopropyl-6-methoxy-*N2*-methyl-*N2*-(prop-2-yn-1-yl)quinazoline-2,4-diamine (III-6g)**

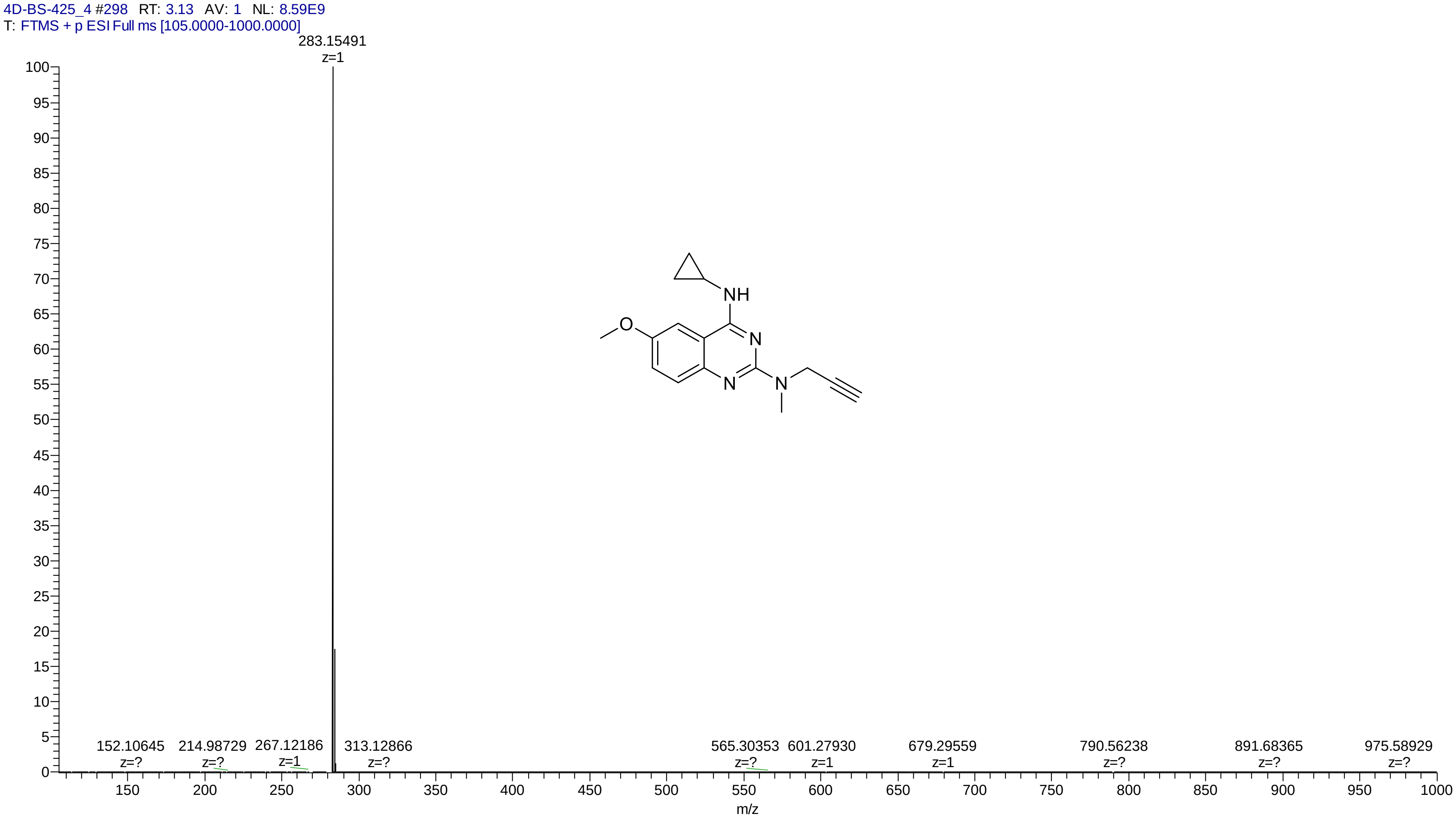

Fig. S135. LC-UV chromatogram for ***N4***-cyclopropyl-6-methoxy-***N2***-methyl-***N2***-(prop-2-yn-1-yl)quinazoline-2,4-diamine (III-6g)

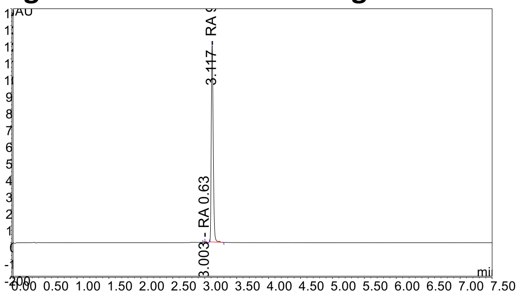

Fig. S136. HRMS spectrum for *N4*-cyclohexyl-6-methoxy-*N2*-methyl-*N2*-(prop-2-yn-1-yl)quinazoline-2,4-diamine (III-6h)

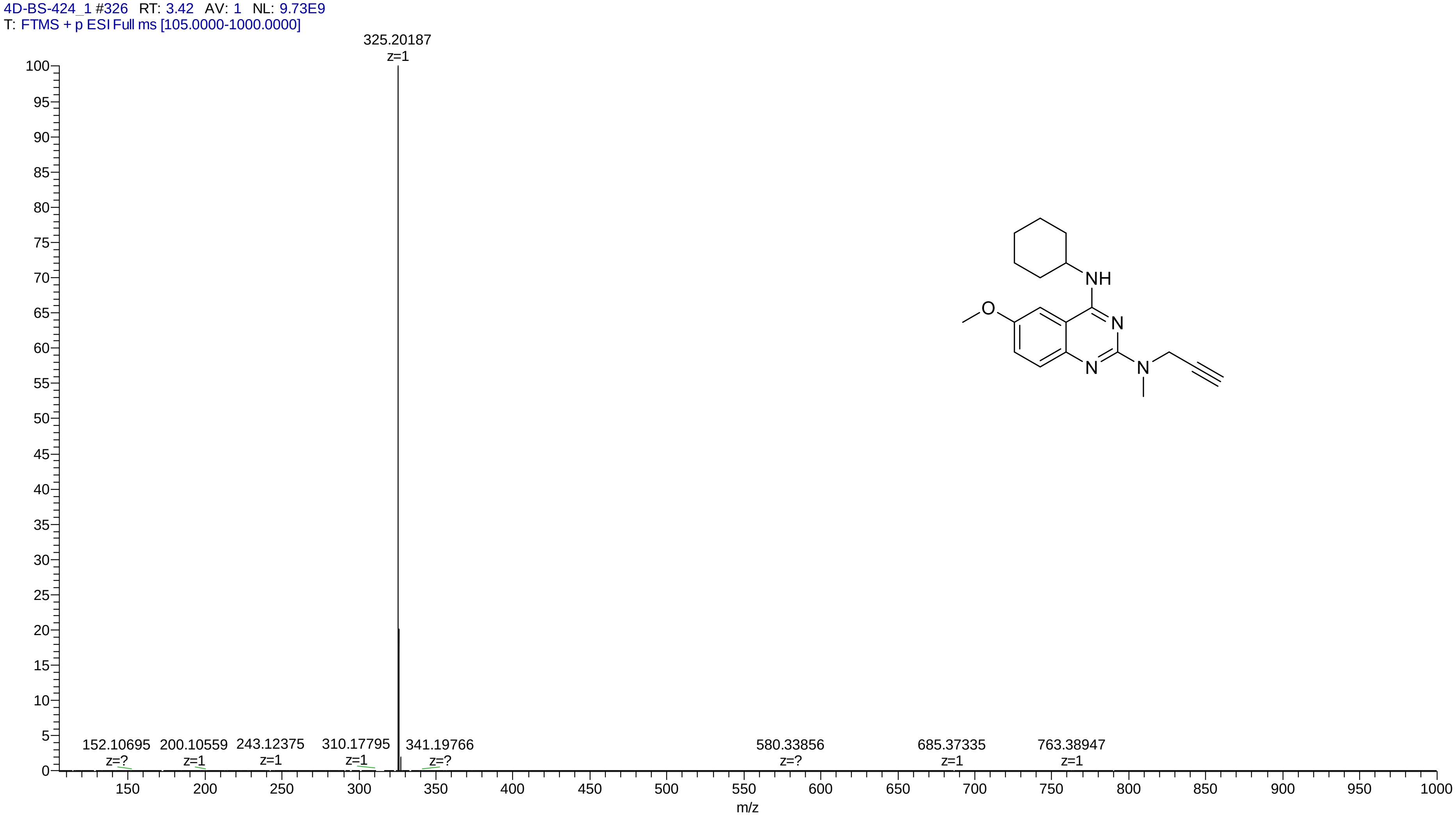

Fig. S137. **LC-UV chromatogram for *N4*-cyclohexyl-6-methoxy-*N2*-methyl-*N2*-(prop-2-yn-1-yl)quinazoline-2,4-diamine (III-6h)**

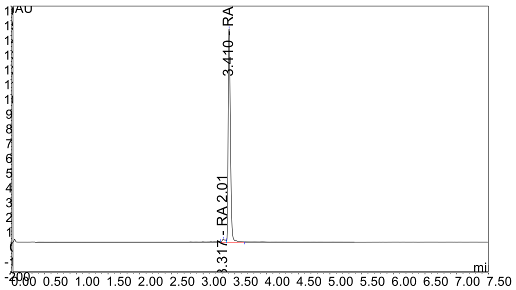

6. References

1. Daina, A.; Michielin, O.; Zoete, V. SwissADME: A Free Web Tool to Evaluate Pharmacokinetics, Drug-Likeness and Medicinal Chemistry Friendliness of Small Molecules. *Sci. Rep.* **2017**, *7*, 42717, doi:10.1038/srep42717.
2. Lipinski, C.A. Lead- and Drug-like Compounds: The Rule-of-Five Revolution. *Drug Discov. Today Technol.* **2004**, *1*, 337–341, doi:10.1016/j.ddtec.2004.11.007.
3. Ghose, A.K.; Viswanadhan, V.N.; Wendoloski, J.J. A Knowledge-Based Approach in Designing Combinatorial or Medicinal Chemistry Libraries for Drug Discovery. 1. A Qualitative and Quantitative Characterization of Known Drug Databases. *J. Comb. Chem.* **1999**, *1*, 55–68, doi:10.1021/cc9800071.
4. Veber, D.F.; Johnson, S.R.; Cheng, H.-Y.; Smith, B.R.; Ward, K.W.; Kopple, K.D. Molecular Properties That Influence the Oral Bioavailability of Drug Candidates. *J. Med. Chem.* **2002**, *45*, 2615–2623, doi:10.1021/jm020017n.
5. Egan, W.J.; Merz, K.M.; Baldwin, J.J. Prediction of Drug Absorption Using Multivariate Statistics. *J. Med. Chem.* **2000**, *43*, 3867–3877, doi:10.1021/jm000292e.
6. Muegge, I.; Heald, S.L.; Brittelli, D. Simple Selection Criteria for Drug-like Chemical Matter. *J. Med. Chem.* **2001**, *44*, 1841–1846, doi:10.1021/jm015507e.
